# Enlightening the Path to Protein Engineering: Chemoselective Turn-On Probes for High-Throughput Screening of Enzymatic Activity

**DOI:** 10.1021/acs.chemrev.2c00304

**Published:** 2023-02-28

**Authors:** Sebastian Hecko, Astrid Schiefer, Christoffel P. S. Badenhorst, Michael J. Fink, Marko D. Mihovilovic, Uwe T. Bornscheuer, Florian Rudroff

**Affiliations:** † Institute of Applied Synthetic Chemistry, OC-163, TU Wien, Getreidemarkt 9, 1060 Vienna, Austria; ‡ Institute of Biochemistry, Dept. of Biotechnology & Enzyme Catalysis, University of Greifswald, Felix-Hausdorff-Str. 4, 17489 Greifswald, Germany; § Department of Chemistry and Chemical Biology, Harvard University, 12 Oxford St, Cambridge, Massachusetts 02138, United States

## Abstract

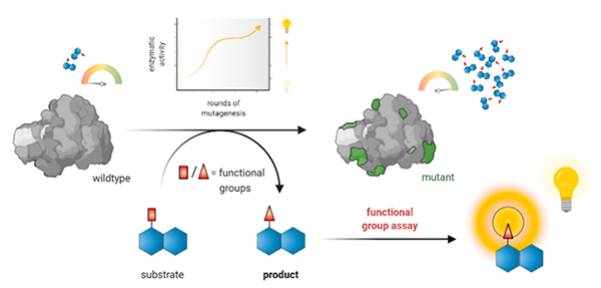

Many successful stories in enzyme engineering are based on the creation of randomized diversity in large mutant libraries, containing millions to billions of enzyme variants. Methods that enabled their evaluation with high throughput are dominated by spectroscopic techniques due to their high speed and sensitivity. A large proportion of studies relies on fluorogenic substrates that mimic the chemical properties of the target or coupled enzymatic assays with an optical read-out that assesses the desired catalytic efficiency indirectly. The most reliable hits, however, are achieved by screening for conversions of the starting material to the desired product. For this purpose, functional group assays offer a general approach to achieve a fast, optical read-out. They use the chemoselectivity, differences in electronic and steric properties of various functional groups, to reduce the number of false-positive results and the analytical noise stemming from enzymatic background activities. This review summarizes the developments and use of functional group probes for chemoselective derivatizations, with a clear focus on screening for enzymatic activity in protein engineering.

## 1 Introduction

Protein engineering involves the design and creation of proteins with specific, desired functionalities.^1^ For applications in synthetic chemistry, these efforts generally involve changing the substrate specificity, increasing the enzymatic activity, inverting and/or improving stereoselectivity, and increasing the stability of the biocatalysts to solvent, pH, temperature, proteolytic agents, *etc*.^2^ All modifications aim to complement or even completely replace reactions that use hazardous or environmentally unfavorable reagents or conditions. The demands of synthetic chemistry, however, do not necessarily align with nature’s repertoire of enzymatically catalyzed reactions.^3^ Consequently, tools for the improvement of existing and the development of new enzymatic catalysts are needed.

In recent years, it has become possible to use rational structure-based and *de novo* design to create new active enzymes. These new methods have been demonstrated to work in improving catalytic performance, broadening substrate range, increasing or inverting existing stereoselectivity, and in creating “new-to-nature” functionality^4^ to obtain products with high value for industrial applications.^5^ In contrast to specific design, methods that test all (or almost all members) of a given population (“screening”) are typically used when there is no prior knowledge of a protein structure or when rational design seems to only deliver incremental improvements.^6−8^

**Figure 1:**
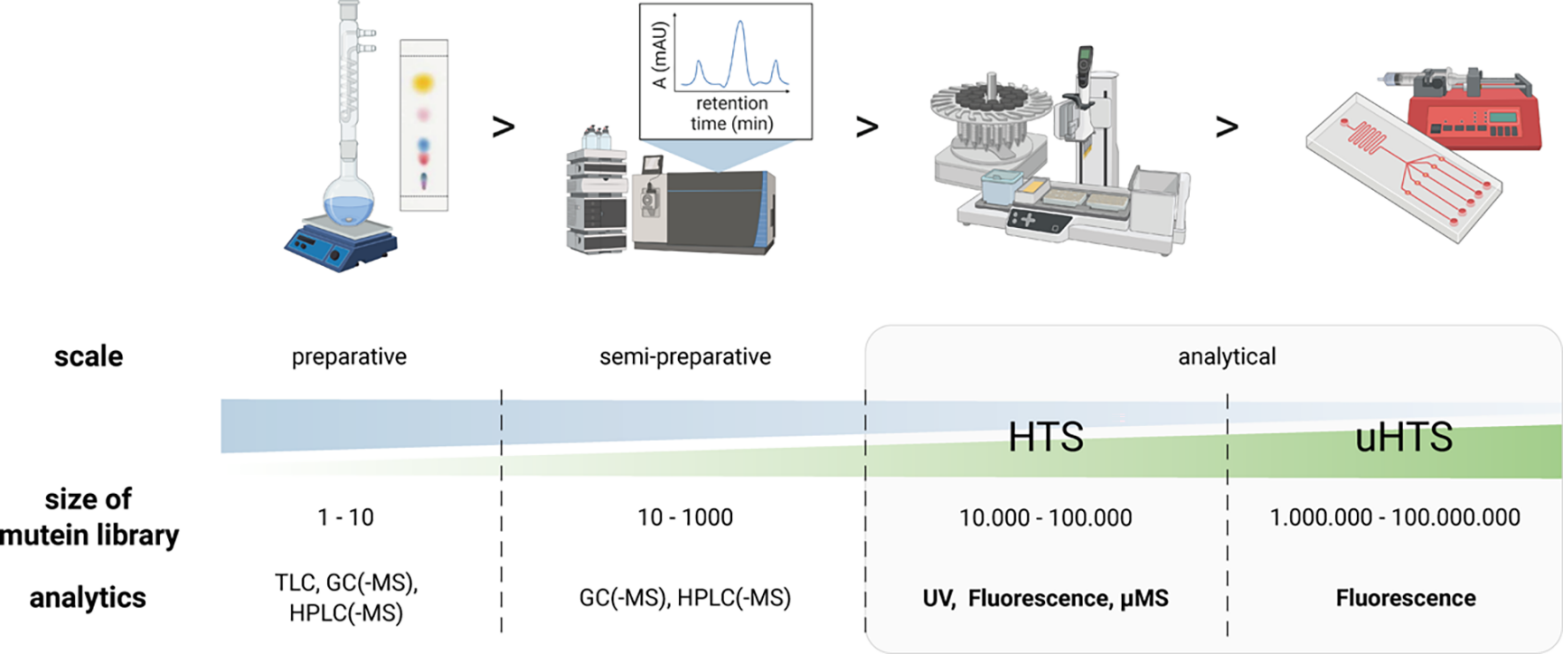
Change of analytical approach depending on screening scale and mutant library size: Increase in library size urges a shift from chromatographic methods to spectroscopic detection for (ultra) high-throughput screenings: (u)HTS. TLC: thin-layer chromatography; GC: gas chromatography; HPLC: high-performance liquid chromatography; (μ)MS: (microscale) mass spectrometry. Created with BioRender.com.

These screening methods are often subsumed under the term “directed evolution,” pioneered in the 1990s and awarded with the 2018 Nobel Prize in Chemistry.^9^ Random mutagenesis (by error-prone PCR or gene shuffling) enables the researcher to explore uncharted territory of chemical reactions by unguided substitutions of the genetic code, thus causing exchanges of single amino acids or regions of an enzyme. Goldsmith and Tawfik found a correlation between increases in activity and the number of mutations in the protein; large improvements (>10^3^-fold activity of the wild type) required an average of five mutations per 10-fold improvement in catalytic efficiency.^10^ A specific example: To cover the whole sequence space of a protein with 300 amino acids by using random mutations at five random positions, 1.9.7 × 10^12^ variants would have to be assessed.^11^ Such large numbers of experiments practically exclude the use of chromatographic methods (*e.g*., GC, HPLC) and can only be managed by spectroscopic techniques, using chromo- or fluorogenic reporters, with short analysis times, low detection limits, and high sensitivity (Figure 1).^6^

Many studies rely on chromo- or fluorogenic substrates that mimic the true target or on enzyme-coupled chromo- or fluorogenic assays for the detection of a wide range of catalytic activities.^12^ The major drawback of these methods is that they generate indirect experimental evidence of the desired activity but no analytical signal from the relevant transformation. The most reliable hits are achieved by detecting the target product.

Functional group assays (FGA) use the functionality of a target molecule as an analytical handle. They thereby reduce the number of false-positive results, and the analytical noise from enzymatic background activities.^13,14^ In this review, we summarize the development and use of functional group probes for the chemoselective detection of a broad range of target molecules in physiological media (*e.g*., aqueous buffered solutions, cell culture media). We approach the topic primarily in the context of protein engineering, focusing on the applicability of those tools to the development of new biocatalysts. With this summary, we wish to target two fields that are heavily involved in this area of research: biochemistry and synthetic organic chemistry. We aim to equip the biochemist with a toolset to pursue the discovery of valuable biocatalysts, and we hope to motivate organic chemists to identify and tackle underdeveloped areas in the field of assay development. The review is structured from a chemist’s point of view, ordered by functional groups typically encountered in molecules of interest in academic and industrial research and development: amines, alcohols, aldehydes, ketones, phenols, epoxides, diols, thiols, *etc*. This classification is intended to enable easy access to information and offer chemical solutions to challenges in screening for enzymatic activity. Compatible buffer ranges, spectral regions of the respective assay product, and the commercial availability of the presented probes are additionally highlighted.

## 2 Assays in High-Throughput Screening

High-throughput screening is essentially a technical method in which every detail matters. If a good photometric assay is available, a library of enzymes based on a single site-saturation mutagenesis (*e.g*., using the NNK codon) can be easily screened for activity in a single 96-well microtiter plate. A combinatorial library of two residues (using NNK codons, as before) requires about 4000 clones (for statistical reasons), or 10 384-well plates.^15^ When there are even more amino acids in the enzyme that need to be mutated and there is no better way of designing variants than combinatorics, the number of variants to be screened rapidly exceeds the (human) capacity for handling plates and performing manipulations, with or without automation. Miniaturization can offer a significant improvement to throughput, with certain implications on the applied downstream analytics used for the high-throughput screening (HTS; 10^4^–10^5^ variants to analyze), and the development of novel (ultra) high-throughput (uHTS; 10^6^–10^8^ variants) screening methods becomes critical.

There are various scalable methods to obtain experimental information about structure–property relationships in enzymes. Generally, the methods with the highest throughput are *in vitro* binding assays.^16^ The data they produce is sometimes rich enough to complete even complicated scientific tasks, such as solving a protein structure from a deep mutational scan. They are not suitable when the goal is to optimize a biocatalyst for conditions relevant in an industrial process.

Most approaches rely on the spectroscopic detection of target or reporter molecules from absorbance or fluorescence measurements in the visual range, or close to it. Whenever a chromatographic separation is included before detection, throughput drops greatly. Fluorescence-activated cell sorting (FACS) can be used if (i) the reaction is run in cells, (ii) the reporter or product is fluorescent, and (iii) it does not permeate between cells. Recent developments in microfluidic fluorescence-activated droplet sorting (FADS) circumvent the issue of cell permeability by compartmentalizing cells in picoliter-sized water-in-oil droplets but in turn require analytes to be hydrophilic to prevent cross-talk between droplets; applications are currently limited to very polar fluorophores.^17,18^ Only few metabolites of interest have useful absorbance or fluorescence, and thus artificial fluorogenic substrates are needed. These artificial probes are often custom-designed and synthesized for a single application only.

**Figure 2:**
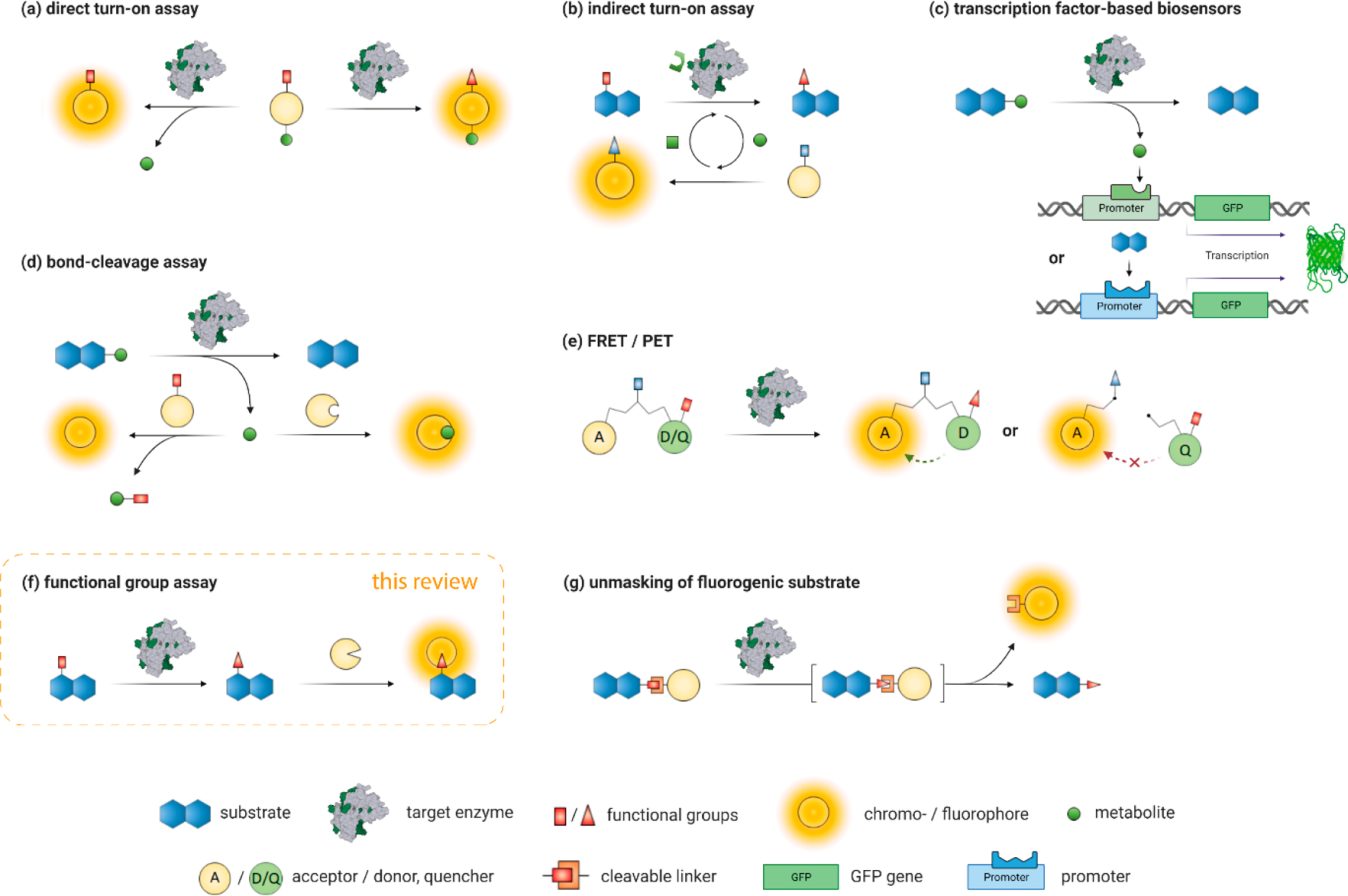
Overview of several ratiometric absorption or fluorescence turn-on strategies applied in directed enzyme evolution in biological systems: (a) direct detection of activity by functional group transformations or cleavage reaction of the target substrate, (b) indirect detection of conversion *via* dye cofactor recycling with a concomitant increase in signal, (c) triggered enhanced transcription of fluorescent biosensors, (d) formation of the dye in a downstream reaction upon cleavage of a metabolic reporter, detection *via* suitable recognition domain or subsequent reaction with a secondary chromo-/fluorogenic substrate, (e) induction of Förster resonance energy transfer (FRET) or suppression of photoinduced electron transfer (PET), (f) functional group transformation is detected *via* subsequent reaction with functional group selective assay component, (g) tandem reaction cascade of a model-substrate (mimic) unmasks chromo-/fluorophore.

High-throughput screening is still in its infancy and largely an academic pursuit where new approaches are being developed for linking enzymatic activity to a spectroscopic signal (Figure 2). While UV absorbance-based assays can detect a broad range of compounds, their sensitivity is usually lower than fluorescence-based ones. Compounds in crude cell lysates might interfere with the absorptive detection of the analyte of choice. Therefore, ratiometric “turn-on” probes, linking product formation to a proportional increase of a ratio of fluorescence signals, constitute the majority in the vast landscape of reporters (Figure 2).^13,19,20^ The increase in fluorescence intensity can result from various molecular implementations: (i) the inherent fluorescence of the desired product, formed by the enzymatic reaction (Figure 2a), (ii) a coupled and strongly correlated process that produces a fluorescent molecule, *e.g*., a cofactor recycling system (Figure 2b), (iii) enhanced transcription of fluorescent biosensors (Figure 2c), (iv) the concomitant formation of fluorescent metabolites (Figure 2d), (v) by induction of Förster resonance energy transfer (FRET) or suppression of photoinduced electron transfer (PET) processes (Figure 2e), or (vi) a tandem reaction producing a fluorescent molecule in a cascade, downstream of the targeted reaction (Figure 2g).

Not all the modes to generate a fluorescent signal listed above are equally applicable to any problem at hand. Given that many relevant products are not or only weakly fluorescent, direct monitoring of their formation is often not feasible (Figure 2a). Not all enzymatic transformations offer possibilities for indirect read-outs, *e.g*., from cofactor recycling (Figure 2b,d). Many protein engineering efforts thus use analytical tools based on fluorogenic substrates that mimic the chemical properties of the target compound to some degree while still being easy to synthesize.^12^ Screening for enzymatic activity with such mimics, however, bears the risk of identifying biocatalysts that are better at producing the mimic and not necessarily the target product. An aphorism capturing this issue, nicknamed as the First Law of Directed Evolution, that is often quoted, says: “You get what you screen for.”^21,22^ Assays based on mimics might be useful for proof-of-concept studies or the detection of new catalytic activity; they are not powerful in the optimization of biocatalysts.

Functional group probes (Figure 2f) use chemoselectivity, a result of steric and electronic properties of a functional group, as an analytical handle. This simplification, reducing the analytical complexity to a distinct interconversion of one functional group to another, often enables a clear discrimination of the desired enzymatic activity, even in the presence of substrates and interfering molecules from cellular background. Functional group probes offer an attractive option to reduce the number of false-positive results and the analytical noise from enzymatic or cellular background activities.^13,14^ Over the last decades, many functional group sensors have been developed, often benefiting from research in bioconjugation chemistry and *vice versa*. Among these examples, many have proven to be applicable in protein engineering, when HTS was used to test large libraries of enzyme variants.

## 3 About High Throughput

In any screening campaign, every manipulation decreases throughput. For instance, if one has to start with plating out cells after transformation, an agar-plate assay will likely have a higher throughput than one based on microtiter plates;^23^ or, transferring lysate from a deep-well plate (for cell growth) to an optical plate (for reading) halves throughput by doubling the number of plates to be handled. As a rule of thumb, throughput doubles when the analysis is performed in the same plate as the cultivation of the microorganism producing the enzyme. That also requires the assay reagents to not damage the DNA, which encodes the sequence of a potential hit.

Assays where reagents for detection at the end can be added from the beginning of the reaction (*e.g*., in a single master mix with the substrate) are better than assays with multiple components that need to be added in a certain order.^24^ There are limitations to such straightforward optimization of protocols. For instance, some reagents react with substrates or enzymes, inactivating them, or are unstable in water in general (*e.g*., fluorescamine **18**(25)) or at certain pH (*e.g*., DTNB **232**(26)). They can only be added at a later stage, when the chosen reaction time has passed or even just before analysis. The duration should be chosen in consideration of the dynamic range of the detection method without extra dilution (or concentration). Variations between wells on microtiter plates (in growth rates and/or expression levels, even for the same variant) are often large and significant, resulting in cases where relative specific activities cannot be estimated by merely quantifying the product. Continuous kinetic measurements are unsuitable because every moment one sample stays in the reader longer than necessary corresponds to other variants not being screened. Normalization to the actual enzyme content (*e.g*., using tagged-protein complementation assays like the split-GFP^27^ or split-luciferase assays^28^) is possible, but it can be expensive and time-consuming—it is therefore rarely done.

The power of HTS lies in producing information by interrogating many variants, not scrutinizing few. For the purpose of discovery, end-point measurements often provide information as good as kinetic measurements. For this reason, HTS is typically stronger in the discovery of new activities or the early rounds of improvement than in improving enzymes with substantial activity. This trend is a conundrum because initial improvements in activity are often simpler to achieve than further optimization: quantitative performance of the assay has to become more granular, more sensitive, and more reliable at later stages than in the beginning. Driven by this increasing challenge, HTS methods are used for tasks at which they do not excel by design, and work-arounds are introduced (*e.g.*, use of lower substrate concentrations, subsequent dilution steps). The dynamic range of assay systems thus needs to match the aim of the study (discovery or optimization).

The requirements for HTS-capable assay development are high. Ideally, an assay makes as few mechanical and fluidic manipulations as possible, use stable compounds, is sensitive, selective, simple, and has a large dynamic range. Any assay that can run at room temperature is simpler than one requiring heating or refrigeration. High temperatures, extreme pH, corrosive, or toxic chemicals and solvents (especially, volatiles and halogenated ones) should be avoided, as they can make assays incompatible with common equipment (plastic multiwell microtiter plates and their readers). It is critical that assays work under aqueous conditions because crude cell lysates are prepared using aqueous buffers. Therefore, this review focuses mainly on probes used to analyze aqueous samples.

## 4 Sensitivity and Selectivity

The best functional group probes are those where fluorescence solely depends on the concentration of the analyte. Such a simple relation can be typically observed in systems with only one enzyme and substrate present in the reaction. Many assays, however, require additional components (*e.g*., more than one substrate or enzyme, cofactors, activators or inhibitors, stabilizers, surfactants, cosolvents, detection reagents, *etc*.), making the selective detection of the main product challenging.

Crude cell lysates, which are the preferred formulation of enzymes used in HTS, compromise the detection because they introduce many intracellular metabolites in high concentrations, often much higher than the concentration of the product. For instance, in *Escherichia coli*, metabolites present in the mM range include several amino acids (glutamate, at 96 mM, aspartate, valine, glutamine, alanine, and citrulline), several nucleotide phosphates or thiols (*e.g*., glutathione, at 17 mM, or coenzyme A, at 1.4 mM).^29^ The high concentrations of these and other metabolites complicates the detection of low enzyme activities with rather nonspecific colorimetric probes. For example, the derivatization of primary amines using *o*-phthaladehyde (OPA, **22**) in the presence of 2-mercaptoethanol (ME)^24,30^ may be influenced by endogenous thiols because evolution of the signal is dependent on the thiol coupling partner.

Buffers too can obfuscate the results of an assay when they contain constituents that might interfere with the chemoselectivity of the probe. For instance, Good’s buffers contain amines, which may therefore compete with the detection of an amino functionality in the product.

Functional group assays work best when the functional group is being created from another in the reaction (*i.e*., a functional group interconversion, FGI). For example, carboxylate reductases produce aldehydes from carboxylic acids, amidases produce amines from amides, and thioesterases produce thiols from thioesters. In such straightforward cases, probes only need to discriminate one functional group from another and not between two of the same type. Hence, many probes are useful for FGI screening.^31^

In many other reactions (*e.g*., aminotransferases, isomerases, *etc*.), functional groups are not interconverted but moved around or between molecules, making chemoselectivity essential and challenging. Probes that cannot differentiate between substrate and product bearing the same functional group (*e.g*., by forming conjugates with different spectral properties from each) would require separation prior to detection (*e.g*., extraction or chromatography). Because the substrate is usually present in high concentration to saturate the enzyme (typically, ∼ 100-fold the *K*_M_ value), specificity for the product by ways of vastly different reaction rate constants is important (because with similar constants, specifically detecting a product at low concentration, is noisy against the background from the substrate).

Examples for the power of chemoselective probes are (i) *o*-diacetylbenzene (DAB, **26**), which can discriminate amines in amino acids from primary amines, used in HTS of amino acid decarboxylases,^32,33^ (ii) β-aryloxy fluorescein aldehyde **38** reported by Leslie *et al*.,^34^ which undergoes accelerated β-elimination by secondary amines^34^ and is valuable for detecting reactions that convert primary to secondary amines (*e.g*., methyltransferases).

Manipulating the reaction conditions can also be used to change selectivity of an assay by changing the reactivity of the target. For example, fluorescamine **18**, a probe for amines, can under acidic conditions be used for the selective detection of aromatic amines in the presence of aliphatic amines because the p*K*_a_ values of these types of amines are sufficiently different.^35^

Because the selectivity profile of most reported assays is not extensively characterized (even for the small number of commercially available probes), it is often unclear to protein engineers which reagent might be the most suitable to detect the product of interest and many need to be tested. Additionally, many studies on assay development add the analyte in large excess over the detection probe. While this approach is advantageous to show the optical performance of the assay, it does not guarantee its practicability for screenings. It would add great value if research groups developing new assays characterized the selectivity of a new probe in commonly used reaction media toward various derivatives of the same substance or reactivity class (*e.g*., nucleophiles, electrophiles, oxidants) and (cellular) interfering molecules.

The goal of this review is to present protein engineers, biochemists, and chemists with an overview of available options for the detection of compounds of interest. For synthetic chemists, we provide a summary of known starting points for the development of even more selective probes.

## 5 Principles for Designing Functional Group Probes

**Scheme 1:**
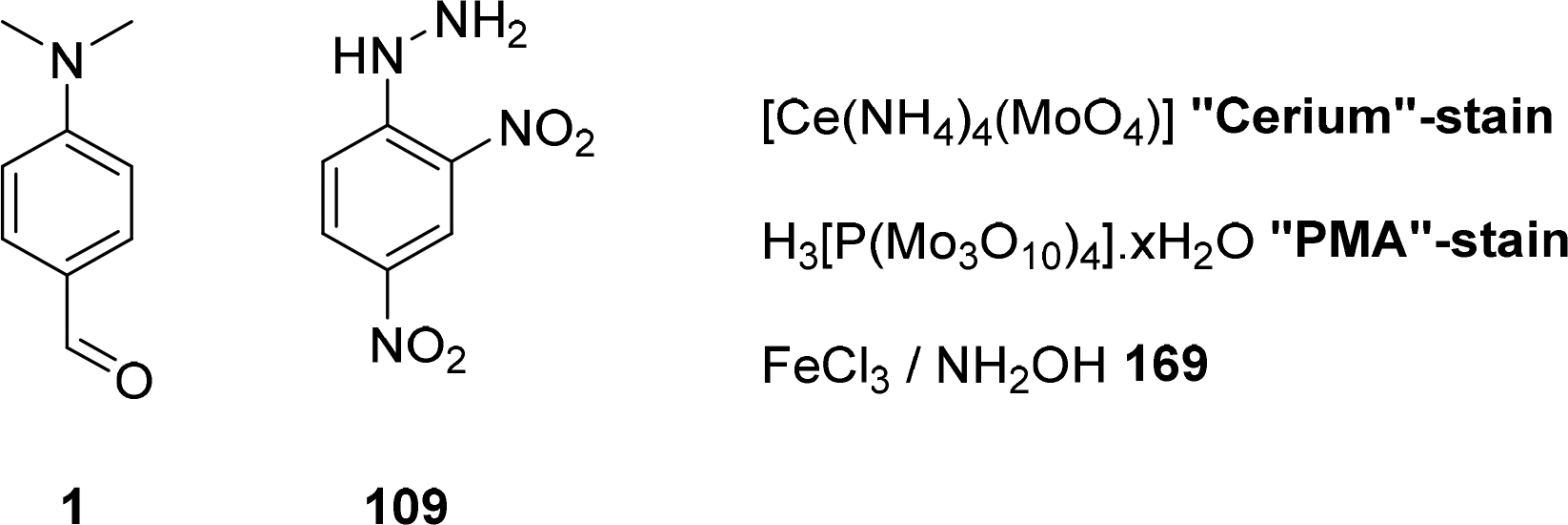
Representative Reagents Typically Used in Thin-Layer Chromatography (TLC) Staining Solutions

The detection of organic functional groups is widespread in chemistry. Thin-layer chromatography,^36^ which is taught in beginner chemistry laboratory courses, often uses chemoselective, chromogenic stains based on reactions with functional groups. Historically, the discovery and development of staining agents improved the characterization of new compounds *via* derivatization and enabled reliable and fast reaction analytics. Many selective reagents enjoyed great popularity, including Ehrlich’s reagent **1**,^37^ 2,4-dinitrophenylhydrazine (DNPH) **109**,^38^ phosphomolybdic acid,^39^ Fe^(III)^Cl_3_/hydroxylamine,^40^ Hanessian’s cerium stain,^41^ Simon’s reagent,^42^ among others, and are still used for illicit drug screenings (Scheme 1).

Only a few of these classic probes have been applied in protein engineering, so far, likely because of the drawbacks that many of these agents entail: they are largely incompatible with biological systems, have poor selectivity or sensitivity, and inadequate optical properties. Nevertheless, some emerged as the structural or conceptual basis for many probes that followed.

Designing chemoselective probes has several requirements to enable their general application:^14^ (i) Most importantly, the probe must react selectively with the target analyte in the presence of substrates and other background (from cells or the medium). Chemoselectivity has to be sufficiently high to promote the preferential reaction with the desired analyte even in the presence of molecules with similar functional groups. (ii) Probes should react fast under actual screening conditions. A HTS assay should not be limited in throughput by the reaction rate of the probe. (iii) If cells need to be kept alive (*e.g*., for cofactor recycling), probes have to be nontoxic.

Two options commonly serve as the starting point for initial development: (i) the discovery of a suitable, highly selective chromo- or fluorogenic reaction and subsequent refinement of the chemical probe’s spectral properties, or (ii) the incorporation of a chemical sensing mechanism into an already established chromophore which causes a desired optical read-out.

Detecting targets at low concentration with a short lifetime brings particularly stringent requirements for selectivity and reactivity. Probes that meet these requirements can enable special methods, such as single-cell droplet assays, or HTS campaigns that search for the proverbial “needle in the haystack.”

In addition to defining the chemical properties of a probe, the choice of the chromogenic platform is also essential. Knowledge about the genesis of and control over the optical signal is necessary for the rational design of good probes. Spectroscopic properties of organic molecules at visible and near-visible wavelengths are closely associated with their electronic (delocalized) structures.^43^ Extension of a conjugated π–electron system upon reaction is a common element in designing chromogenic probes, which generally leads to a bathochromic shift in the absorption maximum. It does not simultaneously guarantee the emission of a fluorescent signal, which is only observed for a small fraction of absorbers due to the action of prominent quenching mechanisms, such as internal conversion (IC) or intersystem crossing (ISC).^44^ Fluorescence is favored over absorption as the signal-generating mode due to a better signal-to-noise ratio and thus lower detection limits. Generally, it is easier to see a strong signal against a weak background than to look for weakening of a strong background.

Most turn-on fluorescent probes are based on a change of the electronic structure of the chromogenic dye,^45,46^ triggered by changes to the size of a delocalized electron system (*e.g*., degree of conjugation of π–electron systems) or to the distribution of electrons in the system (by the introduction, manipulation, or removal of electron-donating or withdrawing groups). The resulting changes in absorption coefficients, quantum yields, and/or Stokes shifts can be measured and, if chemoselective, related to changes of the analytical target. Other mechanisms of fluorescent assays to generate a signal include: suppression of a photoinduced electron transfer (PET),^47^ enabling a fluorescence resonance energy transfer (FRET) between donor and acceptor fluorophores,^48^ modulating an intramolecular charge transfer (ICT),^49^ or enabling an excited-state intramolecular proton transfer (ESIPT).^50^ Recent developments, such as aggregation-induced emission (AIE),^51^ twisted intramolecular charge transfer (TICT),^52^ electronic energy transfer (EET),^53^ or C=N isomerization, have not yet found widespread application in the design of reaction-based turn-on probes.

There are excellent reviews on general design principles of fluorescent chemosensors, with a focus on chemoselective bioimaging of endogenous metabolites,^14^ fluorescent probes in redox biology applications,^54^ imaging of reactive oxygen, nitrogen, or sulfur species (ROS, NOS, RSS),^55,56^ anions and cations,^57^ screening for defined enzymatic functionalities,^13,58^ or the high-throughput aspect of selected methods.^7^ Here, we will elaborate on reaction-based probes in the context of protein engineering. We outline the rational design of most known functional-group probes; some of them have not (yet) been used in protein engineering.

## 6 Chromo- and Fluorogenic Probes for the Detection of Amines and Amino Acids

Amines are a highly valuable class of compounds used in the chemical, pharmaceutical, and agrochemical industries. They are frequently used as intermediates for a vast number of bioactive compounds, especially in their optically pure form.^59^ Efficient methods for their enantioselective preparation typically require sophisticated catalyst design or the use of transition metals.^60,61^ Hence, chemists have turned their attention to enzymes, which have since been established as an excellent approach for the preparation and use of amines as building blocks.^366^

**Figure 3:**
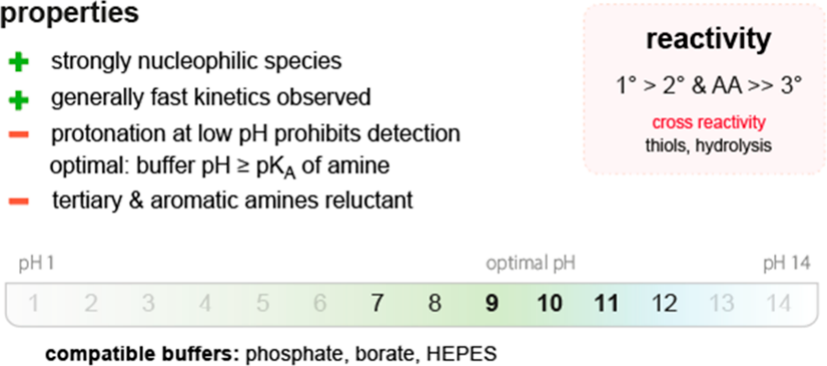
(top) Summary of general properties of amines, reactivity trends toward amine-selective probes, and expected cross-reactivity. (bottom) Compatible pH range and buffers in reported amine-selective assays. Bold numbers indicate the most commonly used pH values.

To assess the progress of transformations that move amino groups intermolecularly, a probe with a significant difference in reactivity between substrate and product is required. Large differences often result from an increase of substitution and steric bulk. Primary amines and ammonia have the highest reactivity and thus can be efficiently discriminated from secondary amines and most amino acids. Tertiary amines are mostly unreactive toward conjugation due to the decreased steric accessibility.^667^

Amidases have the biggest potential to benefit from these chemical probes because they release amino compounds as products. The strong nucleophilicity of this functional group creates possibilities for electrophilic reactions in addition or substitution reactions. Cross-reactivity in biocatalytic studies can be expected with other nucleophiles present (*e.g*., thiols, cyanides, and, if not targeted themselves, amino acids). The rate of those side reactions depends on the actual substrate, the conjugation partner, pH, temperature, and buffer composition.

Amines are generally mildly basic, and reactions with aliphatic amines are thus confined to a pH range of approximately 8–10 to avoid deactivation by protonation. Aromatic amines can be selectively targeted in neutral or slightly acidic regimes because their p*K*_a_ values are generally lower than those of aliphatic analogues (4.5 *vs* 9).^668^

Amine-containing buffers (*e.g*., Tris; tris(hydroxymethyl)aminomethane) must be avoided when using amine-reactive probes because the rate of the reaction with the buffer would greatly exceed that with amino groups in the product (Figure 3).

**Scheme 2:**
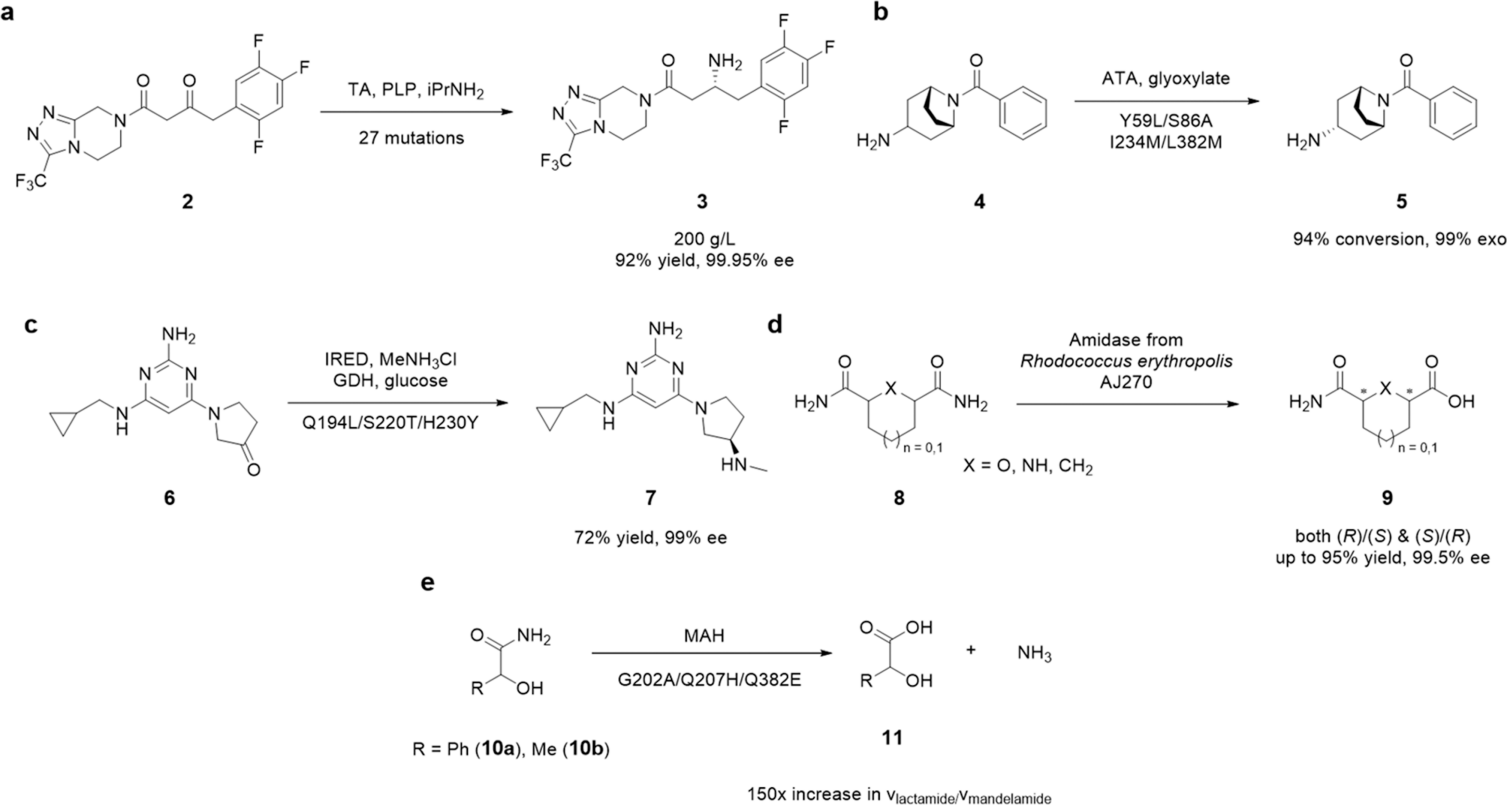
Representative Selection of Enzymatic Transformations for the Production of Amine-Containing Products

Commonly used enzymes to yield amine-containing compounds, include amidases,^62^ lipases,^63,64^ transaminases (TA),^65^ reductive aminases (RA),^66,67^ imine reductases (IRED),^68^ monoamine oxidases (MAO),^69^ and lyases.^70^ Several studies have described the potential of these catalysts (Scheme 2). In a seminal study, Savile *et al*. applied substrate walking, modeling, and a mutational approach to establish a highly potent transaminase for the synthesis of the antidiabetic compound sitagliptin **3** (Scheme 2a).^71^ Their best variant included 27 mutations and converted the ketone **2** to the chiral amine **3** at 200 g/L substrate loading in 50% DMSO, with a 92% assay yield and full enantioselectivity. Challenged with the sterically substantially hindered bicyclic amine **5**, Weiß *et al*. applied protein engineering for the optimization of a fold class I ((*S*)-selective amine transaminase).^72^ Saturation mutagenesis and two rounds of directed evolution facilitated the kinetic resolution of the racemic substrate **4** in excellent yield and full stereocontrol (Scheme 2b). Interestingly, reactions performed with the prochiral ketone as starting material determined the mutant to be unsuitable for direct asymmetric synthesis. Machine-directed evolution was applied by Novartis researchers as a potent enzyme engineering strategy by employing a moderately stereoselective imine reductase as the model system.^73^ One screening cycle already yielded a library of highly active mutants with a dramatically shifted activity distribution compared to that of the traditional directed evolution methodology. Using their most selective and active mutant, the authors synthesized the H4 receptor antagonist ZPL389 **7** in 72% yield with >99% *ee* (Scheme 2c). Smart rational engineering of amidase AJ270 from *Rhodococcus erythropolis* by the group of Wang enabled the desymmetrization of heterocyclic *meso*-dicarboxamides by generating only 10 variants.^74^ Using their biocatalytic platform, they enabled access to both antipodes of *O*,*N*-heterocyclic and carbocyclic dicarboxamides **9** in excellent yields and full stereocontrol (Scheme 2d). Another protein engineering example for amidases was delivered by the group of McLeish.^75^ A comparison of several members of the enzyme family inferred that residues involved in substrate recognition were not conserved. Substitution of position G202, crucial to substrate binding, to slightly bulkier alanine or valine residues, prompted a dramatic decrease in the *k*_cat_/*K*_M_ value of mandelamide **10a** with concomitant increase in lactamide **10b** activity, effectively transforming the biocatalyst into an aliphatic amidase (Scheme 2e). Given the scope of this review, however, only a subset of the stated biocatalysts can be efficiently targeted using the presented methodology.

### 6.1 Nucleophilic Aromatic Substitution (S_NAr_)

**Figure 4:**
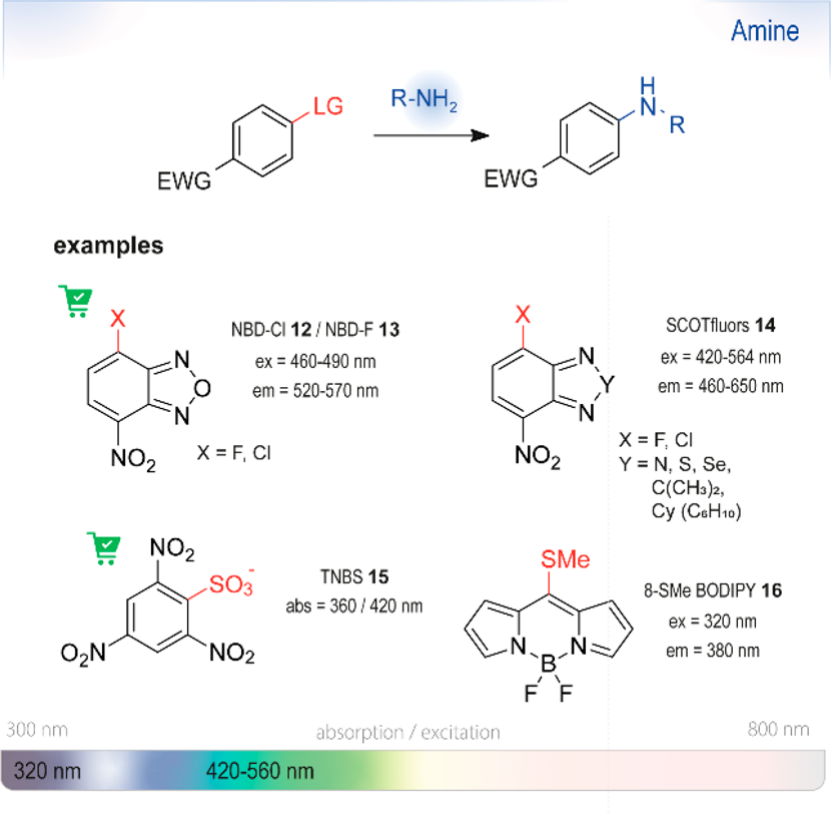
Summary of chromo- or fluorogenic turn-on probes for the selective detection of amines based on nucleophilic aromatic substitution (S_NAr_) with aromatic molecules containing electron-withdrawing groups (EWG). Abs: wavelength typically used for UV absorbance measurements. Ex or em: wavelengths typically used for excitation and emission in fluorescence measurements. The wavelengths of probes in this section are shown on the strip at the bottom representing the visible spectrum.

S_NAr_ reactions of *para*-substituted electron-poor aromatic systems enable the emergence of an optical signal (Figure 4). The change in absorbance (or fluorescence) is commonly explained by the formation of electronic “push-pull” structures: The incorporation of electron-donating groups (EDGs) by reaction with the amine and the pre-existing electron-withdrawing groups (EWGs) in the *para* position enable ICT processes.^76^

The most common leaving groups are halides (Cl^–^, F^–^) and sulfonates (SO_3_^–^); fluorides are most reactive. Historically, this concept was used by Sanger for the detection of amines in insulin (Sanger’s reagent: 2,4-dinitrofluorobenzene, DNFB).^78^ Nitrated aryl systems have high absorption coefficients and led to the development of nitrobenzoxadiazole (NBD) reagents. Ghosh *et al*. were the first to report the use of NBD-Cl **12** as a fluorogenic nucleofuge and noticed different optical signatures depending on the reaction partner. Whereas primary and secondary aliphatic amines formed highly fluorescent products, reactions with *O*, *S*, and *N*-Ar nucleophiles only gave weak fluorescence. Tertiary amines were found to be unreactive.^79^ Watabe *et al*. switched to the fluoro-derivative of NBD, which is 50–100-fold more reactive than NBD-Cl, for the detection of hydroxyproline and proline in blood plasma.^80^ Recent studies used NBD-Cl **12** for the detection of l-ornithine^81^ and NBD-F **13** for the derivatization of Alendronate.^82^ In a recent study, Annenkov *et al*. showed that NBD-Cl can be a viable derivatizing agent even for tertiary amines at room temperature, albeit in dioxane.^77^ The substituted products are believed to undergo subsequent Hofmann elimination or substitution by the expelled chloride ion and form a mixture of highly fluorescent NBD adducts. Application in aqueous systems was not investigated.

**Figure 5:**
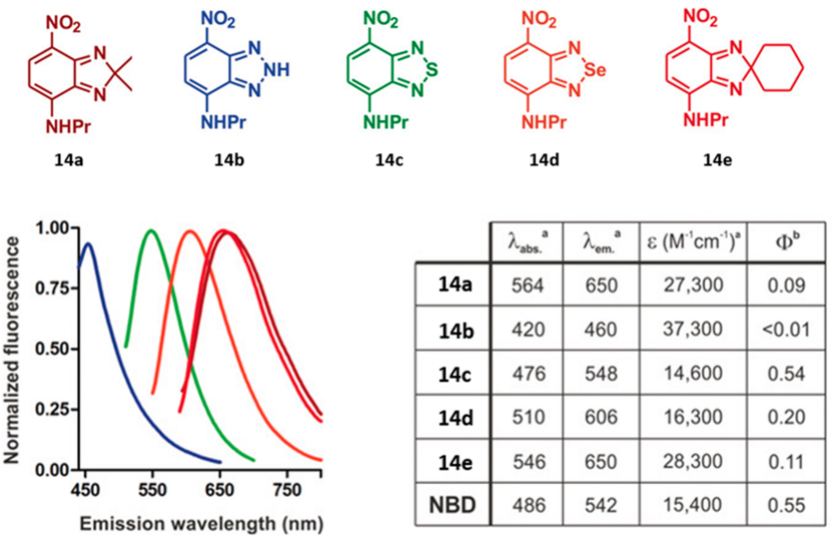
Structures and spectral properties of SCOTfluors **14a**–**e**. ^a^Values determined in EtOH. *^b^*QY (quantum yield) in dioxane using acridine orange, fluorescein, rhodamine 101, and Cy5 as standards. Reproduced with permission from ref (83). Copyright 2019 Wiley-VCH under Creative Commons Attribution 4.0 International License https://creativecommons.org/licenses/by/4.0/.

NBD-fluorophores, with excitation maxima between 460 and 490 nm, are often incompatible with other green-fluorescent reporters, such as the broadly used green fluorescent protein (GFP) with an excitation maximum at 488 nm. To circumvent this overlap and thus improve their usefulness in *in vivo* studies, Benson *et al*. substituted the oxygen in the ring with a quaternary carbon (SCOTfluors **14a**–**14e**, Figure 5). These probes showed tunable emissions in the near-infrared (NIR) spectrum under physiological conditions and were applied for *in vivo* cell imaging.^83^

**Figure 6:**
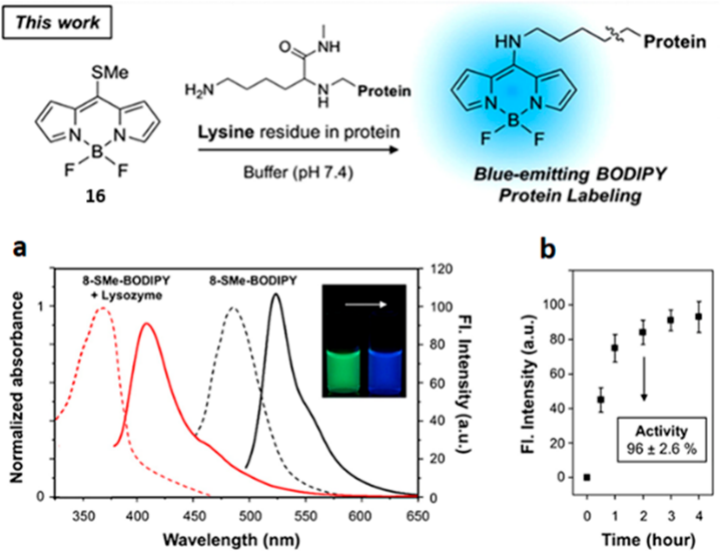
Labeling scheme of lysine residues in protein with **16**. (a) Normalized absorbance spectra (dashed-lines) and fluorescence emission spectra (solid-lines) of **16** (1 μM) and lysozyme labeled with **16** (5 μg/mL lysozyme, 2 h incubation at 37 °C), measured in HEPES buffer (10 mM, pH 7.4, 0.1% dimethyl sulfoxide). The emission spectra were obtained with excitation at 485 and 375 nm, respectively. The inset photo was taken under UV light (365 nm). (b) A plot of time-dependent (0–4 h) fluorescence intensity at 409 nm. The excitation wavelength was 375 nm. Inset box: lysozyme activity calculated using an assay kit at 2 h incubation point. Means and standard deviations were calculated from triplicate measurements. Reproduced with permission from ref (90). Copyright 2017 Korean Chemical Society; Wiley-VCH.

Another historical example of an S_NAr_ reagent is 2,4,6-trinitrobenzenesulfonic acid (TNBS **15**), which was first described by Okuyama and Satake in 1960.^84^ Formation of aniline derivatives upon reactions with aliphatic amines can be traced by observing intense orange bands. Even though it is explosive, TNBS has still found broad use in protein modification of terminal amino acids because it selectively reacts with primary amines.^85−88^ Recently, Olivero *et al*. used the reagent in combination with 3-(4-carboxybenzoyl)quinoline-2-carboxaldehyde (CBQCA, **24**) (*cf*. section 6.3) for the quantification of proteins bound to nanoparticles.^89^ With the emergence of new fluorogenic scaffolds, Kim *et al*. incorporated the amino-responsive function into the BODIPY fluorophore.^90^ Upon reaction with lysine residues, the excitation wavelength of the BODIPY derivative **16** shifts from 525 to 409 nm and could be used for fluorescent labeling under mild conditions (37 °C, 2 h) (Figure 6).

### 6.2 Michael Addition (Type) Reactions

**Figure 7:**
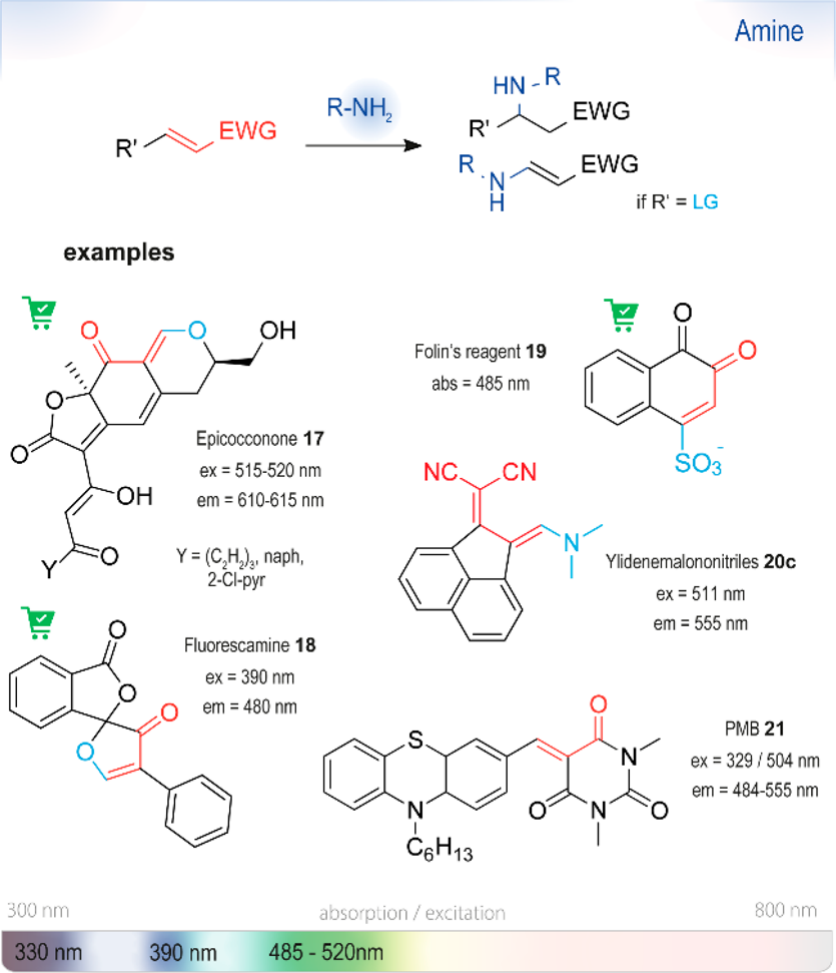
Summary of chromo- or fluorogenic turn-on probes for the selective detection of amines based on Michael addition type reactions. Abs: wavelength typically used for UV absorbance measurements. Ex or em: wavelengths typically used for excitation and emission in fluorescence measurements. The wavelengths of probes in this section are shown on the strip at the bottom representing the visible spectrum.

As functional group probes, Michael acceptors make use of the nucleophilicity of amines in neutral and basic aqueous solutions. Most probes of this type (Figure 7) undergo a Michael addition and subsequent β-elimination: upon 1,4-conjugate addition of the amine, the leaving group is expelled, forming a stable enamine.

**Figure 8:**
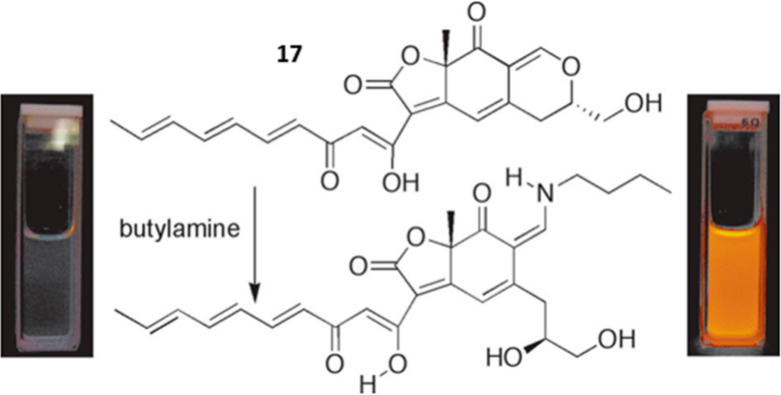
Epicocconone **17** is the active ingredient in Deep Purple Total Protein Stain and is responsible for the apparent noncovalent staining of proteins in polyacrylamide gels and electroblots. The reaction of **17** with amines has shown that **17** reacts reversibly with primary amines to produce a highly fluorescent enamine that is readily hydrolyzed by base or strong acid. Such conditions are used in postelectrophoretic analysis, such as peptide mass fingerprinting or Edman degradation. Reproduced with permission from ref (92). Copyright 2005 American Chemical Society.

A prominent example is epicocconone **17** (Figure 8), a yellow dye isolated from the fungus *Epicoccum nigrum*.^91^ Elucidation of the structure enabled its application for the detection and staining of primary amines.^92^ The conjugate that forms upon reaction with lysine has a high molar absorptivity (10^4^ M^–1^cm^–1^), strong orange to red fluorescence (λ_em_ = 610 nm), and a large Stokes shift (100 nm). The stability of the enamine depends on the pH and is highest at 2.4.^92^ The derivatization is reversible at neutral pH, which enabled the analysis of proteins by staining gels after electrophoresis.^93^ Substitution of the heptatriene chain in **17**, especially with a *p*-chloropyridyl functionality, increases fluorescence yields without impacting the performance in staining.^94^

Fluorescamine **18**, an intrinsically nonfluorescent reagent, is another commonly used probe for the selective derivatization of primary amines.^25^ Although secondary amines and alcohols react with the probe, they do not form fluorescent products. Furthermore, the reaction with alcohols is reversible and thus tolerated in amine screenings.^95^ Unfortunately, due to the poor hydrolytic stability of the reagent, an excess must always be used to achieve reliable derivatization,^96,97^ but because the hydrolyzed product of **18** is not fluorescent, this assay enjoys high popularity. The maximum in reactivity and fluorescence appears at pH 9, but assays performed at pH 6–8 still provide acceptable results with aliphatic amines.

Acetic acid can be used as a reaction medium to quantify aromatic amines, as shown by Rinde and Troll.^35^ Aromatic amines remain unprotonated even in this acidic milieu, making selective detection in the presence of aliphatic amines possible. Two recent studies applied this reagent in HTS: Murugayah *et al*. used **18** to detect activity of *N*-acyl-l-homoserine lactone (AHL) acylase,^98^ and Ashby *et al*. used it to detect the interaction between proteins and nanoparticles (changes in the structure of the studied proteins caused a change in fluorescence, which made conformational changes upon interactions with nanoparticles detectable).^99^

1,2-Naphthoquinone-4-sulfonate (NQS), also called Folin’s reagent **19**, is one of the most commonly utilized chromogenic reagents for the determination of illicit drugs and pharmaceuticals containing aliphatic or aromatic amines.^100^ The reaction of NQS with amines forms an orange dye, which is stable over a broad pH range (2–11). The rate of the reaction is independent of the pH but impacted by the amine substrate.^101^ Most assays are complete in less than 20 min (at pH 9–10).^102^ Besides amines, it is also possible to detect sulfamides and tetrazoles.

**Figure 9:**
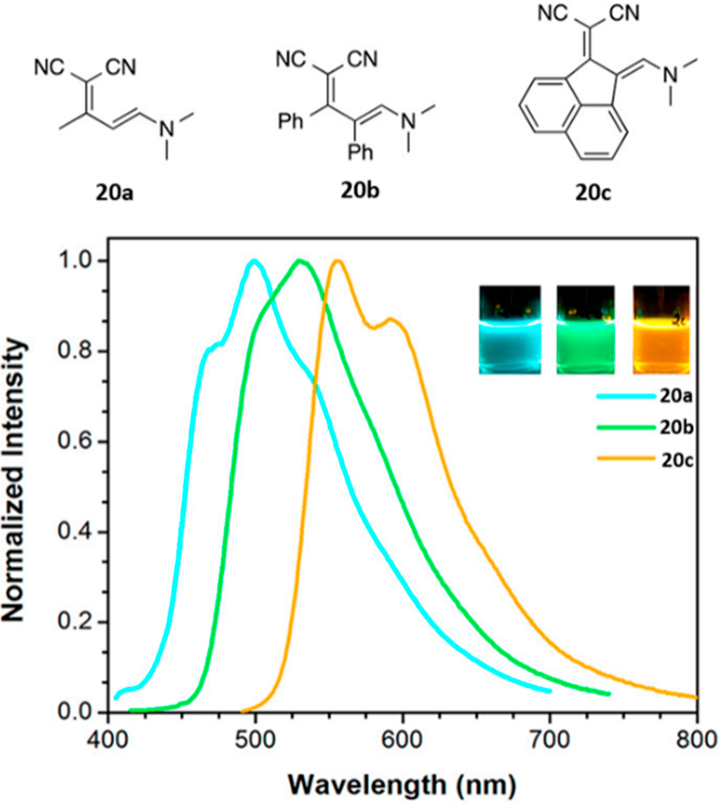
Normalized emission spectra of ylidenemalononitrile enamines **20a**–**c** in CH_2_Cl_2_ at room temperature. Insets show photographs of 1 mM solutions of **20a–c** in CH_2_Cl_2_ irradiated at 365 nm. Reproduced with permission from ref (103). Copyright 2014 American Chemical Society.

Ylidenemalononitriles (compounds **20a**–**c**) are another useful group of compounds for amine assays, as shown by Longstreet *et al*.^103^ They are weakly fluorescent, but upon reaction with (some) primary amines form cyclic amidines with a 900-fold increase in emission intensity. Several derivatives were synthesized (Figure 9, **20a**–**20c**), spanning the spectral excitation (λ_ex_ = 390–510 nm) and emission windows (λ_em_ = 500–555 nm). Their use was demonstrated in the derivatization of a human transferrin glycoprotein at pH 7, albeit in 50% DMSO.

A recent example of a red-emitting probe, PMB **21**, was reported by Sathiskumar and Easwaramoorthi.^104^ In this compound, phenothiazine and 1,3-dimethylbarbituric acid are connected *via* a methylene bridge. Fluorogenic properties of the probe are observed upon reaction with several primary aliphatic and electron-rich aniline systems. The described mechanism suggests that addition of the amine leads to the cleavage of the barbituric acid moiety and the formation of phenothiazine-conjugated imines. Higher substituted amines and deactivated aromatic rings did not give any signal. The strong dependence of the fluorescence emission on the reaction partner can be explained by large differences in ICT processes in the products. Compound **21** was used for the detection of 3-amino-l-tyrosine in buffered medium.

### 6.3 Isoindole Formation

**Figure 10:**
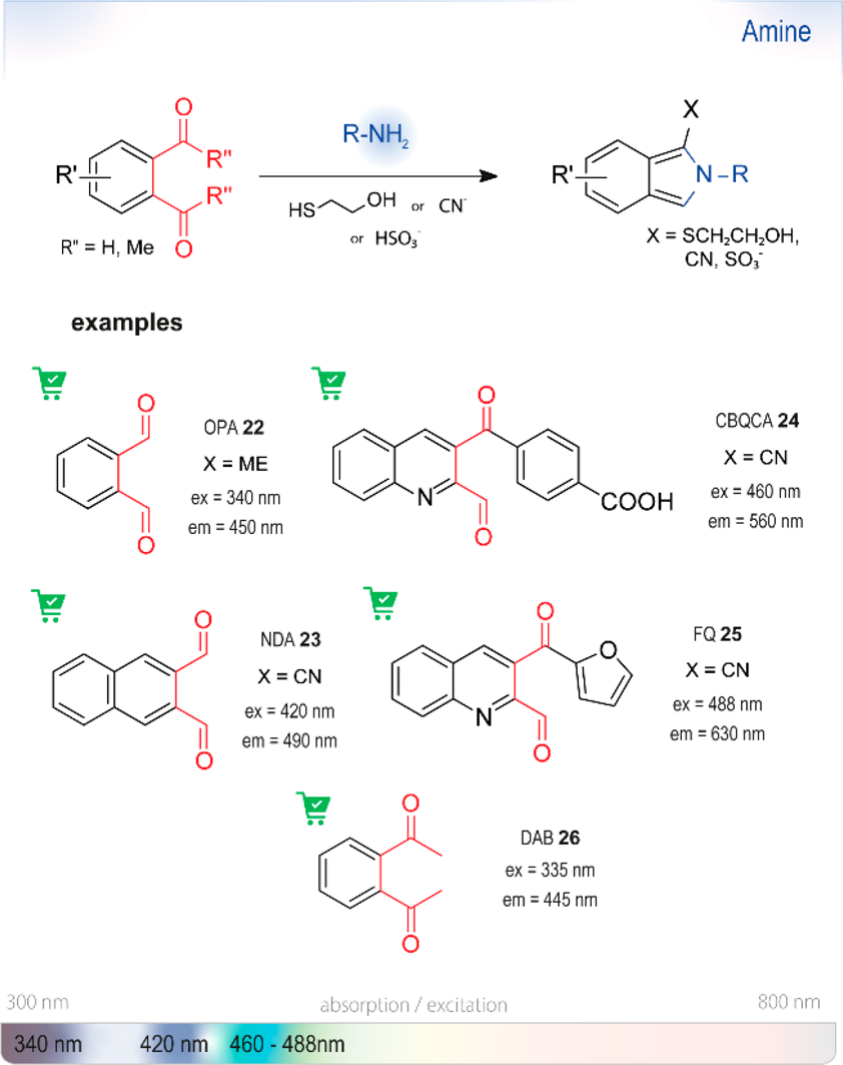
Summary of chromo- or fluorogenic turn-on probes for the selective detection of amines based on isoindole formation with *o*-phthalaldehyde derivatives and *o*-diacetylbenzene. Ex or em: wavelengths typically used for excitation and emission in fluorescence measurements. The wavelengths of probes in this section are shown on the strip at the bottom representing the visible spectrum.

Another approach for the detection of amines is entrapping them in an isoindole structure (Figure 10). In a double-condensation reaction, *o*-phthalaldehydes, such as compound **22**, and amines form highly reactive *o*-quinoid structures.^24,30^ Initial attempts to form isoindolines used reducing agents, *e.g*., 2-mercaptoethanol (ME) or bisulfite,^105^ to “stabilize” these products. Analysis of the highly fluorescent conjugates, however, revealed that 1-substituted isoindoles are formed, their emission depending on the structures of the parent dialdehyde and trapping nucleophiles. Practical trapping agents include aliphatic thiols (*e.g.* ME, cysteine) sulfites, or cyanide. In consequence, this multicomponent system can also be used for the detection of thiols or cyanides. Especially the electron-withdrawing CN-substituent is believed to stabilize the resulting conjugates.^106^ The addition sequence of the reagents is crucial for the intensity of the fluorescence emission.^24^ For instance, addition of the thiol before the amine was beneficial (but not necessary) for strong fluorescence. Amino acid conjugates gave a strong fluorescent signal in the range of pH 7–12. The reactions, however, were typically performed above pH 8 to enable a broad spectrum of amines to be detected. With thiols as nucleophiles, a substantial amount of thiolate (p*K*_a_ ∼ 11) is required.^30^ Generally, sterically accessible amines react fastest with the isoindole probes, reducing the derivatization time to several minutes for simple primary amines.^111,112^ The fluorescence emission, however, depends on multiple factors, including pH, the trapping nucleophile, the detectable amine, and even temperature. Ammonia generates significant background emission: its conjugates are 100–1000-fold more emissive than those of amines.^107^

Enlarging the π–electron system, *e.g*., with naphthalene as in NDA **23**, or analogues,^108,109^ is generally accompanied by a large red-shift and increase in sensitivity. A systematic study by de Montigny *et al*.^106^ revealed a strong dependence of the Stokes shift and the quantum yield of the conjugates on the trapping nucleophile. While reactions with **22** were ideally performed with ME, **23** was shown to be practically nonfluorescent with this thiol donor. Cyanide can be used with both dialdehydes, but premixed solutions of **23** with cyanide were prone to decomposition; they had to be prepared separately. Reactions with amino acids were shown to proceed fastest between pH 8.5 and 10, with a maximum at ∼9.5 (**23**/CN detecting alanine).

CBQCA **24**(89,669) and 5-furoylquinoline-3-carboxaldehyde (FQ, **25**)^670−672^ are additional analogues for the same class. FQ is several orders of magnitude more sensitive than its parent compound OPA **22**, or fluorescamine **18**, in the quantification of bovine serum albumin (BSA), but long incubation periods are necessary (90 min for FQ, compared to 30 min for **22)**. Compound **24** has been used to quantify proteins bound to nanoparticles in HTS by the group of De Crescenzo.^89^ Hapuarachchi and Aspinwall used a combination of **22** and SAMSA-F, a thiol-bearing fluorescein derivative for the labeling of primary amines and amino acids.^110^ Although their method required the subsequent separation of the tagged amines from the unreacted probe by capillary electrophoresis, it showed how mechanistic understanding can be used in design. Recently, Medici *et al*. showed that the ketone analogue of **22**, *o*-diacetylbenzene (DAB, **26**), can efficiently differentiate between primary amines and their corresponding amino acids, thus enabling HTS of amino acid decarboxylases.^32^ As with **22**, ME was used as the trapping nucleophile. Given that the resulting isoindole does not condense to a fully aromatic product, a study by Choi *et al*. attempted to elucidate the structure of the actual fluorophore.^33^

**Scheme 3:**
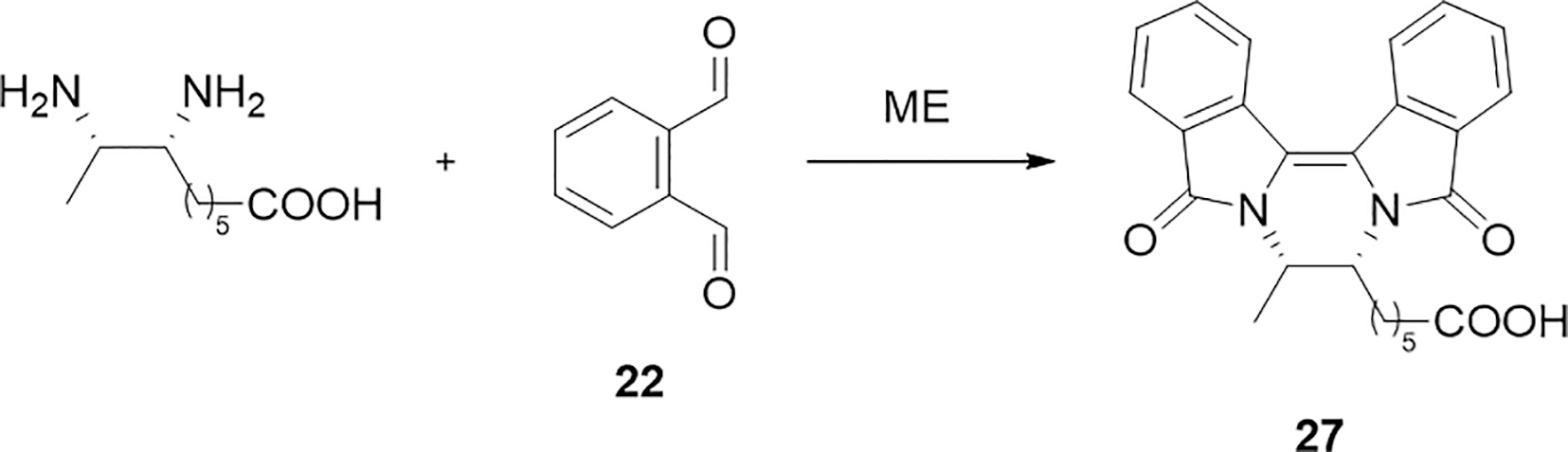
Reaction of **22** with Vicinal Diamine Resulting in the Formation of Pyrazino-diisoindole-dione **27**

The presented probes have been used for detection of amines, amino acids, and even ammonia as nitrogen sources in cellular systems. Examples using compound **22** include screening for activity of: (i) catalase, measured *via* the H_2_O_2_ triggered depletion of GSH,^113^ (ii) nitrilase, by detecting ammonia,^114^ (iii) aminotransferase,^115^ by ME-catalyzed formation of fluorescent pyrazino-diisoindole-diones **27** (Scheme 3). Unexpectedly, ME was not incorporated into the product but was nevertheless needed as a catalyst. The fluorophore exhibited a blue-shifted excitation maximum at 410 nm, only observed with vicinal diamines; other diamines did not show measurable fluorescence upon excitation at this wavelength.

### 6.4 Amide Formation *via* Activated Ester Cleavage

**Figure 11:**
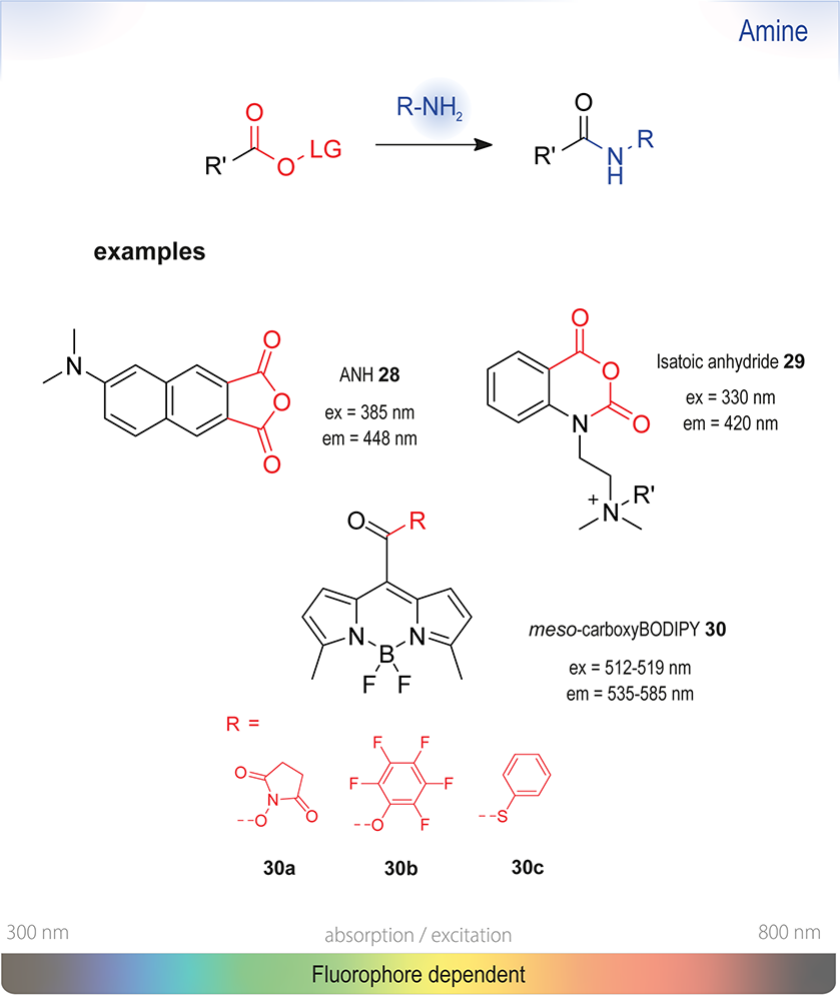
Summary of chromo- or fluorogenic turn-on probes for the selective detection of amines based on amide formation using activated esters. Ex or em: wavelengths typically used for excitation and emission in fluorescence measurements. The wavelengths of probes in this section are shown on the strip at the bottom representing the visible spectrum.

Activated esters are the synthetic chemist’s mimic of nature’s highly reactive thioester or acyl phosphates used for selective amidations. In recent years, many probes were developed by incorporating metastable leaving groups, such as mixed anhydrides, NHS esters (*N*-hydroxysuccinimide),^116^ pentafluorophenyl (PFP)-,^117^ or thiophenols,^118^ into established fluorescent molecules (*e.g*., fluorescein,^119^ coumarin,^120^ BODIPY,^121^*etc*.; Figure 11).^122^ The majority of such designs modify the molecular structure of the dye in a region distant from the fluorophore core, which typically does not significantly change the spectral properties of the dye and thus requires the separation of the conjugate from any unreacted probe after the reaction.^123−126^ Conversely, the incorporation of the reactive handle directly into the fluorophore modulates the fluorescent emission and led to the development of reactive turn-on probes.

Typically, the turn-on effect is achieved by liberating fluorogenic carboxy or hydroxy moieties from their initial state, when they are trapped as reactive anhydrides, carbonates, or labile esters. Two examples for ratiometric turn-on probes: (i) 6-(dimethylamino)naphtho[2,3-*c*]furan-1,3-dione (ANH, **28**)^127^ and derivatives thereof^128^ as well as isatoic anhydride analogues.^129,130^ ANH has great potential as an environment- and solvent-sensitive turn-on fluorophore, but it is only weakly fluorescent in water. An increase in fluorescence was only observed upon conjugation to apolar protein domains. To tackle the challenge of poor aqueous solubility of a majority of apolar turn-on-based (NHS-) probes, the group of Ogle derivatized fluorogenic isatoic anhydride **29** with a quaternary ammonium handle.^130^ This permanently charged group substantially increased the solubility in water, no longer requiring cosolvents for conjugation, *e.g*., with BSA. They furthermore demonstrated the efficiency of their probe using a rapid-injection NMR experiment: a 10-fold excess of *n*-butylamine completely reacted with their probe in less than 5 s.

All reagents of this type, however, were unstable toward hydrolysis, with half-lives of 16 h (pH 7) and 2 h (pH 8.4). This reactivity is generally expected of any activated ester, as hydrolysis under basic conditions always competes with the desired reaction.

**Figure 12:**
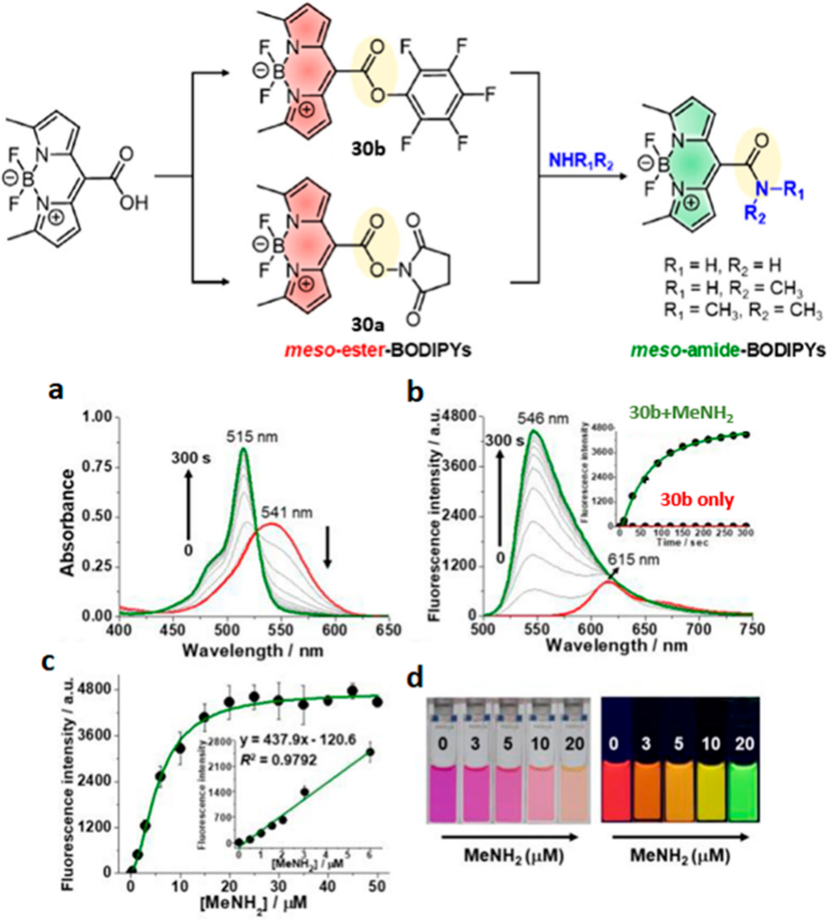
(top) Synthesis of fluorogenic *meso*-active-ester-BODIPYs **30a**/**30b** and their conversion to fluorescent *meso*-amide-BODIPY products upon reaction with amines. (bottom) Absorption (a) and emission (b) spectra of **30b** (10 μM) upon treatment with MeNH_2_ (20 μM) in CH_3_CN as a function of time (0–5 min) at 25 °C. λ_ex_ = 470 nm. (inset) Relative fluorescence intensity at 546 nm as a function of incubation time, in the absence (red) and the presence (green) of MeNH_2_. (c) Relative fluorescence intensity at 546 nm as a function of [MeNH_2_] (0–50 μM). (inset) Calibration curve of fluorescence intensity at 546 nm *vs* [MeNH_2_] (0–6 μM). (d) Photographs of **30b** (20 μM) upon addition of MeNH_2_ (left to right: 0, 3, 5, 10, 20 μM) under ambient light (left) and 365 nm UV irradiation (right). Incubation time = 5 min. Reproduced with permission from ref (131). Copyright 2020 American Chemical Society.

Similar to the well-established methods for amine conjugation using cleavable moieties, the group of Kim adapted their *meso*-carboxyBODIPY **30** probe and achieved a remarkable turn-on effect upon reaction of its activated acid,^131^ initially with the NHS- **30a** and PFP **30b** esters (Figure 12) and later also with thioester **30c**.^132^ Using their PFP derivative **30b**, they showed a 3000-fold increase in fluorescence upon reaction with MeNH_2_ under physiological conditions in less than 5 min. A similar rate was observed for simple primary aliphatic mono- and diamines. An increase in steric bulk caused a substantial decrease in reactivity and required reaction times of 60 min to reach similar turn-on factors (*e.g*., 900-fold with cyclohexylamine). Tertiary and aromatic amines were unreactive. Compound **30a** produced a strong fluorescent signal upon conjugation to lysine or arginine after 5 min under physiological conditions. The other canonical amino acids only gave a weak signal, likely due to a steric effect. Compound **30b** was not tested with amino acids.

**Figure 13:**
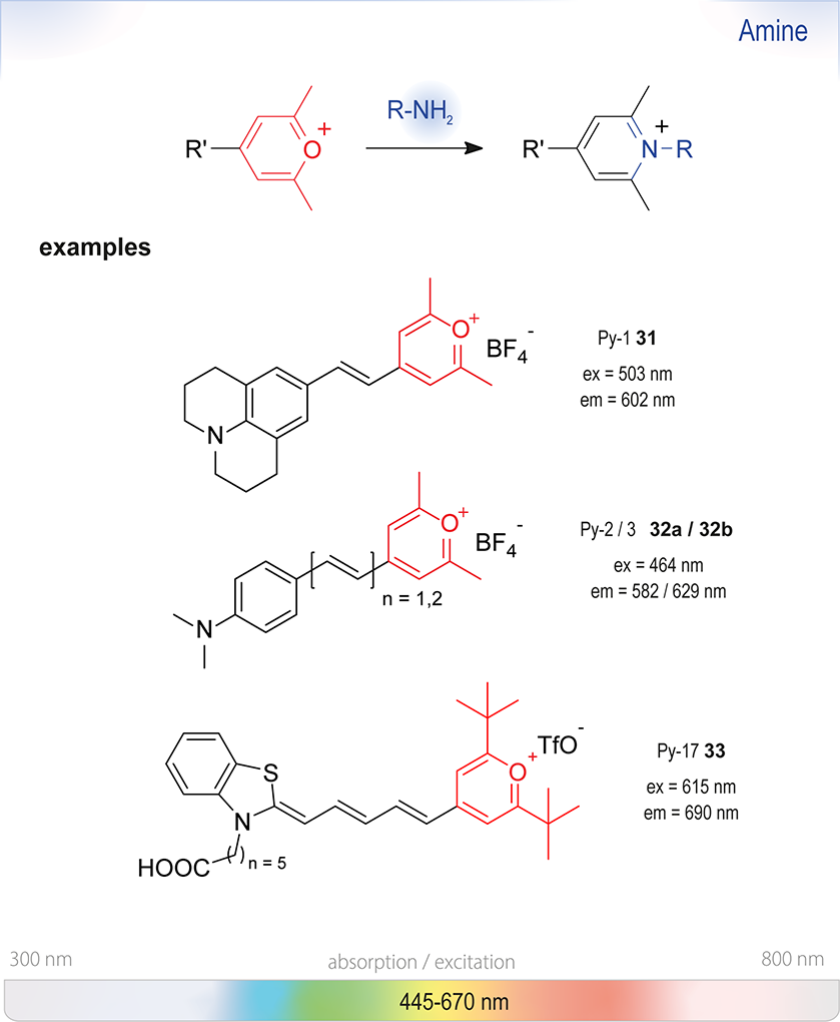
Summary of chromo- or fluorogenic turn-on probes for the selective detection of amines based on pyridinium salt formation with pyrylium salts. Ex or em: wavelengths typically used for excitation and emission in fluorescence measurements. The wavelengths of probes in this section are shown on the strip at the bottom representing the visible spectrum.

**Figure 14:**
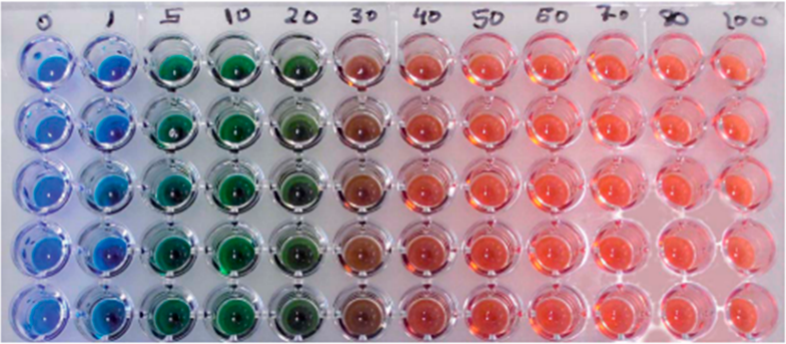
Image of a Py-1 **31** sensing microplate reacted with various concentrations of histamine (the numbers indicate the concentrations of histamine in μg mL^–1^). Reproduced with permission from ref (137). Copyright 2011 The Royal Society of Chemistry.

NHS-derivative **30a** has remarkable hydrolytic stability at pH 7.4, with a half-life of 2.5 d at 25 °C. Interestingly, changing its reactive moiety to a thioester caused a large decrease in reactivity: It would only react with cysteine, subsequently undergoing a reaction cascade known as native chemical ligation (NCL; intermolecular conversion of a labile thioester to a stable amide) and eventually showing turn-on behavior. As with the 8-SMe–BODIPY **16** (*cf*. section 6.1), only amino or amide-conjugates gave the desired increase in fluorescence; esters or thiol ethers remained nonemitters. The importance of the correct placement of amino groups on the BODIPY core was highlighted in a paper by Esnal *et al*.^133^ Other dyes bearing such reactive groups could potentially give a similar turn-on behavior upon cleavage of the fluorescence-concealing group, but they have yet to be explored (Figure 14).^134^

### 6.5 Pyrylium Salt Formation

A fascinating approach for the selective detection of amines builds on initial reports by Katritzky^135^ from 1984. The reaction of his, from then on eponymous, pyrylium salts with amines, forming stable pyridinium salts, was taken up by Wetzl *et al*.^136^ Twenty years later, their group modified the pyrylium moieties with color-forming *p*-anilino substituents (**31** and **32**), which resulted in a new class of chromogenic labels, the so-called “Chameleon” labels (Figure 13). The class, although not limited to pyrylium salts, generally shows strong bathochromic shifts upon conjugation. Due to their straightforward synthesis from the corresponding aldehydes and methyl pyrylium salts, they have already been applied in HTS as chromogenic reagents for the detection of biogenic amines (Figure 14).^137,138^

**Figure 15:**
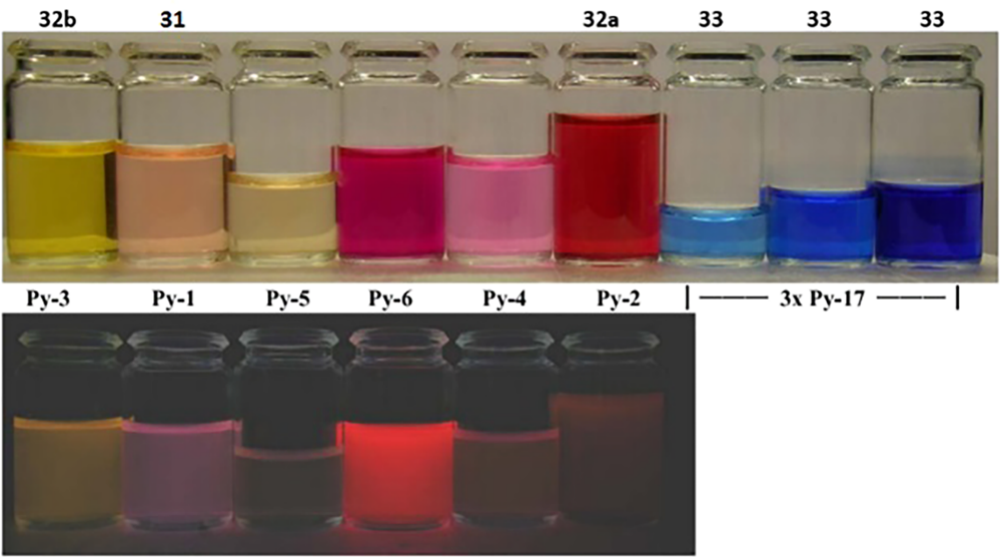
Colors under daylight and the colors of fluorescence in solution of pH 7.0 under a UV lamp (365 nm) of label **31** and of some other Py labels when conjugated to serum albumin at 37 °C. Note that label **33** does not react but remains blue (at any concentration). Reproduced with permission from ref (141). Copyright 2011 The Royal Society of Chemistry.

Although Py-1 **31** is the most widely used derivative,^138−140^ many other spectral bands are addressable by modification of the aromatic core as exemplified by the benzothiazole derivative **33** (Figure 15). The reactivity of those salts could furthermore be fine-tuned by modifying the substituents of the pyrylium moiety.^141^ Primary amines and amino acids (or proteins containing terminal amino groups) could be efficiently labeled at physiological conditions in less than 30 min. Anilines required reactions to be run at 50 °C and in organic solvents. Reactions with secondary amines or ammonia did not form colored products and thus did not interfere with the assay. These probes were stable in acidic solution (pH < 5) but decomposed quickly even at slightly basic pH. The hydrolysis products, however, also did not interfere with the optical read-out.^142^

**Figure 16:**
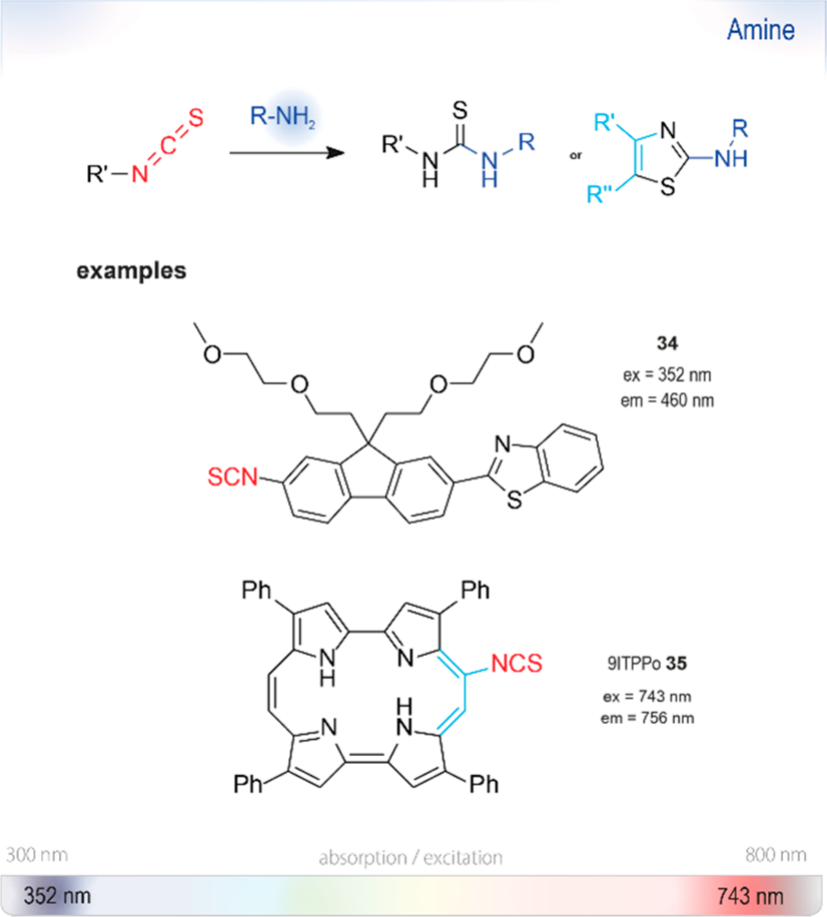
Summary of chromo- or fluorogenic turn-on probes for the selective detection of amines based on the formation of thioureas or 2-aminothiazoles from isothiocyanates. Ex or em: wavelengths typically used for excitation and emission in fluorescence measurements. The wavelengths of probes in this section are shown on the strip at the bottom representing the visible spectrum.

### 6.6 Thiourea or 2-Aminothiazole Formation

Isothiocyanates (ITCs) have long known to be reactive toward a great number of nucleophiles and have been used for the synthesis of stable thiocarbamates and thioureas (Figure 16).^143,144^ Fluorescein isothiocyanate (FITC) is used for protein labeling, relying on the strong nucleophilicity of amines (compared to alcohols or water). It has disadvantages similar to the active esters of fluorescein discussed above: relatively small changes in the electronic structures upon conjugation require separation of the tagged amines from the unreacted label.^145,146^ Thus, only a few examples of efficient use as turn-on probes have been reported.

**Figure 17:**
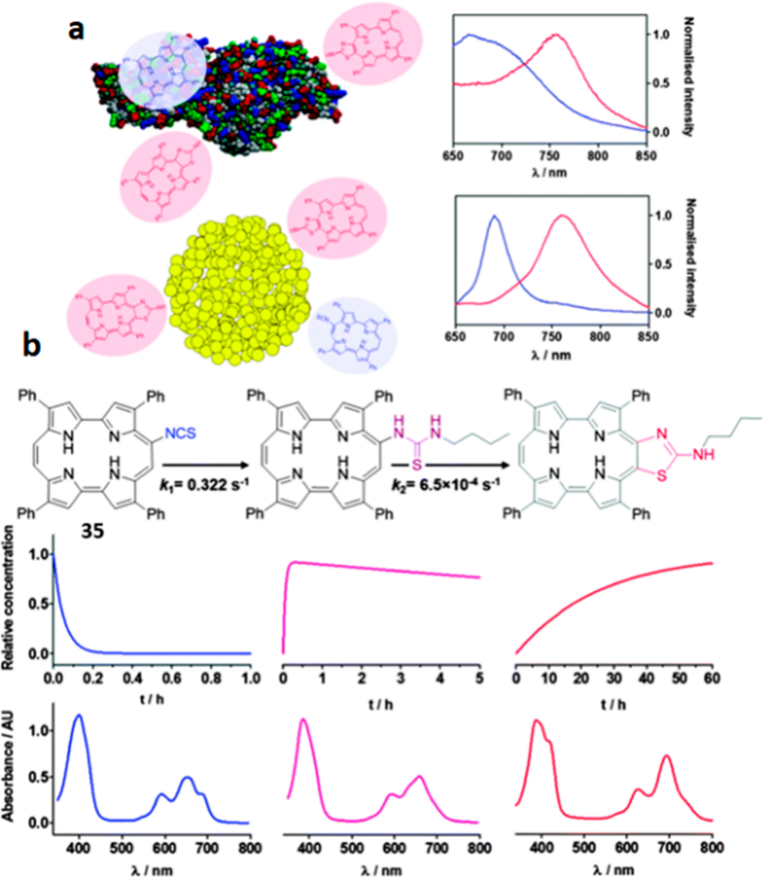
(top) Fluorescence spectra of unbound 9-ITPPo **35** (blue) and its covalent adduct ATAZPo (red) with BSA (a) and gold nanoclusters (AuNCs) (b). (bottom) Absorbance of a mixture of **35** (15 μM) and *n*-butylamine (3 mM) in toluene: Structures, time curves of concentration, and spectral profiles of the reactant, the intermediate, and the product (from left to right). Reproduced with permission from ref (148). Copyright 2015 The Royal Society of Chemistry.

The group of Belfield, experienced in the synthesis of fluorene-based dyes, reported the synthesis and simple modification of isothiocyanate dyes, such as compound **34**, that exhibited a small hypsochromic shift upon conjugation with an amine.^147^ Using their probe, they performed the exemplary derivatization of reelin, a large extracellular matrix glycoprotein, at physiological pH. Although in these experiments the unreacted fluorophore was removed after the reaction, they could show a large increase in quantum yield upon thiourea formation, in DMSO.

Recently, Planas *et al*. used another prominent scaffold, porphycene, as a fluorogenic sensor.^148^ They modified it (9ITPPo **35**; Figure 17) to give a 70 nm Stokes shift to the near-IR upon conjugation, enabling spectral discrimination from the unreacted probe. Isolation of the reaction products of **35** with several primary, secondary, and even aromatic amines revealed that the underlying cause for the color change was a cyclization of the initially formed thiourea into a 2-aminothiazole fragment. Although synthetic methods for this type of cyclization are known,^149,150^ the spontaneous cyclization, particularly for these scaffolds, had previously not been reported.^151^ For a proof of concept, derivatizations of BSA, and amino-functionalized gold nanoclusters were performed in carbonate buffer at pH 9.2 and in EtOH. Although the first step (formation of thiourea) was completed in less than 15 min, the fluorescence continued to shift significantly due to the rate-limiting cyclization, which required prolonged reaction times. Biocompatible auxiliaries to the cyclization might thus lead to the development of new turn-on methods based on these ITC scaffolds.

**Figure 18:**
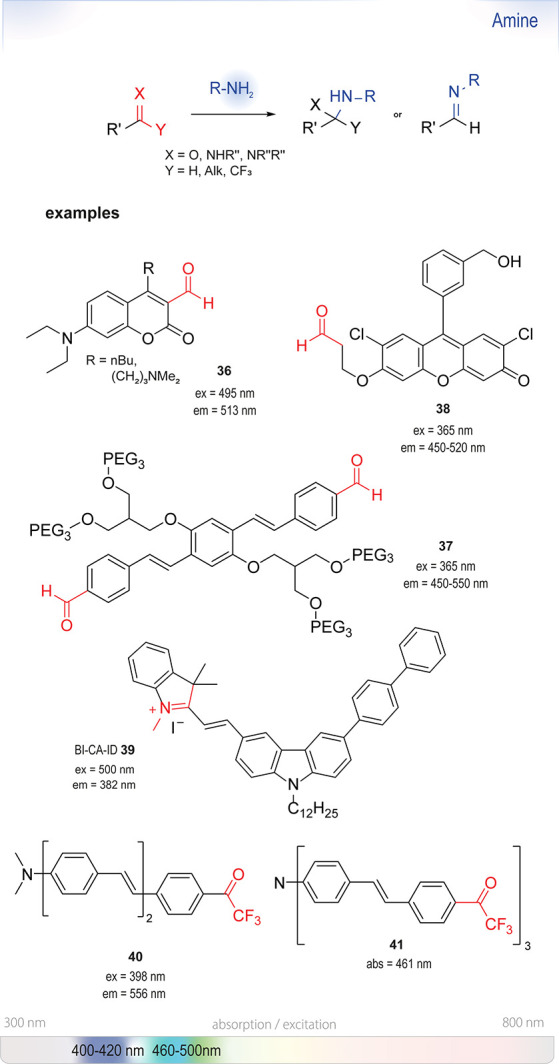
Summary of chromo- or fluorogenic turn-on probes for the selective detection of amines based on hemiaminal, aminal, or Schiff’s base formation. Abs: wavelengths typically used for UV absorbance measurements. Ex or em: wavelengths typically used for excitation and emission in fluorescence measurements. The wavelengths of probes in this section are shown on the strip at the bottom representing the visible spectrum.

### 6.7 Hemiaminal, Aminal, or Schiff’s Base Formation

Another approach for the detection of amines is based on the disruption of electron-withdrawing properties of carbonyl, imine, or imino groups (Figure 18). When amines add to such functional groups and form stable hemiaminals, they tend to induce a hypsochromic shift of conjugated electron systems.^284^ In some cases, further elimination (of water, alcohols, or amines) to imines or iminium salts is favored, and these transformations have also been shown to result in changed spectral features.

**Figure 19:**
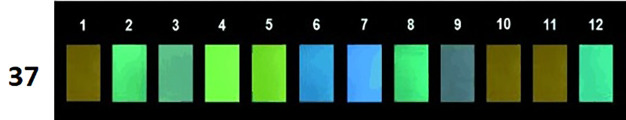
Amine-sensing panels of **37** (10 μmol L^−1^) in water: (1) 37; (2) butylamine; (3) tert-butylamine; (4) benzylamine; (5) cyclohexylamine; (6) ethylene diamine; (7) 1,3-diaminopropane; (8) cadaverine; (9) morpholine; (10) ephedrine; (11) 4-aminopyridine; (12) ethanolamine. Reproduced with permission form ref (154). Copyright 2012 Wiley-VCH.

**Scheme 4:**
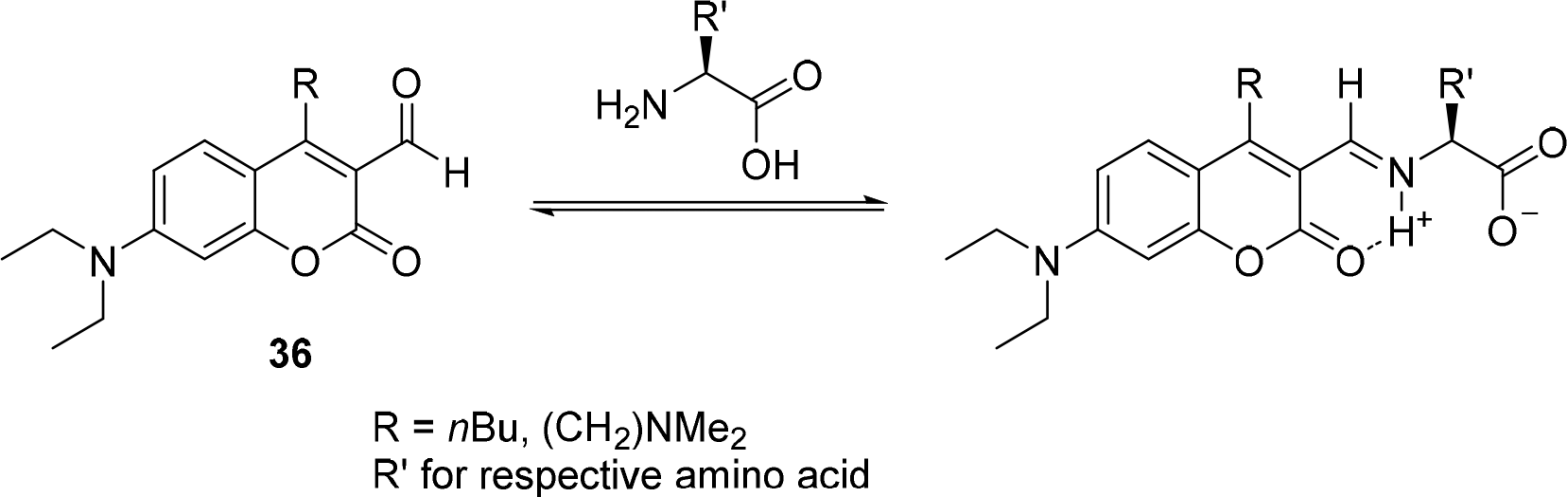
Sensing Mechanism of **36**: Chelation of the Formed Iminium Species by the Coumarin Moiety Triggers a Turn-on Response^152^

The first turn-on probes based on the formation of Schiff’s bases were reported by the group of Glass in 2003:^152^ They used coumarin aldehydes, such as compound **36**, for the detection of amino acids and primary amines. By mimicking chelation-based metal sensors, based on the protonation of the formed imine (Scheme 4), they achieved a 26-fold increase in fluorescence at pH 7.4 (buffered) with glycine as the target. Water-soluble analogues containing aliphatic tertiary amine groups as well as carboxylic acid functionalities were also synthesized but not yet spectroscopically characterized. Alkylation of the 4-position of the coumarin was found to be necessary for a large change in absorption: the imine adopts a *trans*-configuration due to steric repulsion.

A second example emerged from the experience with cross-shaped (“cruciform”) fluorophores by the group of Bunz.^153^ Based on initial designs of such dyes, Kumpf and Bunz synthesized the water-soluble, aldehyde-appended distyrylbenzene **37**, which showed strong turn-on behavior in aqueous systems.^154^ Their reaction with a broad set of (biogenic) primary and secondary amines as well as amino acids at μM concentrations yielded different Stokes shifts upon the formation of hemiaminal or imine structures, with emissions in the range from 450 to 520 nm. Among the tested amino acids, only cysteine, arginine, and lysine gave an increase in fluorescence. The signal strength was strongly dependent on the amine. Whereas morpholine exhibited merely a 10-fold turn-on factor, the reaction with benzylamine or ethylenediamine caused a ∼200–300-fold increase in fluorescence. Compound **37** and its aminal and imine products were stable in neutral aqueous solution.

**Scheme 5:**
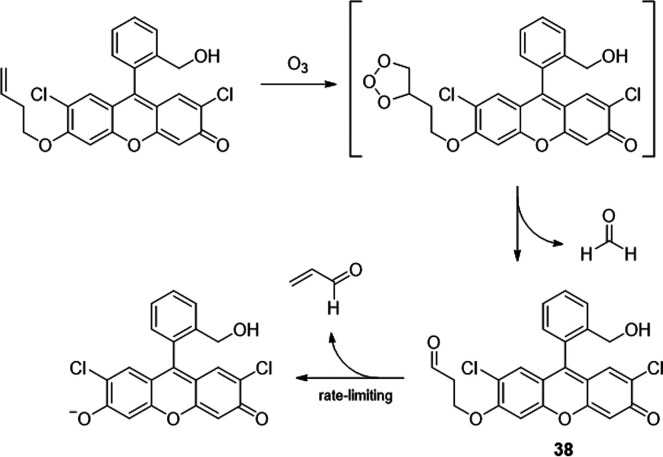
Ozone Sensing Mechanisms Employing **38**.^34^

A recent example of the use of Schiff’s base formation was reported by Leslie *et al*. (Scheme 5).^34^ This study, aiming to optimize their previously reported chemodosimeter for the detection of ozone, revealed that the intermediately formed β-aryloxy fluorescein aldehyde **38** undergoes β-elimination, which is rate-determining and catalyzed by amines. The addition of pyrrolidine increased the rate 30-fold and drove the reaction to completion in less than 3 min; the uncatalyzed reaction took 1.5 h. From a selection of cyclic secondary amines, they identified azetidine as the best catalyst. Anilines were excluded from the reaction screening due to their known reactivity with air and ozone. The authors speculated that the efficiency of the catalyst was primarily dependent on steric effects and the p*K*_a_ of the amine. The rate enhancement was greatest at pH 6 to 7, and the elimination product of **38** showed the highest fluorescence between pH 5 to 9. However, due to the focus of the study on detecting ozone, use of **38** as an amine-selective probe was not investigated.

Another reagent relying on the nucleophilic attack of the amine onto an electrophilic center was developed by Wu *et al*.^673^ Their synthetically readily accessible iminium dye BI-CA-ID **39** effectively recognized primary amines upon reaction by ratiometric discoloration of the dye solution and emergence of a blue-shifted fluorescence emission with a peak at 382 nm. The reaction with primary and secondary amines was reported to be instantaneous. Completely discoloring of sample solutions at 40 μM in less than 1 min (containing 800 μM amine) in 50% ACN (acetonitrile)/water mixtures was observed. Even tertiary amines were reported to be viable for the assay, when allowing reaction times of up to 10 min. Aromatic amines were reported to be completely unreactive toward the probe.

Trifluoroacetyl probes (*e.g*., **40** and **41**) are another class of compounds not yet proven to be applicable in aqueous systems but with frequent occurrence in literature. Although we generally limit this review to reactive probes for aqueous solutions, these compounds deserve an honorable mention. Initial reports by Mohr already exemplified the usefulness of these dyes as potent fluorogenic sensors for amines in organic solvents.^155−157^ At the core, stilbene or azobenzene motifs are responsible for the spectral features of the dyes; they have since then been modified into even larger delocalized structures.^158^ Their kinetics and thermodynamics have been studied,^159^ they have been turned into star-shaped, tripodal fluorophores with variable reactive trifluoroacetyl reactive sites,^160^ and incorporated into a BINOL-scaffold for the detection of diamines.^161−163^ A demonstration of their viability in aqueous systems is yet to be published.

## 7 Chromo- and Fluorogenic Probes for Alcohols

Alcohols are crucial building blocks for the manufacturing of agrochemicals, flavor and fragrance compounds, and pharmaceutical drugs, especially chiral alcohols in their optically pure form. Typical methods to prepare them enantioselectively typically require the use of expensive, complicated chiral catalysts or transition metals, which can be toxic.^164−166^ Biocatalytic approaches are considered an effective and green alternative due to their mild reaction conditions and remarkable enantioselectivity. A recent review by Chen and de Souza highlighted the recent developments in the field.^167^ Although a large variety of biocatalysts is available for the formation of chiral alcohols, only few are used in combination with functional group probes. One possible explanation is the relatively low reactivity of alcohols, which impedes selective targeting, especially in an environment where many competitive, strong nucleophiles are present. Below, we highlight a few pertinent examples, chosen from the many success stories of applications and optimization of such enzymes.

**Figure 20:**
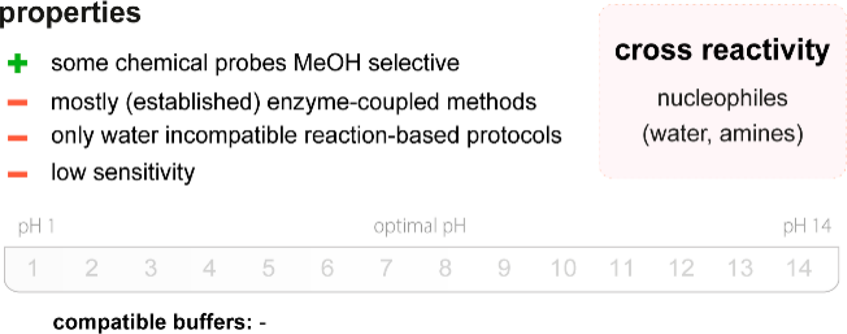
Summary of general properties of alcohols and expected cross-reactivity (bottom).

**Scheme 6:**
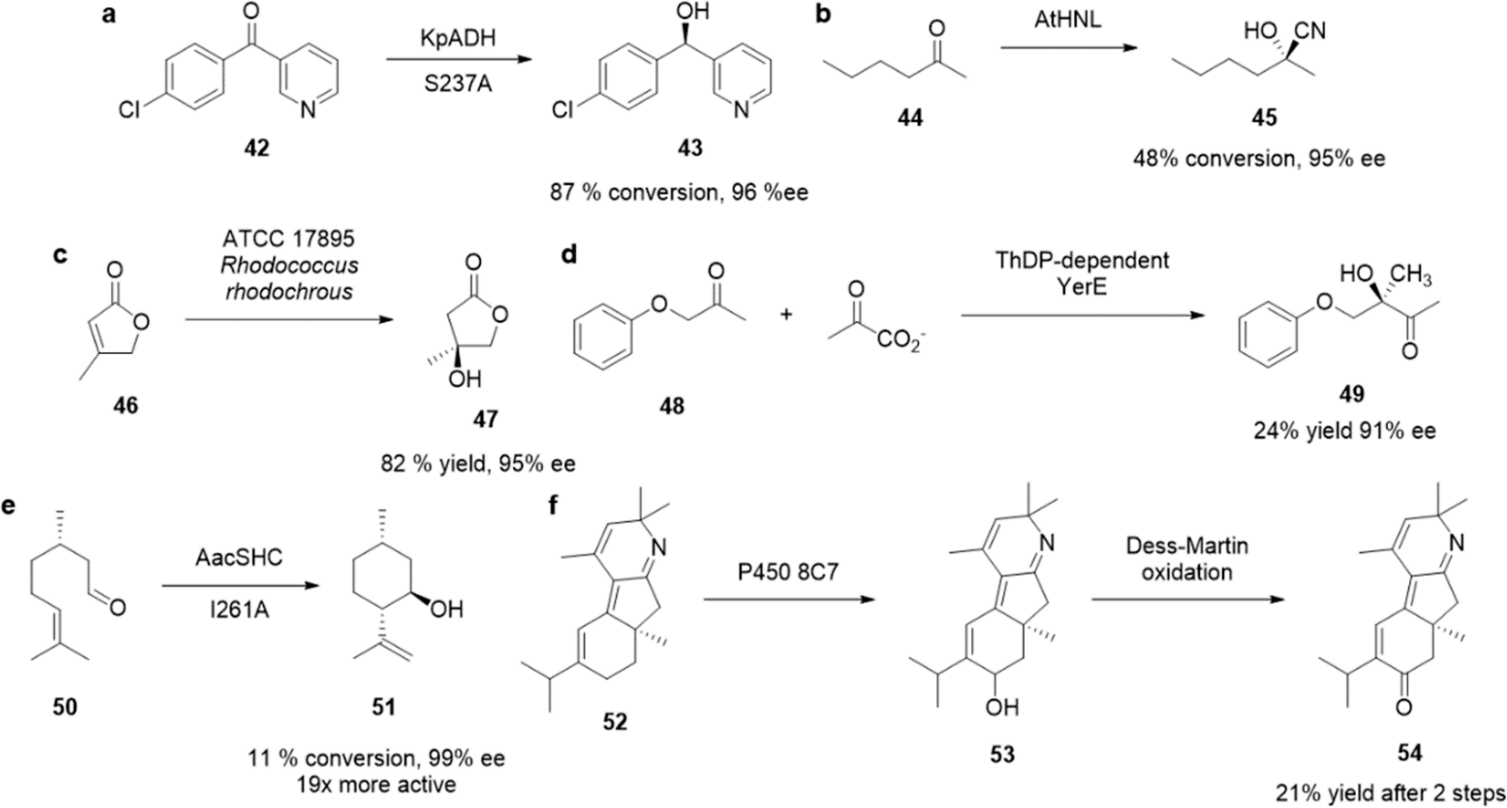
Representative Selection of Enzymatic Transformations for the Production of Alcohol-Containing Products

Nature offers a broad repertoire of catalysts for the synthesis of chiral alcohols (Table 1).^168^ Using directed evolution, Zhang *et al*. achieved the reduction of 1-(4′-chlorophenyl)-1-(pyridine-2′-yl)-methyl ketone (CPMK, **42**; Scheme 6a).^674^ They identified a crucial mutation (S273A) among a library of 2000 clones using the colorimetric DNPH **109** assay to quantify unconverted starting material. In an attempt to identify new enzymes based on sequence similarities, the group of Pohl discovered a new (*R*)-selective HNL from *Arabidopsis thaliana*.^170^ This enzyme converted a wide range of benzaldehydes, and aliphatic aldehydes and ketones, to the corresponding cyanohydrins and was established as an alternative to other known (*R*)-selective HNLs. For instance, the enzyme formed the cyanohydrin of 2-hexanone **44** with 95% ee (48% conversion; Scheme 6b).

**Table 1:** List of Enzyme Classes for the Enantioselective Synthesis of Chiral Alcohols

catalyst	activity
alcohol dehydrogenases (ADH)^171^ and ketoreductases (KRED)^172^	asymmetric reduction of carbonyl compounds
α-ketoacid carboligases^173^ or aldolases^174^	decarboxylative or regular aldol reactions
hydroxynitrile lyases (HNL)^175^	addition of cyanide to aldehydes forming cyanohydrins
(Michael) hydratases^176^	addition of water to unactivated or α,β-unsaturated compounds
epoxide hydrolases^177^	enantioselective hydrolysis of epoxides affording vicinal diols
oxidases^178^	oxidative functionalization of C–H bonds
haloacid dehalogenases,^179^ haloalkane dehalogenases^180^ or halohydrin dehalogenases^181^	hydrolysis of 2-haloalkanoic acids, halogenated aliphatics, or vicinal haloalcohols
lipases^182^ or esterases^183^	(dynamic) kinetic resolution of racemic mixtures

Chen *et al*. tackled the challenging enzymatic Michael addition of water motivated by initial reports for the formation of (*S*)-3-hydroxy-3-methylfuranone **47** from 3-methylfuran-2(5*H*)-one **46** using *Rhodococcus rhodochrous* ATCC 17895.^185^ They compared six closely related *Rhodococcus* strains and found similar activities for the transformation with all six bacteria. Optimization of the process parameters of their initially studied strain enabled the formation of **47** in 82% isolated yield and 95% ee on gram scale (Scheme 6c).

Lehwald *et al*. reported the first asymmetric intermolecular carboligation of an aldehyde and a ketone using a ThDP-dependent flavoenzyme from *Yersinia pseudotuberculosis* (Scheme 6d).^186^ Their catalytic system overcame the known hurdle of low electrophilicity and increased steric bulk of ketones (*e.g*., **48**), which typically hinders aldol reactions with these substrates. The carboligase is promiscuous regarding the substrate, accepting a broad range of cyclic and open-chain ketones, diketones, and α- or β-ketoesters.

An unusual example for the synthesis of chiral alcohols was reported by Hammer *et al*.^187^ They exploited the highly Brønsted-acidic active site of a squalene hopene cyclase (SHC) from *Alicyclobacillus acidocaldarius* and enabled a stereoselective Prins reaction of (*R*)-citronellal **50**. A single point mutation in the active site (I261A) facilitated the stereospecific cyclization to (−)-iso-isopulegol **51**, among the four possible products (Scheme 6e).

Enzymatic late-stage modifications are a flourishing trend in total synthesis. Biocatalysts are uncontested in their selectivity, especially in transformations of highly decorated compounds. The group of Stoltz used an enzymatic approach for the late-stage oxidation of **52** at the C-7 position and screened a small library of cytochrome P450 catalysts.^188^ A double mutant was shown to selectively oxidize the intermediate **52** to the corresponding alcohol **53**, which was subsequently treated with Dess–Martin periodinane to form the desired natural product Nigelladine A **54** (Scheme 6f).

**Scheme 7:**
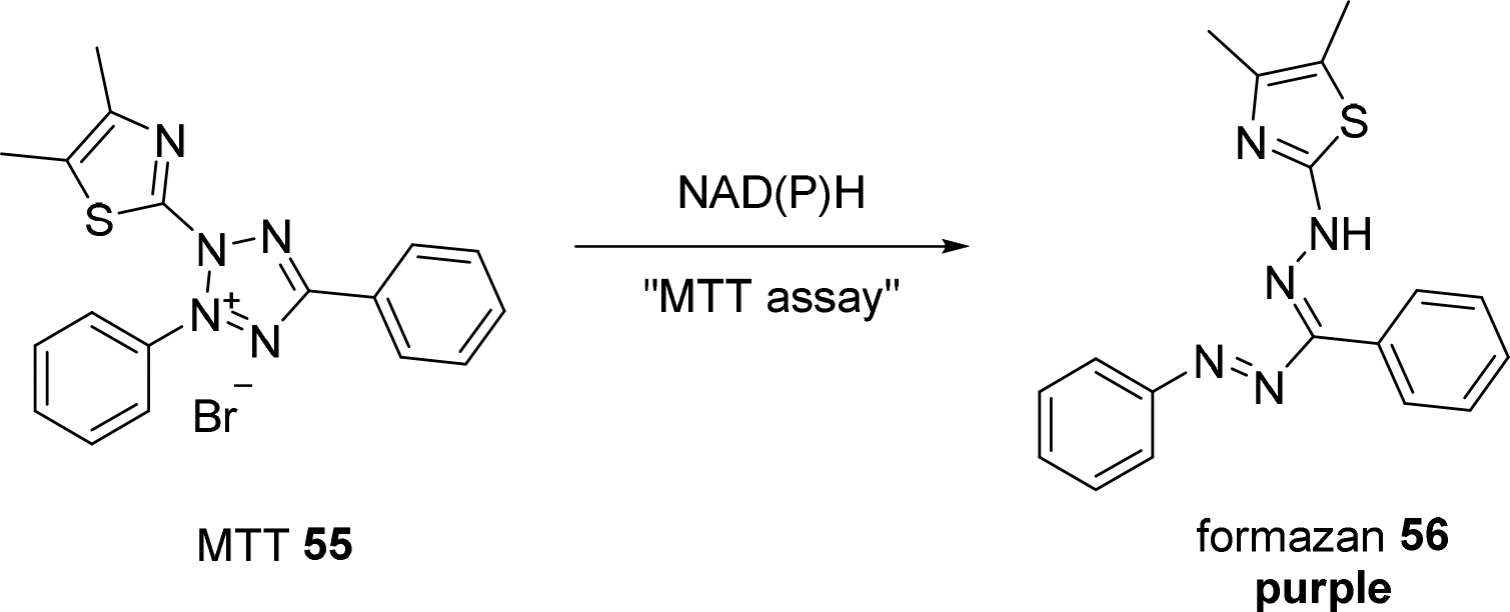
MTT Assay: Detection of NAD(P)H or “Reducing Equivalents” *via* the Reduction of MTT **55** to the Purple Formazan **56**

Many protein engineering efforts rely on indirect detection methods using coupled enzymatic reactions, summarized below. The most widely applied protocol oxidizes the produced alcohols with appropriately chosen alcohol dehydrogenases (ADHs) or alcohol oxidases (AOs), generating NADH, which can be quantified *via* the reduction of MTT, 3-(4,5-dimethyl-2-thiazolyl)-2,5-diphenyl-2*H*-tetrazolium bromide **55**, forming purple-colored formazan **56** (λ_abs,max_ = 550 nm) (Scheme 7).^189−191^

The oxidized products can also be directly detected using carbonyl-selective assays (*cf*. sections 9 and 10). Chromogenic Purpald **113** has already been used for this purpose by detecting formaldehyde (FA), for example, in the directed evolution of a terpene synthase^192^ and in engineering a CYP153A35 oxidase to enhance its ω-hydroxylation activity toward palmitic acid.^193^

Luciferase can be used to further oxidize aldehydes (produced *via* ADH catalysis from the target alcohols) to quantify alcohol production by the emergence of a blue-green luminescence (λ_em,max_ = 490 nm).^194,195^

The drawbacks and limitations of these enzymatically coupled detection methods have motivated the development of alcohol-sensitive probes despite the known issues with selectivity in relevant reaction environments. These probes rely either on an approach similar to that described for detecting amines (nucleophilic attack on electrophilic carbonyl species, *cf*. section 6.7) or on coordinating metals. Unfortunately, these methods have been found broadly incompatible with aqueous media or poorly sensitive, so far impeding their application in protein engineering. The competition with other nucleophilic species, biogenic amines, but mostly water, creates too much noise in detection. Separation of the analytes from competing nucleophiles (*e.g*., extraction into an organic solvent) might ultimately be necessary to overcome these limitations (Figure 20).

### 7.1 Hemiacetal Formation

**Figure 21:**
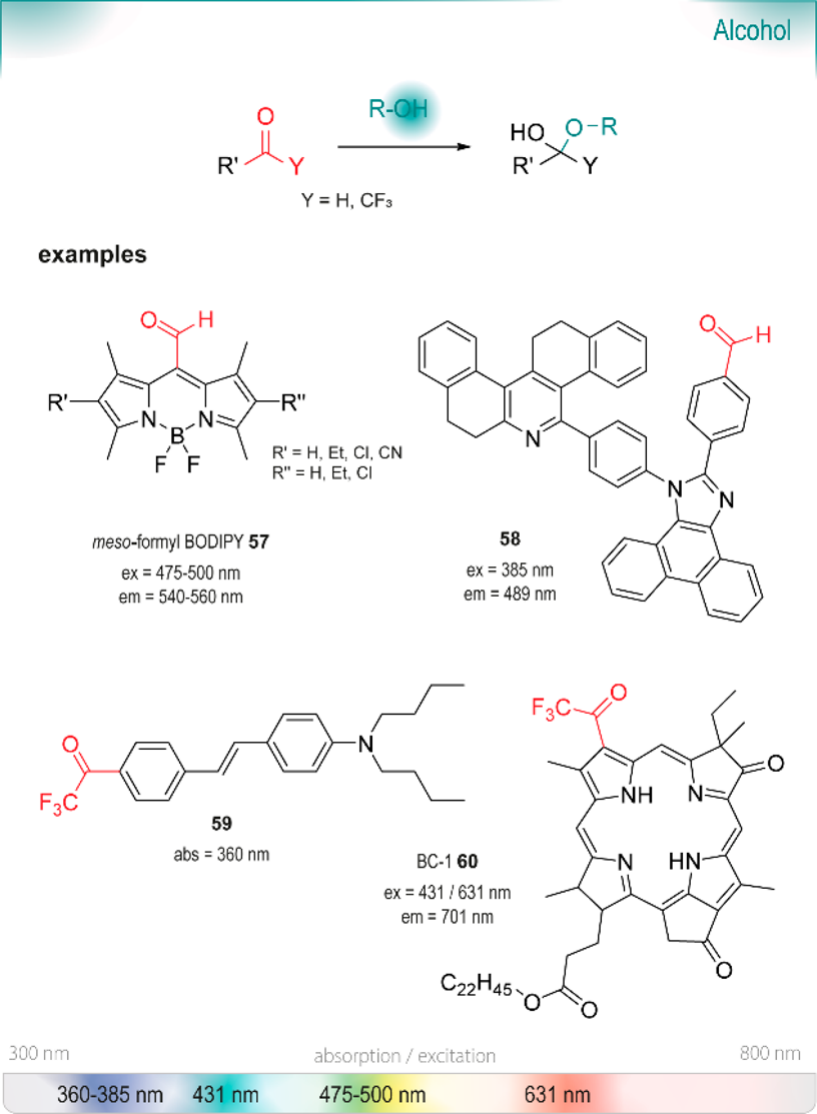
Summary of chromo- or fluorogenic turn-on probes for the selective detection of alcohols based on hemiacetal formation. Abs: wavelengths typically used for UV absorbance measurements. Ex or em: wavelengths typically used for excitation and emission in fluorescence measurements. The wavelengths of probes in this section are shown on the strip at the bottom representing the visible spectrum.

The reversible formation of hemiacetals from aldehydes or trifluoroacetyl groups can be used to detect alcohols, as the addition of the alcohol changes the spectral properties of these probes (Figure 21). This mechanism is identical for amino and carboxylate probes and will create background signals in the presence of these substance classes (*cf*. sections 6.7 and 11.3).

**Figure 22:**
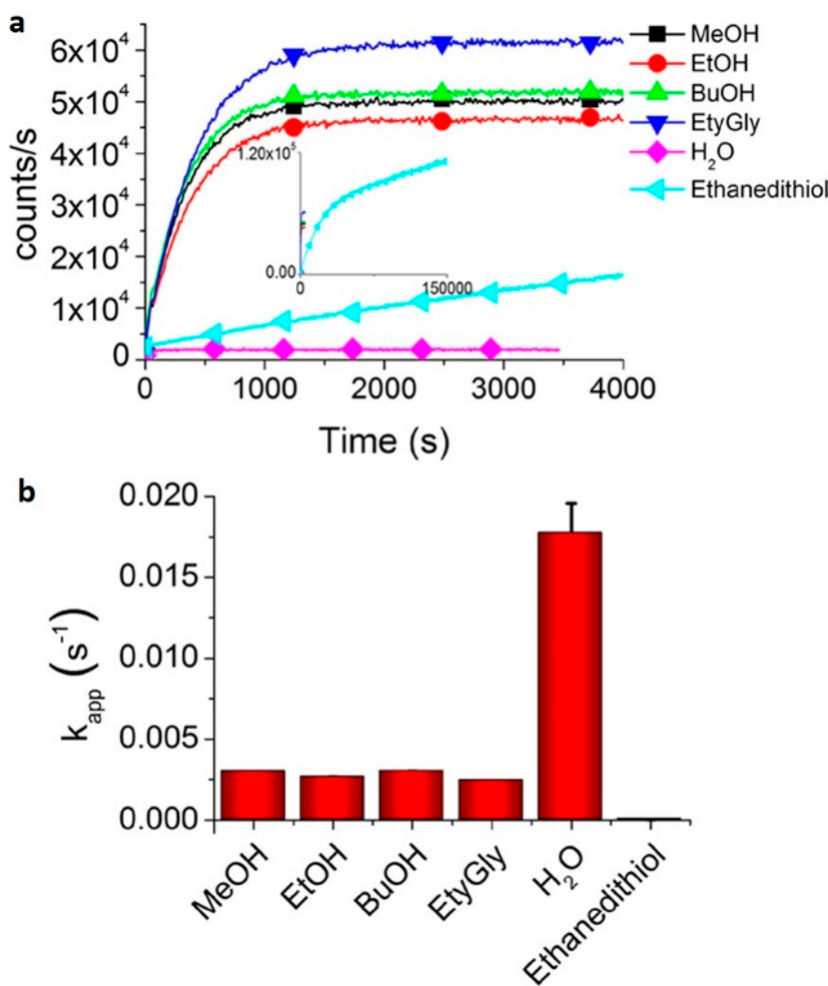
(a) Fluorescence–time trajectories following the reaction of 4.5 μM of compound **57** (R*′* = Cl, R′′ = H) and 1.09 M methanol (MeOH), ethanol (EtOH), butanol (BuOH), ethylene glycol (EtyGly), water (H_2_O), or ethanedithiol at 21 °C in ACN supplemented with 0.333 mM of *p*-TsOH. (b) Apparent rate constants for various nucleophiles tested were obtained from fitting the intensity–time trajectories in (a). Reproduced with permission from ref (196). Copyright 2017 American Chemical Society.

The group of Cosa applied this method with their *meso-*BODIPY scaffolds **57**, which become fluorescent upon nucleophilic addition of primary alcohols (Figure 22).^196,197^ Conversely, amines formed nonfluorescent imines. Incorporation of electron-withdrawing substituents (*e.g*., CN) onto the BODIPY core and the use of catalytic amounts of *p*-toluenesulfonic acid strongly improved the reaction rate. Still, the reaction with water was 10 times faster, and assays had to be performed in dry organic media.

**Figure 23:**
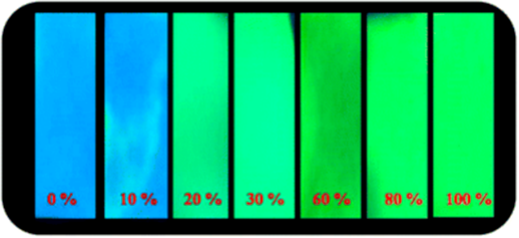
Photographs showing the color changes of sensor **58** before and after the addition of methanol vapor. The images were taken under UV irradiation (365 nm). Reproduced with permission from ref (198). Copyright 2021 Royal Society of Chemistry and the Centre National de la Recherche Scientifique.

Sawminathan and Iyer designed the polarity-sensitive fluorescent dye **58**, based on an imidazole core functionalized with a conjugated push–pull system.^198^ Addition of MeOH and EtOH triggered a hypsochromic shift of the fluorescence maximum, enabling the ratiometric detection of these alcohols in ACN. Compound **58** was applied for the determination of MeOH in biodiesel and used to detect vapors with paper-based test strips (Figure 23).

Mohr *et al*. extensively studied solvatochromic trifluoroacetyl stilbenes (*e.g*., compound **59)**,^156,199,200^ which are used for the detection of amines (*cf*. section 6.7) and found a hypsochromic shift of absorption upon hemiacetal formation in many apolar and polar organic solvents. The reaction with EtOH could be either followed by an increase in absorbance at 360 nm or as fluorogenic turn-off probe (λ_ex,max_ = 450 nm, λ_em,max_ = 570 nm) in buffered media. The fluorescent effect was only observed at concentrations of EtOH above 10 v/v%.

Takano *et al*. developed the trifluoroacetyl compound BC-1 **60**, synthesized from a lyophilized bacteriochlorin derivative.^201^ This alcohol-sensitive probe fluoresces upon excitation either at 431 or 731 nm, circumventing potential optical interference from the sample. The probe was adsorbed on PVC (because the study was focused on developing alcohol-responsive membranes), giving a 2.5-fold increase in fluorescence with 25 v/v% EtOH in buffered solutions at pH 4–8.

### 7.2 Coordination to Metals and Chelation

**Figure 24:**
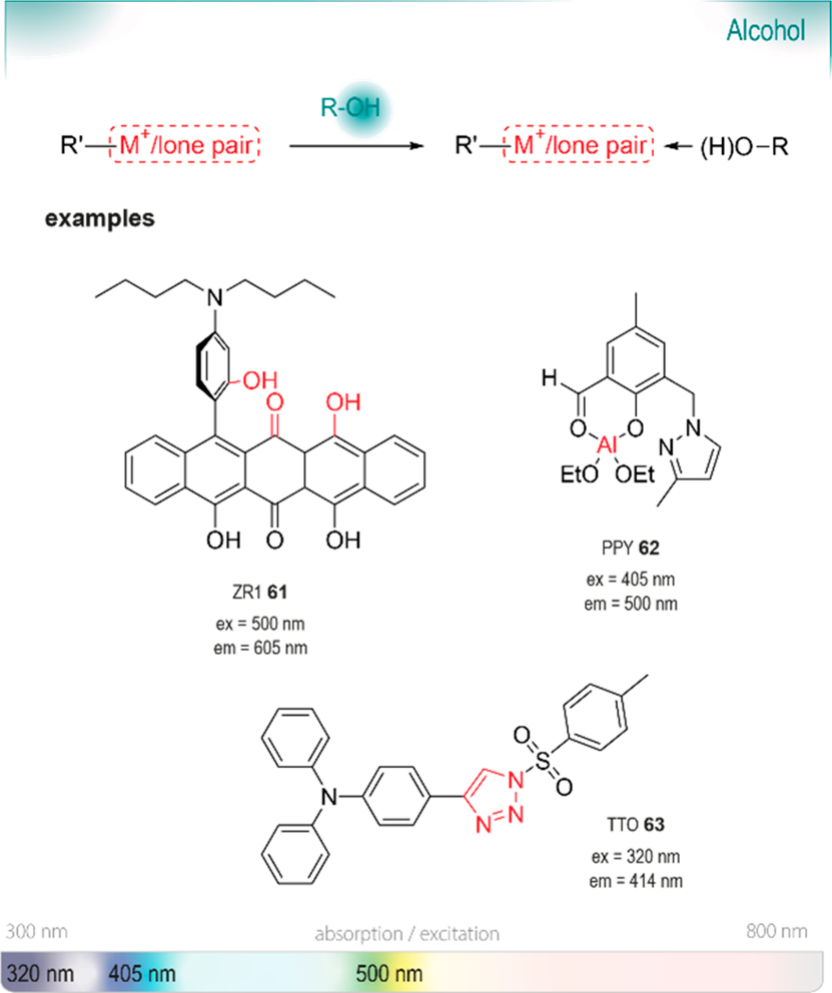
Summary of chromo- or fluorogenic turn-on probes for the selective detection of alcohols based on the coordination to chelation centers. Ex or em: wavelengths typically used for excitation and emission in fluorescence measurements. The wavelengths of probes in this section are shown in the strip at the bottom representing the visible spectrum.

Alcohols can be detected using reactions where they act as ligands, are coordinated to metals, or are chelated by conformationally rigid organic structures (Figure 24). Zhao *et al*. developed the probe ZR1 **61**, which tautomerizes upon coordination to an alcohol,^202^ causing an increase in ICT and concomitant increase in purple color and fluorescence (at 605 nm). The reaction is selective for MeOH; other alcohols did not react. This selectivity was demonstrated by titrating an ethanolic solution of the probe with methanol. The presence of water was problematic: concentrations as low as 15% eradicated the signal, making an application in aqueous environments impossible.

A probe developed by Roy *et al*. uses an aluminum species for the selective detection of MeOH.^203^ Displacement of coordinating ethoxide on PPY **62** by MeOH caused a change in geometry from the tetrahedral to the octahedral complex, suppressing intramolecular PET and causing an approximately 7-fold increase in fluorescence at 405 nm. A small percentage of water (5 v/v%) was necessary to observe the emission. Higher concentrations than 5% were detrimental, leading to complete annihilation of the signal at 75% and above.

The group of Wang developed a so-called donor−π-system–acceptor (D−π–A) probe TTO **63**, using click chemistry to form the triazole as a coplanar π–electron bridge between the sensing units.^204^ This approach enabled the facile synthesis of several structural analogues. The emissive properties of the probe were strongly dependent on the solvent and were shown to be selective for MeOH; there was only weak fluorescence with EtOH and none with any higher alcohols. Similar to **62**, an increase in water concentration was detrimental to the observed signal intensity.

### 7.3 Ring Opening of 3-Aminorhodanine

**Figure 25:**
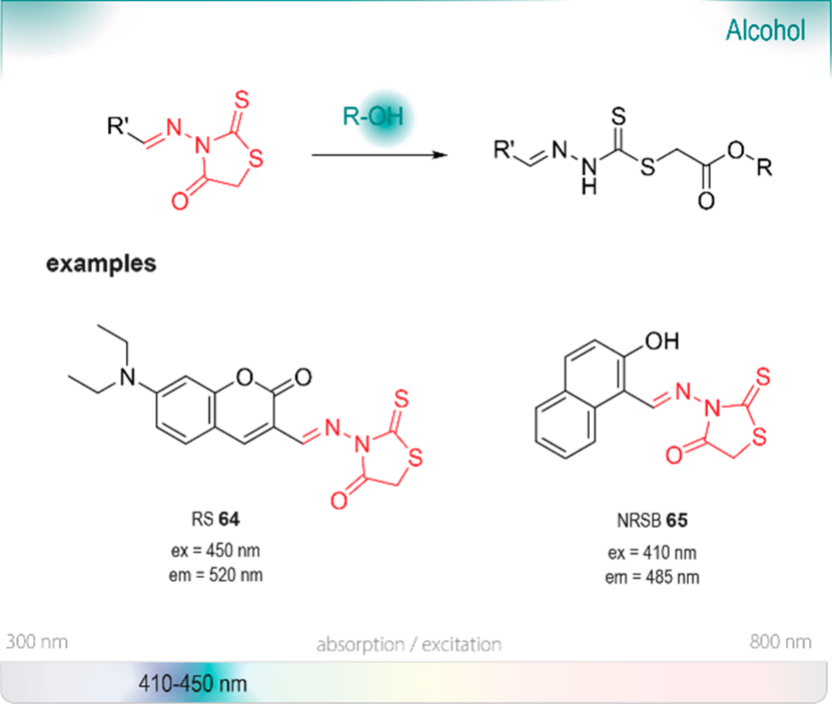
Summary of chromo- or fluorogenic turn-on probes for the selective detection of alcohols based on the ring-opening of 3-aminorhodanine derivatives. Ex or em: wavelengths typically used for excitation and emission in fluorescence measurements. The wavelengths of probes in this section are shown in the strip at the bottom representing the visible spectrum.

A new strategy for turn-on probes responsive to MeOH was reported by Kumar *et al*. They synthesized Schiff’s bases of 3-aminorhodanine with aldehyde-bearing fluorophores, which undergo ring-opening methanolysis at the imide C–N bond (Figure 25). The probes are typically nonemissive due to inhibited isomerization of the C=N bond and additionally active PET from the lone pair of the aldimine N atom to the fluorophore. The initially studied coumarin probe RS **64** was proven to be unreactive toward water and alcohols, including EtOH, *n*PrOH, and *n*BuOH; other nucleophiles were not tested.^205^

**Figure 26:**
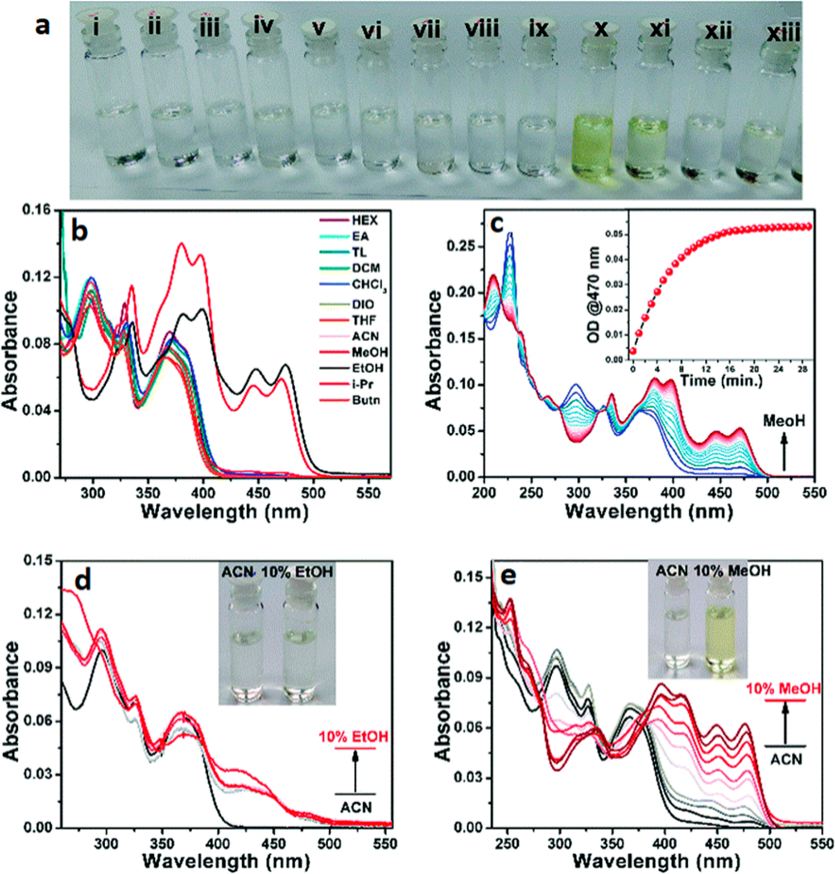
(a) The visible color change of **65** (5 mM) in different solvents: (i) hexane, (ii) toluene, (iii) cyclohexane, (iv) ethyl acetate, (v) chloroform, (vi) dichloromethane, (vii) tetrahydrofuran, (viii) dioxane, (ix) ACN, (x) MeOH, (xi) EtOH, (xii) *i*PrOH, and (xiii) *n*BuOH. (b) The absorption spectra of **65** in different solvents, (c) time-dependent absorption spectra (0–30 min) of 5 mM **65** in MeOH; inset: plot of optical density at 470 nm *vs* time. (d,e) Absorption spectra of **65** (5 mM) at different ratios of ACN:EtOH (0–10%) and ACN:MeOH (0–10%), respectively. Reproduced with permission from ref (207). Copyright 2019 Royal Society of Chemistry and the Centre National de la Recherche Scientifique.

The function of the probe was demonstrated in aqueous and ethanolic solutions after 15 min reaction times, with a limit of detection (LOD) of 0.042 wt % MeOH in water. Later, the authors reported a hydroxynaphthalene analogue NRSB **65**,^207^ which had similar functionality and reactivity trends, except for EtOH (also detected). Incubation with increasing MeOH concentrations (0–10 wt %) in ACN demonstrated a potential for colorimetric detection with the naked eye (LOD: 0.46 wt %; Figure 26).

The conjugate of **65** with MeOH was only weakly fluorescent. The addition of Al^3+^ to the ring-opened product greatly increased the emission due to chelation-enhanced fluorescence (CHEF) by inhibiting *cis*–*trans* isomerization as well as PET. Hence, both MeOH and Al^3+^ are necessary for a strong turn-on response.

## 8 Chromo- And Fluorogenic Probes for Diols

**Figure 27:**
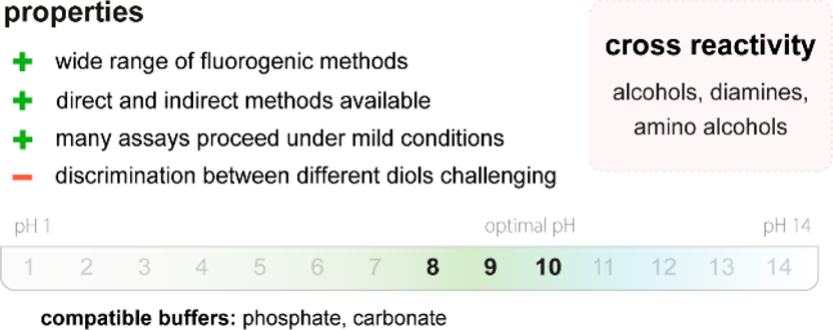
(top) Summary of general properties of diol sensors and expected cross-reactivity. (bottom) Compatible pH range and buffers in reported diol-selective assays. Bold numbers indicate the most commonly used pH values.

Diols are found in many relevant compounds, including pharmaceuticals^208,209^ and natural products.^210,211^ They are used as precursors^208,209,212−215^ in the synthesis of sulfites, sulfates, epoxides, and carbonyl compounds.^216,217^ Cyclohexadiene diols have been frequently used as starting material for carbohydrates, alkaloids, prostaglandins, and terpenes.^218^ The reactivity of diols is similar to that of alcohols: They can react as nucleophiles forming ethers, or esters with a carbonyl donor (Figure 27). Furthermore, they can be oxidized to carbonyl compounds or undergo dehydration or condensation reactions used in polyester synthesis.^217,219,220^

**Scheme 8:**
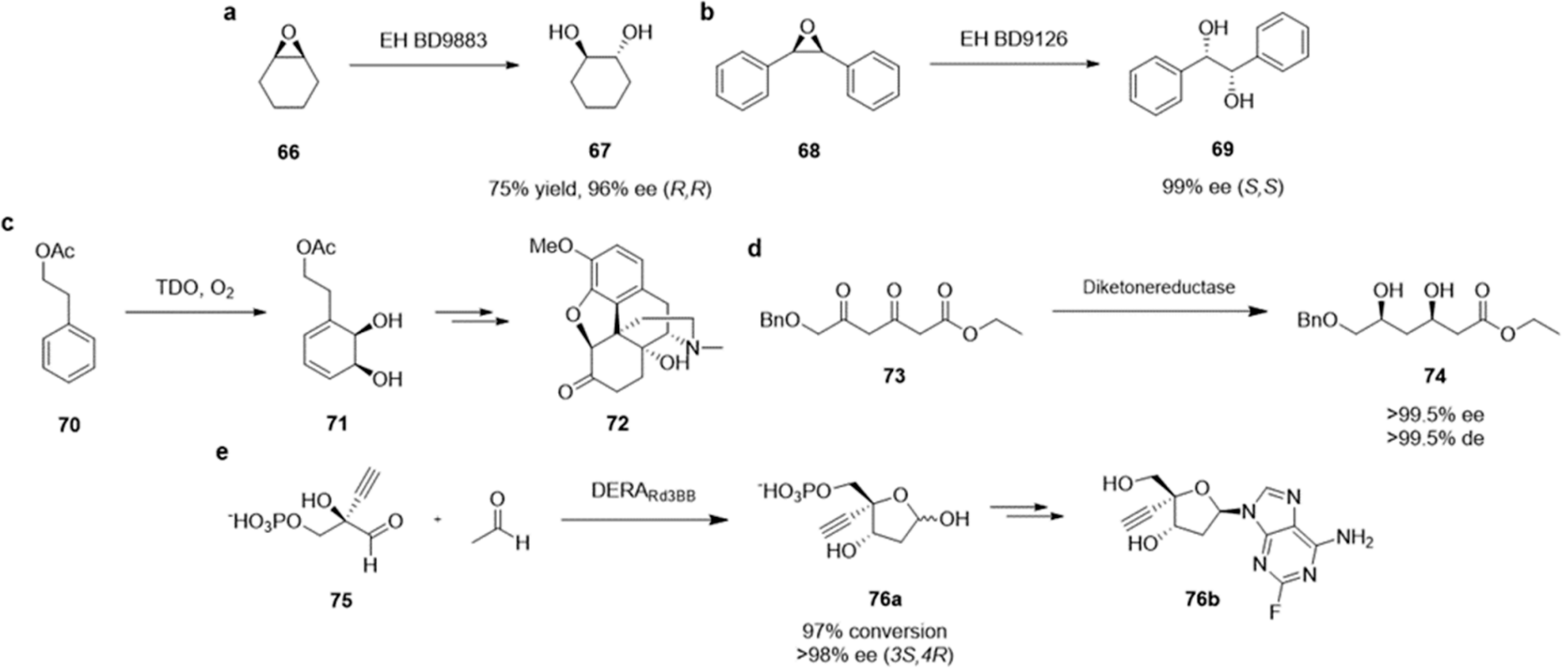
Representative Selection of Enzymatic Transformations for the Production of Diol-Containing Products

Common enzymatic approaches to generate vicinal diols use epoxide hydrolases,^221^ dicarbonyl reductases,^209^ or dioxygenases.^222,223^ Zhao *et al*. reported the discovery of several microbial epoxide hydrolases (EH) that enabled the diastereoselective synthesis of (*R*,*R*)-diol **67** or (*S*,*S*)-diol **69***via* desymmetrization of various aliphatic and aromatic *meso*-epoxides **66** and **68** (Scheme 8a,b).^224^ Makarova *et al*. designed four different synthetic routes for *ent*-oxycodone **72**. In all approaches, the initial synthetic step employed a toluene dioxygenase (TDO) to obtain a diene-diol **71** from a whole-cell fermentation with *E. coli* (Scheme 8c).^225^

Enzymatic procedures yielding 1,3-diols include the enzymatic reduction of 1,3-diketones with *Pichia farinosa* IAM 4682,^226^ aldol additions with aldolases,^227^ or the reduction of hydroxy ketones with ketoreductases.^228^ Wu *et al*. employed a diketoreductase from *Acinetobacter baylyi* for the stereoselective double reduction of ethyl 3,5-diketo-6-benzyloxy hexanoate **73** to ethyl 3*R*,5*S*-dihydroxy-6-benzyloxy hexanoate **74**, which is used in the synthesis of statin drugs (Scheme 8d). They optimized the whole-cell catalytic system coexpressing diketoreductase and glucose dehydrogenase to work without added cofactors at a high substrate concentration (15-fold increase from base level).^229^ Deoxyribose 5-phosphate aldolase (DERA) from *Shewanella halifaxensis* was used to synthesize the 1,3-diol **76a**, an intermediate in the biocatalytic synthesis of islatravir **76b** (Scheme 8e). In a nine-enzyme cascade, the product could be obtained in a single aqueous solution without isolation steps in 51% yield.^230^

Numerous diol-sensing assays employ boronic acid-based probes and make use of the formation of fluorescent cyclic boronate esters as a read-out. Works in this area have been reviewed extensively.^211,231−235^ Other approaches are based on indirect detection, such as converting diols to aldehydes and their subsequent detection with aldehyde-sensing assays. It remains challenging to detect diols selectively. Particularly, the discrimination between carbohydrates with minor differences in reactivity and stereochemistry is problematic.^236^

### 8.1 Esterification of Boronic Acids

**Figure 28:**
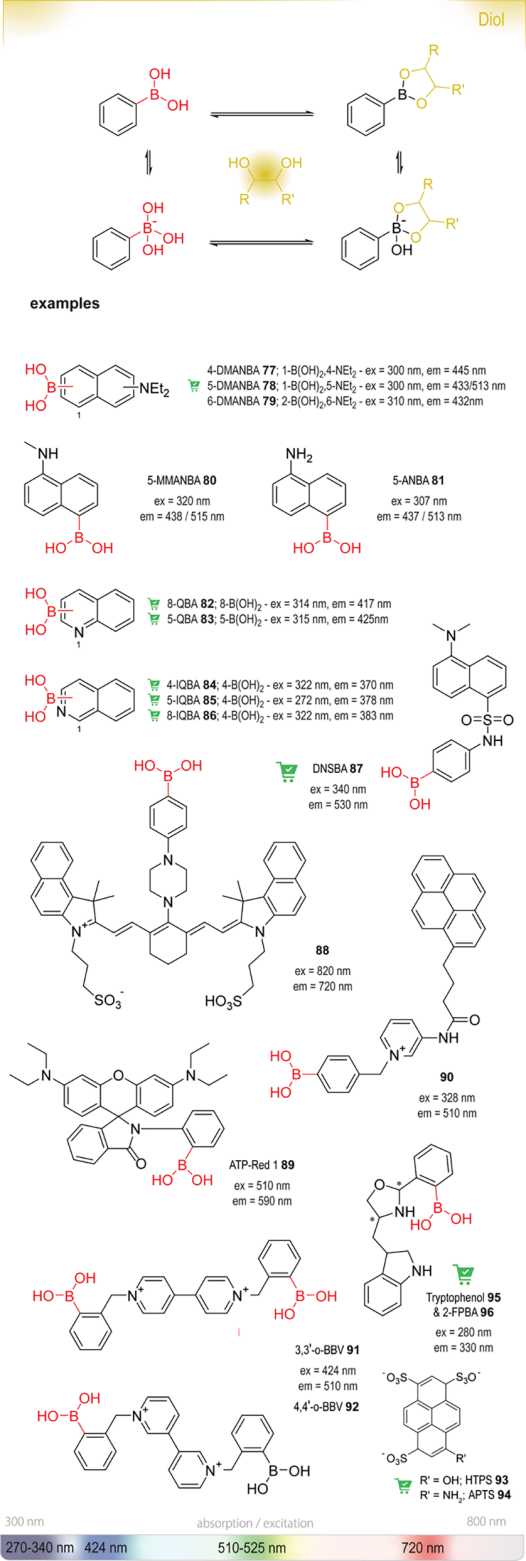
Summary of fluorogenic turn-on probes for the selective detection of diols based on the formation of fluorescent boronate esters. Ex or em: wavelengths typically used for excitation and emission in fluorescence measurements. The wavelengths of probes in this section are shown in the strip at the bottom representing the visible spectrum.

One approach to detect 1,2- or 1,3-*cis*-diols is to use boronic acid derivatives: They react reversibly in aqueous solution, forming cyclic boronates (Figure 28).^237^ Since initial reports by Czarnik *et al*. and Shinkai *et al*., various boronic acid-based fluorescent probes have been developed.^238,239^ In 1992, Yoon and Czarnik were the first to describe the use of anthranylboronic acid as a fluorescent probe for polyols in water.^239^ In general, fluorescent boronic acid-based probes consist of a fluorophore as a signal unit and a boronic acid as a recognition site.^240^ Probes with various fluorophores, including naphthalene, anthracene, quinoline, coumarin, cyanine, and BODIPY, have been developed.^231,232^ Upon binding of the diol, a change in fluorescence based on processes, like PET, ICT, or FRET, can be observed.^211,232,235,241^

The interaction between boronic acids and diols is pH-dependent. Boronic acids (neutral) are trigonal planar, whereas the boronates (anionic) are tetrahedral; their esters behave analogously. Usually, the p*K*_a_ values of the boronate esters are lower than those of boronic acids.^235^

In general, equilibrium constants of ester formation with neutral boronic acids are lower than with boronates. Therefore, common hypotheses are that (i) diol binding is favored at a higher pH value when mainly anionic boronate is present and (ii) that boronic acids with low p*K*_a_ values tend to have high affinities for diols, and (iii) that a pH value higher than the p*K*_a_ of the boronic acid is favorable for diol binding. There are reports contradicting these statements, indicating that not only the p*K*_a_ of the boronic acid but also the p*K*_a_ of the diol needs to be considered.^235,242^

Steric hindrance, buffer composition, and the solvent play a crucial role as well. Generally, diols with a small distance between their oxygen atoms bind to boronic acids with higher affinity, quantified by a small (and constrained) dihedral angle in the diol and consequently an O–B–O bond angle that favors sp^3^ hybridization of the tetrahedral molecule.

Boronic acids are Lewis acids and might interact with buffer components in that way, particularly if the buffer contains Lewis bases, *e.g*., phosphate or chloride, influencing how the probe reacts with water or diols.^235,243,244,242^ Cross-reactivities might arise with functional groups such as amino alcohols, cyanides, alcohols, fluorides, α-hydroxy acids, or α-amino acids.^241,243^

Many boronic acid-based probes have been developed for various structurally different saccharides. Gao *et al*. developed the naphthalene- and ICT-based probe 4-(dimethylamino)naphthalene-1-boronic acid (4-DMANBA, **77**) to detect saccharides in phosphate buffer at pH 7.4. The amino group acted as a donor and the boron atom as an acceptor in the ICT process. A 41-fold increase in emission intensity at 445 nm was observed upon adding fructose, which was the largest reported increase with an ICT-based probe at that time. Besides fructose, also galactose, glucose, sorbitol, and tagatose showed fluorescent responses. Moreover, the water-soluble probe could be readily synthesized in one step.^245^ The authors observed that a change in the substitution pattern strongly influenced the fluorescence properties. The derivative 6-DMANBA **79** proved to be a turn-off fluorescent probe, whereas 5-DMANBA **78** is a ratiometric probe showing large changes in fluorescence intensity at 513 and 433 nm upon binding to diols.^246,247^

Zhang *et al*. described several water-soluble naphthalene-based probes for saccharides and observed that 5-(monomethylamino)-naphthalene-1-boronic acid (5-MMANBA, **80**) and 5-aminonaphthalene-1-boronic acid (5-ANBA, **81**) showed an even higher increase in emission intensity (71-fold and 66-fold, respectively) upon the addition of fructose than previously reported structural analogues.^248^ From their studies of carbohydrates on the surfaces of cells, Yang *et al*. identified quinoline fluorophores as particularly stable and water-soluble analogues. For example, 8-quinolineboronic acid (8-QBA, **82**) is a useful probe for the detection of carbohydrates in aqueous solutions at physiological pH. At a pH < 5, background fluorescence was observed. Addition of fructose led to a 47-fold fluorescence increase at pH 7.5. A fluorescence enhancement was also observed with galactose, tagatose, and arabinose.^249^ The structural analogue 5-QBA **83** exhibited a similar enhancement of fluorescence. Its binding constants were higher than **82**, and the probe could be applied from pH 3.5 and higher.^250^

Cheng *et al*. developed various fluorescent isoquinolinylboronic acid-based probes (IQBA) **84**–**86**, some with opposite changes in fluorescence intensity depending on whether fructose or glucose was added. Weak binding of these probes to *cis*-cyclohexanediol and binding of some probes with methyl α-d-glucopyranose as a model glycoside was observed.^251^

Elfeky reported a probe, 4-(5-dimethylamino-naphthalene-1-sulfonamido)-3-(4-iodophenyl)butanoic acid (DNSBA **87**), which, when combined with a diol quencher, showed fluorescence recovery in the presence of various saccharides and nucleosides at pH 8.2. In contrast, the binding of the hydroxylated acids citric acid, tartaric acid, and glucuronic acid prompted a decrease in fluorescence intensity.^240^

Near-infrared (NIR) probes have several advantages over emitters in the visible range, particularly no interference from the autofluorescence of biomolecules. Samaniego Lopez *et al*. developed a water-soluble NIR turn-on probe **88**, which consisted of a tricarbocyanine, a piperazine unit as a linker, and a boronic acid moiety. They observed that the selectivity was pH-dependent: at pH 7.4, the probe had a high affinity for fructose and sorbitol but not for galactose, glucose, or mannose. Increasing the pH from neutral to basic increased the fluorescence. The probe also showed a fluorescent response upon binding to mucin, human serum albumin, and asialofetuin.^252^

Wang *et al*. reported the fluorescent, rhodamine B-based probe ATP-Red 1 **89**, which rapidly and selectively reported intracellular ATP levels using a multisite-binding strategy. In the presence of ATP, the boronic acid formed an ester with the ribose moiety of ATP, and π–π stacking between the xanthene and the adenine moieties and electrostatic interactions of the amino group with the phosphate groups took place.^253^

Huang *et al*. introduced a concept to improve selectivity, based on ensembles of boronic acid receptors (**90**). Interactions of π–electron systems and the pyridinium cation and interactions between the diols and the boronic acids led to the formation of conjugates with d-fructose, (unordered 1:1 stoichiometry) and with d-glucose (ordered 2:1 stoichiometry). The ordered complex gave strong fluorescence at 510 nm, but the unordered was only weakly fluorescent at 380 and 400 nm. Binding of the probe to d-glucose was approximately four times stronger than with d-fructose. To improve this ratio, Huang *et al*. added phenylboronic acid, which binds to fructose 40 times stronger than to glucose, to improve the selectivity for glucose.^254^

**Figure 29:**
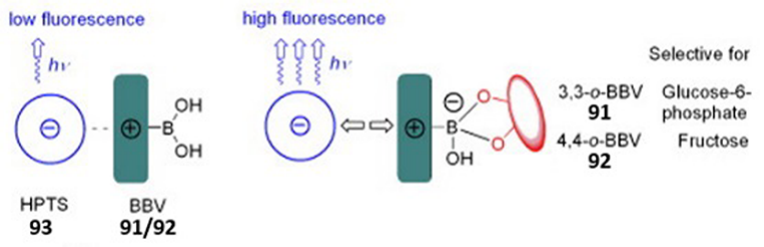
Sensing mechanism for the selective detection of fructose or glucose-6-phosphate by **91** or **92** and viologen HPTS **93**. Reproduced with permission from ref (255). Copyright 2009 Elsevier BV.

Another concept for carbohydrate detection with boronic acid derivatives uses the combination of a cationic viologen compound linked to a boronic acid moiety and a fluorescent anionic dye. The fluorescence of the dye is quenched by coordination to the viologen compound and restored in the presence of saccharides. Vilozny *et al*. applied a system composed of the boronic acid-appended viologens 3,3′-*o*-BBV **91**/4,4′-*o*-BBV **92** and commercially available, fluorescent 8-hydroxypyrene-1,3,6-trisulfonic acid trisodium salt (HPTS, **93**) for enzymatic assays of sucrose phosphorylase (E.C. 2.4.1.7) and phosphoglucomutase (PGM, E.C. 5.4.2.2).^255^ The binding of the enzymatic products, fructose and glucose-6-phosphate, to the boronic acid moieties led to the recovery of fluorescence, which allowed the successful HTS of PGM inhibitors and the determination of enzyme kinetics (Figure 29).^255^ Sharrett *et al*. showed that instead of HPTS **93**, aminopyrene trisulfonic acid (APTS) **94** could also be used, which had constant fluorescence intensity from pH 4 to 10 and could be readily decorated with handles for polymerization. They applied it for the continuous detection of glucose (in the mM range) by immobilization of the APTS monomers in hydroxyethyl methacrylate hydrogels.^256^

Differentiation between diol-containing compounds remains a major challenge in the development of boronic acid-based probes. Choi *et al*. sought a rapid method to screen for activity in P450 mutants and, among other approaches, conducted preliminary experiments with probe **82**. They found the assay incompatible with solid agar plates and not fast or sensitive enough to detect *cis*-diols in the nM range in solution. High noise from other diol-containing compounds caused many false-positive identifications of mutants.^23^

One approach to achieve strong binding affinity and high selectivity is to use probes with multiple recognition elements, for instance, by including several boronic acid moieties or other functional groups.^231,257,258^ Using this principle, fluorescent probes for the detection of diol-containing molecules, such as glucosamine,^259^ digoxin,^260^ rifampicin,^260^ erythromycin,^260^ and d-glucose,^261^ have been developed.

As previously mentioned, chiral diols are part of many intermediates, pharmaceuticals, and catalysts. Fast and straightforward (probe-based) methods to determine the enantiomeric or diastereomeric excess (*ee*, *de*) and the absolute configuration are desirable. Shcherbakova *et al*. developed a technique that enabled the determination of *ee*/*de*, absolute configuration, and yield of 1,2- or 1,3-diols in high throughput. The assay is based on the self-assembly of 1,2- or 1,3-diols with enantiopure tryptophan methyl ester (or tryptophanol, **95**) and 2-formylphenylboronic acid (2-FPBA, **96**), resulting in diastereomeric boronates that exhibit different fluorogenic properties depending on the absolute configuration. This approach was applied in determining the optical purity of chiral diols such as atorvastatin.^262,263^

### 8.2 Oxidative Cleavage

**Figure 30:**
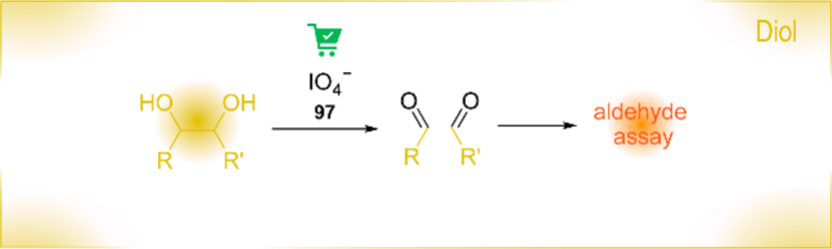
Selective detection of diols based on the cleavage reaction by metaperiodate **97** and subsequent application of aldehyde sensing methodology (for aldehyde-selective probes, see section 9).

**Scheme 9:**
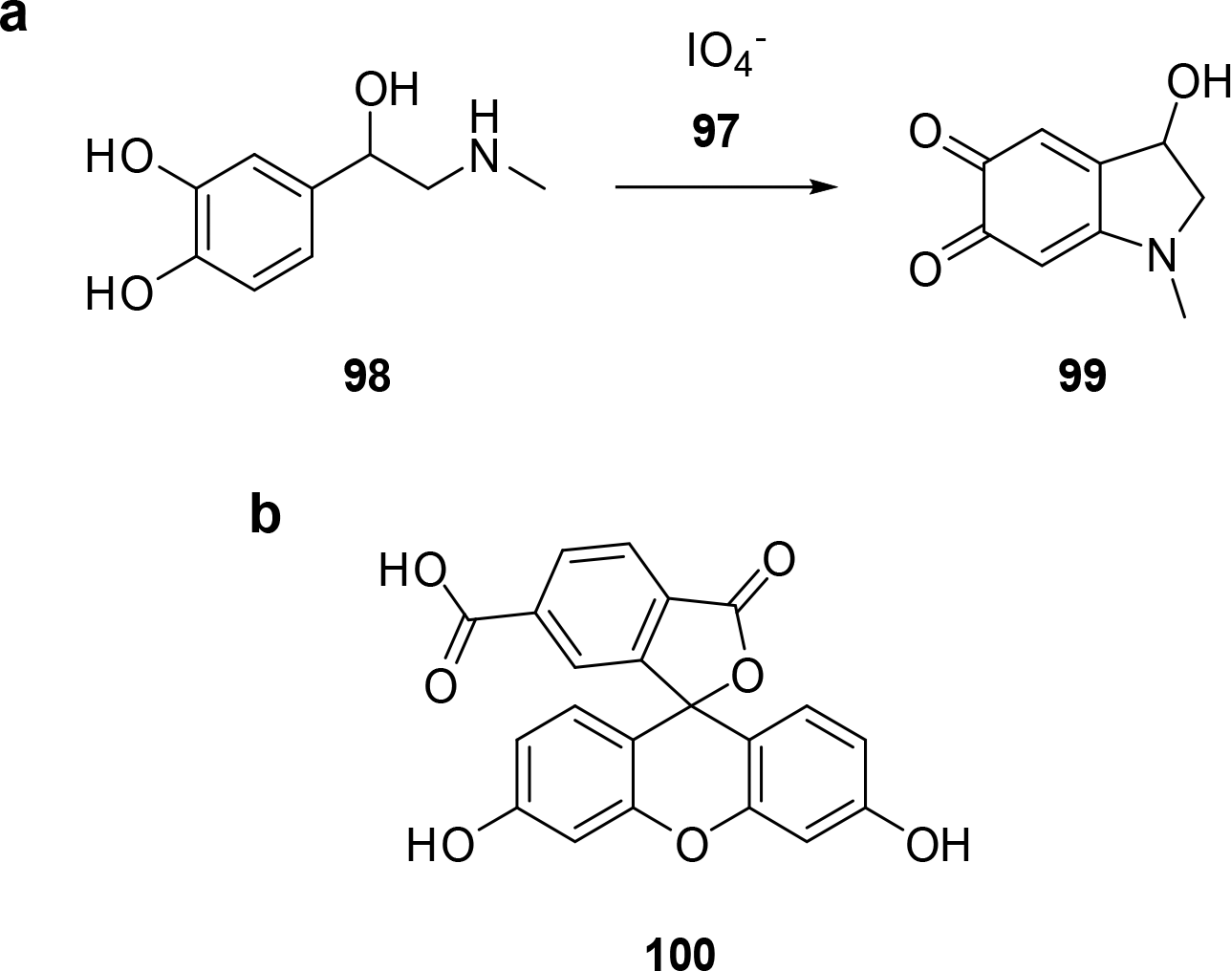
(a) Oxidation of Adrenaline **98** by Metaperiodate **97**, Forming Red-Colored Adrenochrome **99**, Which Allows for Determination of Excess **97** (Back Titration of Diol);^265^ (b) Structure of Carboxyfluorescein, Which Can Also Be Used as a **97** Indicator; The Mechanism for Its Oxidation Is Not Known

An indirect approach to detect diols is converting them to carbonyl compounds by oxidative cleavage with metaperiodate **97** and subsequently using a carbonyl-specific assay (Figure 30) (*cf*. section 9). The reaction of 1,2-diols with periodic acid or periodates leads to the formation of a cyclic intermediate, which subsequently collapses to give ketones and/or aldehydes.^219^ Doderer *et al*. used imine-based probe **123** to detect the formed aldehydes colorimetrically. With this approach, they determined the activity of epoxide hydrolase from *Streptomyces antibioticus* Tü4 toward styrene oxide and 1-butene oxide.^264^ Other assays are based on cleavage using stoichiometric amounts of periodate and subsequent titration of the remaining unreacted oxidant with chromogenic or fluorogenic reagents.^265,266^ Wahler and Reymond demonstrated that adrenaline **98** could be used this way: it reacted rapidly with the excess of sodium periodate, forming the red dye adrenochrome **99** (Scheme 9a).^265^

Kirschner and Bornscheuer used the above approach in the screening of Baeyer–Villiger monooxygenase mutants for enhanced activity and enantioselectivity. The reactions formed acetic acid esters, which were hydrolyzed by an esterase, yielding a 1,2-diol that was detected.^267^ Apart from 1,2 diols, this so-called adrenaline test can also be applied with 1,2-amino alcohols, 1,2-diamines, or α-hydroxy ketones. It has been used to assay lipases, esterases, phytases, and epoxide hydrolases.^265,268,269^

Titration of residual periodate was also efficiently performed with carboxyfluorescein **100** (Scheme 9b), which exhibited a decrease in fluorescence upon its reaction with residual periodate.^270^ Sialic acids in glycoproteins can also be detected and quantified using periodate, as shown by Matsuno and Suzuki. They converted the released formaldehyde to a fluorescent dihydropyridine derivative with acetoacetanilide **135** (cf. section 9.4) and ammonia. *N*-acetylneuraminic acid, *N*-acetylneuraminyllactose, sorbitol, and galactitol all gave enhanced fluorescence, but d-glucose and methyl α-d-glucoside did not; this effect was explained by their slow oxidation at 0 °C.^271^

**Figure 31:**
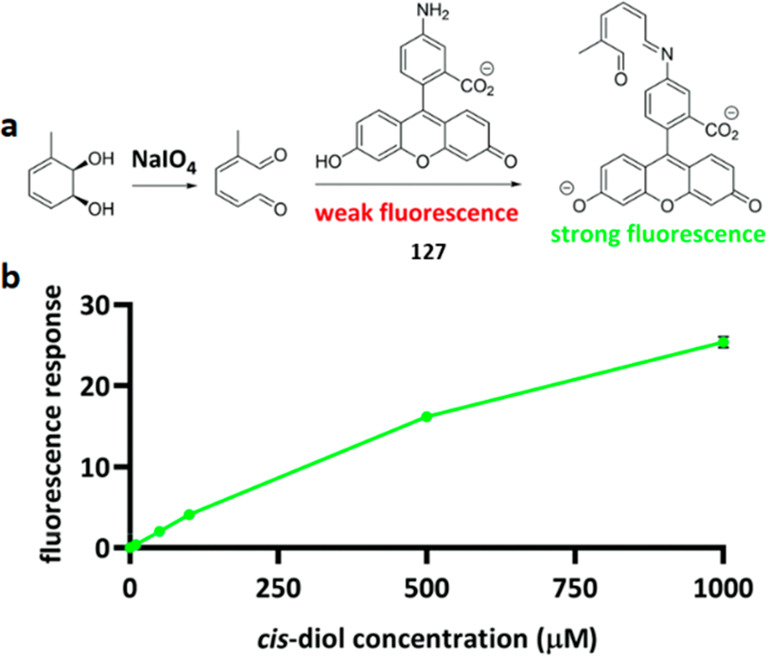
(a) Coupled reactions employed by the fluorescence-based assay system for the detection of *cis*-diol metabolites using fluoresceinamine **127**. (b) Concentration-dependent fluorescence response of the assay to the presence of *cis*-diol metabolites. All studies were performed in triplicate; all measurements demonstrated standard deviation <5%; fluorescence responses normalized to negative control ([*I* – *I*_0_]/*I*_0_). Reproduced with permission from ref (272). Copyright 2021 The Royal Society of Chemistry.

Recently, Preston-Herrera *et al*. developed a HTS assay for Rieske dioxygenase activity. These enzymes catalyze the stereo- and regioselective dihydroxylation of aromatic compounds. The assay was based on oxidizing enzymatically formed *cis*-diols to the dialdehydes with sodium metaperiodate and the subsequent reaction with fluoresceinamine **127** (cf. section 9.2), which led to a fluorescence enhancement at 520 nm (Figure 31). It was applied in HTS of toluene dioxygenase variants to broaden their substrate scope.^272^

## 9 Chromo- and Fluorogenic Probes for Aldehydes

Aldehydes play an essential role in many areas of modern civilization, including their widespread use as food and flavor ingredients (*e.g*., nutty, fatty, fruity, or grassy notes), as synthetic precursors for fine chemicals and pharmaceuticals, as well as their use as valuable building blocks for the synthesis of macromolecules in the rubber and plastics industry.^675,676^ Although aldehydes are highly reactive and often toxic, they are ubiquitous as metabolic intermediates of natural compounds, drugs, and xenobiotics. Volatile aldehydes, such as formaldehyde, acetaldehyde, and acrolein, are prevalent in the exhaust of internal combustion engines, in tobacco smoke, and as industrial pollutants.^637,677^

**Figure 32:**
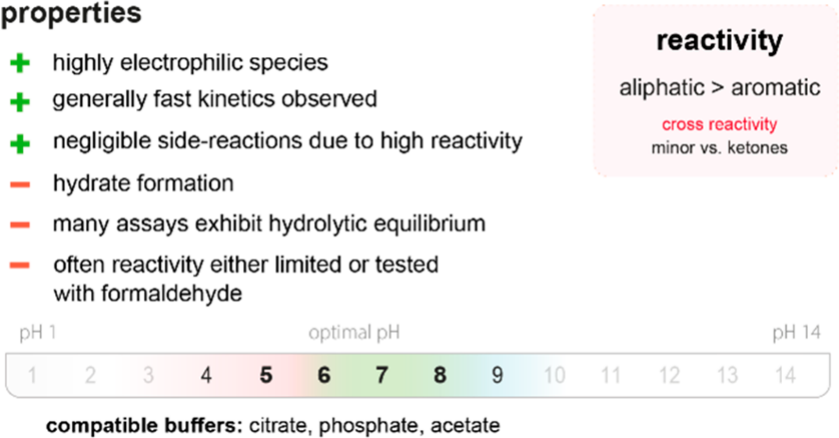
(top) Summary of general properties of aldehydes, reactivity trends toward aldehyde-selective probes, as well as expected cross-reactivity during screenings. (bottom) Compatible pH range and buffers in reported aldehyde-selective assays. Bold numbers indicate the most commonly used pH values.

**Scheme 10:**
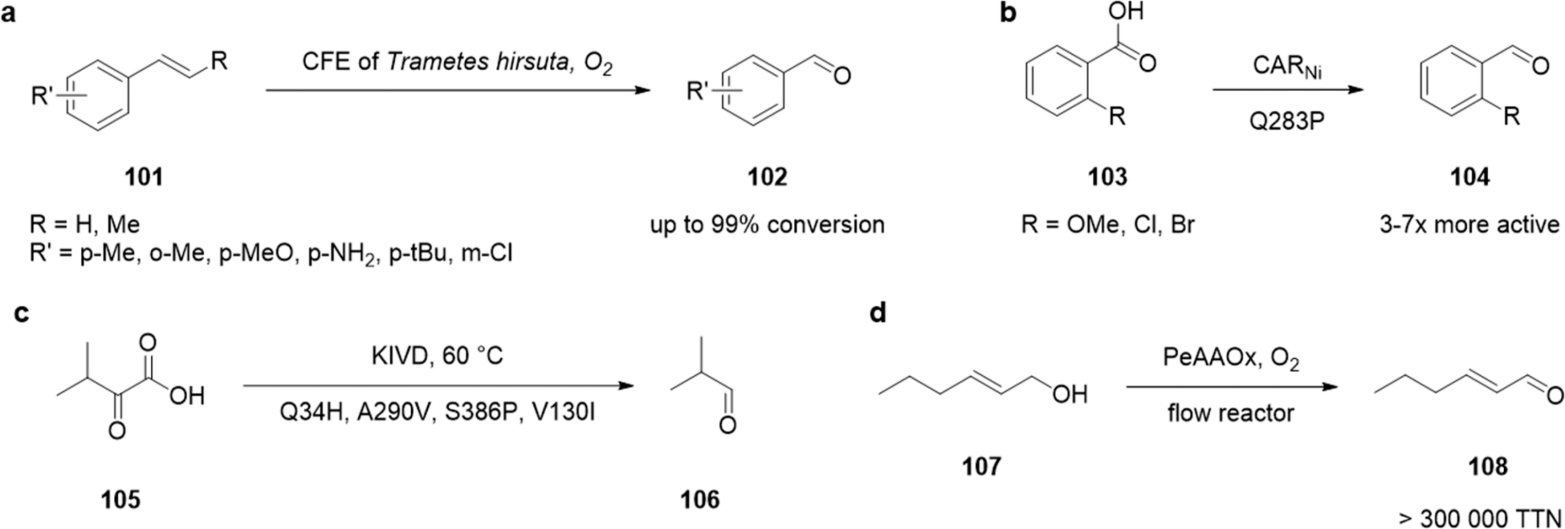
Selection of Enzymatic Transformations for the Production of Aldehyde-Containing Products

Most microorganisms use aldo-keto reductases (AKRs) or alcohol dehydrogenases (ADHs) to eliminate aldehydes, limit their accumulation, and reduce their toxicity.^678^ This homeostatic machinery complicates the biocatalytic synthesis of aldehydes in whole cells. Nevertheless, the drawbacks of typical approaches of organic synthesis to make aldehydes (toxicity of metals; additional steps required for reduction to alcohols or protection; unforgiving, narrow reaction parameters) have motivated the development of enzymatic transformations in recent years. By repurposing natural enzymatic pathways, a broad set of biocatalysts has been identified and applied in the biotransformation of suitable substrates to aldehydes (Scheme 10). These pathways include reduction of carboxylic acids by carboxylic acid reductases (CAR),^273^ decarboxylation of α-ketoacids by decarboxylases (KDC),^274^ reduction of activated esters by acyl-ACP reductases or acyl-CoA reductases,^275^ or the well-studied oxidation of alcohols by alcohol dehydrogenases (ADH)^276^ and alcohol oxidases (AO).^277^ These biocatalysts now rival classic methods from organic chemistry in their performance, convenience, and safety.

While screening for activity in allylic oxidation, the group of Kroutil found an alkene oxidase that is capable of the biocatalytic equivalent of ozonolysis, converting substituted styrene derivatives **101** to the corresponding benzaldehydes **102**.^278^ Employing a different biocatalytic route toward aromatic aldehydes, Schwendenwein *et al*. optimized the promiscuous CAR from *Nocardia iowensis* to improve its activity for sterically demanding 2-substituted benzoic acid derivatives **103**.^279^ Screening 6000 mutants using a selective ABAO aldehyde assay (**126**) identified a single-point mutation (Q283P) which led to a 3–7-fold increase in activity toward *o*-substituted substrates.

Soh *et al*. used a directed evolution strategy to optimize the decarboxylation of 2-ketoisovalerate **105** catalyzed by a KDC at 60 °C. Facilitating the reaction at this temperature would enable the production of isobutyraldehyde **106** during the decomposition of cellulose in lignocellulolytic thermophiles. Random mutagenesis combined with further modeling was used to identify a mutant with a 4-fold increased half-life at the desired temperature.^280^

Improvements in catalytic utility of alcohol oxidases were achieved by tackling the problem of poor O_2_ solubility in aqueous media, which limits mass transfer and results in low reaction rates. By running reactions in continuous flow, the group of Hollmann demonstrated the oxidation of *trans*-hex-2-enol **107** to *trans*-hex-2-enal **108** with turnover numbers >3 × 10^5^ using an aryl alcohol oxidase from *Pleurotus eryngii* (PeAAOx).^281^

The high reactivity of aldehydes, higher than many other electrophilic species, makes the detection of these compounds in physiological environments relatively simple. Their reactive nature also means that unwanted reactions occur readily, such as oxidations, reductions, and condensation reactions with cellular material.^679^ Often, their volatility requires fast-reacting probes to achieve efficient detection. Additionally, formation of geminal diols at non-neutral pH needs to be taken into account; this is important, especially for the detection of formaldehyde, which exists predominantly in its hydrated form in water.^680^ Most applied ratiometric assays rely on the reaction of amines or amino-derivatives with aldehydes, forming imines, hydrazones, or oximes. These primary conjugates are often converted further to stable heterocycles *via* intermolecular reactions, irreversible rearrangements, or oxidations to prevent their hydrolysis. Among these conjugates, hydrazones or oximes are inherently more stable due to the inductive effect of the adjacent nitrogen or oxygen atoms.^342^ Because aldehydes are much more reactive than other carbonyls, only minimal cross-reactivity can be expected with ketones, esters, or amide derivatives. Aromatic or sterically demanding aldehydes react more slowly than aliphatic ones; electron-donating effects can additionally reduce their electrophilicity. The low reactivity can be compensated by using highly nucleophilic (electron-rich) reagents, which sometimes are prone to oxidation (Figure 32).

### 9.1 Hydrazone Formation

**Figure 33:**
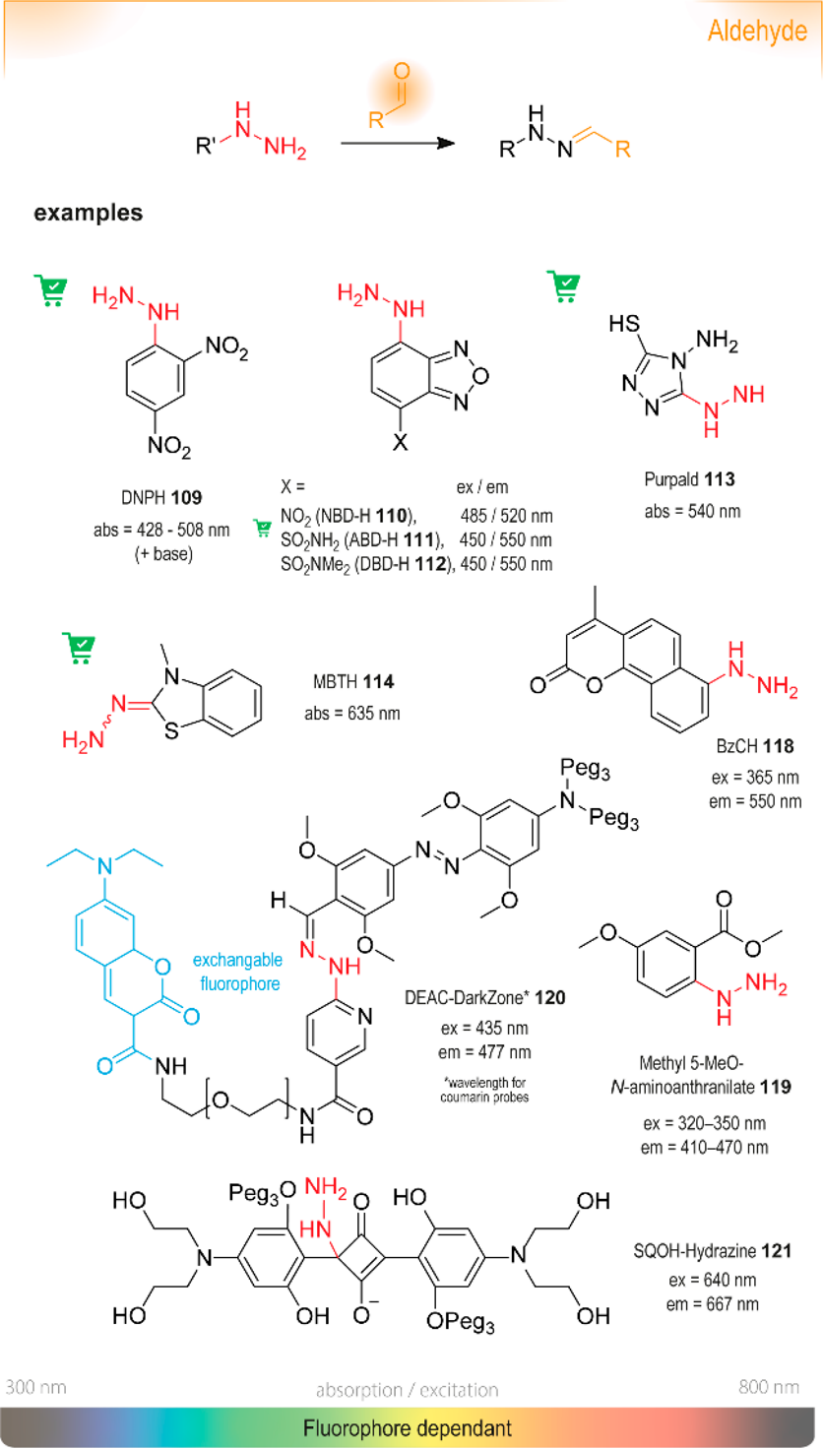
Summary of chromo- and fluorogenic turn-on probes for the selective detection of aldehydes based on the formation of hydrazone adducts. Abs: wavelengths typically used for UV absorbance measurements. Ex or em: wavelengths typically used for excitation and emission in fluorescence measurements. The wavelengths of probes in this section are shown in the strip at the bottom representing the visible spectrum.

The most widely applied method to detect aldehydes is based on the formation of stable hydrazones (Figure 33). Historically, hydrazines were used to identify (potentially liquid) carbonyl species by derivatization to solid hydrazones, which typically have well-defined and thus extensively catalogued melting points.^282^ The drive toward developing fast analytical tools has repurposed these compounds for use in spectroscopic detection systems,^38^ which have continuously evolved since their initial application as TLC staining solutions.

An example of a reagent used for selective carbonyl detection is dinitrophenylhydrazine (DNPH, **109**), also called “Brady’s reagent”. Since its first mention in 1926,^283^ it has been widely used to detect the carbonylation of proteins. It requires a strong acid as a catalyst to form hydrazones, which limits the application of such reagents for detection *in situ*. Unreacted probe has to be separated because of the small Stokes shift.^285^ However, deprotonation of the resulting hydrazones with strong bases typically causes an 80 nm bathochromic shift, separating the signal from background created by the unreacted reagent.^38^ Solutions of **109** are generally prepared in dilute phosphoric acid^286^ or dilute HCl.^287^ This method was used to quantify carbonyls in oxidized proteins^286^ and to determine the concentration of benzaldehyde and phenylpyruvate in the optimization of the stereoselectivity of a threonine aldolase.^288^ The reagent was also used in a HTS of variants of mandelate racemase to detect oxidized α-hydroxy acids.^287,289^

The use of **109** is limited to absorbance in the UV, so its fluorescent benzoxadiazole (BD) analogues have been widely adopted as fluorogenic reporters for aldehydes and ketones;^290^ they emerged from the fluorogenic amino-selective NBD-Cl **12**.^291,292^ Substituting the electron-withdrawing nitro-group with more soluble sulfonamides (ABD-H **111**, DBD-H **112**) increases reaction rates with carbonyl compounds while maintaining a similar spectral profile. The cleanest reactions with aldehydes were found with NBD-H **110** (commercially available as hydrazine adduct), whereas the sulfonamides reacted more cleanly with ketones.

Generally, the substituted hydrazines presented here are synthesized from commercially available chlorides or fluorides by nucleophilic aromatic substitution with hydrazine. Similar to **109**, assays are typically performed in acidic^290,291^ solution or with a high excess of the reagent under physiological conditions (pH 7.5 in PBS buffer).^293^

**Figure 34:**
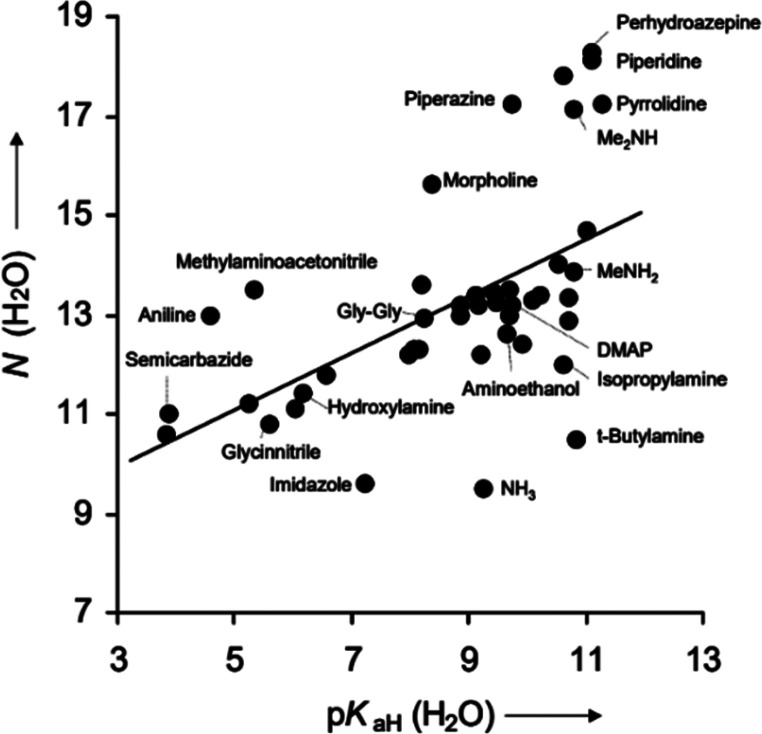
Plot of the nucleophilicity parameters N of amines *versus* p*K*_aH_ in water.^298^ Reproduced with permission from refs. (299 and 300). Copyright 2007 American Chemical Society.

Anilines are known to catalyze the conjugation:^294−296^ their low p*K*_a_ values (Figure 34) make them nucleophilic even at pH 5–7, forming highly reactive imines as intermediates and thus activating the carbonyl group. These species undergo fast conjugations at physiological pH. Aniline derivatives bearing hydrogen-bond donors in *o*-position were found to additionally boost the reaction by iminium formation. These “catalysts” are typically used in excess to the applied probes to compensate for the slow kinetics caused by the poor solubility and reactivity of assay probes. Furthermore, a high salt concentration was found to be beneficial for the conjugation of such systems.^297^

NBD-H **110** is the most prominent representative of this class of probes. It has been used in HTS to evaluate esterase reactions with vinyl esters as acyl donors in organic solvents.^293^ The release of acetaldehyde upon esterification was tracked efficiently using this assay. The sulfonamide analogues (**111**, **112**) have yet to be used in HTS. Nevertheless, they have seen use in the chromatographic separation of unsaturated aliphatic aldehydes with low molecular mass.^162,301^

**Scheme 11:**
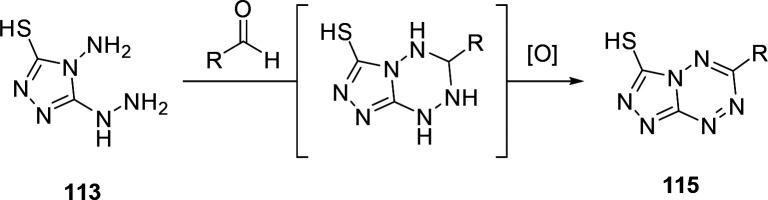
Sensing Mechanism of Purpald **113***via* the Intermediate Formation of Cyclic Tetrazinanes and Subsequent Oxidation to Fully Aromatized Violet-Colored Tetrazines **115**

A long known reagent that is still in regular use is 4-amino-3-hydrazino-5-mercapto-1,2,4-triazole (Purpald, **113**).^302,303^ This chromogenic probe reacts with aliphatic and aromatic aldehydes at room temperature to form tetrazinanes, which quickly oxidize to the violet-colored tetrazines **115** (Scheme 11). The coloration requires the oxidation of the tetrazinanes, which can be promoted with H_2_O_2,_ dilute periodate, or atmospheric oxygen. Vigorous shaking of the sample to enable aeration is often sufficient.^304^ The assay is performed under strongly basic conditions (*e.g*., 0.5–2 M NaOH).^304,288,305^

Purpald has been extensively used in off-line HTS and protein engineering studies. Lauchli *et al*. used the reagent for the directed evolution of a terpene synthase: Non-natural isoprenoids released methanol upon cyclization by enzymatic catalysis,^192^ which was oxidized to formaldehyde using an alcohol oxidase and subsequently detected using **113** at 550 nm. Another example, from the group of Arnold, used **113** in protein engineering of a P450 monooxygenase.^306^ Directed evolution transformed a known epoxidase from the rhodobacterium *Labrenzia aggregata* (P450_LA1_) into an efficient *anti*-Markovnikov alkene oxidase. The aldehydes produced in the reaction were detected using **113**.

The group of Fraaije engineered an alcohol oxidase from*Phanerochaete chrysosporium* (PcAOX) to enhance the oxidation of glycerol.^307^ The cavity-enlarging mutation F101S, which increased *k*_cat_ to 3 s^–1^, was identified using an assay based on **113**.

**Scheme 12:**
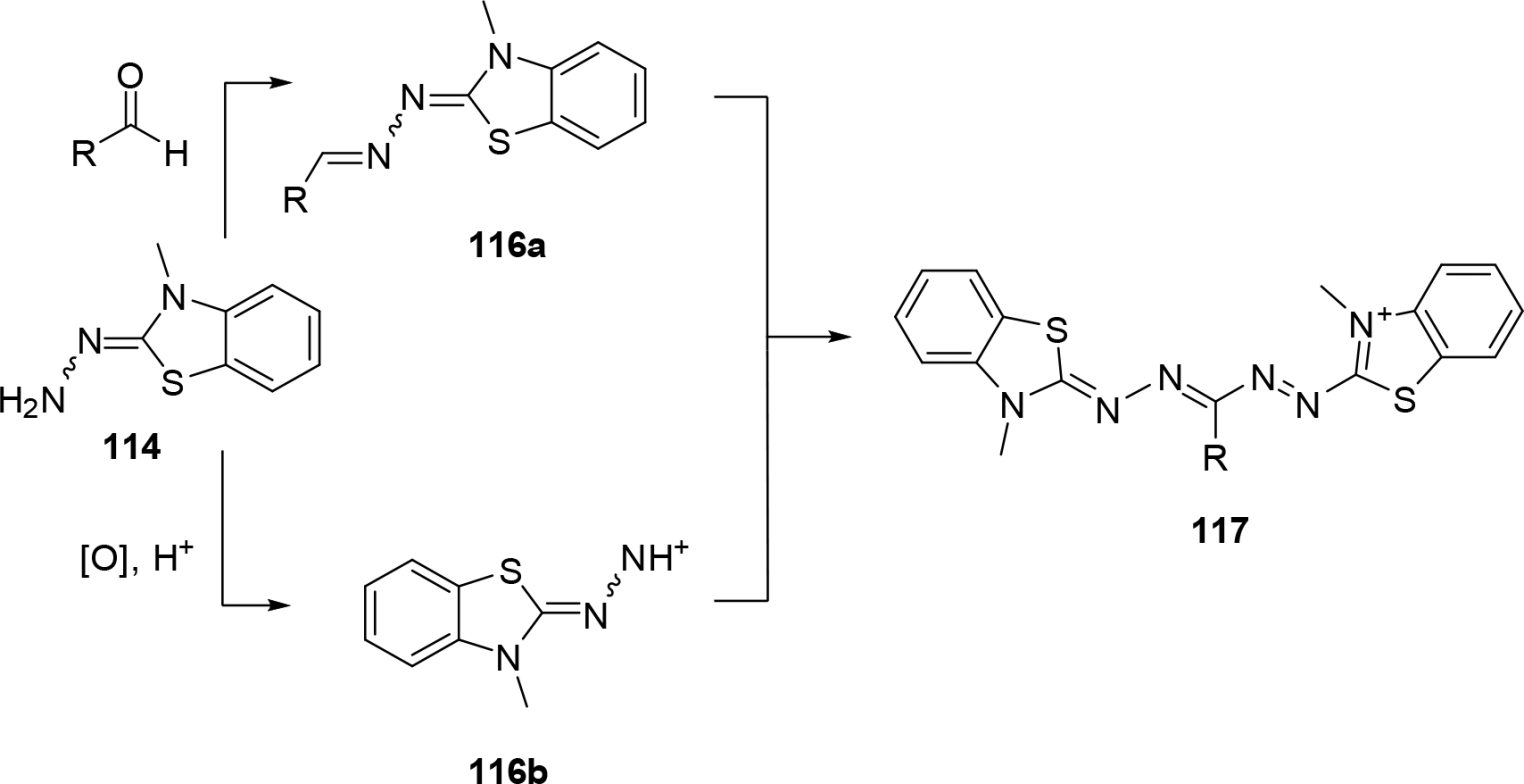
Sensing Mechanism for MBTH **114**

Another widely used hydrazine probe for the detection of aldehydes is 3-methyl-2-benzothiazolone hydrazone (MBTH) **114**.^308^ A mechanistic study revealed that two equivalents of the reagent are required to form the blue, cationic dye: one molecule reacts with the aldehyde (**116a**) and the other is oxidized (**116b**). They further react to form the highly conjugated system **117**, a dark-blue dye with an absorption maximum at 635 nm (Scheme 12).

Concluding from the mechanistic analysis, the chromogenic reaction only happens when there is an excess of **114** that has not reacted with an aldehyde and is available for oxidation. An excess of aldehyde might thus not be detectable.^309^ Contrary to **113**, the reaction of **114** is performed at acidic pH (0.1 M HCl) and with ferric chloride as the oxidant, forming the blue dye. Modifications to the assay include the addition of sulfamic acid, which prevented the emergence of turbidity during the oxidation process.^310^

In a comparison of three chromogenic reagents for the detection of formaldehyde (Purpald **113**, Nash reagent **132** (*cf*. section 9.4), and MBTH **114**), **114** proved to be the only dye that formed stable adducts and prevented further oxidation toward formic acid in the screening of an alcohol oxidase (AO).^305^ Even though **114** has not yet found widespread use in protein engineering, it has been applied to determine the concentration of reducing sugars^311,312^ and of total aldehyde content in fuel ethanol samples.^313^

Recently, the group of Bane synthesized a dye with a benzocoumarin core and a hydrazine handle. This reagent, BzCH **118**, was used for the detection of carbonylation in A549 lung carcinoma cells.^681^ The quantification of the carbonyl content in the cells was performed in HEPES buffer for 4.5 h or in growth medium for 30–60 min at room temperature. Upon reaction with an aldehyde, the emission shifted from 430 to 550 nm due to disaggregation of the fluorophores. The conjugated hydrazones additionally exhibited an exceptionally large Stokes shift (195 nm) with no meaningful overlap between its absorption and emission spectra. The emission of the probe was shown to be stable from pH 4 to 8.

**Figure 35:**
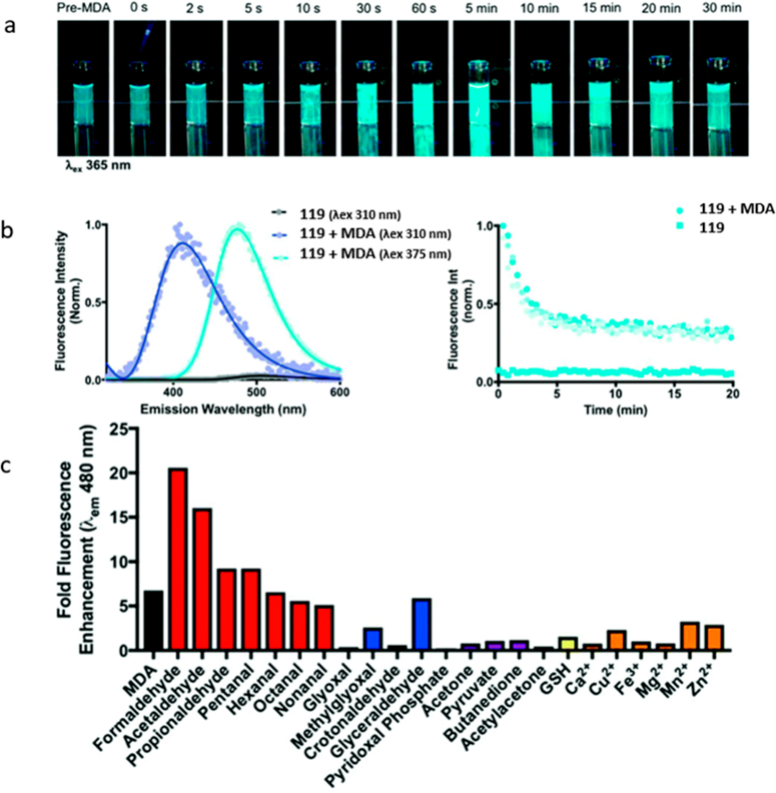
(a) **119** as a fluorogenic probe for the detection of MDA. (a) Malondialdehyde (MDA) was added to a 25 μM PBS solution of **119** (pH 7.2) under illumination at 365 nm and photographed at indicated time points. (b) (left) Emission spectra of 1 μM **119** (black) or **119** after 1 min incubation with 5 equiv MDA at 37 °C and excited at 310 nm (blue) or 375 nm (cyan). (right) Formation and rapid decay of short-lived intermediate formed in the reaction between **119** and MDA followed by fluorescence spectroscopy with λ_ex_/λ_em_ = 375/480 nm. Kinetics of 25 μM **119** only (squares) and **119** + MDA (circles) are shown, with three replicates of **119** + MDA shown (light, medium, and dark cyan). (c) **119** shows broad specificity to aliphatic aldehydes. The enhancement of fluorescence relative to **119** only was recorded with λ_ex_/λ_em_ 375/480 nm following 1 min of incubation of 1 μM solutions of **119** in PBS (pH 7.2) with 5 equiv of MDA (black bar), simple aliphatic aldehydes (red bars), dialdehydes, and complex aldehydes (blue bars), ketones (purple bars), glutathione (yellow bar), and biogenic metal cations (orange bars). Reproduced with permission from ref (314). Copyright 2019 The Royal Society of Chemistry.

Methyl 5-MeO-*N*-aminoanthranilate **119** is a fluorogenic probe for aldehydes developed by Suchý *et al*., based on a modified anthranilic acid. It was used to measure the concentration of aldehydes in cells.^314^ The reaction with their test substate (malondialdehyde) immediately led to the formation of a blue-green fluorescent species (λ_em_ = 480 nm), followed by a rapid decay of the signal (50% within 5 min; Figure 35). Still, initial enhancement of fluorescence with aliphatic aldehydes was between 5- and 20-fold.

In PBS buffer, the reagent cyclized to blue fluorescent indazoles, which would not react any further; no such reaction occurred in DMSO. Because the desired reaction is much faster than the side reaction in PBS (*e.g*., 10-fold faster with hexanal), the probe could still be used to study the peroxidation of lipids in HEK293 cells incubated with diethyl maleate.

The group of Kool developed the fluorogenic hydrazine derivative “DarkZone” **120**, preloaded with an aromatic azoaldehyde that quenches fluorescence.^315^ Upon reaction with the target aldehyde, the quencher is liberated and the desired turn-on is achieved. A variety of other fluorogenic dyes could be incorporated into the “DarkZone” approach (*e.g.*, BODIPY, DEAC, Cy3, fluorescein, TAMRA). Only reactions with aliphatic aldehydes produced a strong signal because bisaryl hydrazone products (similarly to the structure of the original probe) also caused a quenching of the fluorescence emission. To facilitate conjugation at physiological pH, the authors tested various anilines and identified 5-methoxyanthranilic acid as a nontoxic and efficient catalyst for the transformation. Those tests were performed with a large excess of catalyst and analyte (dye/aldehyde/aniline catalyst in a ratio of 1:4000:10000) in phosphate-buffered systems at pH 7, resulting in an enhancement of up to 30-fold. To demonstrate the applicability of their system, aldehyde production from ethanol in K562 cells was monitored using their acetylated fluorescein analogue, which resulted in a 2.5-fold increase in fluorescence after incubation of the cells for 24 h.

**Figure 36:**
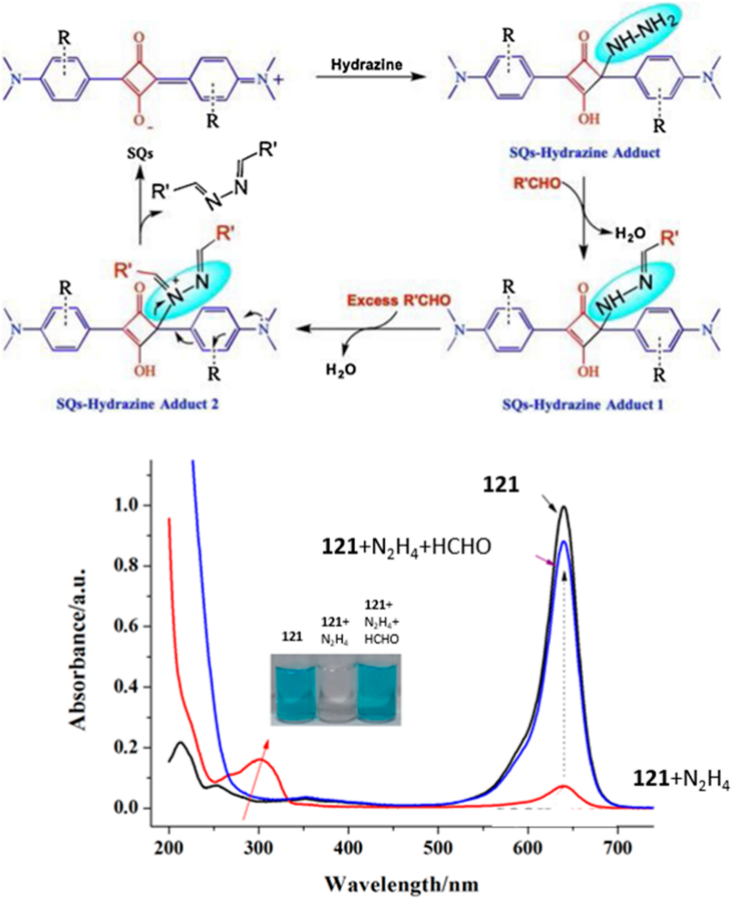
(top) Scheme of the reaction mechanism of colorimetric and fluorescent detection of aldehydes based on the SQs–N_2_H_4_ adduct **121**. (bottom) The changes in UV–vis absorption of dye **121** (6.0 mM) upon addition of N_2_H_4_ (3.0 mM) and subsequent addition of formaldehyde (10.0 mM). Reproduced with permission from ref (316). Copyright 2019 Elsevier BV.

Liu *et al*.^316^ developed hydrazine dyes based on the squaraine (SQ) scaffold, such as **121**, which have narrow absorbance and fluorescence spectra in the near-infrared region.^317^ The cyclobutene ring is electron-deficient and thus susceptible to nucleophilic attack; this property is extensively used in colorimetric and fluorescent probes for the detection of nucleophiles.^318^ The loss of π-conjugation upon the addition of various amines or hydrazine bleaches the blue color. Subsequent addition of aliphatic or aromatic aldehydes reverts the optical properties of the parent SQs, most prominently observed with the SQ–hydrazine system (Figure 36). Using the water-soluble SQOH–hydrazine **121**, addition of an excess of formaldehyde was proven to completely scavenge the hydrazine, accompanied by a 23-fold increase in absorption and 86% recovery of the emission intensity of the original squaric acid dye in water.

### 9.2 Imine Formation (and Trapping)

**Figure 37:**
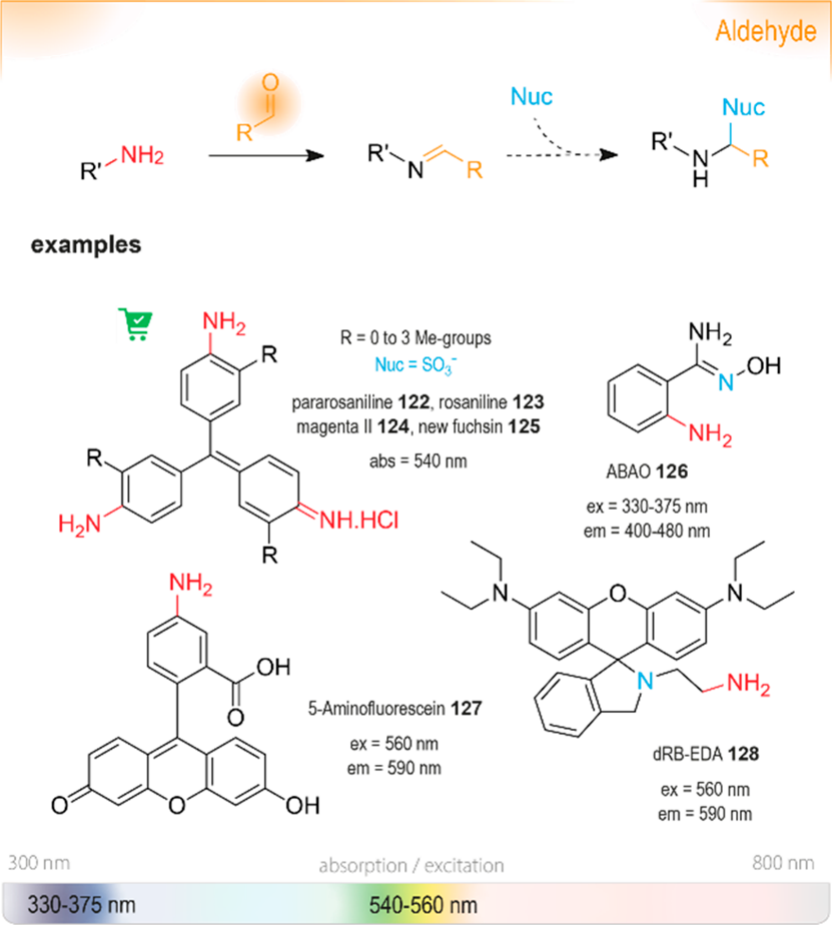
Summary of chromo- and fluorogenic turn-on probes for the selective detection of aldehydes based on the formation of imines (Schiff’s bases). Subsequent entrapment of these electrophilic imines by intra- or intermolecular nucleophiles makes the reaction irreversible. Abs: wavelengths typically used for UV Absorbance measurements. Ex or em: wavelengths typically used for excitation and emission in fluorescence measurements. The wavelengths of probes in this section are shown in the strip at the bottom representing the visible spectrum.

This group of reagents uses changes of spectral properties upon formation of Schiff’s bases from amines with aldehydes. Imines are less stable in water than hydrazones. Therefore, most of these probes irreversibly entrap the initially formed imine with intra- or intermolecular nucleophiles (Figure 37). The earliest examples of this class of compounds include pararosaniline **122**, its methylated analogues rosaniline (also Schiff’s reagent, **123**), magenta II **124**, and “new fuchsin” **125**.

Methylation has a large effect on the absorption spectra after conjugation to formaldehyde.^319^ Robins *et al.* used NMR spectroscopy to study the colored species and the kinetics of its formation.^320^ They found that “careful calibration is required” to obtain accurate results when quantifying aldehydes, with a complicated dependency on the concentrations of the dye, the sulfurous acid (or SO_2_) used as the nucleophile, and the aldehyde.

**Figure 38:**
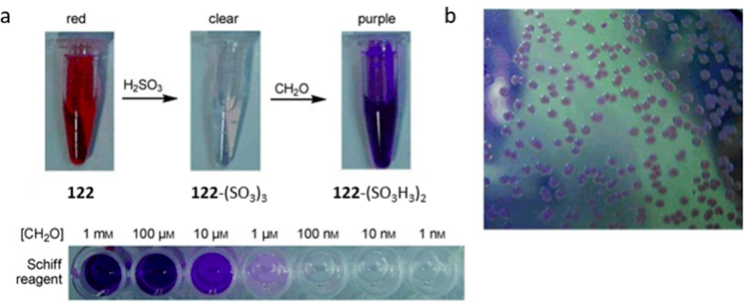
(a) The color of the aldehyde-sensing probe **122** changes to purple when treated with formaldehyde (detection limit: μM concentrations of formaldehyde in the *in vitro* system). (b) Color changes on solid agar plate from *O*-dealkylation activity. Aldehyde appears to diffuse out of the cells; cell color changes are observed in the presence of **122**. Reproduced with permission from ref (23). Copyright 2013 Wiley-VCH.

Despite these issues, the reagent has been used to determine *o*-dealkylation activity of 4′,7-dihydroxyisoflavone (daidzein) hydroxylase by detection of the purple formaldehyde adduct using an *in vivo* assay on LB agar plates (Figure 38).^23^

**Figure 39:**
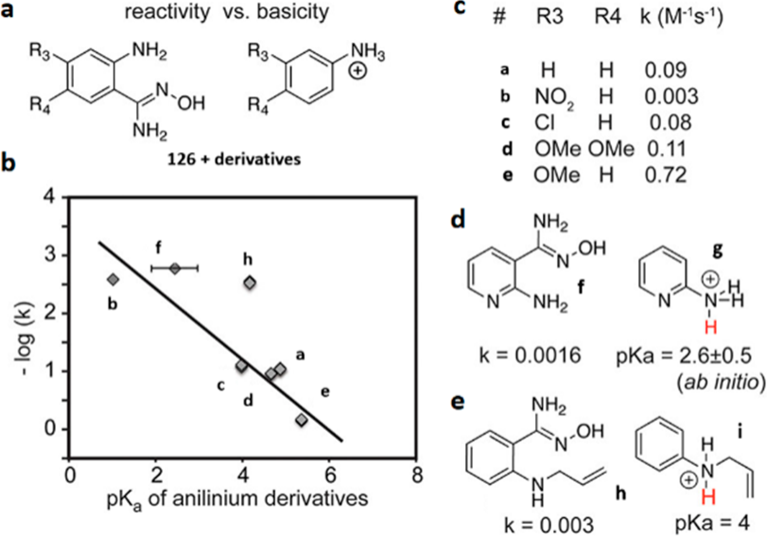
Relationship between the measured reaction rates and p*K*_a_ of the corresponding protonated anilines for seven ABAO analogues **126a–g**. (i) Structures of ABAOs **126** and corresponding protonated anilines. (ii) Scatter plot showing relationship between log *k* and p*K*_a_. (iii) Reaction rates measured by NMR in CD_3_COONa buffer (100 mM) at pD 4.5.(iv) Structures and reaction rates for **126f** and **126g**. Basicity of aniline nitrogen in 2-aminopyridine / **126f** was estimated using *ab initio* calculations. Reproduced with permission from ref (321). Copyright 2014 American Chemical Society.

Amino benzamidoxime (ABAO, **126**) reagents are another class of probes using an initial Schiff base formation for the selective detection of carbonyl compounds. According to the postulated mechanism, the intermediately formed imine is trapped by the nitrogen of the oxime. The resulting quinazoline oxides show a substantial shift in absorbance from 360 to 405 nm and strong fluorescence at 490 nm. Kitov *et al*.^321^ were the first to report an in-depth analysis of these detection systems and found a linear free energy relationship (LFER) with the p*K*_a_ of the respective anilinium species (Figure 39). A *p*-nitro substituent led to a loss of activity while a *p*-methoxy group increased the conjugation speed.

Rudroff *et al*. used ABAO for a systematic mutation study of a carboxylic acid reductase from *Nocardia iowensis* (CAR_*NI*_).^279,322^ Using the 3-methoxy substituted **MeO-126** reagent as a chromogenic aldehyde probe, 6000 mutants of CAR_NI_ mutants created using error-prone PCR (epPCR) were screened for improved activity with a panel of 20 aromatic aldehydes. The best mutant (Q283P) had a 9-fold improvement in *K*_M_ and worked even with typically unreactive *o*-substituted benzoic acids. In a similar fashion, the ABAO assay was used in screening a library of 598 clones of *Mycobacterium marinum* (CAR_MM_) to improve the production of piperonal in *Pichia pastoris*.^323^ The assay is typically performed under acidic conditions (pH 4.5) and was also shown to work at physiological pH, but with lower activity.^324^

Xing *et al*. demonstrated the use of 5-aminofluorescein **127** as potent fluorogenic probe for the detection of a broad range of aliphatic aldehydes.^325^ Using the restored fluorescence emission of the dye upon Schiff base formation, they used **127** to detect microbial oxidation of primary alcohols by the ADH NCIMB 621 from *Gluconobacter oxidans* at pH 6.0 at mM substrate loadings. The detection was improved by using a biphasic mixture of water and isooctane, which accumulated hydrophobic aldehydes in the organic phase. Building upon these findings, Preston-Herrera *et al*. applied probe **127** in a HTS assay for activity of a Rieske toluene dioxygenase.^272^ They converted the *cis*-diol metabolites to the corresponding dialdehydes by oxidation with sodium metaperiodate, followed by detection of the dialdehydes with **127** (*cf*. section 8) (Figure 31). The authors showed that Tris buffer was detrimental for the oxidation due to possible reaction of metaperiodate with the hydroxy groups of the buffer. Reactions performed in HEPES or phosphate buffers worked well, with the highest rates at pH 6.5, which was attributed to acid catalysis of the imine formation. A 10–30-fold enhancement of fluorescence emission was observed using the optimal conditions after 5 h. They then used the assay to evaluate mutants from a site-saturation mutagenesis, identifying the critical active site residue L272 and subsequently applying the assay for the screening of a broad range of toluene derivatives.

**Scheme 13:**
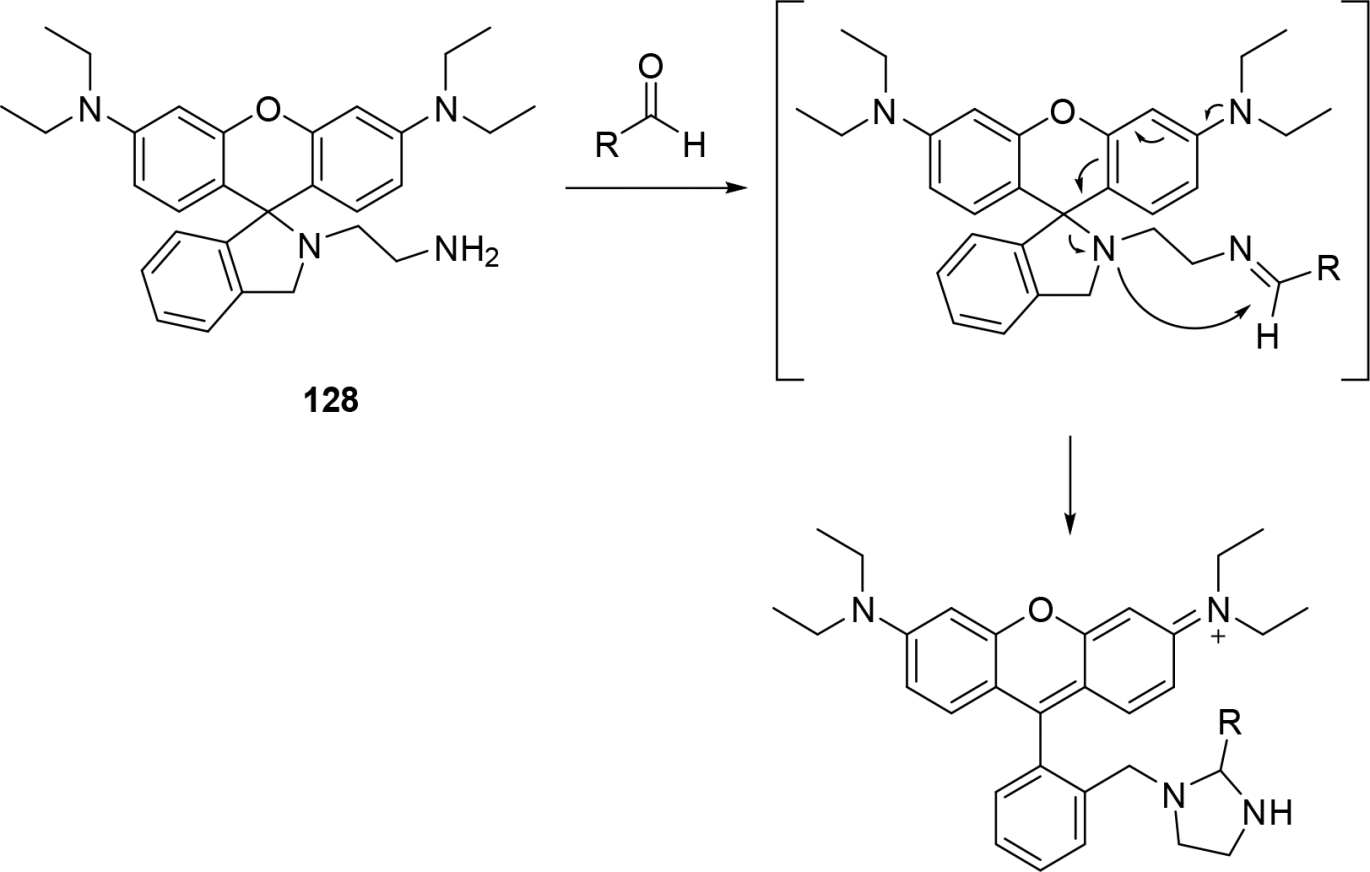
Sensing Mechanism for dRB-EDA **128**: Schiff’s Base Formation with Formaldehyde Triggers the Ring-Opening of the Deoxylactam Moiety

**Figure 40:**
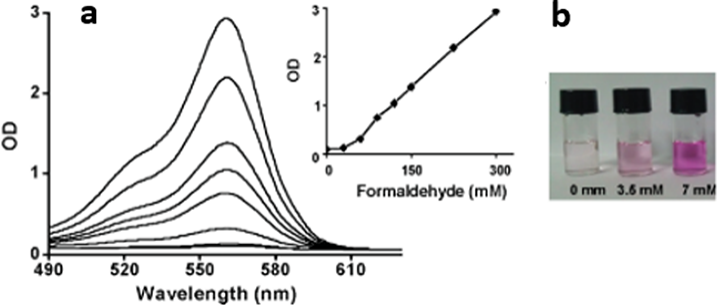
Chromogenic detection of formaldehyde with **128**. (a) UV–absorption spectra of **128** in the presence of formaldehyde (concentration: 0, 3.5, 7, 10.5, 14, 17.5, and 35 mM, from bottom to top). The inset shows the titration curve by absorbance at 560 nm. (b) Visual detection of formaldehyde (0–7 mM as indicated) with **128** in DMF after incubation at room temperature for 90 min. Reproduced with permission from ref (326). Copyright 2011 The Royal Society of Chemistry.

Li *et al.* recently reported the development of the rhodamine derivative dRB-EDA **128**, which undergoes ring-opening of its deoxylactam moiety upon reaction with an aldehyde (Scheme 13).^326^ Cleavage of the C–N bond restores conjugation and causes an increase in fluorescence (only shown in DMF). Solutions of **128** (1 mg mL^–1^) exposed to low mM concentrations of formaldehyde showed a strong increase in absorption at 560 nm and fluorescence at 590 nm after 90 min (Figure 40).

The detection of simple aliphatic and aromatic aldehydes, such as 4-hydroxybenzaldehyde and hexanaldehyde, was shown. The assay was furthermore applied for the staining of oxidized sialoproteins on the cell surface of L929 cells, which were then analyzed by confocal fluorescence microscopy.

### 9.3 Imidazole Formation

**Figure 41:**
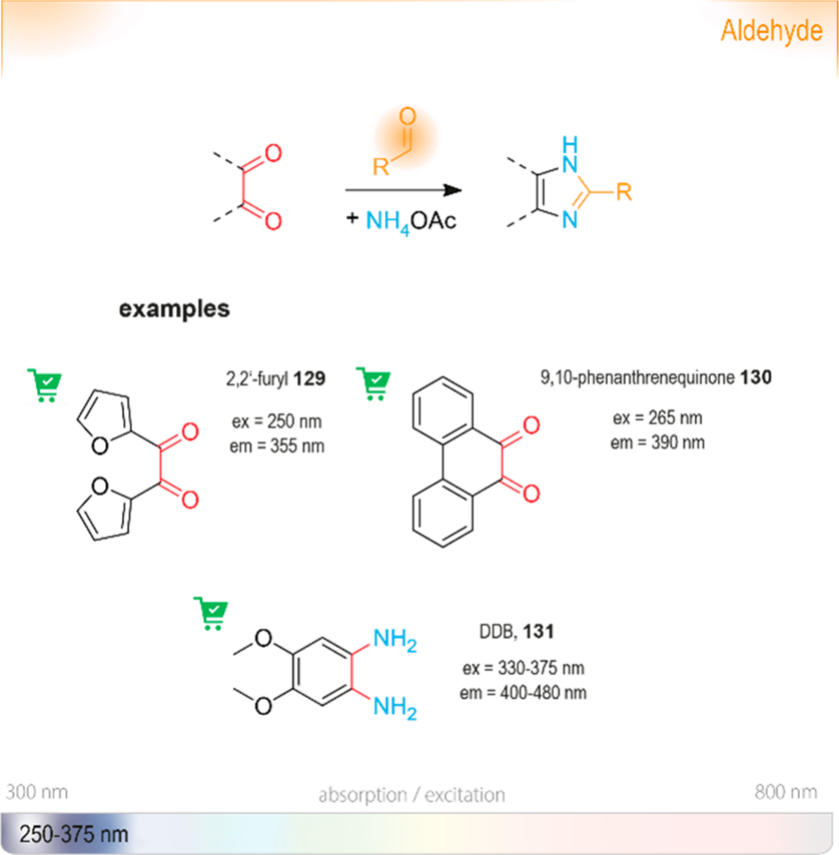
Summary of fluorogenic turn-on probes for the selective detection of aldehydes based on the formation of imidazole structures. Ex or em: wavelengths typically used for excitation and emission in fluorescence measurements. The wavelengths of probes in this section are shown in the strip at the bottom representing the visible spectrum.

A frequent design for sensitive assays uses the formation of stable heterocycles that irreversibly trap the analyte while concomitantly enlarging a delocalized electron system. This method has been applied for the detection of aldehydes using a combination of 1,2-diketones and suitable ammonia donors, forming imidazoles (Figure 41). Variations of the long known colored 2,4,5-triphenylimidazole (trivial name: lophine) formed by the condensation of benzil, benzaldehyde, and ammonia^327^ have been analyzed as potential chemiluminogens.^328^ Two particularly promising derivatives emerged from these studies: 2,2′-furil **129** and 9,10-phenanthrenequinone **130**. Both probes were used as derivatization reagents, with sensitivity down to the nM range, for the monitoring of aliphatic aldehydes in human serum.^329,330^ Derivatizations typically required incubation at 100 °C for 30 min in concentrated ammonium acetate solutions, making an *in situ* analysis of aldehydes impossible.

The aromatic diamine analogue 1,2-diamino-4,5-dimethoxybenzene (DDB, **131**) has been reported as an effective reagent for the fluorometric detection of aromatic aldehydes,^331^ acrolein,^332^ and methylglyoxal.^333^ Derivatizations either required strongly basic conditions at room temperature or heating to 80 °C in DMSO. Its application *in situ* or in protein engineering has not yet been reported.

### 9.4 1,4-Dihydropyridine Formation

**Figure 42:**
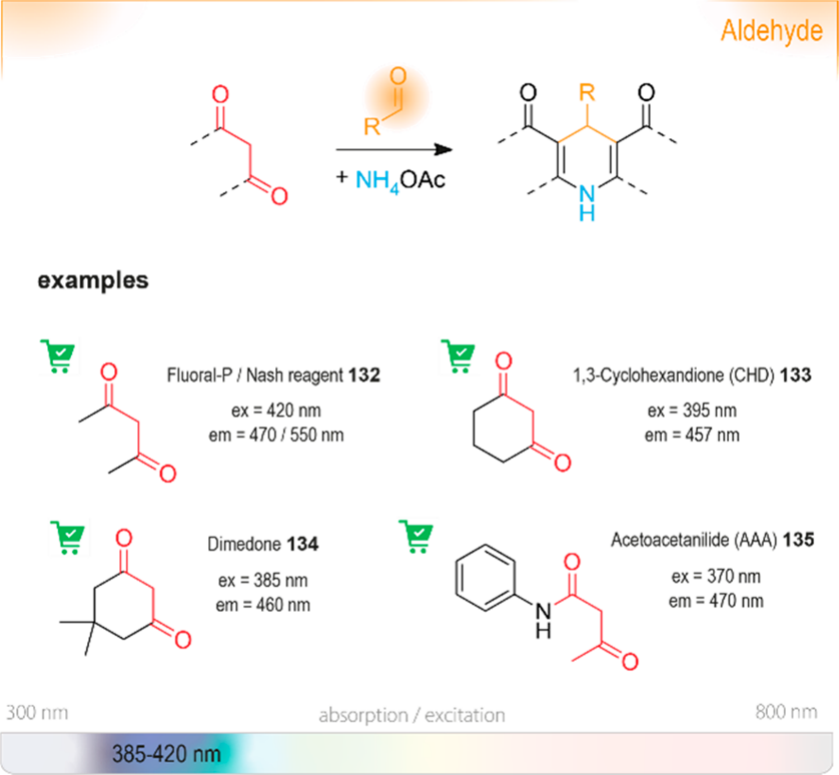
Summary of fluorogenic turn-on probes for the selective detection of aldehydes based on the formation of 1,4-dihydropyridines. Ex or em: wavelengths typically used for excitation and emission in fluorescence measurements. The wavelengths of probes in this section are shown in the strip at the bottom representing the visible spectrum.

Aldehydes and ammonia condense with 1,3-diketones to form chromogenic 1,4-dihydropyridines, a reaction also used for the Hantzsch pyridine synthesis (Figure 42).^334^ The yellow-colored products^335^ often have chromogenic and fluorescent properties, depending on the 1,3-diketone.^336^ The simplest diketone, acetylacetone **132**, can be used, either in its ketone form (“Nash reagent”) or condensed with ammonia (Fluoral-P), for the detection of aldehydes at room temperature in 2 M ammonium acetate buffer at pH 6.^337,338^ Conjugates with formaldehyde showed particularly strong fluorescence, other aliphatic and aromatic aldehydes gave only weak fluorescence in solution (and could only be detected with sensitive detectors).

Sawicki and Carnes hypothesized that the rigidity of the fluorophore might influence signal strength^336^ and tested 1,3-cyclohexanedione **133** and dimedone **134**, resulting in a 100-fold higher fluorescence than with acetylacetone when detecting substituted aldehyde analytes. The increased steric bulk of these sensitive reagents required that the reaction be incubated at 100 °C for 5 min to reach completion. Furthermore, deprotonation of the products using tetraethylammonium hydroxide resulted in a bathochromic shift of approximately 60 nm (λ_em_ = 460 nm, λ_ex_ = 520 nm).

These assays were used to detect aliphatic and aromatic aldehydes in blood plasma or seawater.^339,340^ Anthon and Barrett compared three assays used to detect formaldehyde, all based on the AO-catalyzed oxidation of methanol: the Nash reagent **132**, Purpald **113**, and MBTH **114**. Based on absorbance measurements, the Nash reagent had the lowest sensitivity.^305^

Li *et al*. used acetoacetanilide (AAA, **135**) for the detection of formaldehyde, addressing the problem of low reactivity with sterically demanding diketone reagents. Quantification of μM concentrations was possible at room temperature in 10 min in 4 M ammonium acetate buffer at pH 7.5. The conjugation was selective for formaldehyde; even acetaldehyde did not react with the probe.^341^

### 9.5 Oxime Formation and Fragmentation

**Figure 43:**
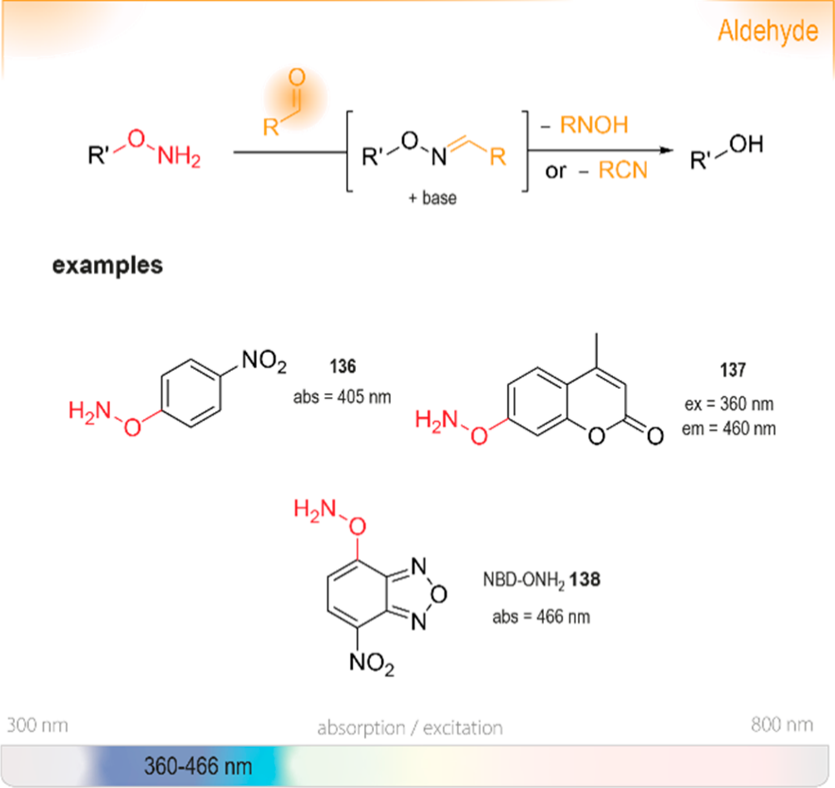
Summary of chromo- and fluorogenic turn-on probes for the selective detection of aldehydes based on the formation of unstable oximes. Fragmentation of these intermediates leads to the liberation of colored phenols. Abs: wavelengths typically used for UV absorbance measurements. Ex or em: wavelengths typically used for excitation and emission in fluorescence measurements. The wavelengths of probes in this section are shown in the strip at the bottom representing the visible spectrum.

Similar to hydrazine reagents, oxyamines react rapidly with carbonyl compounds, forming stable oximes even at low concentrations.^342,343^ These conjugates are stable toward hydrolysis under physiological conditions and have been used for the chemoselective ligation of macromolecular fragments.^344,345^ The group of Reymond observed the fragmentation of the oxime bond at pH > 11, similar to the Kemp reaction:^346^ Base-induced β-elimination of the aldoximes forms a nitrile and a phenolate (Figure 43). Synthetic modification of known nitrophenol or umbelliferone chromophores with the reactive oxyamine moiety enabled the base-induced release of the dyes (**136** and **137**),^347^ and thus spectroscopic or fluorometric measurement of aldehyde conjugation. The same reaction was catalyzed by BSA at neutral pH, where electron-rich aromatic aldehyde and ketone conjugates remained stable.

The unspecific hydrolysis of the oxyamine still proceeded with a significant rate, making the detection of aldehydes problematic. The authors thus opted for a sequential approach: formation of the oxime was stopped by adding acetone in excess and any unreacted probe was thus converted into a stable ketoxime. Only then was the fragmentation of the aldoxime initiated by the addition of base.

**Scheme 14:**
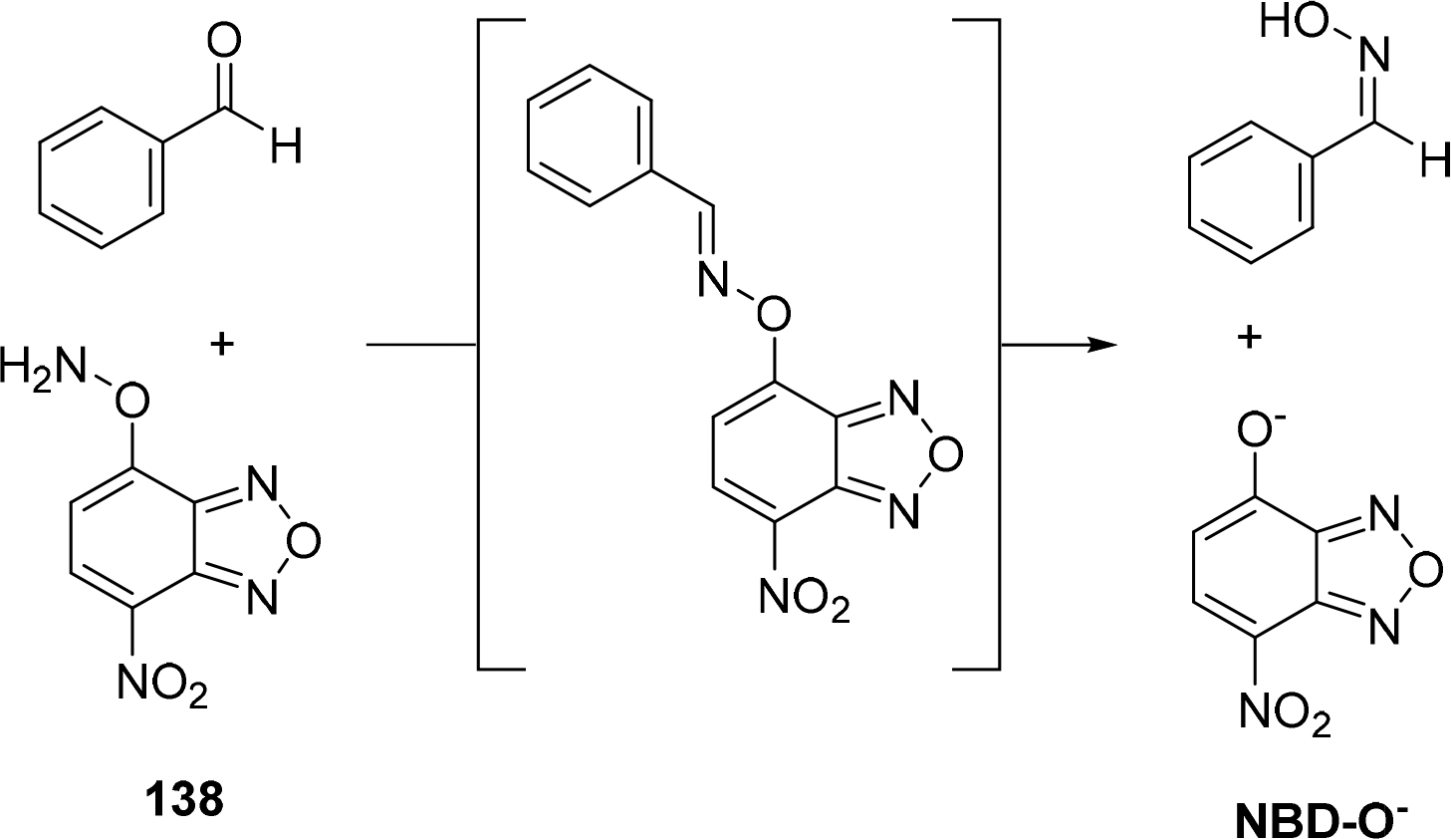
Enzyme-Catalyzed Formation of the **138**–Benzaldehyde Adduct Could Be Efficiently Traced by Spontaneous Decomposition Forming Colored NBD-O^–^

This assay was used in screening for the release of formaldehyde by lipase-catalyzed hydrolysis of pivaloyloxymethyl ethers, but a low signal-to-noise ratio caused by the spontaneous hydrolysis of the oxyamine made the system impractical. A similar observation was made by the group of Roelfes during their investigations toward designer enzymes catalyzing hydrazone and oxime bond formation.^348^ Contrary to the aforementioned report, base-induced fragmentation of their oxyamine-modified NBD–benzaldehyde adduct **138** caused the formation of the corresponding aldoxime and phenolate. The formation of *p*-nitrophenolate derivative NBD-O^–^ was used for the quantification of the catalytic efficiency of their mutants (Scheme 14). At 25 °C and pH 7.4 the system was able to discriminate the enzymatically catalyzed process against background hydrolysis of the unreacted probe.

### 9.6 Aza-Cope Reaction

**Figure 44:**
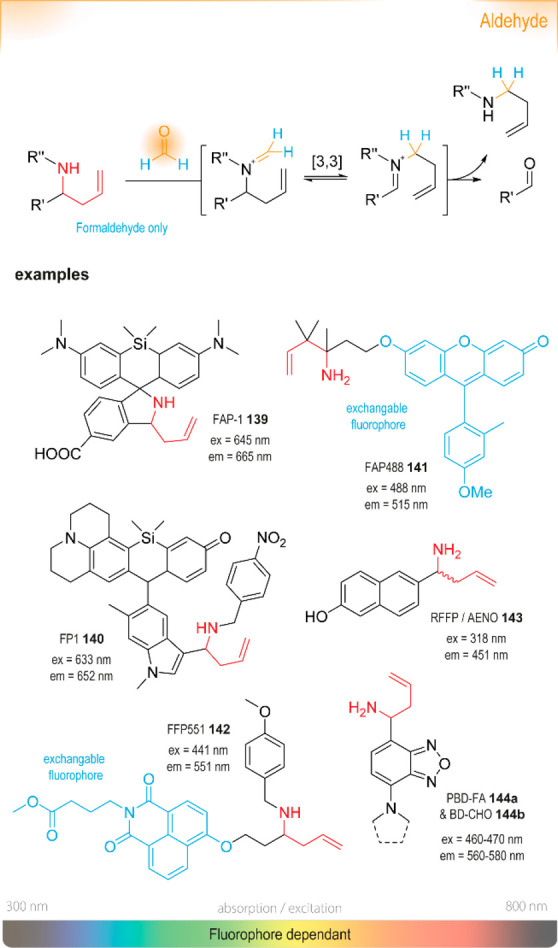
Summary of fluorogenic turn-on probes for the selective detection of formaldehyde based on the 2-aza-Cope sigmatropic rearrangement of initially formed Schiff’s bases. Fragmentation of these intermediates leads to the liberation of colored phenols. Ex or em: wavelengths typically used for excitation and emission in fluorescence measurements. The wavelengths of probes in this section are shown in the strip at the bottom representing the visible spectrum.

A recently reported approach for the detection of formaldehyde is based on mechanistic investigations from 1950 by Horowitz and Geissman.^349^ Their attempts to achieve double alkylation of allylamines with formaldehyde under acid catalysis remained unsuccessful and only formed cleavage products, which were recently shown to result from a 2-aza-Cope sigmatropic rearrangement (Figure 44). This finding led to the development of a diverse set of probes based on the same mechanism. Although this class of compounds has sparked growing interest over the last years, their reactivity limits applications to the detection of formaldehyde. Recent reviews summarize these and other approaches, including the detection method presented in this section.^359,360^

The first studies were both based on silicon rhodamine dyes but relied on different fluorogenic mechanisms. One approach built on the finding that spirocyclization renders rhodamine dyes weakly emissive at physiologically relevant pH.^350^ Probe FAP-1 **139** undergoes ring opening after an aza-Cope rearrangement. Subsequent hydrolysis of the resulting imine yields an aldehyde that can not re-(spiro-)cyclize.^351^ This assay could detect formaldehyde at 100 μM concentration by an ∼8-fold increase in fluorescence (1 h, pH 7.4). The other approach uses the same rearrangement to liberate a *p*-nitrobenzyl group, which is known to quench fluorescence through a donor-excited PET process.^352^ The assay using probe FP1 **140** could detect formaldehyde at 250 μM concentration by a ∼7-fold increase in fluorescence (3 h, pH 7.4).

Building upon these initial reports, the group of Chang developed their assay toward a general 2-aza-Cope reaction trigger with a self-immolative β-elimination linker. They created the homoallylamine probe **141** that, in response to formaldehyde, unmasked fluorogenic phenols on a fluorophore.^353^ By decoration of four different phenol-bearing fluorophores (coumarin, carbofluorescein, resorfurin, and rhodol), they achieved a 2–10-fold increase in fluorescence upon formaldehyde conjugation at 100 μM concentration (2 h, pH 7.4, 25 °C).

**Figure 45:**
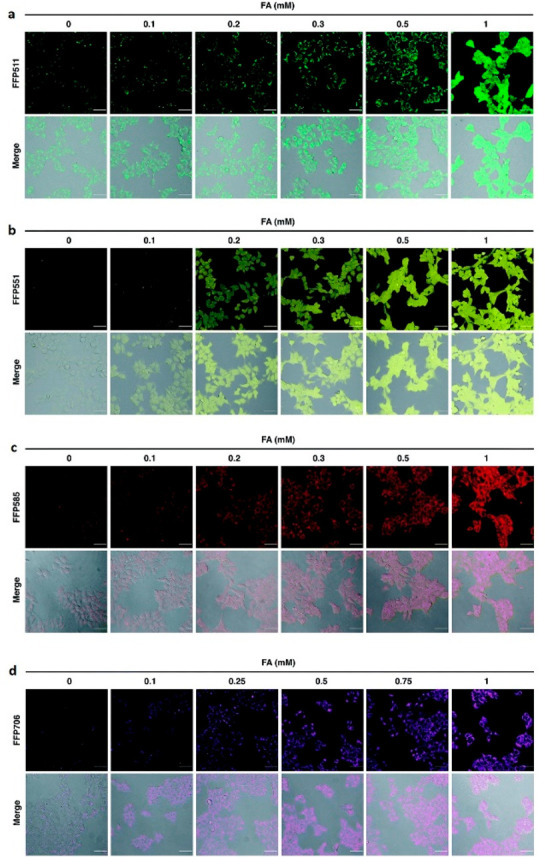
Confocal fluorescence images of exogenous formaldehyde (FA) with FFP fluorescent probes **142** in HEK293T cells. (A–D) Cells were loaded with (a) FFP511 (Tokyo Green fluorophore) (20 μM), (b) FFP551 (**142**) (10 μM), (c) FFP585 (resorufin fluorophore) (5 μM), or FFP706 (hemicyanine fluorophore) (2 μM) for 30 min and treated with FA of varying concentrations (0–1 mM) for 60 min before being imaged by confocal fluorescence microscopy. Scale bars represent 50 μm. Reproduced with permission from ref (354). Copyright 2021 The Royal Society of Chemistry.

Du *et al*. recently investigated a broad set of *N*-substituted homoallylamines **142** as probes for formaldehyde.^354^*N*-*p*-methoxybenzyl homoallylamine was found, by experiment and computation, to have the highest reactivity in the 2-aza-Cope reaction with formaldehyde. Using similar self-immolative linkers as above, they synthesized five silenced phenol-bearing fluorophores **142**, with excitation wavelengths ranging from 445 to 706 nm. Exposure to formaldehyde at 500 μM concentrations under physiological conditions prompted a turn-on factor of 5–125. All probes were successfully applied in the visualization of changes of intracellular formaldehyde in living cells (Figure 45).^354^

**Figure 46:**
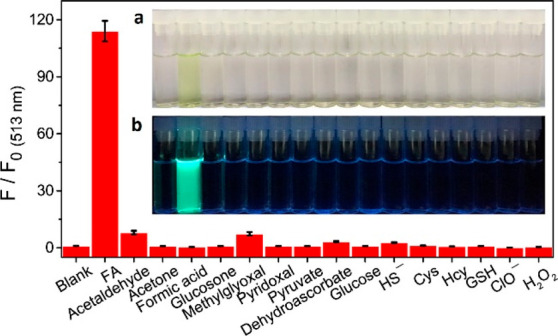
Fluorescent responses of **143** (10 μM) in DMF/PBS solution (v/v = 1/4, pH 7.4, 10 mM) upon addition of various small molecular species (5.0 mM). λ_ex_/λ_em_ = 390/513 nm; slits, 2.5/2.5 nm. Each spectrum was recorded after 3 h at 37 °C. Error bars are ±1SD, *n* = 3. Inset: (a) The color and (b) fluorescence images of **143** (10 μM) in the presence of various small molecular species (5.0 mM).^356^ Reproduced with permission from ref (356). Copyright 2016 Elsevier BV.

Making these reagents typically requires great synthetic effort, which is impractical for biochemists. Consequently, many probes based on simple synthetic modifications of known fluorophores have been developed in recent years. For instance, He *et al*. and Xu *et al*. reported the naphthalene-based probe RFFP/AENO **143**, which can be synthesized in a one-pot reaction from commercially available 6-hydroxy-2-naphthaldehyde.^355,356^ The use of the reagent as turn-on probe with a 100-fold enhancement of fluorescence within 2.5 h for 1 mM formaldehyde was reported (Figure 46).^356^

The groups of Lin and Peng reported the synthesis of turn-on probes PDB-FA **144a** and BD-CHO **144b**, based on NBD as a fluorophore,^357,358^ in a three-step procedure from commercial 4-chlorobenzoxadiazole. The probes showed 55-fold (PDB-FA) and 255-fold enhancement (BD-CHO) upon exposure to excess formaldehyde after 2 h at pH 7.4.^359,360^

## 10 Chromo- and Fluorogenic Probes for Ketones

Ketones are widespread in nature. This electrophilic functional group is present, for instance, in the carbohydrate family of ketoses, as a polar element in important steroids (*e.g*., testosterone, progesterone), in fragrance compounds derived from terpenoid units,^361^ or as the structurally important methyl ketone motif^362^ (*e.g*., camphor, carvone, pulegone, 2-heptanone). Ketones are also found as biological intermediates and energy storage units in important regulatory systems of cells (Krebs cycle,^363^ fatty acid synthesis,^364^ ketogenesis^365^).

**Figure 47:**
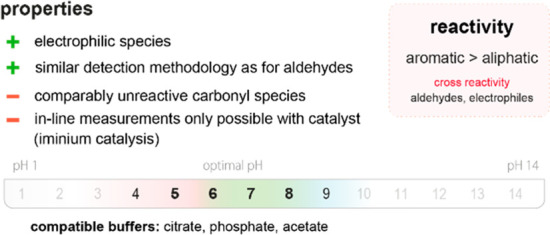
(top) Summary of general properties of ketones, reactivity trends toward ketone-selective probes as well as expected cross-reactivity during screenings. (bottom) Compatible pH range and buffers in reported ketone-selective assays. Bold pH numbers indicate the most commonly used pH values.

In industry, ketones are widely used in bulk as solvents (*e.g*., acetone or methyl ethyl ketone, MEK) or as synthetic intermediates. They have found broad use in biocatalysis as starting materials for the synthesis of chiral alcohol and amines^366^ using transaminases (TA), alcohol dehydrogenases (ADH),^367^ Baeyer–Villiger monooxygenases (BVMO),^368^ or aldolases.^369^

**Scheme 15:**
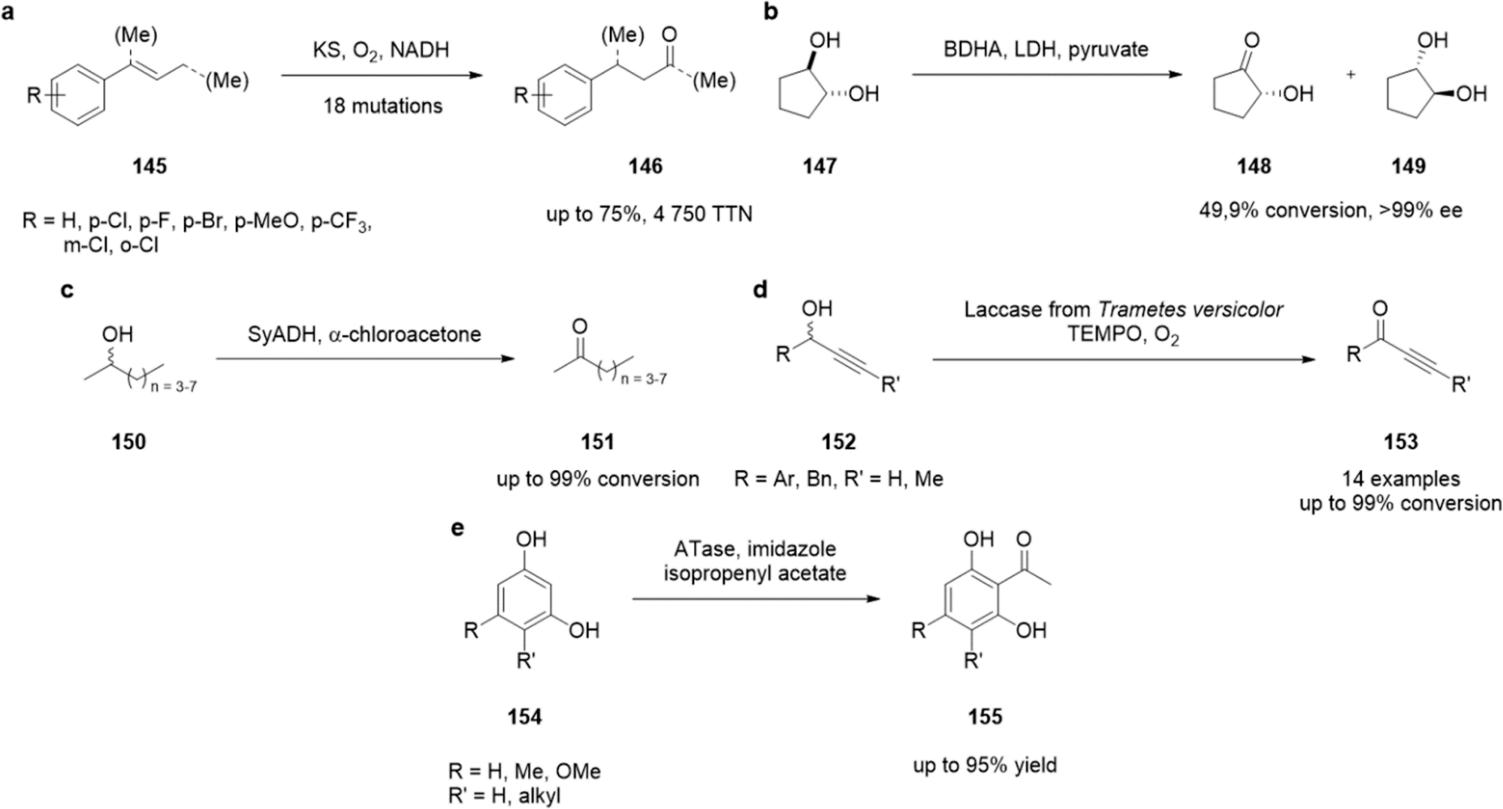
Representative Selection of Enzymatic Transformations for the Production of Carboxylic Acid-Containing Products

They can be synthesized biochemically from β-ketoacids by decarboxylation using carboxy lyases,^370^ using aldolases,^371^ from carbinols using TDP-dependent lyases,^372^ or using acyl transferases (ATase).^373^ These biocatalysts have already been demonstrated as good alternatives in organic synthesis. The group of Hammer recently optimized a ketone synthase for the regioselective *anti*-Markovnikov oxidation of aromatic alkenes. Starting from a P450 wild-type, 12 rounds of evolution identified 18 mutations that were beneficial for the stereoselective oxidation of styrene derivatives **145** (Scheme 15a).^374^

Many applications of ketone synthesis use alcohol dehydrogenases as mild oxidative catalysts. Zhang *et al*. applied the highly regio- and enantioselective ADH from *Bacillus subtilis* BDHA for the oxidation of racemic *trans*-cyclic vicinal diols **147** (Scheme 15b)^375^ to (*R*)-α-hydroxy ketones **148** and (*S*,*S*)-cyclic diols **149** in >99%*ee* at 50% conversion in one pot.

The group of Kroutil identified an ADH from *Sphingobium yanoikuyae* for the opposite purpose, oxidizing the aliphatic alcohols **150** to the corresponding ketones **151** without stereopreference and without the need for a huge excess of ketones as hydrogen acceptors for cofactor recycling (Scheme 15c).^376^ The researchers found that α-Cl, -F, or -MeO substituted ketones were not oxidized, hence limiting the necessity for organic recycling reagents to one molar equivalent only.

González-Granda *et al*. reported the oxidation of propargylic alcohols **152** using the catalytic system composed of a laccase from *Trametes versicolor*, the oxy-radical (2,2,6,6-tetramethylpiperidin-1-yl)oxyl (TEMPO), and oxygen as terminal oxidant.^377^ Under these mild conditions, the products were obtained in good yields (Scheme 15d).

The group of Kroutil was the first to report an enzymatic Friedel–Crafts acylation of activated arenes **154**, a reaction that is typically in the realm of classical organic chemistry, as well as the first biocatalytic equivalent to the Fries rearrangement (the intramolecular analogue of the Friedel–Crafts reaction).^373^ They reported conversions of up to 95%, using readily available isopropenyl acetate as acetyl donor and without any CoA-activated reagents (Scheme 15e).

Ketones are much less reactive than most aldehydes due to an increase in steric bulk and the diminished electrophilicity caused by the electron-donating effect of the attached alkyl groups (Figure 47). This relatively low activity makes specific derivatization of ketones challenging; reactivity and selectivity of reagents are inversely correlated. With certain exceptions, approaches are similar to those introduced in the section on aldehydes (*cf*. section 9).

Ketones react slowly in conjugations under physiological conditions,^295^ particularly at low concentration. Most assays require harsh reaction conditions that are incompatible with biological systems. Only a few studies have addressed the challenge of developing selective conjugations for ketones, so far, likely for the following reasons: (i) Aldehydes are generally more important in synthesis due to their broader versatility as chemical building blocks, (ii) It is difficult to match the rates of aldehyde conjugations (under both abiological and physiological conditions). Ketones are mostly used as negative examples to highlight the specificity of carbonyl probes for aldehyde conjugations.

### 10.1 Hydrazone Formation

**Figure 48:**
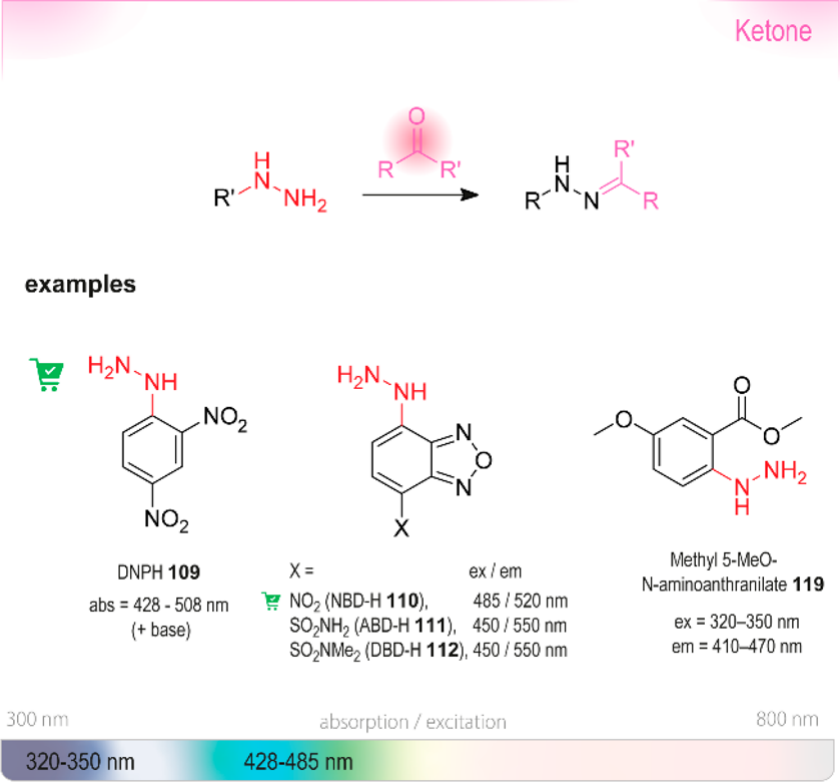
Summary of chromo- and fluorogenic turn-on probes for the selective detection of ketones based on the formation of hydrazone adducts. Abs: wavelengths typically used for UV absorbance measurements. Ex or em: wavelengths typically used for excitation and emission in fluorescence measurements. The wavelengths of probes in this section are shown in the strip at the bottom representing the visible spectrum.

**Figure 49:**
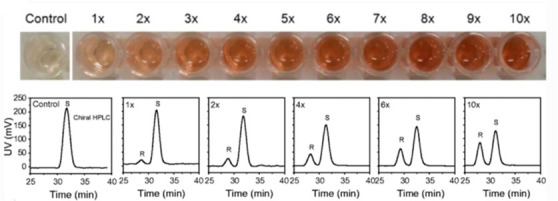
Variation in color of reaction mixtures containing different concentrations of **109** and the validation of the developed HTS method by chiral HPLC analysis. Control, no **109** was added into the reaction mixture; 1–10×, different concentrations of **109** were added into the reaction mixture (1×, 8.4 mg/mL). Reproduced with permission from ref (289). Copyright 2017 Springer-Verlag.

The most promising probes to detect ketones use the fast reaction of carbonyl compounds with hydrazines forming stable hydrazones (Figure 48). In principle, the approach is the same as for aldehydes, summarized above (*cf*. section 9.1), but only a small number of assays showed useful reactivity toward ketones. The long-known reagent DNPH **109** has been used by Yang *et al*. as an at-line analytical tool and in HTS for the activity of mandelate racemase variants by detection of oxidized α-hydroxy acids.^289^ Enantioselective mandelate dehydrogenase D-MDH was used to selectively oxidize the desired enantiomer, which was converted to the hydrazone using **109** and detected at 450 nm after basification with aqueous NaOH (Figure 49).

The BD-probes (NBD-H **110**, ABD-H **111**. DBD-H **112**) form fluorescent derivatives with aliphatic and aromatic ketones at room temperature, but only in ACN.^290^ Among these compounds, **110** is the most broadly applied for conjugations with aldehydes and ketones that work even under physiological conditions.^293,378^ Crisalli and Kool used anilines to catalyze conjugations at neutral pH. Reactions with aromatic ketones were improved using these catalysts, but they still proceeded slower than reactions with aldehydes.^296,379^ Wang *et al*. showed that increased salt concentrations were beneficial for conjugation rates at physiological pH.^297^

Among the already described fluorogenic hydrazines (*cf*. section 9.1), methyl 5-MeO-*N*-aminoanthranilate **119** was tested as a probe for aliphatic ketones and showed a small (unquantified) increase in fluorescence emission upon conjugation.^314^

### 10.2 Aldol Reaction

**Figure 50:**
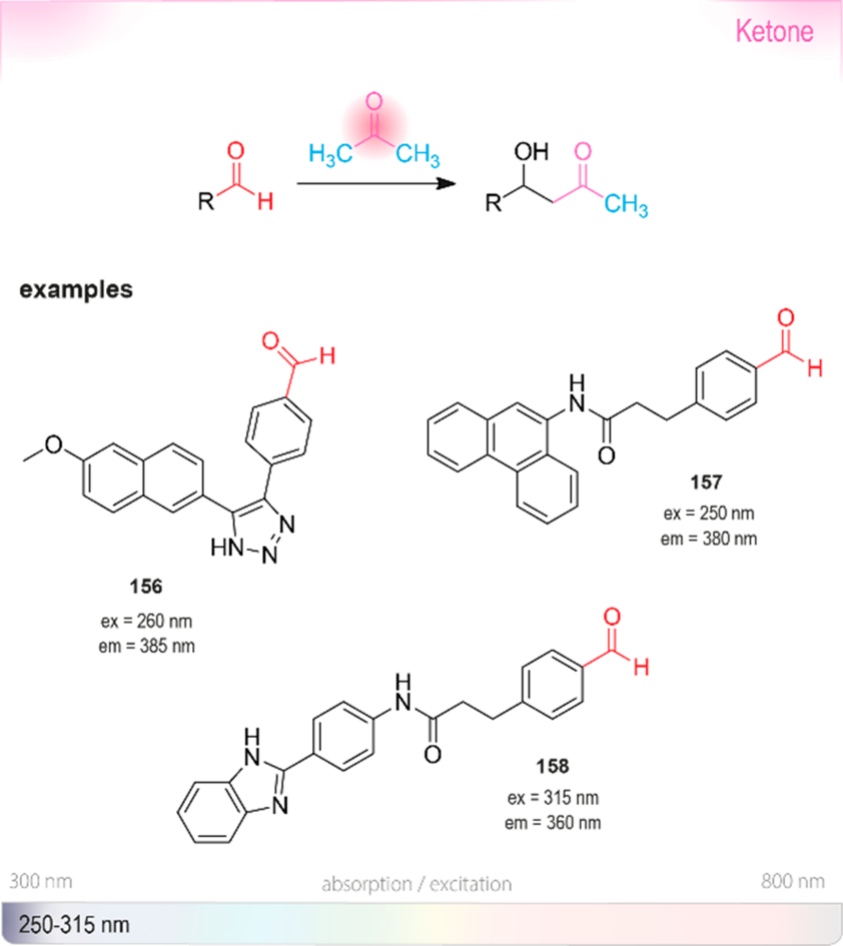
Summary of fluorogenic turn-on probes for the selective detection of acetone based on aldol reactions. Ex or em: wavelengths typically used for excitation and emission in fluorescence measurements. The wavelengths of probes in this section are shown in the strip at the bottom representing the visible spectrum.

When ketones tautomerize to enols, they can participate in aldol reactions (Figure 50). Similar to the hemiaminal formation used for the detection of amines (*cf.*section 6.7), electron-withdrawing carbonyl groups can be used to trigger a hypsochromic shift of the absorption maximum upon aldol reactions. This method has been explored by the group of Barbas and Tanaka.^380−382^ They investigated different designs (compounds **156**–**158**) to detect acetone, which required activation *via* enamines formation using catalytic amounts of pyrrolidine, arginine, glycine, proline, or the aldolase antibody 38C2. A 1000-fold excess of the substrate was required to afford useful conversions. The triazine aldehyde **156** and the phenanthrene aldehyde **157** showed similar excitation and emission properties (approximately 100–1000-fold turn-on) from pH 5 to 9. Exchange of the linked amide aryl-moieties to the benzimidazole analogue **158** red-shifted the excitation maximum from 260 to 315 nm. In turn, it also lowered the sensitivity of the assay (3–20-fold turn-on) due to the parent aldehydes becoming stronger emitters.

### 10.3 Michael Addition

**Figure 51:**
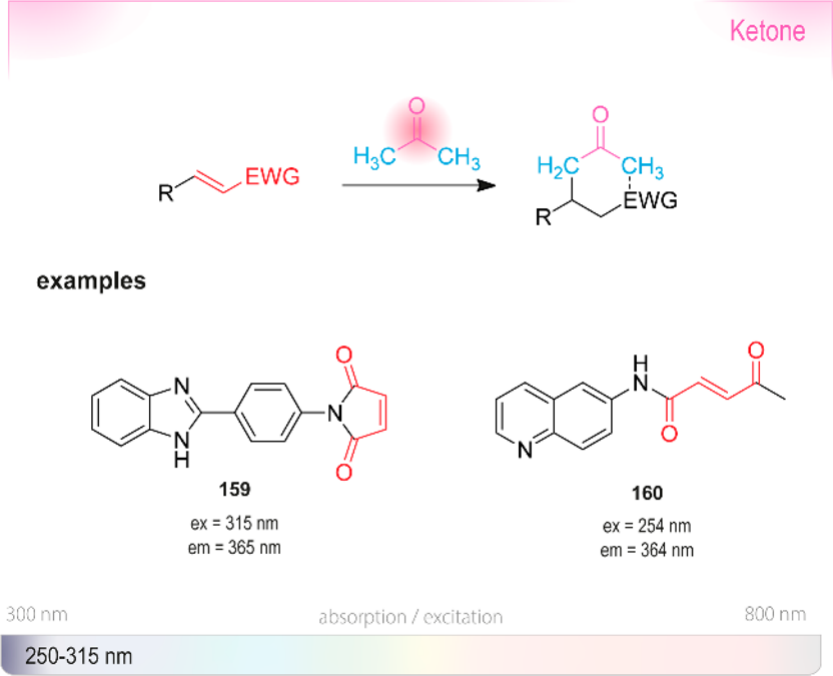
Summary of fluorogenic turn-on probes for the selective detection of acetone based on a Michael addition approach. Ex or em: wavelengths typically used for excitation and emission in fluorescence measurements. The wavelengths of probes in this section are shown in the strip at the bottom representing the visible spectrum.

**Scheme 16:**
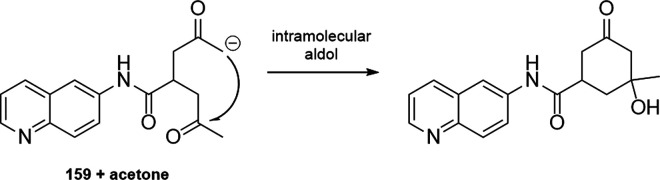
Michael Addition of Acetone onto **159** Triggers a Subsequent Aldol Reaction Forming Cyclic Hydroxyketones

The activation *via* enamine formation can be also used with Michael acceptor probes in 1,4-additions. The benzimidazole **159** and 6-aminoquinoline **160** have useful turn-on properties upon conjugation to ketones (Figure 51).^381^ Exposition of fluorogenic 6-aminoquinoline probe **160** to acetone additionally triggers an immediate cyclization by aldol-condensation with the terminal methyl ketone (Scheme 16). Due to a strong spectral overlap between probe and conjugate, their application is problematic. Both probes are slower than the previously reported aldol sensors (*cf*. section 10.2) and also require a 1000-fold excess of acetone. Due to the unattractive properties of the screening system, no additional studies were undertaken.

### 10.4 Other Methods

**Figure 52:**
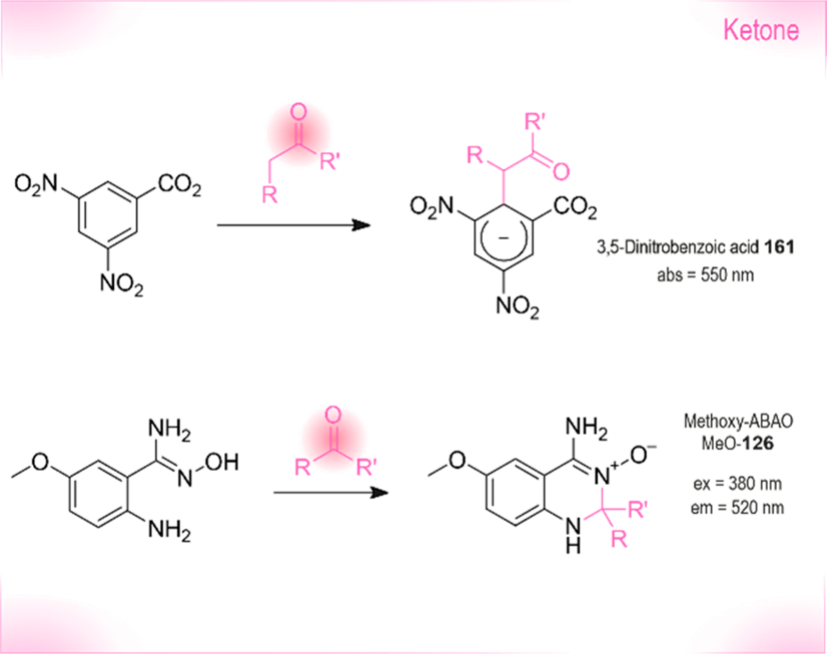
(top) Chromogenic and fluorogenic detection of ketones bearing an enolizable position *via* the formation of the purple-colored Janovsky complexes with 3,5-dinitrobenzoic acid **161** under strongly basic conditions. (bottom) Fluorogenic detection of ketones *via* the formation of fluorescent ABAO-adducts (*cf*. section 9.2). A methoxy substitution of the ABAO core **126** is necessary to increase the reactivity of the aniline NH_2_ position.

Two additional methods for the detection of ketones are (i) the Janovsky reaction, and (ii) reaction with ABAO **126** (Figure 52).

The Janovsky reaction requires analytes to form reactive enolates under strongly basic conditions, which then form stable, purple-colored σ-complexes with 3,5-dinitrobenzoic acid **161**. This method was used to rapidly evaluate 11 substrates for Baeyer–Villiger monooxygenases by monitoring the decrease of absorbance at 550 nm.^383^

Reaction with ABAO, initially suspected to be unreactive toward ketones, emerged as an effective probe for aliphatic and aromatic ketones.^324^ The methoxy analogue **MeO-126** reduced long reaction times with its reactivity enhanced by the electron-donating effect of the methoxy group. A set of 24 ketones was tested at pH 5.0 using an equimolar amount of ABAO at 10 mM concentration. Fluorometric evaluation of the reaction after 30 min revealed a ∼2–20-fold turn-on. The assay was applied in enzyme mining and directed evolution of alcohol dehydrogenases (ADHs) for the catalytic oxidation of rhododendrol to raspberry ketone (*p*-hydroxybenzyl acetone).

## 11 Chromo- and Fluorogenic Probes for Carboxylic Acids

Carboxylic acids are found in many compounds in nature,^384^ pharmaceuticals,^385^ and food constituents.^386^ Many structures bearing this functional group are involved in essential physiological processes, such as prostaglandins regulating inflammation and blood pressure^387^ or citric acid and succinic acid in the Krebs cycle.^388^ Fatty acids are the building blocks of lipids and are involved in energy metabolism,^389^ whereas α-amino acids are the building blocks for peptides and proteins.^390^

**Figure 53:**
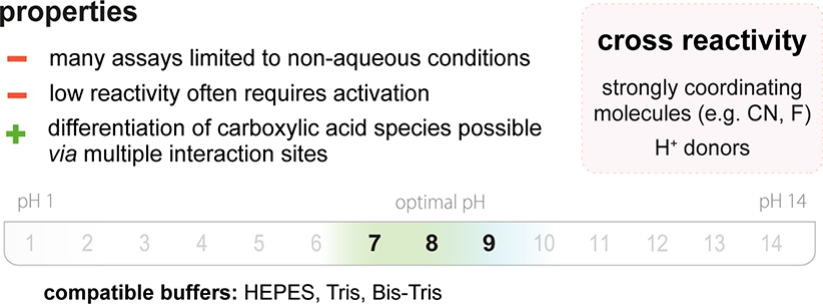
(top) Summary of general properties of carboxylic acid sensors as well as expected cross-reactivity during screenings. (bottom) Compatible pH range and buffers in reported carboxylic acid-selective assays. Bold pH numbers indicate the most commonly used pH values.

Various biocatalytic methods for the synthesis of carboxylic acids have been developed, complementing nonenzymatic approaches.^391^ These methods include the hydrolysis of nitriles by nitrilases,^392−394^ the conversion of aldehydes by hydroxynitrile lyases with subsequent hydrolysis,^395^ the hydrolysis of esters by esterases,^396^ and the oxidation of alcohols or aldehydes by dehydrogenases and oxidases.^397^

**Scheme 17:**
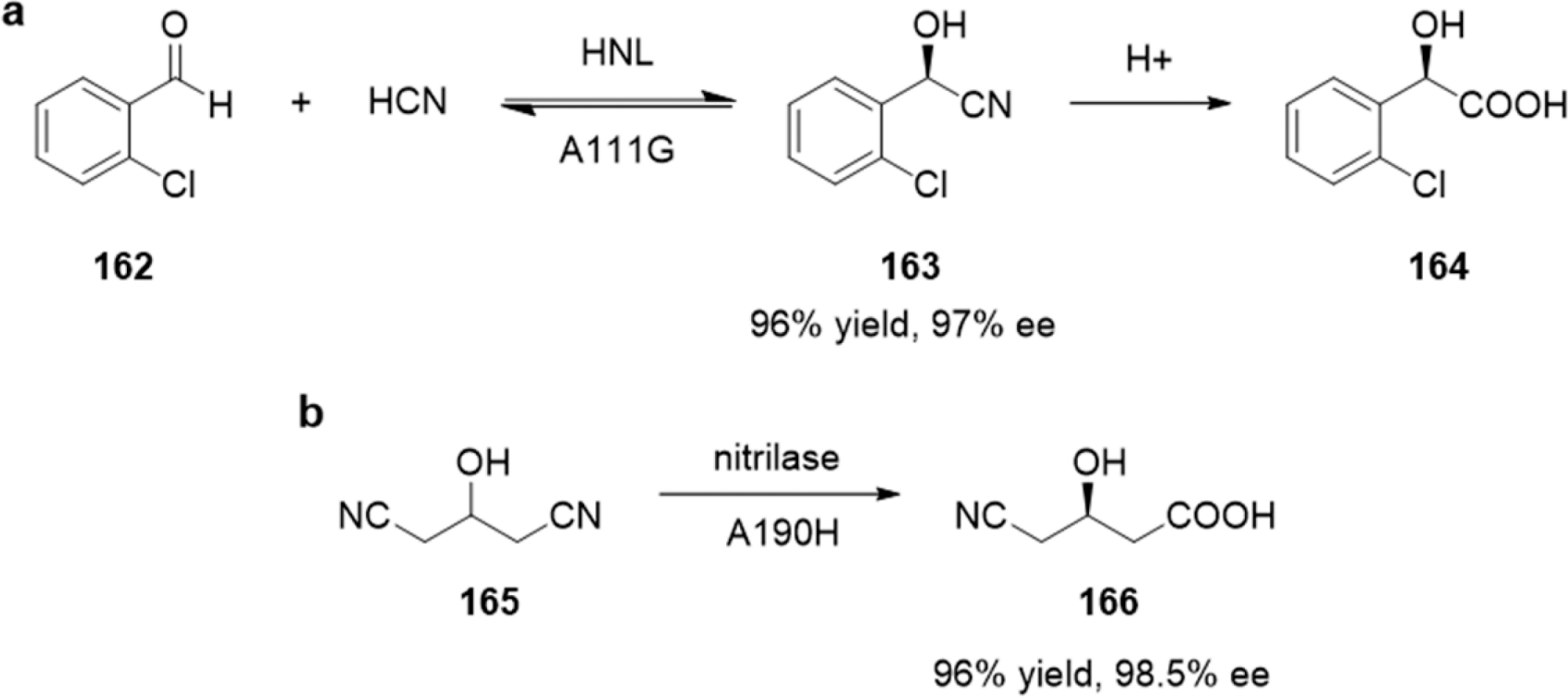
Representative Selection of Enzymatic Transformations for the Production of Carboxylic Acid-Containing Products

Glieder *et al*. reported a hydroxynitrile lyase mutant from *Prunus amygdalus* for the enantioselective synthesis of α-hydroxyacids by the addition of HCN to aldehydes and the subsequent hydrolysis of the intermediately formed cyanohydrins. The enzyme was stable under acidic conditions and enabled the conversion of 2-chlorobenzaldehyde **162** to (*R*)-2-(2-chlorophenyl)-2-hydroxyacetonitrile **163** at pH 3.4 with 96% yield and 97%*ee*. Hydrolysis of the intermediate yielded the enantiopure α-hydroxyacid **164** (Scheme 17a).^395^

DeSantis *et al*. identified a nitrilase mutant for production of (*R*)-4-cyano-3-hydroxybutyric acid **166**, an intermediate in the synthesis of the drug Lipitor, by directed evolution. The hydroxybutyric acid **166** could be obtained in 96% yield with 98.5%*ee* and a volumetric productivity of 619 g L^–1^d^–1^ (Scheme 17b).^398^

The reactivity of carboxylic acids is mainly determined by their acidic hydroxy group and carbonyl functionality. Under physiological and basic conditions, carboxylic acids (p*K*_a_ ∼ 3–5^399,400^) are deprotonated, negatively charged carboxylates, which render nucleophilic attacks at the carbonyl center impossible (Figure 53).^401^ Also, protonated carboxylic acids are much less reactive than derivatives like acyl halides or anhydrides, which generally makes an activation for derivatizations necessary. Therefore, detection methods based on the conversion of an acid to a fluorescent derivative (*e.g*., hydrazide, hydroxamate) usually involve activation as the first step (*e.g*., with dicyclohexylcarbodiimide DCC, **167**). Many approaches for carboxylic acids involve monitoring of pH shifts by pH indicators^406^ and the enzymatic conversion of acids with NADH.^407^

Other methods exploit the properties of carboxylic acids and carboxylates to form intermolecular hydrogen bonds, which enable their detection as adducts with probes that exhibit hydrogen bond donating groups, such as amides, thioureas, or guanidinium moieties.^403,404^ Some carboxylate probes are based on the coordination of the anion to a metal ion, *e.g*., Zn^2+^ or Cu^2+^.^405^

A challenge in sensing carboxylic acid is the selective detection and the differentiation of acids.^403,408,409^ One method to improve selectivity is introducing multiple recognition sites at the probe.^405,409^ Many of the assays require nonaqueous conditions or apolar/aprotic solvents (*e.g*., chloroform) to prevent competitive interactions between the solvent and the binding sites of the probe.^410−413^

### 11.1 Activation and Nucleophilic Trapping

**Figure 54:**
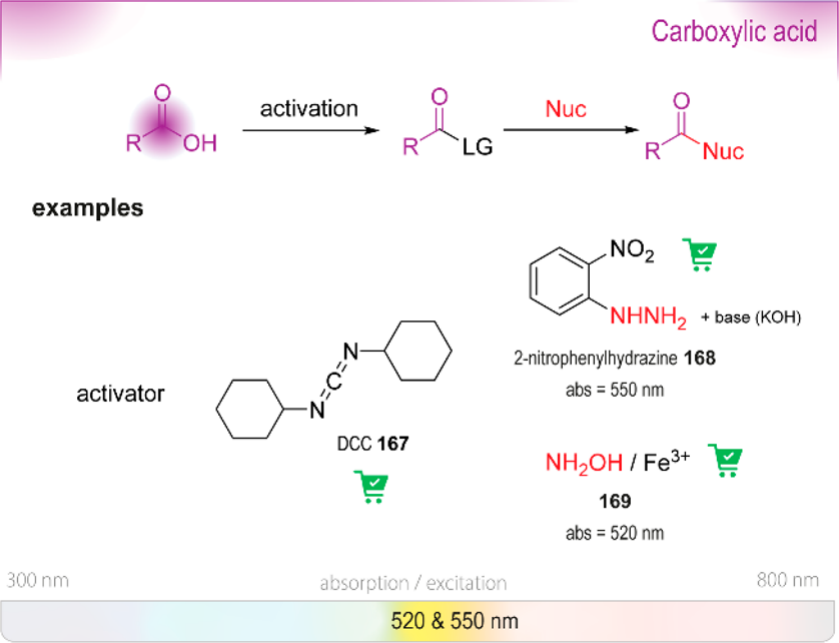
Summary of chromogenic turn-on probes for the selective detection of carboxylic acids based on the entrapment of activated carboxylic acid species. Abs: wavelengths typically used for UV absorbance measurements. The wavelengths of probes in this section are shown in the strip at the bottom representing the visible spectrum.

Carboxylic acids can be detected by converting them to acid hydrazides with hydrazine derivatives (Figure 54).^414,415^ Miwa *et al*. reported a colorimetric method using 2-nitrophenylhydrazine **168** in ethanolic solution in the presence of **167** as a coupling agent and catalytic amounts of pyridine. The resulting acid hydrazides had a violet color upon basification with KOH, and their absorbance could be measured at about 550 nm.^416^

Another colorimetric method uses derivatization of activated carboxylic acids to hydroxamic acids with hydroxylamine **169**, followed by the formation of a colored ferric hydroxamate (Figure 54).^417,418^ Based on work by Kasai *et al*.^419^ and Takeuchi *et al*.,^420^ He *et al*. reported a spectrophotometric HTS assay to detect various aliphatic and aromatic carboxylic acids in aqueous solutions at room temperature by converting them to ferric hydroxamates. The carboxylic acids were converted to hydroxamic acids with hydroxylamine perchlorate **169·ClO**_**4**_ in the presence of **167**. Purple ferric hydroxamates were formed upon the addition of an acidic solution of ferric perchlorate. The absorbance of the ferric hydroxamate was measured at 520 nm. He *et al*. applied this method to assay the nitrile-hydrolyzing enzymes *Rhodococcus erythropolis* CGMCC 1.2362 and *Alcaligenes* sp. ECU0401.^421^

**Figure 55:**
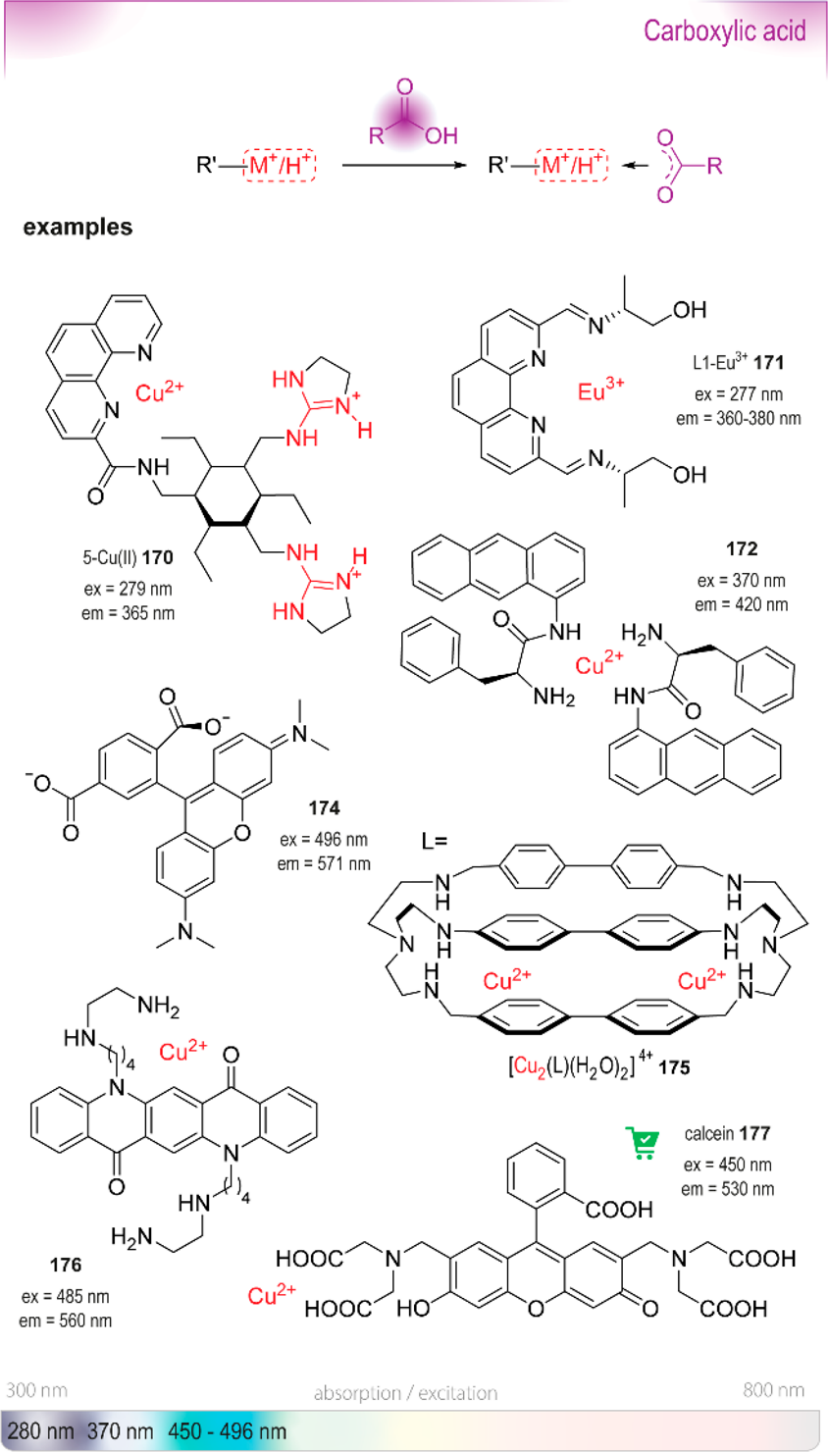
Summary of fluorogenic turn-on probes for the selective detection of carboxylic acids based on the coordination to metal centers or hydrogen bond donors. Ex or em: wavelengths typically used for excitation and emission in fluorescence measurements. The wavelengths of probes in this section are shown in the strip at the bottom representing the visible spectrum.

### 11.2 Coordination to Metals and Hydrogen Bond Donors

Formation of complexes with a probe *via* hydrogen bonding or electrostatic interactions can be used to detect carboxylic acids and carboxylates (Figure 55).^405,422−424^ These interactions can induce fluorescence by electronic changes of the probe: Nonbonding electron pairs of amines or adjacent electron-donating groups are known to quench fluorescence by PET processes, which can be restored by coordination of the amines to carboxylic acids.^410,424^ Similarly, quenching has been observed by binding of amine probes to Cu^2+^ ions. By coordination and thus scavenging of the Cu^2+^ ions by carboxylates, the probe’s fluorescence emission can be effectively restored.^413,425^ In some cases, hydrogen bonding or electrostatic interactions of carboxylate and probe lead to a fluorescence response by the displacement of a noncovalently bound fluorescent indicator from the probe (indicator-receptor-complex) by the carboxylate which results in the release of the fluorescent dye.^408,411^

**Figure 56:**
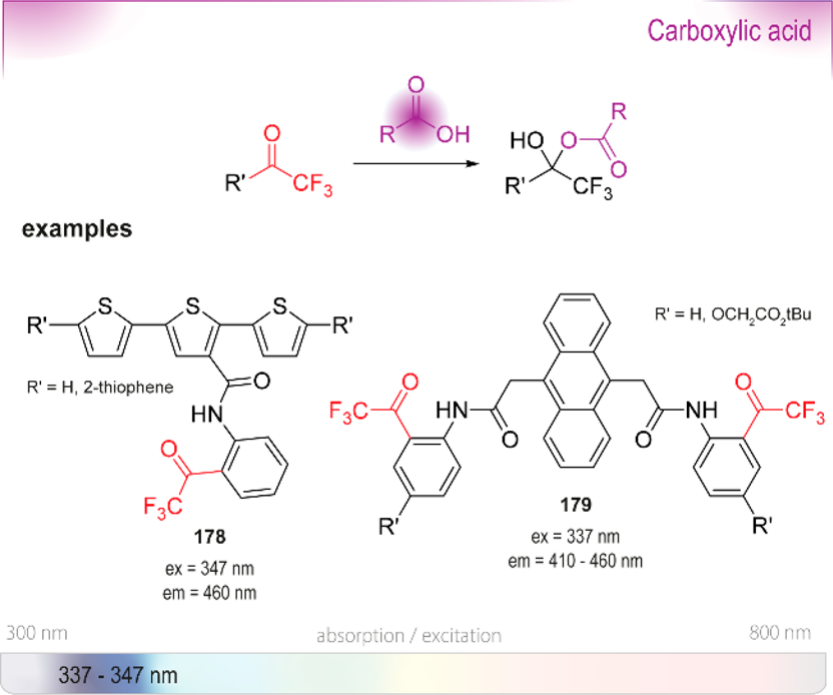
Summary of fluorogenic turn-on probes for the selective detection of carboxylic acids based on nucleophilic addition onto trifluoroacetyl groups. Ex or em: wavelengths typically used for excitation and emission in fluorescence measurements. The wavelengths of probes in this section are shown in the strip at the bottom representing the visible spectrum.

Cabell *et al*. developed probe **170** for the detection of citrate in HEPES buffer (pH 7.4), enabling the quantification of citrate in beverages at μM concentration. Probe **170** uses a 1,10-phenanthroline moiety as a fluorophore and guanidinium moieties as recognition sites ensuring effective hydrogen bonding to carboxylates in water. Fluorescence is quenched while phenanthroline binds Cu^2+^. When tribasic citrate binds to Cu^2+^ and the two guanidinium groups of the probe, electron transfer from the metal to the fluorophore changes as the metal becomes more electron-rich, which leads to an increase in fluorescence at 365 nm.^425^

Xu *et al*. developed the Eu^3+^ complex **171**, based on a phenanthroline scaffold with chiral amino alcohol groups as ligands that enabled the selective detection of malate anions in aqueous solutions (0.01 M HEPES, pH 7.4, with 0.3% DMSO). Binding of Eu^3+^ suppresses the fluorescence at about 380 nm, which gets restored when malate forms a complex with the ligand. Other carboxylates, such as alanine, phenylalanine, mandelate, and tartrate, did not show significant responses. The authors assumed that the selectivity for malate arose from the preorganized structure of **171** and the complementary shapes of probe and anion. The enantiomers of malate gave different responses: 487% enhancement with l-malate and 332% with d-malate.^426^

Burguete *et al*. reported a detection method for citrate based on the fluorescent anthracene-probe **172** in methanolic aqueous solution (70%, pH 7.5–8.0). Their probe consisted of a nonfluorescent Cu^2+^ complex of two aminoanthracene moieties, each coupled with an l-phenylalanine, and enabled the detection of citrate in the μM range. The addition of citrate led to a displacement of the anthracene moieties, which resulted in a more than 15-fold increase in fluorescence at 420 nm. Mandelate, tartrate, and malate gave small increases in fluorescence (∼10–15%) compared to citrate; succinate, fumarate, lactate, acetate, and glutarate did not produce any increase. Only l-glutamate, which is similar to citrate in the number of potential coordination sites (two carboxylates and one amino group), showed a similar increase in fluorescence (40%).^427^

Boiocchi *et al*. developed a probe based on the indicator-displacement approach to detect dicarboxylates in water. The binding of the cryptate complex **175** to the fluorescent indicator carboxy-rhodamine **174***via* the coordination of the two Cu^2+^ ions to two carboxylates led to the quenching of the indicator’s (**174**) fluorescence. A displacement of **174** by selected dicarboxylates restored fluorescence at λ_em, max_ = 570 nm. Only 1,4-, 1,5-, and 1,6-dicarboxylates terephthalate, glutarate, and adipate that all have an appropriate spacial distance between their COO^–^ groups (irrespective of their carbon chain length) could coordinate to both Cu^2+^ ions and displace the indicator. Dicarboxylates with shorter or longer distances (*e.g.*, succinate, pimelate, phthalate, or isophthalate) led to no or only minor fluorescence emission. In the case of the very small oxalate anion, a coordination of two molecules to bind both Cu^2+^ also resulted in the displacement of the indicator.^411^

Amino acids are known to form stable complexes with Cu^2+^ ions.^408^ Klein and Reymond reported the Cu^2+^-based probe **176** that was used in HTS of the hydrolysis of *N*-acetyl-l-methionine by acylase I and of l-leucinamide by aminopeptidase. The complex of the quinacridone-derived ligand and Cu^2+^, gave no fluorescent signal. Due to the weak chelating effect of this macrocycle, amino acids competed with the ligand and displaced it. The fluorescence of the released quinacridone-derived ligand was measured at 584 nm. The low solubility of **176** in aqueous buffer required the use of organic cosolvent (∼40%), lowering enzymatic activity; alternatively, the probe and solvent could be added after the enzymatic reaction has completed.^428^

In later reports, substitution of the quinacridone-derived ligand with the commercial fluorescein-based ligand calcein **177** was proven to be beneficial due to its increased solubility in aqueous buffers. The probe showed enhanced fluorescence at 530 nm with various amino acids, such as valine, alanine, methionine, leucine, serine, histidine, *N*-acetylmethionine, and glycine, at μM concentrations. The assay worked at pH 6–10 in various buffers (bis-tris, phosphate, borate, Tris) and mixed solvent systems (0–50% organic), including EtOH, MeOH, ACN, and DMSO. Strongly coordinating buffers (*e.g*., bis-tris-propane) are incompatible because they bind to metal ions. The assay does not work at low pH as the protonated carboxyl groups of **177** do not coordinate well. Probe **177** enabled HTS for activity of proteases, such as trypsin, chymotrypsin, and subtilisin.^429^ Xu *et al*. validated this approach and used the Cu^2+^–calcein complex for HTS of more than 2500 leucine dehydrogenase mutants with 2-oxobutyric acid as substrate. They detected 11 mutants with a higher activity than the wild type.^430^

### 11.3 Nucleophilic Addition onto Trifluoroacetyl Groups

Carboxylates can react with trifluoroacetyl ketones in a nucleophilic addition (Figure 56). As in sections 7.1 and 6.7, addition disrupts electron-withdrawing effect of the carbonyl group on conjugated systems and leads to changes in fluorescence.^431,432^ Kim and Ahn developed trifluoroacetophenone derivatives **178** with a terthiophene or a pentathiophene moiety as fluorophores and a trifluoroacetyl group as recognition site for carboxylates. Fluorescence of the terthiophene probe rose 120-fold in the presence of acetate in ACN. Other anions, such as phosphate, fluoride, and cyanide, also enhanced the fluorescence.^432^

The trifluoroacetyl carbonyl moiety can also function as a recognition site for amines (*cf*. section 6.7), as shown by Ryu *et al*. in the development of probe **179**, which uses two trifluoroacetyl groups for the detection of α-amino carboxylates. Binding of the amine and the carboxylate group to both recognition sites resulted in the formation of a bridged adduct accompanied by an increase in fluorescence. The addition of glycinate resulted in a 110-fold enhancement of fluorescence in ACN. Other α-amino carboxylates caused similar emissions; the α-substituents did not affect the formation of the adduct. Probe **179** discriminated α-amino carboxylates against β- and γ-carboxylates.^431^

## 12 Chromo- and Fluorogenic Probes for Carboxylic Acid Esters

Carboxylic acid esters are widely found in nature,^433^ pharmaceuticals,^434,435^ fine chemicals,^436^ agrochemicals,^437^ and the food industry.^438^ Most are long-chained triglycerides in fats and oils and are of central importance in energy metabolism.^389,439^ Short-chained derivatives are typically found in fruit and flower fragrances (*e.g*., benzyl acetate in jasmine^440^ or isoamyl acetate in banana^441^). Due to their flavor-carrying and fragrant nature, they are valuable ingredients in cosmetics, food, and beverages.^442^ Moreover, they are important building blocks for polymers,^443^ lubricants,^444^ plasticizers,^445^ and surfactants.^446^ Enzymatic approaches for the synthesis of carboxylic acid esters include esterifications and transesterifications catalyzed by esterases and lipases.^447−449^

**Figure 57:**
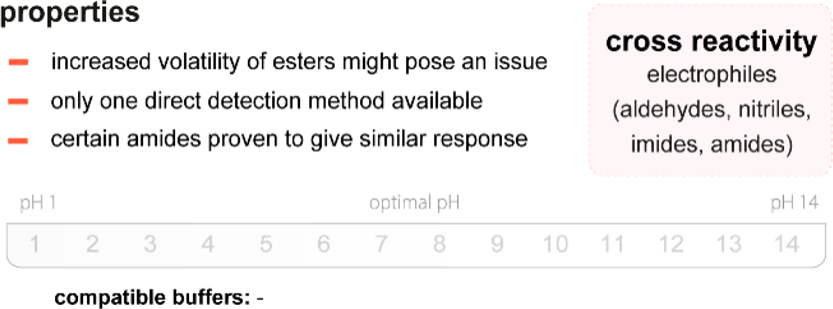
(top) Summary of general properties of carboxylic acid ester sensors as well as expected cross-reactivity during screenings. Due to the lack of direct reactive probes applicable in aqueous environments, no compatible buffers and pH ranges can be stated.

**Scheme 18:**
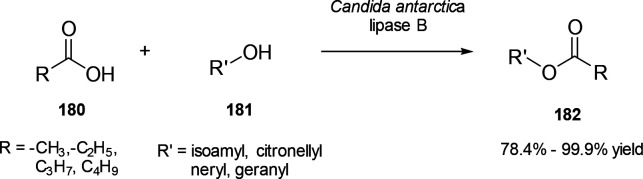
Representative Selection of Enzymatic Transformations for the Production of Carboxylic Acid Ester-Containing Products

Lozano *et al*. reported a biocatalytic approach for the synthesis of aliphatic esters **182** by esterification of C1–C4 carboxylic acids **180** with fragrant alcohols **181** (isoamyl alcohol, citronellol, geraniol, and nerol) using an immobilized lipase from *Candida antarctica* (Novozym 435) in the ionic liquid *N*,*N*′,*N*″,*N*′′′-hexadecyltrimethylammonium bis(trifluoromethylsulfonyl)imide (Scheme 18). The authors optimized the workup procedure and achieved yields >78% for all esters. Geranyl valerate was obtained with the highest product concentration of 0.757 g mL^–1^.^450^

Carboxylic esters are compounds derived from carboxylic acids and alcohols. The electrophilic carbonyl center can react by substitution of the alcohol group with many nucleophiles, such as amines, alkoxylates, and hydroxylamines. Under basic or acidic conditions, esters can also hydrolyze.^402,451^ The volatility of many carboxylic acid esters must be considered in quantitative measurements^452^ (Figure 57).

**Figure 58:**
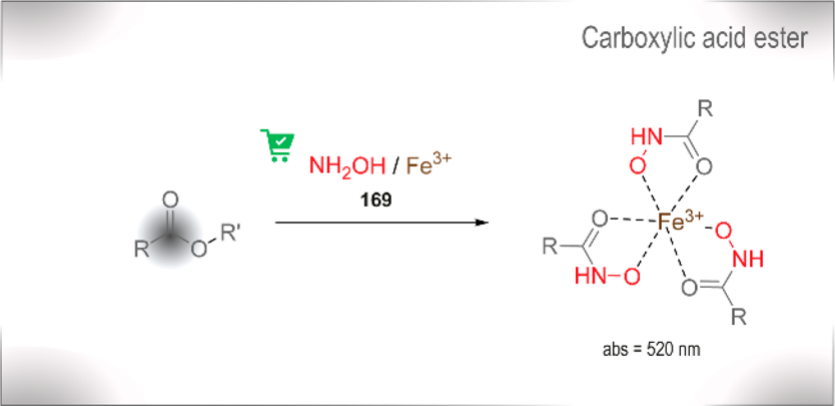
Chromogenic detection of carboxylic acid esters *via* the formation of the hydroxamic acids and subsequent complexation with Fe^3+^ resulting in purple-colored ferric hydroxamates.

The only direct colorimetric approach for the detection of carboxylic acid esters is based on the conversion to hydroxamic acids with hydroxylamine hydrochloride **169** in the presence of a base (Figure 58).^453−455^ The subsequent addition of ferric chloride under acidic conditions results in the formation of a ferric hydroxamate complex, which has a typical magenta color.^453,454^ We described a similar principle for the detection of carboxylic acids (see above).

Esters are inherently reactive toward hydroxylamine (*cf*. section 11) and do not need activation. Buckles and Thelen tested the scope and limitations of this approach with various aromatic and aliphatic esters. Besides simple carboxylic esters, also polymer esters and glycerides of fatty acids were detected. Carbonates, chloroformates, sulfonates, urethanes, and esters of inorganic acids gave negative results. Coloration was also observed in the presence of other electrophiles, including aldehydes, trihalomethyl compounds, imides, nitriles, and amides.^453^ Although only certain amides were shown to give a positive colorimetric response, an extraction step might be useful before analysis to reduce false-positive hits from endogenous proteins.

Based on the same concept, Löbs *et al*. developed a method for the rapid screening of ethyl acetate production by four different strains of *Kluyveromyces marxianus* growing on various C5, C6, and C12 carbon sources. The formed esters were extracted with hexane and subsequently converted to ferric hydroxamates at room temperature. The absorbance was measured at 520 nm. All strains exhibited the highest ethyl acetate production employing the C6 sugars glucose and fructose. The assay was also validated for ethyl butyrate, ethyl hexanoate, isoamyl acetate, and ethyl octanoate.^456^

## 13 Chromo- and Fluorogenic Probes for Epoxides

Epoxides react with various nucleophiles, oxidizing, and reducing agents due to its high polarity and strain of the three-membered ring. Nucleophilic ring-opening reactions, leading to 1,2-disubstituted products, are of particular synthetic interest.^457,458^ Possible products include: amino alcohols, glycol ethers, alcohols, hydroxynitriles, and chlorohydrins.^459^

**Figure 59:**
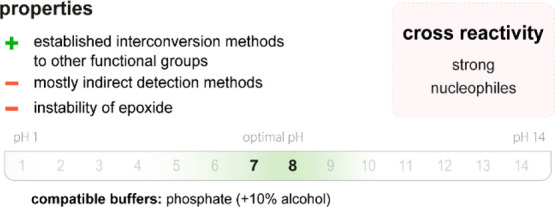
(top) Summary of general properties of epoxide sensors as well as expected cross-reactivity during screenings. (bottom) Compatible pH range and buffers in reported epoxide-selective assays. Bold pH numbers indicate the most commonly used pH values.

Epoxides are used as industrial intermediates in the production of pharmaceuticals,^460−463^ soaps,^462^ lubricants,^461^ cosmetics,^461,462^ and surfactants.^461^ Enantiopure epoxides are particularly valuable chiral functional groups for the production of more complex compounds.^464−466^ Simple epoxides, such as ethylene oxide or propylene oxide, are of interest for the production of ethylene glycols, ethanolamines, ethylene glycol ethers, and propylene glycols, polyether polyols, and propylene glycol ethers.^467,468^ Enzymatic methods for epoxide synthesis include the oxidation of alkenes by monooxygenases (MO), the conversion of halohydrins by halohydrin epoxidases, and the use of epoxide hydrolases.^216,469^

**Scheme 19:**
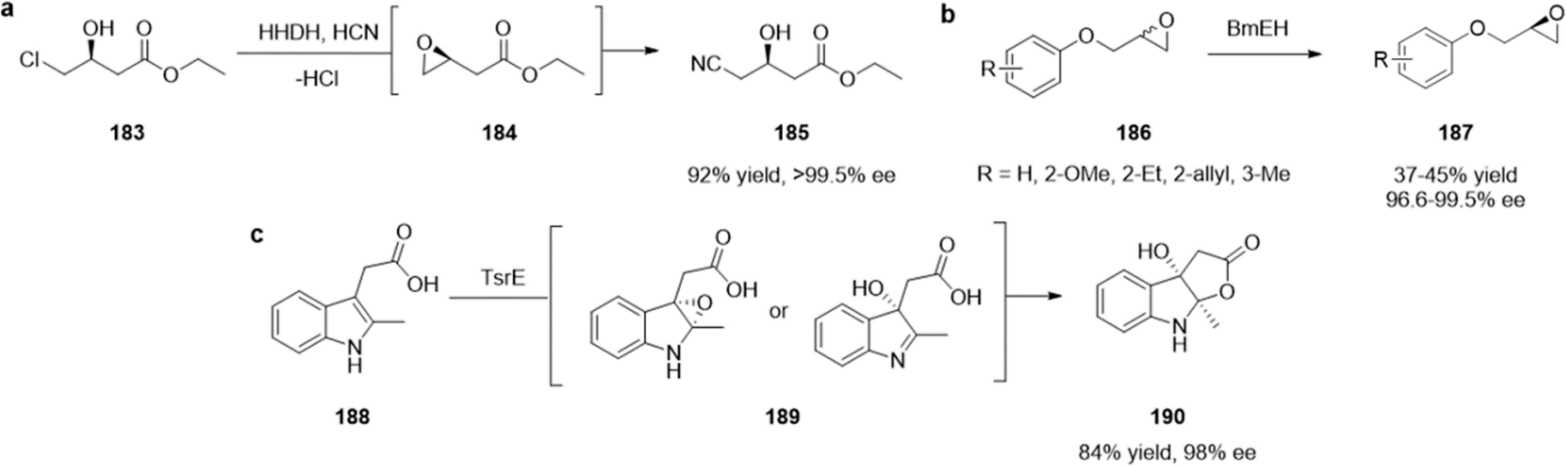
Representative Selection of Enzymatic Transformations for the Production of Epoxide-Containing Products

Halohydrin dehalogenases and epoxidases catalyze the reversible enantioselective dehalogenation of vicinal halohydrins **183** to the corresponding epoxides. Ma *et al*. employed halohydrin dehalogenase (HHDH, EC 3.8.1.5) in the second step of a two-step biocatalytic process to synthesize hydroxynitriles, which were used as intermediates in an environmentally friendly synthesis of atorvastatin. They exploited the promiscuitiy of halohydrin dehalogenases, accepting cyanide as a nucleophile: after formation of epoxide **184**, cyanide readily opened the ring and gave the corresponding β-hydroxynitrile **185** (Scheme 19a). Iterations of DNA shuffling increased the enzyme activity >2500-fold over the wild type, and ethyl (*R*)-4-cyano-3-hydroxybutanoate **185** was obtained in 92% yield.^470^

Kong *et al*. created a library of variants of the epoxide hydrolase from *Bacillus megaterium* (BmEH) to expand the substrate scope from phenyl glycidyl ether toward bulkier epoxides. By mutation of two active site residues, variants with an improved activity (6–340-fold) for nine β-blocker precursors were discovered. Resolution of the racemic epoxide precursors **186** yielded the (*S*)-epoxides **187** in 37–45% yield and 96.6–99.5%*ee* (Scheme 19b).^471^

Lin *et al*. reported a method for the oxidative dearomatization of indoles yielding asymmetric furoindolines using the flavin-dependent monooxygenase TsrE. The enzyme catalyzed the 2,3-epoxidation of 2-methyl-3-indole-3-acetic acid **188** and the subsequent epoxide opening of **189** in the synthesis of (3a*S*,8a*R*)-3a-hydroxy-8a-methyl oxofuroindoline **190**, which was obtained in 84% yield and 98%*ee* (Scheme 19c).^472^

Assays for epoxides are usually based on nucleophilic ring-opening reactions, and detection often happens indirectly. They are mostly based on the conversion of epoxides to aldehydes, thiols, or diols and the application of aldehyde-, thiol-, or diol- detection methods. Detection might be complicated by instability (*e.g*., to hydrolysis), side reactions,^466,473^ and volatility.^458,467,474^ Epoxides can undergo reactions with hemoglobin or nucleic acids and are therefore associated with mutagenicity, carcinogenicity, and genotoxicity^475^ (Figure 59).

### 13.1 Nucleophilic Ring-Opening by Pyridine Derivatives

**Figure 60:**
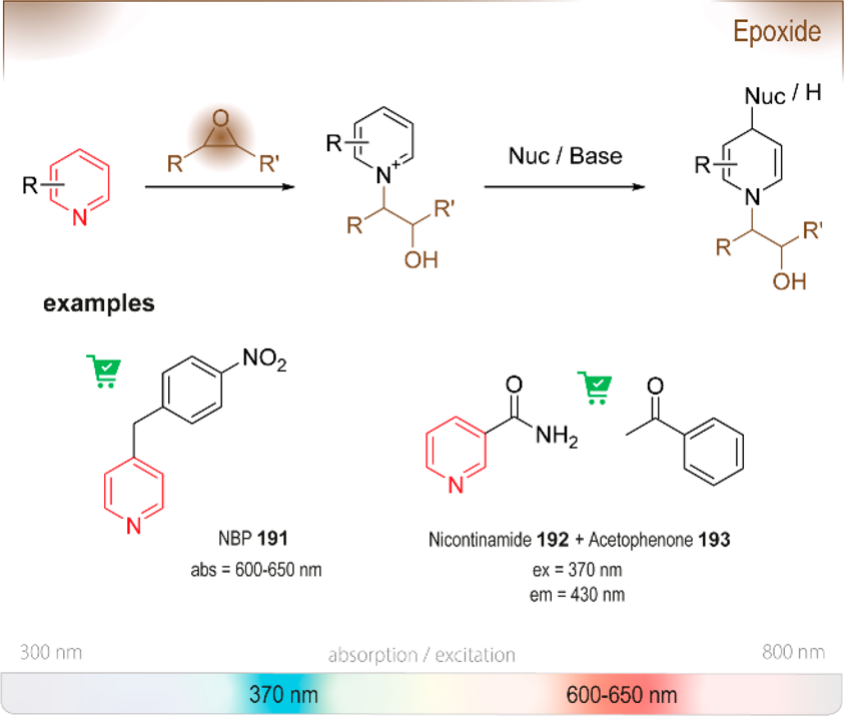
Summary of chromo- and fluorogenic turn-on probes for the selective detection of epoxides based on ring-opening reactions by nucleophilic pyridine derivatives. Abs: wavelengths typically used for UV absorbance measurements. Ex or em: wavelengths typically used for excitation and emission in fluorescence measurements. The wavelengths of probes in this section are shown in the strip at the bottom representing the visible spectrum.

Various epoxide assays are based on ring-opening reactions by nucleophilic pyridine derivates, resulting in colorimetric or fluorometric signals (Figure 60). The commercially available reagent 4-(*p*-nitrobenzyl)pyridine (NBP, **191)** is often applied to detect epoxides.^461,463,475−478^ The nucleophilic substitution reaction of **191** with epoxides results in an uncolored intermediate, which can subsequently be converted to the blue-colored dihydropyridine using base. Changes in absorption in the range of 560–620 nm have been used for read-outs.^460,461,475,477^ Zocher *et al*. developed a HTS assay with **191** for microtiter plates to screen for activity of an epoxide hydrolase.^460^ Alcalde, Farinas, and Arnold also used an NBP-based HTS assay for directed evolution to improve styrene epoxidation by cytochrome P450 BM-3 139-3.^461^ Probe **191** can also be used to detect organophosphorus compounds, ethylenimines, and other alkylating reagents, and side reactions with these compound classes might interfere with the detection of the epoxides.^463^

**Scheme 20:**
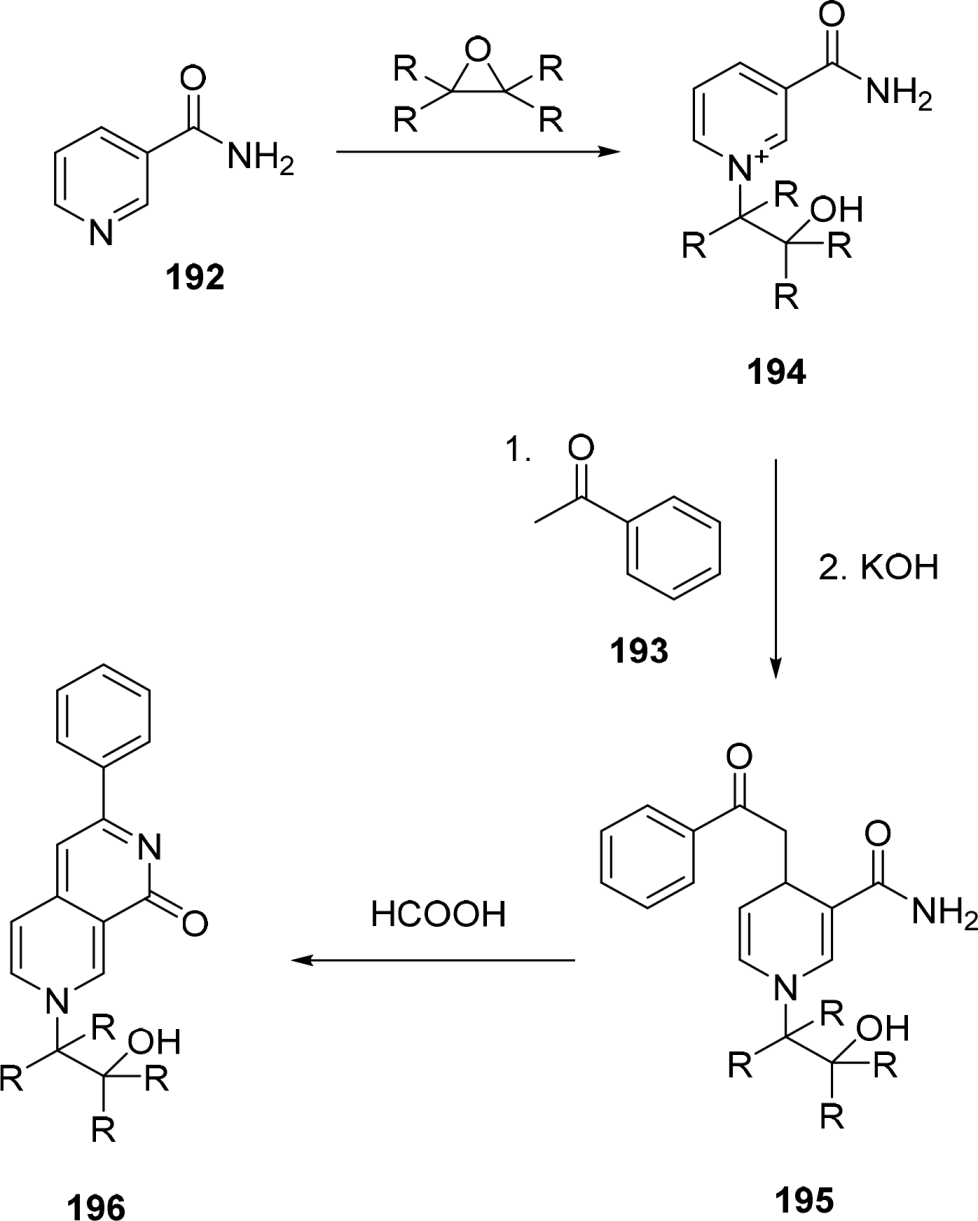
Sensing Mechanism for Epoxides Employing the Combination of Nicotinamide **192** and Acetophenone **193**

Because **191** is insoluble in water,^475^ Nelis and Sinsheimer described a similar fluorometric detection method with high sensitivity and solubility in aqueous systems. Their approach was based on the alkylation of nicotinamide **192** by aliphatic epoxides (Scheme 20). The alkylated amides **194** were converted to dihydropyridine derivatives **195** with acetophenone **193** under basic conditions, followed by a cyclization triggered by formic acid, which resulted in the formation of fluorescent products **196**. This method is not specific for epoxides; it detects various electrophilic compounds. Fluorescence of NADP^+^, itself an alkylated nicotinamide, might interfere, too.^476^

### 13.2 Indirect Detection

**Figure 61:**
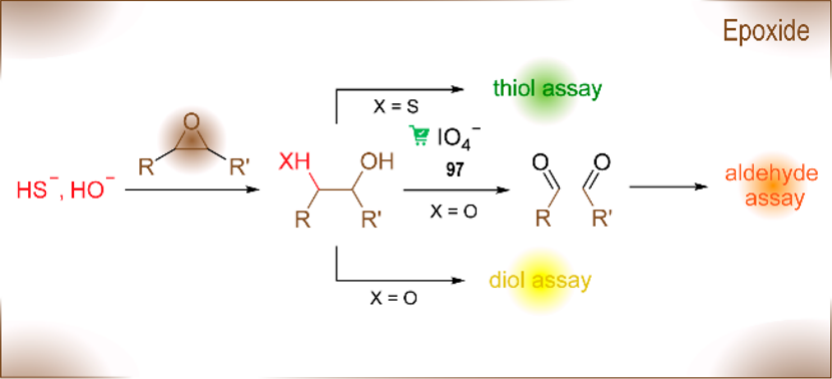
Selective detection of epoxides based on indirect detection methods after initial nucleophilic ring-opening reactions with hydroxide or bisulfide. The resulting intermediates can be detected with thiol sensing assays (*cf*. section 15), diol sensing assays (*cf*. section 8), or by using a similar method discussed for the indirect detection of diols by oxidative cleavage with metaperiodate **97** and subsequent detection using aldehyde-sensing assays (*cf*. section 9).

In many cases, the detection of epoxides is done indirectly by conversion to other functional groups and subsequently detecting those (Figure 61). Sano and Takitani developed a two-stage approach to detect epoxides in ethanolic aqueous solutions. The epoxides were converted to the respective *S*-alkylated derivatives by ring-opening with sodium sulfide. The resulting thiolates could be efficiently labeled by using a system known for fluorogenic isoindole formation employing *o*-phthalaldehyde **22** and taurine (*cf*. section 6.3).^479^

Another approach for the indirect detection of epoxides is based on their conversion to diols and the subsequent application of diol-sensitive assays. Doderer *et al*. described a colorimetric HTA that could be applied for all epoxides with at least one hydrogen substituent. The epoxides were converted to the diols using epoxide hydrolase, which were subsequently cleaved by periodate **97** to the corresponding aldehydes and/or ketones. The formed aldehydes could then be detected by Schiff’s reagent **123**. The absorption of the resulting magenta dye was measured at 560 nm.^264^ Doderer and Schmid reported another concept for diols based on oxidative cleavage with periodate to the corresponding carbonyls. Titration of the residual periodate **97** with carboxyfluorescein resulted in a detectable decrease in fluorescence.^270^ Some of the carbonyl species resulting from diol cleavage already have a strong UV absorbance, allowing their direct detection. Mateo *et al.* used this property for the determination of styrene oxide by detecting benzaldehyde at 290 nm after hydrolysis and cleavage. They used this method for the fast determination of activity of epoxide hydrolase.^483^

Halohydrin dehalogenases are commonly used as enzymatic catalysts to obtain epoxides from aliphatic or aromatic halohydrins. This and the reverse reaction is accompanied by a change in pH, which enables the indirect detection of epoxides using pH indicators, such as phenol red or bromothymol blue.^480,481^ Other assays make use of the direct change in UV absorption or fluorescence upon the conversion of epoxides to their corresponding diols.^474,482^ Wixtrom and Hammock reported two such spectrophotometric assays.^474^

## 14 Chromo- and Fluorogenic Probes for Phenols

Phenols and their derivatives are an important structural motif ubiquitous in nature in the form of catechols, quinones, lignans, flavones, or in polymeric form in lignin, flavolan, or tannins, among others. These hydroxylated aromatic structures are also crucial in industry (epoxide and phenolic resins, azo dyes, explosives) and as biologically active ingredients (drugs, herbicides).^484^

**Figure 62:**
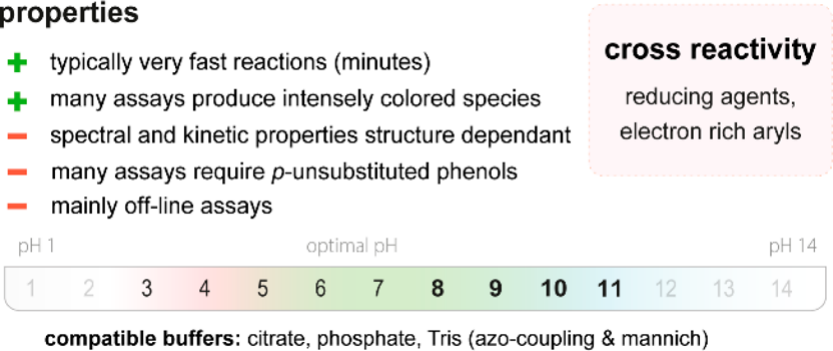
(top) Summary of general properties of phenol sensors as well as expected cross-reactivity during screenings. (bottom): Compatible pH range and buffers in reported phenol-selective assays. Bold pH numbers indicate the most commonly used pH values.

Synthesis of hydroxylated aromatic compounds^485^ typically requires harsh conditions, which has motivated a shift to enzymatic production of this class of compounds.^486^

With the emergence of novel biocatalysts, namely mono- (MO), di- (DO), or peroxygenases (PO) as well as tyrosinases (TYR), which enable aromatic hydroxylation, the scientific community has strived to discover and develop improved enzymatic catalysts.^486^ Lan Tee and Schwaneberg summarized the developments for directed evolutions of oxygenases, its success stories, and challenges in a recent review.^487^

**Scheme 21:**
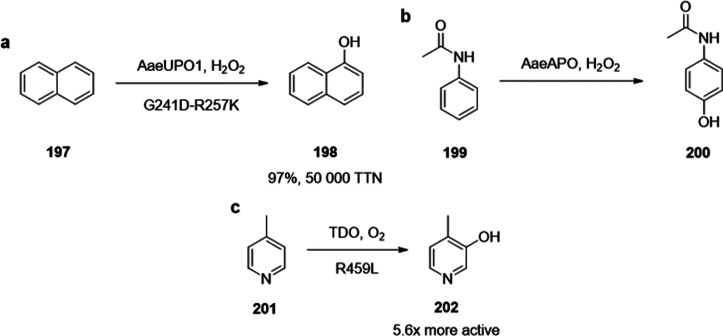
Representative Selection of Enzymatic Transformations for the Production of Phenol-Containing Products

Among many examples for the success of these transformations (Scheme 21), Molina-Espeja *et al*. applied directed evolution strategies for the optimization of an unspecific peroxygenase from *Agrocybe aegerita* (AaeUPO1), engineered for the synthesis of 1-naphthol **198**.^488,682^ The resulting double mutant (G241D-R257K) enabled the regioselective synthesis with a total turnover number of 50 000. The wild-type has also been efficiently applied for the hydroxylation of acetanilide **199** (among other relevant precursors), resulting in the selective production of acetaminophen (paracetamol) **200**.^489^ The group of Arnold evolved a toluene dioxygenase to improve the turnover of 4-picoline **201**, resulting in the discovery of the single-point mutant R459L with 5.6-times higher activity than the wild type.^490^

Phenols are more acidic than alcohols because the anion formed in deprotonation is stabilized by the conjugated electron system of the aryl. The delocalized charge increases the nucleophilicity and the antioxidant properties of phenols (stabilization of radicals compared to benzene).^492^ Their qualities as antioxidants are often used as a means to quantifying phenol content in terms of total antioxidant capability (AOC).^493^

Several assays have been developed in the food and live sciences due to the necessity for the assessment of antioxidant properties: the Folin–Ciocalteu (FC) test,^494−496^ ABTS (2,2′-azino-bis(3-ethylbenzothiazoline-6-sulfonic acid) radical scavenging,^497^ ORAC (oxygen radical absorbance capacity),^498^ FRAP (ferric-reducing antioxidant power),^499^ CuPRAC (cupric ion reducing antioxidant capacity),^499^ TEAC (trolox equivalent antioxidant capacity),^500^ and DPPH (1,1-diphenyl-2-picrylhydrazyl) radical scavenging.^497^ None of these established methods are recognized as universal,^499,501,502^ and the correct selection for the study of AOC remains difficult.

Reducing power of the matrix can be problematic in detecting phenols, particularly in biological media with many reducing compounds (*e.g*., ascorbic acid, sulphites, reducing sugars).^503^ Hence, reaction-based methods that integrate the phenol into a chromo- or fluorogenic product have proven superior in sensitivity.^504^ Due to the direct conjugation of the phenol structures into the final chromophores, large differences in absorbance can be expected for each analyte (Figure 62).

### 14.1 Electrophilic Aromatic Substitution (S_EAr_) with Activated Amino Groups

**Figure 63:**
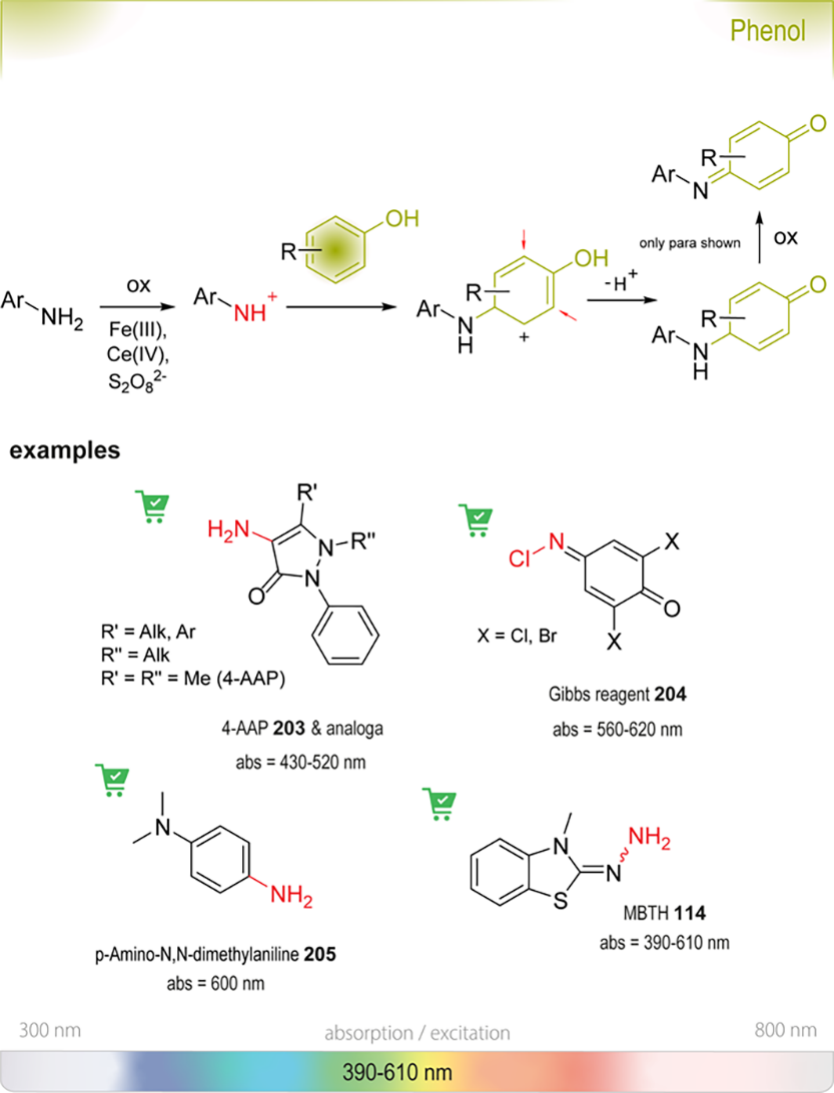
Summary of chromogenic turn-on probes for the selective detection of phenols based on the electrophilic aromatic substitution with oxidized amino derivatives. Abs: wavelengths typically used for UV absorbance measurements. Ex or em: wavelengths typically used for excitation and emission in fluorescence measurements. The wavelengths of probes in this section are shown in the strip at the bottom representing the visible spectrum. Alk = alkyl, Ar = aryl.

Most methods rely on trapping the nucleophilic phenol or phenolate with a positively charged probe in an electrophilic aromatic substitution (Figure 63). The subsequent oxidation of the dienone typically triggers the large change in color (and/or intensity) and increases the stability of the products (iminoquinones). The most widely applied method uses commercially available 4-aminoantipyrine (4-AAP, **203**), initially reported by Emerson in 1943.^505^ Its oxidative coupling to phenols produces an intensely amber to red colored complex,^493,506,507^ which is most sensitive at pH 9–10.^506,508^ Appropriate buffering is necessary to reduce noise, which was typically caused by a distinct drop in pH upon the addition of reagents.^508,509^

Typically, K_3_[Fe(CN)_6_] or K_2_S_2_O_8_ are used as oxidants, although recently systems consisting of iron(III)-octacarboxyphthalocyanine and *t*-BuOOH have been demonstrated as fast, water-soluble oxidants.^510^ Synthetic optimizations of the parent structures have been proposed with the exchange of alkyl and aryl-side substituents, with little improvement over the 4-AAP assay.^509,511^ Only phenols with hydrogen, halides, or alkoxy substituents in *para*-position are detectable.^509^ This assay system has been used for the colorimetric determination of phenolic compounds in pharmaceutical preparations,^506^ tetracyclines,^512^ and many other examples.^508,513^

Probe **203** was also used in directed evolution of hydroxylases toward aromatic and heterocyclic compounds^504,514^ and esterases by the group of Schwaneberg.^515^ Reliable detection of phenolic substrates at 50–800 μM was enabled after 30 min incubation. Choi *et al*. applied the probe in HTS and directed evolution of a tyrosine phenol-lyase.^516^

Probe **204** (“Gibbs reagent”) is a prominent example in this category. It is already preoxidized (aniline is trapped as chloroimide), thus does not require an oxidant.^517^ The chloroimide is rapidly hydrolyzed at pH > 8.5, yielding the free imine. Both forms react exclusively at the *para*-position of the phenol ring. As with **203**, only hydrogen, halide, or alkoxy substituents are tolerated.^518,519^ Even though decomposition of the quinoneimine was shown to occur in alkaline medium, the formation of the product is favored, and the reaction is not hampered by prolonged reaction times. Full conversion of phenol was reported using a 10-fold excess at pH 10 after approximately 10–20 min at μM concentrations.^520^

**204** was used by Ang *et al*. for the directed evolution of an aniline dioxygenase for bioremediation purposes, targeting the resulting catechol derivatives.^521^ Arnold’s group used it in HTS of dioxygenase activity using solid-phase digital imaging^488^ and for the evolution of a toluene dioxygenase toward 4-picoline (4-methylpyridine).^490^ Here, the initially formed *cis*-dihydrodiol product was not detected and deemed to be unstable. Its spontaneous rearomatization by dehydration, forming the 3-hydroxy-4-picoline **202**, allowed the detection with **204** without using dihydrodiol dehydrogenase or decreasing of pH, which is typically required for this transformation. Bornscheuer *et al*. also used the reagent to test the selectivity of 28 lipases and esterases in the hydrolysis of butanoates of *ortho*-, *meta*-, or *para*-substituted phenols.^522^

Taking advantage of the well-studied spectroscopic properties of phenol blue, Altahir *et al*. developed a phenol probe based on *p*-amino-*N*,*N*-dimethylaniline **205**.^523^ Using a similar protocol as for the 4-AAP assay, the two-electron oxidation of the aniline with K_3_[Fe(CN)_6_] under basic conditions enabled the detection of eugenol contained in personal-care products.

MBTH **114**, known as a tool to detect aldehydes (*cf*. section 9.1), has also been efficiently used for the derivatization of phenols.^524^ Da Silva Granja *et al*. evaluated **114** among other methods for the determination of total phenol content (**203**, FC assay, ABTS assay, DPPH **109** assay) and found it to be equally useful.^503^ The reaction with **114** works in dilute H_2_SO_4_^503,524^ and at at pH 8–9,^525,526^ contrary to the typically neutral to basic conditions required by reagents **203** and **204**. Oxidations are typically performed with K_3_[Fe(CN)_6_], FeCl_3_, or K_2_S_2_O_8_. Oxidative coupling mediated by laccase or horseradish peroxidase have also been reported.^527,528^ Reactions with phenols are not limited to the *para*-position, as *ortho*-positions have also been successfully derivatized. Side reactions can be expected with amines, electron-rich aromatic compounds, and aldehydes (*e.g*., reducing sugars). Nolan and O’Connor applied the assay for the quantification of a toluene-4-monooxygenase activity producing *para*-substituted phenols. These were detected after tyrosinase-assisted transformation of the products to 4-substituted catechols.^529^

### 14.2 Azo Coupling

**Figure 64:**
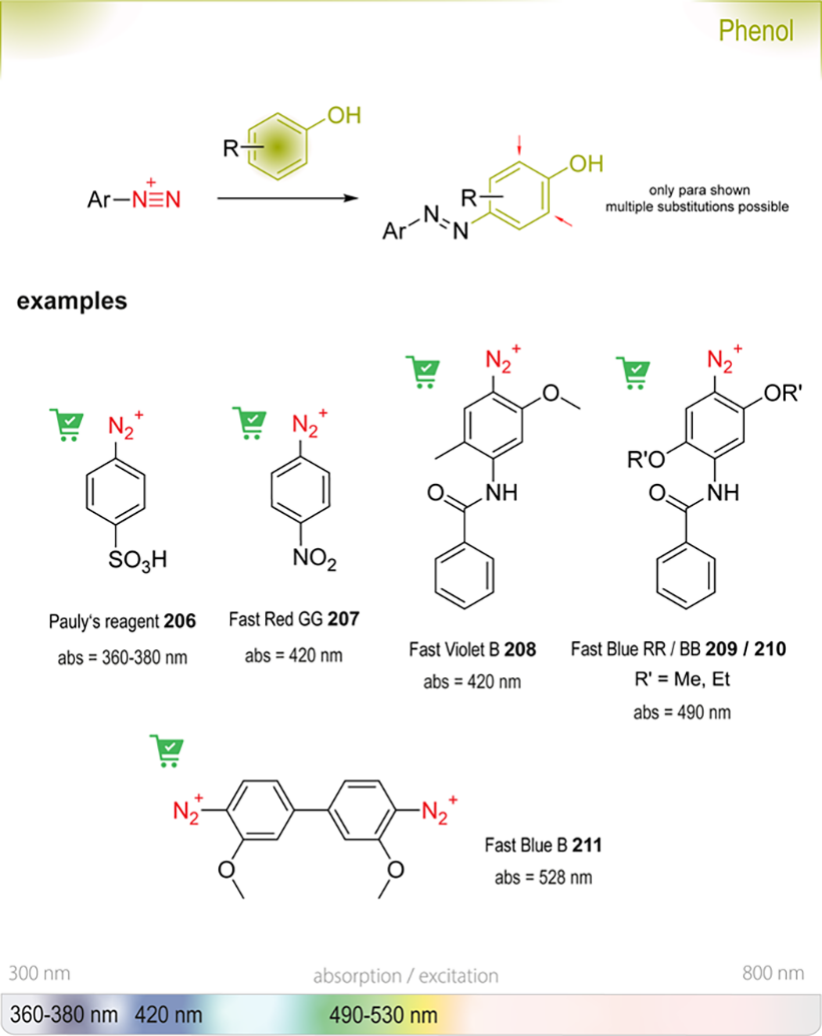
Summary of chromogenic turn-on probes for the selective detection of phenols based on the formation of azo-dyes. Abs: wavelengths typically used for UV absorbance measurements. The wavelengths of probes in this section are shown in the strip at the bottom representing the visible spectrum.

Phenols can be detected using azo-coupling reactions (Figure 64). Herein, aromatic diazonium salts react as strong electrophiles with activated arenes in electrophilic aromatic substitutions at the *ortho*- or *para*-unsubstituted positions.^530^ The resulting azo compounds often have vivid colors due to their large conjugated systems and are widely used as dyes and pigments.^531,532^

Urbányi and Mollica tested 27 differently substituted aniline derivatives and established a strong dependency of the sensitivity of these probes on the functionality and substitution pattern of their aromatic systems. Electron-poor diazonium salts were shown to form more intensely colored products after fixed reaction times (6 min). Although no underlying kinetic study was undertaken and single points were used as read-outs, the authors concluded that electron-poor systems are the better analytical probes for rapid screenings.^533^

Diazotized sulfanilic acid **206** (Pauly’s or Ehrlich’s diazo reagent) has been used as early as 1883 by Ehrlich for the detection of bilirubin in urine samples^534^ and has found many applications in the detection of phenolic derivatives since then.^535−538^ Its electron-poor character, however, makes it unstable; fresh preparation before use is necessary.

The emergence of commercially available, stabilized diazonium salts, either by decoration of the aromatic core with electron-donating functionalities or complexation with suitable anions, such as zinc chloride or tetrafluoroborates, enabled broad usage of these reagents (“Fast” salts) for the detection of phenols in food, water, and biological samples.^538−541^ The high stability of electron-rich arenes has been systematically studied by Schotten *et al*.^542^ by comparing differently substituted anilines. The formation of reversibly cleavable triazenes with secondary amines was reported to further increase stability.

Currently, many differently substituted diazonium salts are commercially available, with a broad scope of applications in different buffer systems. Johnston and Ashford studied the nine most widely used “Fast” reagents for the quantification of activity of hog liver esterase, using α-naphthyl acetate as substrate.^543^ Fast Red GG **207** (*p*-nitrobenzenediazonium tetrafluoroborate) and Fast Violet B **208** emerged as the best combination of reagents to cover a combined pH range of 3–9.5. Coupling reactions with these probes were reported to be complete in under 5 s at approximately 1 mM concentrations using 5-fold excess naphthol, allowing the determination of initial rates.

It has been reported for **207** (but not for **208**) that enzymatic activity might be inhibited by certain diazonium salts or their stabilizers.^544^ Purification of commercial material or validation of activity by an orthogonal method might prove useful. Lugg tested combinations of 24 airborne phenols and anilines with 25 Fast salts and thus showed the differences in spectral responses of these assays.^545^ The author concluded that probe **210** has universal applicability and a low background. The salt has also been used to quantify phenol in air and urine^546^ and *o*-phenylphenol in aqueous samples.^547^

Structural analogues with red-shifted absorption maxima (Fast Blue RR **209**, BB **210**, and B **211**) have also been used to detect phenol in samples.^538,539^ Probe **211** was used by the group of Wood for the directed evolution of a toluene *o*-monooxygenase.^548^ Among other protocols for HTS for aromatic hydroxylations using **203** and **204**, Otey and Joern presented a concise summary for the detection of phenols, also using **208** with a large set of differently substituted phenols.^513^

### 14.3 Mannich Type Reaction

**Figure 65:**
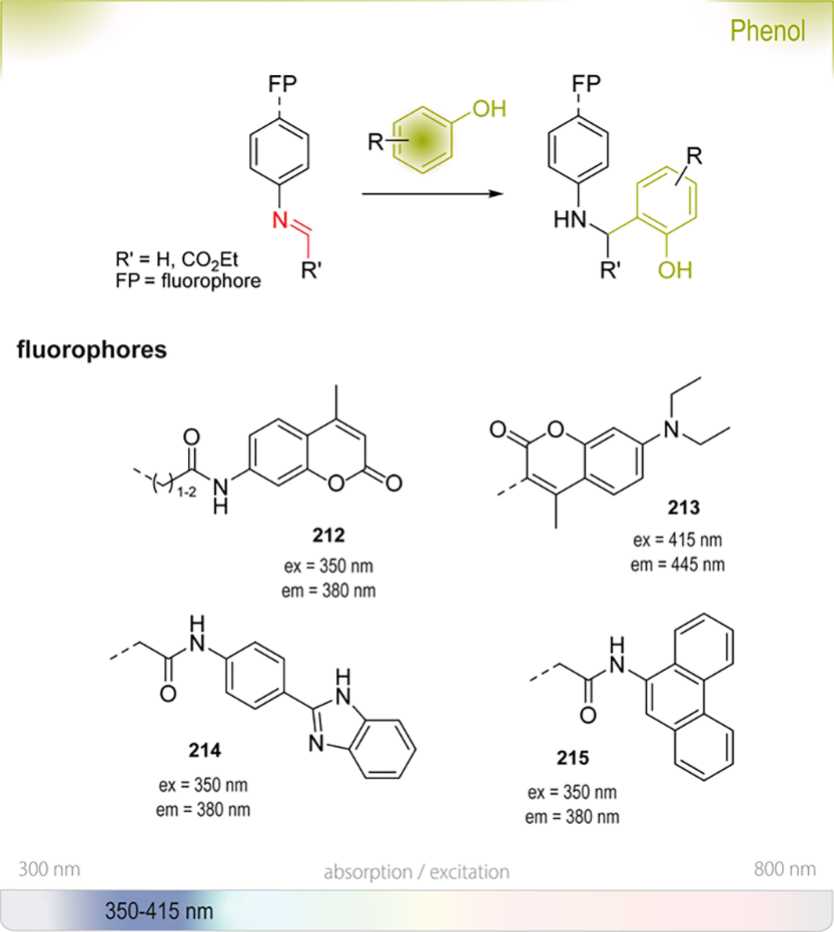
Summary of fluorogenic turn-on probes for the selective detection of phenol based on Mannich-type reactions. Ex or em: wavelengths typically used for excitation and emission in fluorescence measurements. The wavelengths of probes in this section are shown in the strip at the bottom representing the visible spectrum.

A new approach for a fluorogenic detection of phenols is based on Mannich-type reactions of activated arenes (Betti reaction; Figure 65).^549,550^ The group of Tanaka reported the reaction of phenols and peptides bearing tyrosine units with cyclic imines to proceed without the use of catalysts from pH 2–10.^551^

They designed several fluorogenic reporters **212**–**215** based on this mechanism.^683^ Substitution patterns of the attacking arene are crucial: only *ortho*-phenol substituted product gave the desired increase in fluorescence. The authors hypothesized that intramolecular interaction between the phenolic hydroxyl group and the amine, or π–π stacking interactions between the phenol and fluorophore aryl moieties, were essential for fluorescence to arise. Using methyl 4-hydroxyphenylacetate and their coumarin probe **212**, they demonstrated an approximately 100-fold increase in fluorescence after 70 min at pH 7. The slow reaction required a 1000-fold excess of phenol at μM concentrations. No further investigations and optimization of the reaction conditions were undertaken to test the limits of the system.

## 15 Chromo- and Fluorogenic Probes for Thiols

Thiols are essential components of biological systems.^552^ Compounds such as glutathione (GSH), cysteine (Cys), homocysteine (Hcy), and coenzyme A are involved in various biological processes including regulation of the oxidation–reduction states of the cells,^553^ protein synthesis,^554^ signal transduction,^555^ detoxification of xenobiotics,^556^ fatty acid biosynthesis,^557^ and posttranslational modifications.^558^ Aberrations of thiol levels in cells are considered to be related to diseases such as Parkinson’s disease,^559^ Alzheimer’s disease,^560^ osteoporosis,^561^ and cancer.^562^ Therefore, the development of selective and sensitive detection methods for thiols has become an important research field in clinical diagnosis.^559,562^

Other thiols, *e.g*., thiophenols, are used in the agrochemical industry for pesticide syntheses,^563,564^ in the pharmaceutical industry,^565^ and in the food industry as flavoring ingredients (*e.g*., 2-ethylthiophenol with a roasted, smokey odor).^566^

Thiols are nucleophilic and react with electrophiles. In this regard, their reactivity is highly correlated with their p*K*_a_ value, as the compounds become stronger nucleophiles when deprotonated. A typical p*K*_a_ of aromatic thiols is ∼6.5, and ∼8.5 for aliphatic thiols.^567,568^ Fluorescence-based assays can be applied for thiol detection and quantification, in addition to chromatography, UV/vis spectroscopy, potentiometry, and mass spectrometry.^569−571^ Many of the fluorescence assays use the nucleophilicity of the thiols in reactions such as the Michael addition, cleavage of benzenesulfonamides and benzenesulfonates, cyclization with an aldehydes, cleavage of (*O*-, *S*-, *Se*-)phenyl ethers, cleavage of disulfides, or make use of the high affinity of thiols toward metals.^251,567,572^

**Figure 66:**
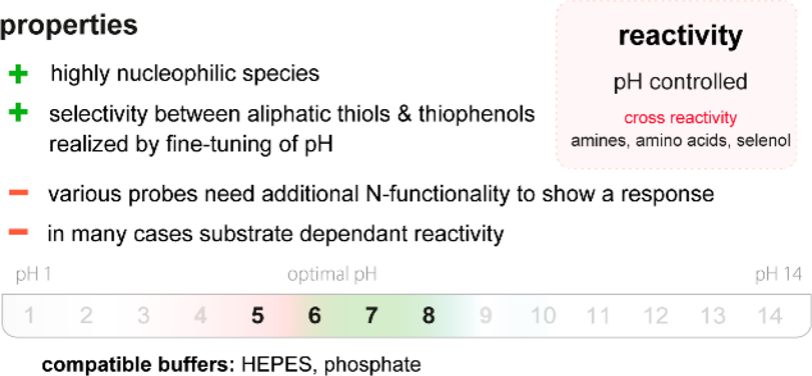
(top) Summary of general properties of thiols, reactivity trends toward thiol-selective probes, and expected cross-reactivity. (bottom) Compatible pH range and buffers in reported thiol-selective assays. Bold pH numbers indicate the most commonly used pH values.

Potential challenges in the development/application of fluorescent probes for thiols include cross-reactivity with other nucleophiles (*e.g*., cyanides,^573,574^ amines, and amino acids),^575^ requirements for a narrow pH range,^576^ and slow reaction rates under physiological conditions (Figure 66). Thiols occur in a large range of concentrations in cells, making selective detection even more difficult; Bennett *et al*. quantified metabolite concentrations in *E. coli* and found GSH at 17 mM and Hcy at only 0.37 mM.^29^ Additionally, selectivity between thiols can be an issue (*e.g*., GSH *vs* Cys/Hcy, or the distinction between Cys/Hcy, GSH, and H_2_S).^566,568,577−579^ Various probes thus require substrates with an amino group in addition to the thiol functionality to produce a color/fluorescence response. In the following section, those probes are labeled with a blue “NH_2_-required”.

These probes discriminate GSH and Cys/Hcy based on a reaction sequence: nucleophilic substitution reaction or conjugate addition, and subsequent intramolecular thiol to amine rearrangements or cyclizations. These S–N rearrangements for Cys/Hcy are favored compared to GSH due to lower energy transition states.

Some assays that discriminate Cys/Hcy against GSH are based on the cyclization of Cys and Hcy with aldehyde-containing probes, forming stable five-and six-membered rings. Most development is focused on detecting the thiols already mentioned (Cys, Hcy, GSH, and coenzyme A). Hence many assays were only tested with these substrates.

### 15.1 Nucleophilic Aromatic Substitution (S_NAr_)

**Figure 67:**
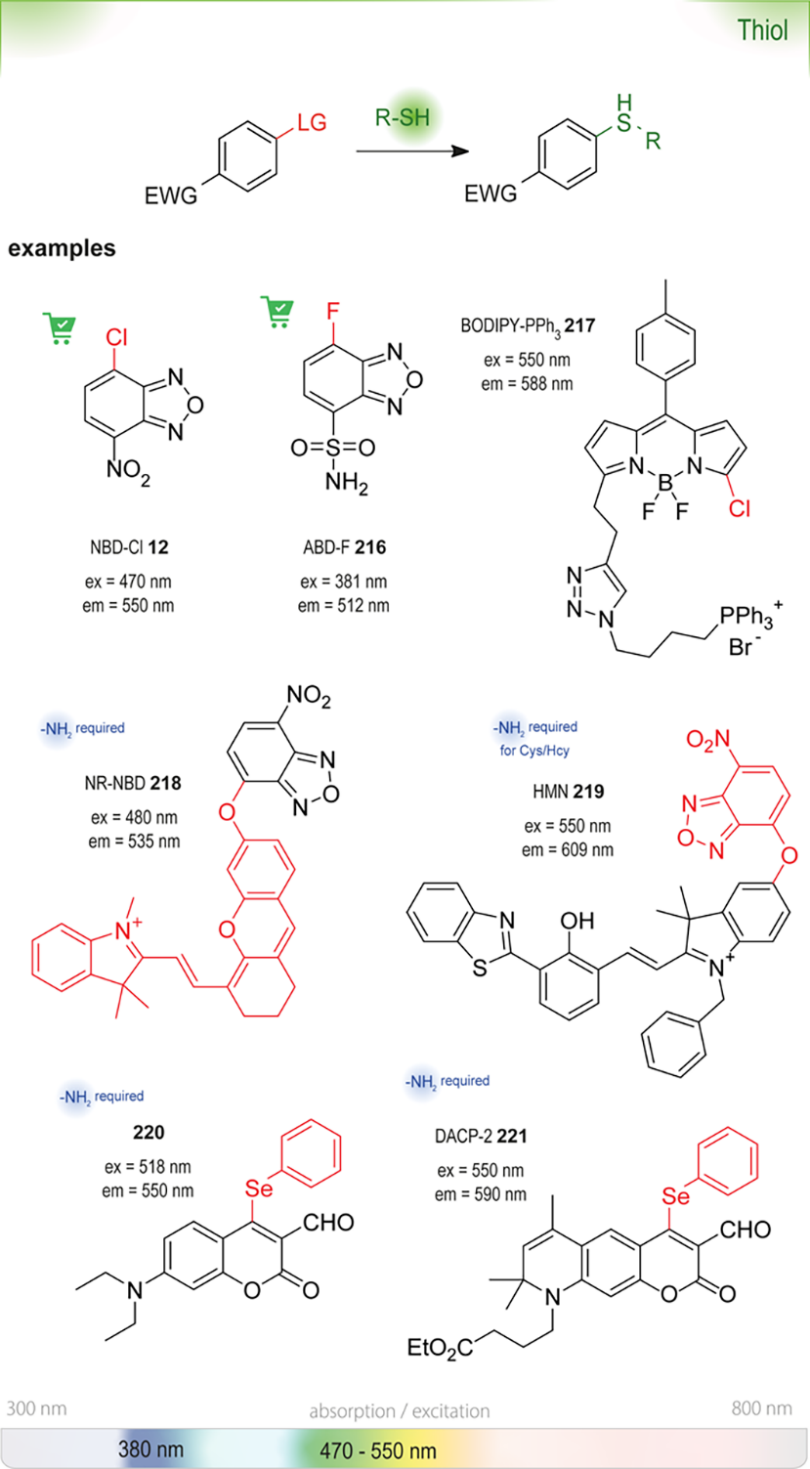
Summary of fluorogenic turn-on probes for the selective detection of thiols based on nucleophilic aromatic substitution reactions (S_NAr_) with aromatic systems containing electron-withdrawing groups (EWG). Ex or em: wavelengths typically used for excitation and emission in fluorescence measurements. The wavelengths of probes in this section are shown in the strip at the bottom representing the visible spectrum.

Thiolates readily react with electron-deficient centers *via* nucleophilic aromatic substitution (S_NAr_). Electron-withdrawing groups at the aromatic ring provide the needed electron deficiency for the substitution reaction. Common leaving groups for the thiol-mediated S_NAr_ include phenylsulfonyl moieties, halogenides, *O*-phenyl ethers, and *S*-phenyl ethers (Figure 67).^580^

In 1968, Ghosh and Whitehouse observed that benzofurazans, *e.g*., 4-chloro-7-nitro-2,1,3-benzofurazan (NBD-Cl, **12**), showed a fluorescent response upon reaction with amino groups. They also noticed that thiol derivatives were slightly fluorescent.^79^ Since then, **12** and (aminosulfonyl)-7-fluoro-2,1,3-benzoxadiazole (ABD-F, **216**), which was developed some years later by Toyo’oka and Imai,^581^ have been applied for the detection of thiols and activity screenings of carnitine palmitoyltransferase, citrate synthase, and acetylcholinesterase. In these cases, the commercially available probes showed a fluorescent signal upon reaction with the enzymatically released thiols.^582,583^

**Scheme 22:**
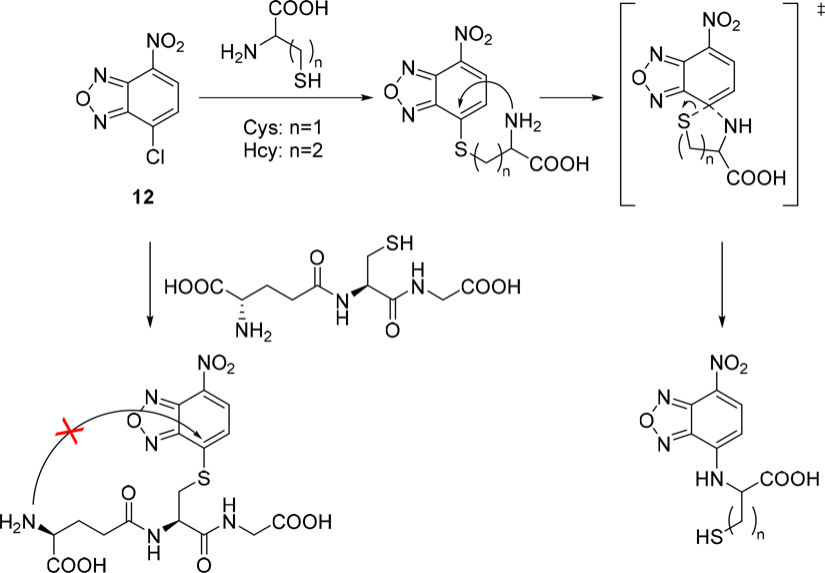
Proposed Mechanism for the Reaction of NBD-Cl **12** with Cys, Hcy, and GSH^584^

Niu *et al*. showed that **12** could be used for the selective and rapid detection of Cys/Hcy in the presence of GSH under mild conditions in an aqueous acetonitrile (25% ACN). The selectivity for of Cys/Hcy is potentially linked to an intramolecular displacement of the thiolate, leading to the formation of the *N*-substituted NBD derivative with strong fluorescence. In contrast, the *S*-substituted derivative with GSH only gave negligible fluorescence. The observation that the *S*-substituted and not the *N*-substituted derivative was obtained in the case of GSH was explained by comparing transition states (showing an unfavorable macrocyclic transition state with GSH, and favorable five- or six-membered rings as transition states with Cys and Hcy). An possible explanation for the faster response with Cys than Hcy could be that the formation of five-membered rings is faster than six-membered ones (Scheme 22).^584^

**Figure 68:**
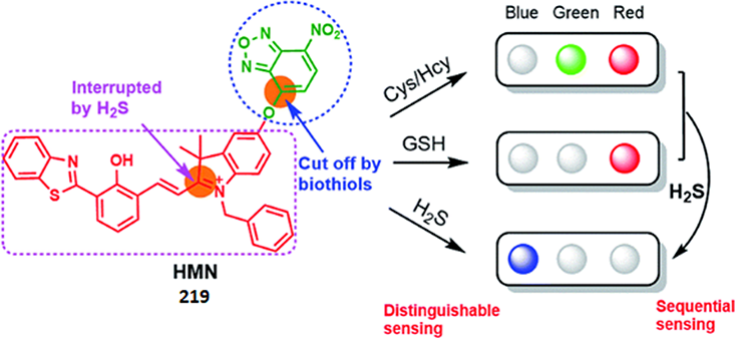
Design of the probe HMN **219** and the proposed fluorescence signal changes in response to individual or sequential detection of thiols with three well-defined emission bands. Reproduced with permission from ref (568). Copyright 2017 Royal Society of Chemistry.

The ratiometric BODIPY probe (BODIPY-PPh_3,_**217**) was used for the selective detection of GSH over Cys/Hcy by nucleophilic substitution of chlorine with the thiolate. Distinction between GSH and Cys/Hcy was possible because GSH gave a highly fluorescent, sulfur-substituted conjugate, but Cys/Hcy formed weakly fluorescent, amino-substituted BODIPY derivatives upon rearrangement.^585^ By adding a triphenylphosphonium moiety, it was possible to target the probe at mitochondria.^586^ Hu *et al*. made use of thiols’ ability to cleave phenyl ethers and developed a nonfluorescent probe with an NBD scaffold (NR-NBD, **218**) that enabled rapid discrimination (within a few minutes) between Cys/Hcy and GSH under aqueous conditions by using two excitation wavelengths, accompanied by a color change from blue to green.^587^ He *et al*. enhanced this idea and were the first that expanded the functionality with the probe HMN **219**, that simultaneously distinguished between Cys/Hcy, GSH, and hydrogen sulfide (Figure 68).^568^

*S*-Phenyl ethers and Se-phenyl ethers can also be used as leaving groups. The bond length of the carbon-heteroatom bond increases from oxyethers over thioethers to selenoethers, with a concomitant decrease in stability. The most stable bond (oxyethers) also makes the reaction rate of *O*-phenyl ether-based probes generally slow.^578,588,589^ However, rates can be increased by adding electron-withdrawing groups at the aromatic ring, for example, in nitrophenyl-*O*-ethers.^568,589^

In 2015, Kim *et al*. developed probe **220** for the selective and fast detection of GSH under physiological conditions. A phenylselenide linked to a coumarin probe was used as a leaving group. The electrophilicity of the probe was increased by installing two carbonyl groups adjacent to the reaction site, enabling a fast substitution reaction with the nucleophilic thiol. Subsequently, an intramolecular cyclization forming an iminium group was observed. With **220**, detection of GSH in an aqueous DMSO solution was possible within seconds.^590^ Later, the authors presented modifications of the probe by substituting the diethylamino group with an *N*-heterocyclic ring and introduction of an ethyl butanoate group. They envisioned that by restricting the free rotation of the amino group and the additional ethyl butanoate functionality, the low quantum yield and the poor water solubility of **220** would be improved. Although probe **221** (DACP-2) showed a slower response (about 10 min) than **220**, it indeed was better soluble in water and had a higher quantum yield (0.48 *vs* <0.001). It enabled the selective detection of GSH and Cys/Hcy.^591^

### 15.2 DNBS Cleavage *via* Nucleophilic Aromatic Substitution (S_NAr_)

**Figure 69:**
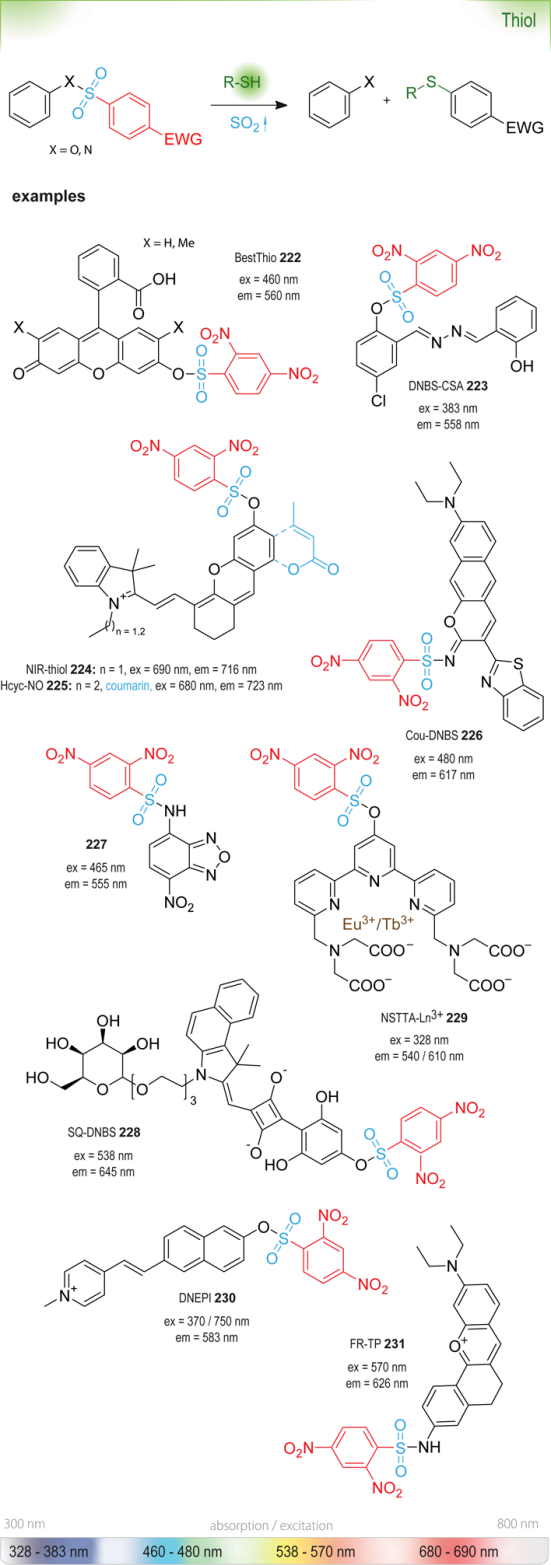
Summary of fluorogenic turn-on probes for the selective detection of thiols based on 2,4-dinitrobenzene sulfonyl (DNBS) cleavage by nucleophilic aromatic substitution reactions (S_NAr_) with aromatic systems containing electron-withdrawing groups (EWG). Ex or em: wavelengths typically used for excitation and emission in fluorescence measurements. The wavelengths of probes in this section are shown in the strip at the bottom representing the visible spectrum.

**Figure 70:**
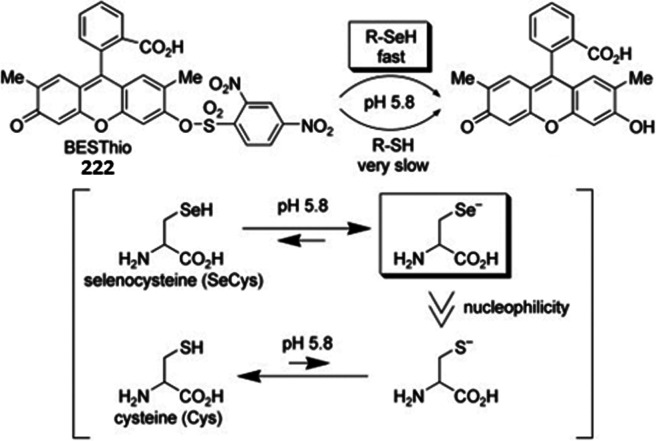
Fluorescent probe BESThio **222** and its discrimination between selenols and thiols. Reproduced with permission from ref (597). Copyright 2006 Wiley-VCH.

DNBS (2,4-dinitrobenzene sulfonyl) is a commonly used building block in thiol-sensitive probes. Upon reaction with thiolates, the benzenesulfonamide or benzenesulfonate bond is cleaved, SO_2_ is released, and fluorescence is restored (Figure 69).^592−596^ In 2005, Maeda *et al*. developed the DNBS-based probe BESThio **222**, which showed a high increase in fluorescence within minutes upon reaction with thiols under mild aqueous conditions (pH 7.4, 37 °C). At the same time, no fluorescence signal was observed with amines, such as ethylamine or piperidine, or alcohols, such as catechol or glucose, at pH 5.8–7.4. The authors demonstrated that their probe could be used as an alternative to the commonly used colorimetric Ellman’s reagent **232** and applied it in the fast screening of ChE (cholinesterase) inhibitors. Furthermore, they showed that their probe could detect selenols and distinguish between thiols and selenols by adjusting the pH from 7.4 to 5.8 (Figure 70).^597,592^

**Figure 71:**
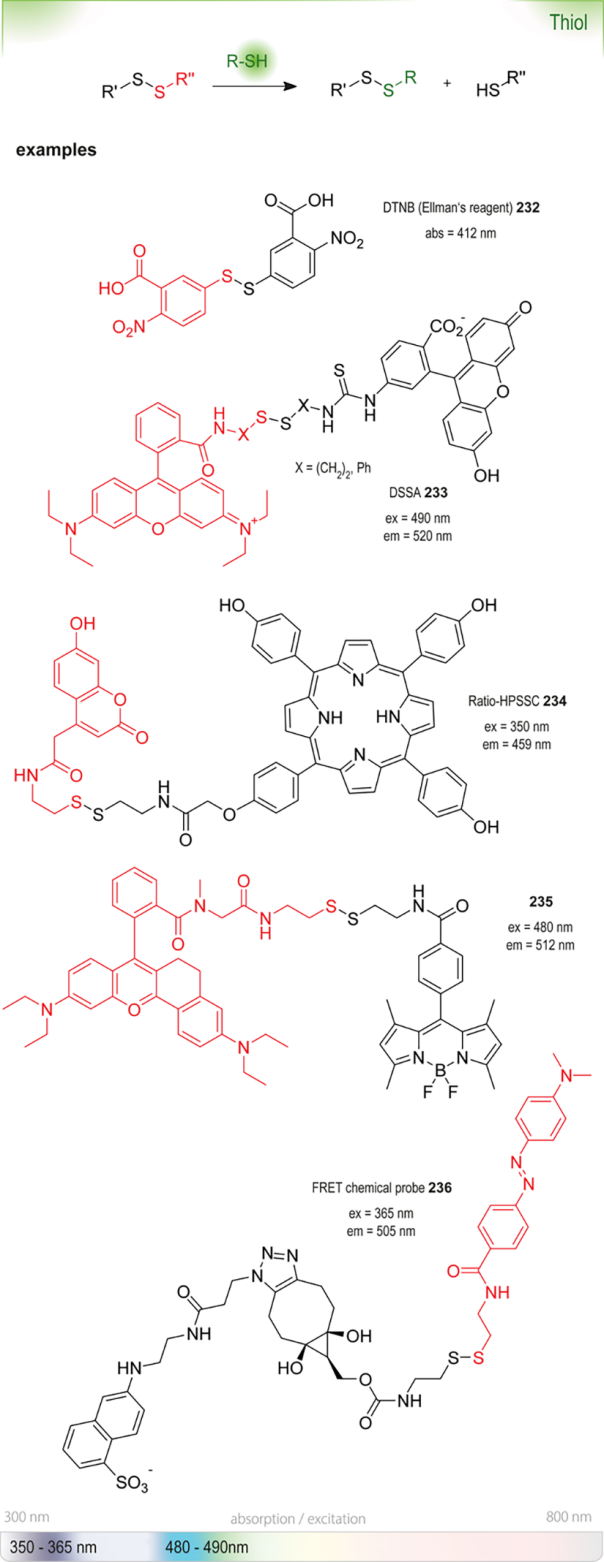
Summary of fluorogenic turn-on probes for the selective detection of thiols based on disulfide bond cleavage. Abs: wavelengths typically used for UV absorbance measurements. Ex or em: wavelengths typically used for excitation and emission in fluorescence measurements. The wavelengths of probes in this section are shown in the strip at the bottom representing the visible spectrum.

Peng *et al*. investigated methods to detect the activity of β-lactamase and reported an indirect detection approach based on aggregation-induced emission (AIE) and excited-state intramolecular proton transfer (ESIPT).^598^ Fluorophores that exhibit AIE fluoresce strongly in the solid state or as aggregates in solution. The AIE and ESIPT characteristics of salicylaldehyde azine-based fluorophores depend on their hydroxyl groups. In probe DNBS-CSA **223**, one hydroxyl groups was blocked by a 2,4-dinitrobenzenesulfonate group. The reaction of **223** with thiols led to their deprotection, which recovered fluorescence by AIE and ESIPT.

The indirect detection method by Peng *et al*. consisted of three steps. After the enzymatic cleavage of the β-lactam ring of the cefazolin substrate, a spontaneous elimination yielded a thiol, which was detected with DNBS-CSA by releasing CSA (salicylaldehyde azine derivative) and thereby enabling AIE and ESIPT fluorescence. This approach enabled the detection of β-lactamase activity under mild conditions in solution (detection limit 0.5 mU mL^–1^) and on paper-based test strips.^598^

Yuan *et al*. described the NIR-fluorescent probe NIR-Thiol **224**, with DNBS as a recognition moiety, for the detection and imaging of thiols in living mice. The probe does not fluoresce by itself and showed a fast response to μM concentrations of thiols. The addition of Cys in PBS buffer/ACN (v/v = 7:3; pH 7.4) resulted in a 50-fold increase in fluorescence, reaching the maximum within 60 s. Probe **224** was proven to be stable under assay conditions (24 h) and could be stored as a solid for more than one year.^599^

Challenges in clinical diagnostics motivated the development of fast fluorescent probes for thiols.^582,600−602^ Lin *et al*. developed the NIR DNBS-based probe Hcyc-NO **225** that allowed the almost instantaneous detection (<5 s) of thiols relevant for evaluating cellular oxidative stress, including GSH, Cys, and Hcy. The assay works under mild, aqueous conditions (PBS/DMSO 8:2, pH 7.4) and showed detection limits of 0.08 μM (Cys), 0.20 μM (Hcy), and 0.11 μM (GSH). Although probe **225** readily reacts with thiols from pH 4–8, it was observed that more basic conditions resulted in increased fluorescence.^603^

Lu *et al*. reported a far-red fluorescent probe based on the iminobenzo[*g*]coumarin dye and DNBS as recognition site (Cou-DNBS, **226**), which reacted quickly even with low amounts (nM) of thiols under basic or neutral conditions. It was used for monitoring intracellular fluctuations in thiol levels, demonstrated by incubating HeLa cells that had been pretreated with H_2_O_2_ with the probe and imaging the development of thiol concentration over time. Fluorescence increased from zero (or insignificant noise) to strong signals within 20 min, indicating thiol recovery.^600^

Development of probes that are specific to a particular thiol is challenging. Thiophenols are toxic to aquatic organisms and animals and harmful to human health after prolonged exposure. Therefore, probes that can detect thiophenols and differentiate between those and other thiols are of interest.^593,604−606^ Jiang *et al*. developed the essentially nonfluorescent NBD-Cl **12** modified probe **227**, with a benzenesulfonamide reaction site. The selectivity for thiophenols was achieved by relying on the p*K*_a_ differences between aliphatic thiols (∼8.5) and thiophenols (∼6.5). Thus, thiophenolates could rapidly cleave the benzenesulfonamide bond under neutral conditions, whereas the aliphatic thiols remained in the less reactive neutral form. No fluorescence response was observed for aliphatic thiols, such as *t*-butyl thioalcohol, GSH, and Cys, or other nucleophiles (*e.g*., NaCN, BnNH_2_). The water-soluble probe was synthesized in two steps from **12** with moderate yield and gave a more than 50-fold increase in fluorescence intensity within 10 min of addition of thiophenol or derivatives (two equivalents), such as 4-methyl-, 4-methoxy, or 4-chlorothiophenol. A lower fluorescence enhancement was measured with 4-nitrothiophenol, explained by the strong electron-withdrawing properties of the nitro-group.^593^

The same concept for discrimination between aliphatic and aromatic thiols was utilized by Xiong *et al*. They developed the fluorogenic and chromogenic probe SQ-DNBS **228**, with a rapid response (≤10 min) and high selectivity toward thiophenols. Their squaraine-based probe with DNBS as the reactive site was used to detect and quantify thiophenols in water samples. The far-red/NIR probe showed a high sensitivity (LOD: 9.9 nM), good photostability (>1 h under constant irradiation), and could be applied under neutral or slightly basic aqueous conditions without the need for a cosolvent. Furthermore, the color change from pink to blue upon the addition of thiophenols enabled the convenient detection by the naked eye.^607^

Lanthanide complexes can be useful to eliminate background fluorescence in biological samples emanating from flavins, porphyrins, or nicotinamide adenine dinucleotides. Whereas the biogenic fluorescent signals typically have short decay times (<10 ns), the photoluminescence (PL) of lanthanide complexes (*e.g*., with Eu^3+^ or Tb^3+^) takes 10–1000 μs to decay (1000 to 100 000 longer lasting). By starting the measurement only after the autofluorescence has already decayed, a better signal-to-noise ratio can be obtained. With the DNBS-based Eu^3+^/Tb^3+^ complex NSTTA-Ln^3+^**229**, Dai *et al*. quantified thiols at μM concentration in aqueous samples and living cells, using this time-gated acquisition.^595,608,609^

Shang *et al*. and Fan *et al*. synthesized two-photon fluorescent DNBS probes. The probe DNEPI **230**, by Fan *et al*., exhibited a fast response (about 2 min) and a high selectivity for Cys over other thiols like Hcy and GSH.^572,610^ The probe FR-TP **231**, developed by Shang *et al*., showed high selectivity toward thiophenols, a 155-fold fluorescence enhancement with an emission in the far-red region, and a rapid response (complete within minutes). Probe **231** enabled the detection of thiophenol poisoning in an animal model for thiophenol inhalation, using a two-photon fluorescence microscope.

### 15.3 Disulfide Cleavage

Thiols can cleave disulfide bonds, which can be used to detect them (Figure 71).^594^ Chromogenic DTNB **232** (“Ellman’s reagent”) has been frequently used to quantify thiols and enzymes.^611−613^ When thiols react with **232**, the disulfide bond is cleaved, and chromogenic 5-thio-2-nitrobenzoate with an absorption maximum at 412 nm is released. Instability at pH > 8 creates issues with high background signal and interferences with absorption from other biomolecules (*e.g*., hemoglobin) and led to the development of other reagents.^612,614−618^

Based on the work by Adamczyk and Grote, Pullela *et al*. synthesized fluorescein- and rhodamine-containing probes (DSSA, **233**) with a disulfide linker that could be used for detecting thiols *in vivo* and *in vitro* in the mM range under aqueous conditions.^619^

Cao *et al*. synthesized the ratiometric fluorescent probe Ratio-HPSSC **234**, based on the tetrakis(4-hydroxyphenyl)porphyrin-coumarin scaffold to detect thiols under neutral conditions in ethanolic (50%) aqueous buffer solutions. A change in emission color from red to blue enabled the visual detection of thiols, with a detection limit for Cys at 0.73 μM. It was shown that the probe was suitable for imaging thiols in living cells.^620^

Wang *et al*. developed the ratiometric probe **235**, with a disulfide bond, which linked a BODIPY moiety as FRET donor and rhodamine as FRET acceptor. It showed a high sensitivity for GSH and enabled detection down to the low mM range.^621^

**Figure 72:**
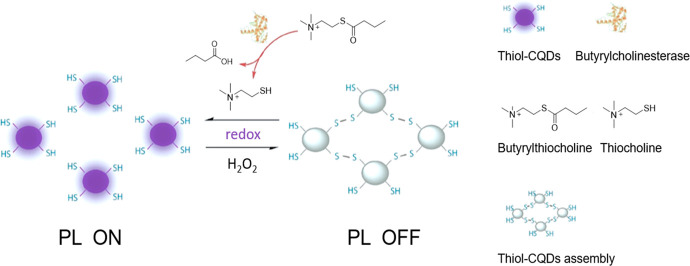
Schematic illustration of redox-controlled fluorescent nanoswitch and detection strategy for BChE activity based on thiol-triggered disulfide cleavage on fluorescent carbon nanoparticles. Reproduced with permission from ref (614). Copyright 2018 American Chemical Society.

Chen *et al*. pursued a different strategy using fluorescent carbon nanoparticles, so-called carbon quantum dots, which exhibit beneficial properties including tunable fluorescence emissions, easy functionalization, and high photochemical stability. They reported a nanoswitch based on fluorescent carbon nanoparticles, which had thiol groups covalently attached at their surface (Figure 72). These quantum dots showed a green fluorescence that vanished when the dots aggregated due to disulfide bond formation. In the presence of thiols, the disulfide bonds were cleaved and fluorescence was restored. This process proved to be reversible as the disulfide linkage and reaggregation could be triggered by H_2_O_2_. This concept was applied for evaluating the activity of butyrylcholinesterase (BChE) and for screening butyrylcholinesterase inhibitors using butyrylthiocholine iodide as a substrate. Conversion of the substrate yielded butyrate and thiocholine, which reacted with the quantum dots and thereby restored the signal. The assay could also be used to quantify the activity of AChE by changing the substrate to acetylthiocholine chloride.^614,622^ Gong *et al*. developed another method to determine the activity of BChE and test its inhibitors. Their disulfide containing probe (“FRET chemical probe” **236**) enabled measuring BChE activity in serum directly without interference from GSH, explained by the probe’s steric hindrance.^615^

### 15.4 Diselenide Cleavage

**Figure 73:**
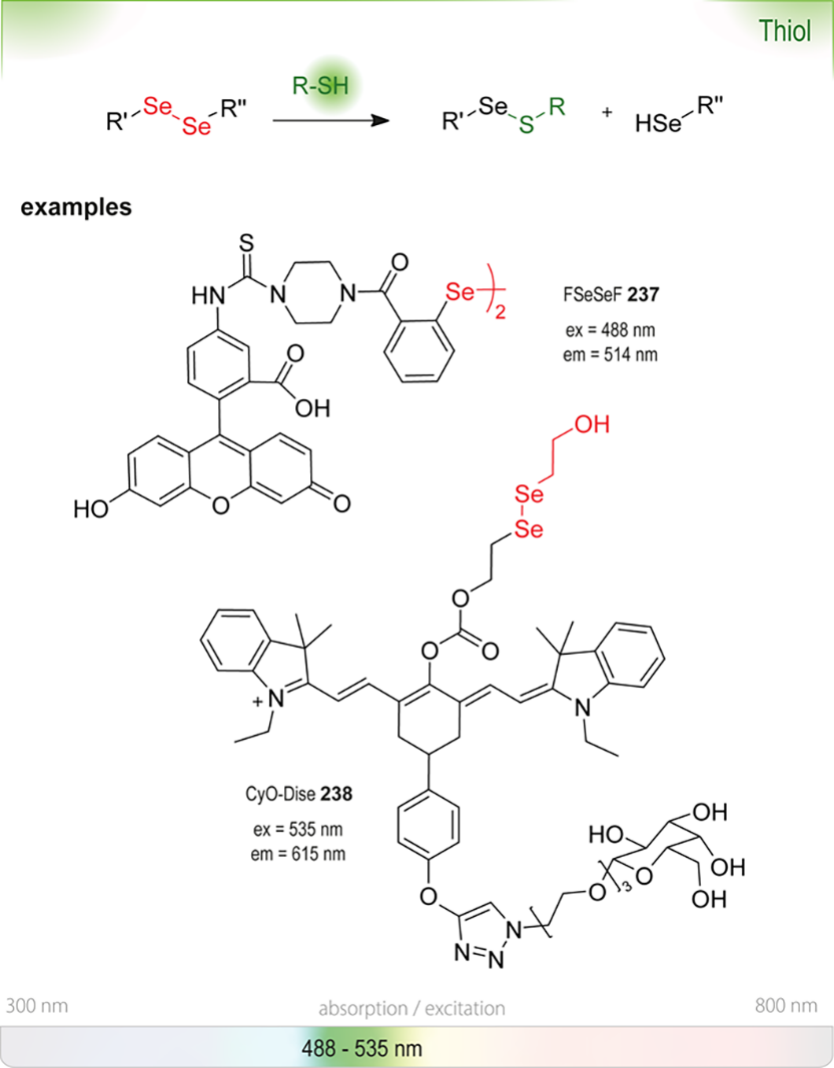
Summary of fluorogenic turn-on probes for the selective detection of thiols based on diselenide bond cleavage. Ex or em: wavelengths typically used for excitation and emission in fluorescence measurements. The wavelengths of probes in this section are shown in the strip at the bottom representing the visible spectrum.

Diselenides (R-Se-Se-R) can be used instead of disulfides as a reactive center to detect thiols. (Figure 73). Diselenide bonds have lower bond energies and are cleaved faster by the nucleophilic attack of thiols than disulfides.^623,624^ An notable toxicity of organoselenium compound should be expected due to their oxidizing as well as inhibitory properties for certain thiol-containing enzymes.^625^

Lou *et al*. developed the diselenide probe FSeSeF **237**, with two fluorescein moieties as signal reporters that were linked by a diselenide bond. Upon cleavage of the diselenide bond, the two fluorescein-containing moieties are released, resulting in strong fluorescence. Probe **237** showed high selectivity for thiols and rapid response (within milliseconds for GSH in PBS buffer pH 7.4). The reaction is reversible in the presence of H_2_O_2_, and the probe was thus used for the detection of redox changes mediated by thiols and ROS.^626^ Han *et al*. designed the diselenide-based fluorescent probe Cyo-Dise **238**, which showed high selectivity for GSH and could detect it within seconds in HEPES buffer (pH 7.4) by fluorescent response, and the color change from green to red. The ratiometric fluorescent probe **238** was applied for evaluating changes in GSH levels in HepG2 and HL-7702 cells under hypothermic and hyperthermic conditions and in HepG2 and HepG2/DDP xenografts on nude mice.^627^

### 15.5 Selenium–Nitrogen Bond Cleavage

**Figure 74:**
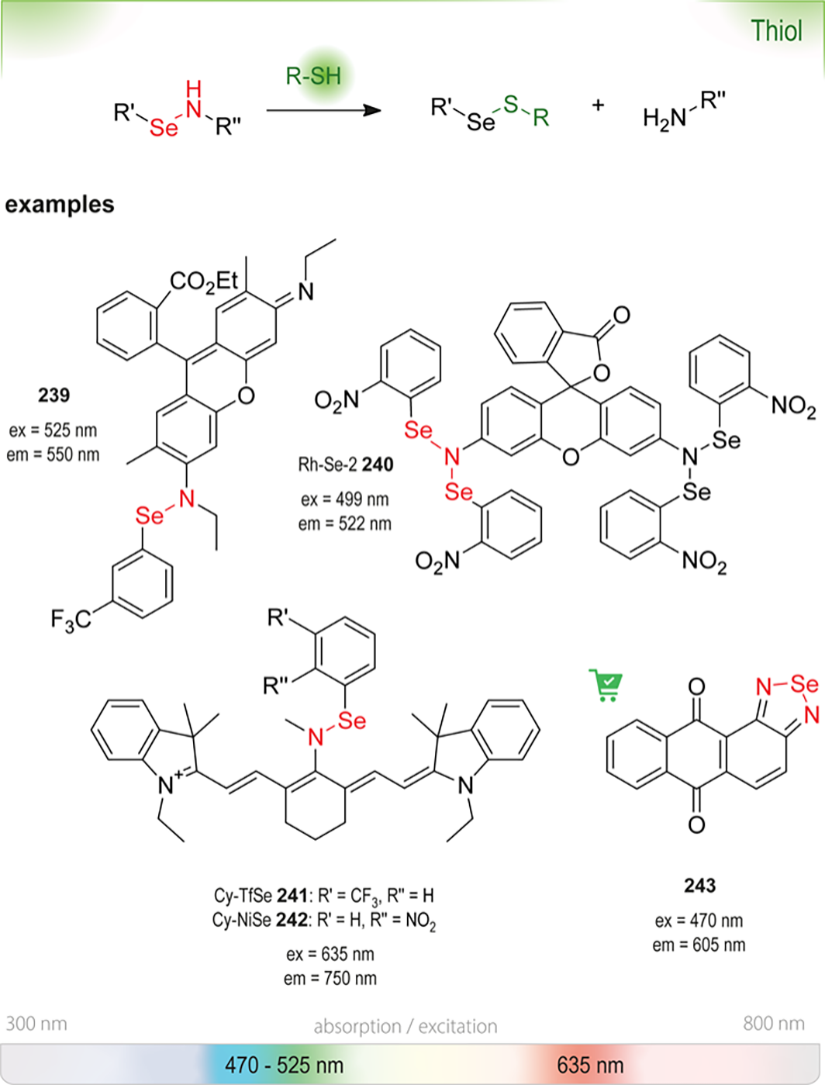
Summary of fluorogenic turn-on probes for the selective detection of thiols based on selenium–nitrogen bond cleavage. Ex or em: wavelengths typically used for excitation and emission in fluorescence measurements. The wavelengths of probes in this section are shown in the strip at the bottom representing the visible spectrum.in the strip at the bottom representing the visible spectrum.

Thiols are sufficiently nucleophilic to cleave selenium–nitrogen bonds. Based on this reactivity, thiol-detecting probes containing selenium–nitrogen linkages have been developed (Figure 74). In 2007, Tang *et al*. designed the weakly fluorescent rhodamine-based probe **239** with a Se–N bond, which releases the strongly fluorescent dye rhodamine 6G upon cleavage. They demonstrated that probe **239** could be used for the quantitative detection of GSH (LOD: 1.4 nM) in PBS buffer (pH 7.4). Interestingly, a higher sensitivity was observed for the protein-bound thiols in glutathione reductase or metallothionein than for free thiols, such as Cys, 2-mercaptoethanol, or dithiothreitol.^628^

The high noise and only moderate enhancement of fluorescence (about 6-fold) prompted Tang *et al*. to develop a better rhodamine-based probe, Rh-Se-2 **240**. Structural modification prevented an opening of the spirocycle under screening conditions, thereby reducing noise. Probe **240** reacted fast (within minutes) and selectively with thiols, with a >170-fold enhancement in fluorescence in aqueous solution. In addition to the turn-on response in fluorescence, the color changed from light yellow to yellow-green.^629^

**Figure 75:**
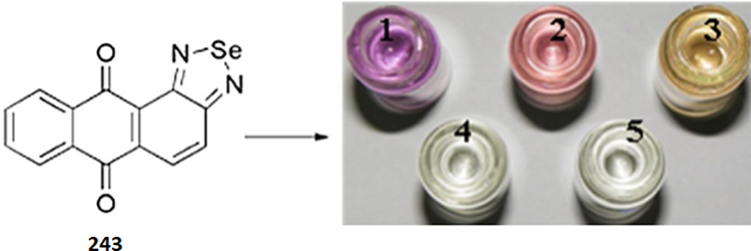
Structure and color changes of **243** in the presence of amino acids (1, Cys; 2, Hcy; 3, GSH; 4, none; 5, other natural amino acids). Reproduced with permission from ref (631) . Copyright 2018 Elsevier BV.

The cyanine-dye-based NIR probes Cy-TfSe **241** and Cy-NiSe **242** developed by Wang *et al*. showed a fast signal response with thiols in PBS buffer at physiological pH. Fluorescence of these dyes was stable from pH 4.0 to 8.6.^630^ Tian *et al*. designed the colorimetric and fluorescent probe **243**, with two Se–N bonds that allowed differentiation of Cys, Hcy, and GSH (Figure 75), whereas GSH and Hcy only cleaved one Se–N bond, Cys cleaved both. Differences in the absorption and fluorescence spectra allowed distinguishing between GSH, Hcy, and Cys simultaneously. Determination of the kinetic constants and equilibrium constants showed that the reactivity of Cys toward the probe is much higher than that of GSH and Hcy, which could explain that only the presence of Cys led to the cleavage of both bonds.^631^

### 15.6 Cyclization with Aldehydes

**Figure 76:**
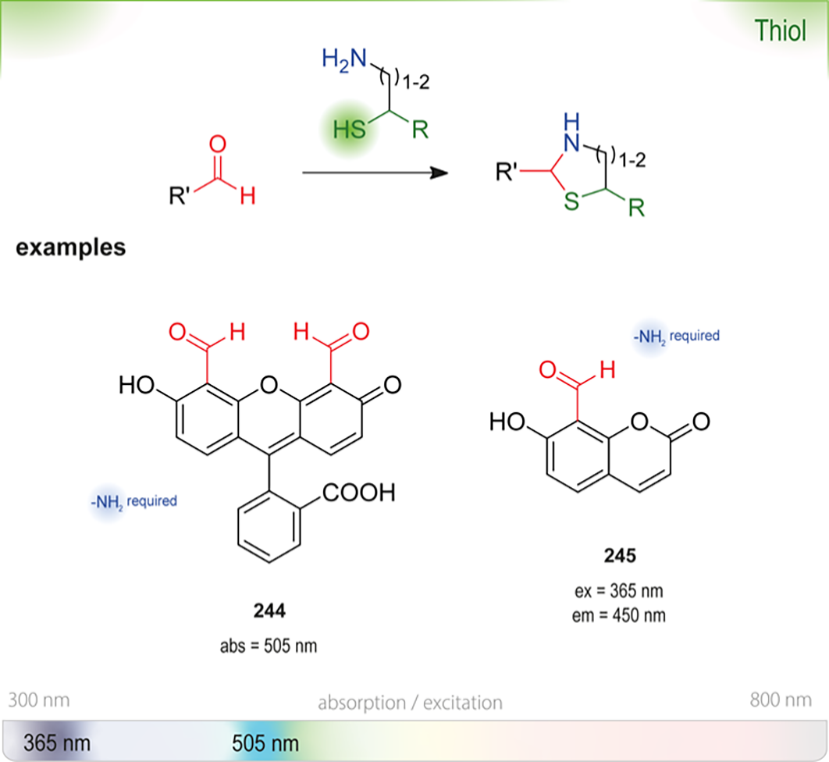
Summary of fluorogenic turn-on probes for the selective detection of thiols based on the cyclization with aldehydes. A subsequent cyclization *via* an additional NH_2_ moiety limits the application to analytes containing both functional groups. Abs: wavelengths typically used for UV absorbance measurements. Ex or em: wavelengths typically used for excitation and emission in fluorescence measurements. The wavelengths of probes in this section are shown in the strip at the bottom representing the visible spectrum.

1,2- and 1,3-Aminothiols can form five- or six-membered rings with aldehydes. Based on this mechanism, fluorescent probes with aldehyde moieties for the selective detection of cysteine and homocysteine have been reported (Figure 76).^575,632,633^ In 2004, Rusin *et al*. developed the xanthene-based probe **244**, bearing an aldehyde for colorimetric and turn-off fluorometric detection of Cys and Hcy in alkaline aqueous solutions (pH 9.5).^632^ The requirement for basic conditions prompted the development of other thiol detecting probes that could be used under physiological conditions. Lee *et al*. reported the coumarin-based probe **245** with a salicylaldehyde moiety, which showed high selectivity for Cys/Hcy and could be used at neutral pH.^634^

### 15.7 Conjugate Addition (1,4-Addition)

**Figure 77:**
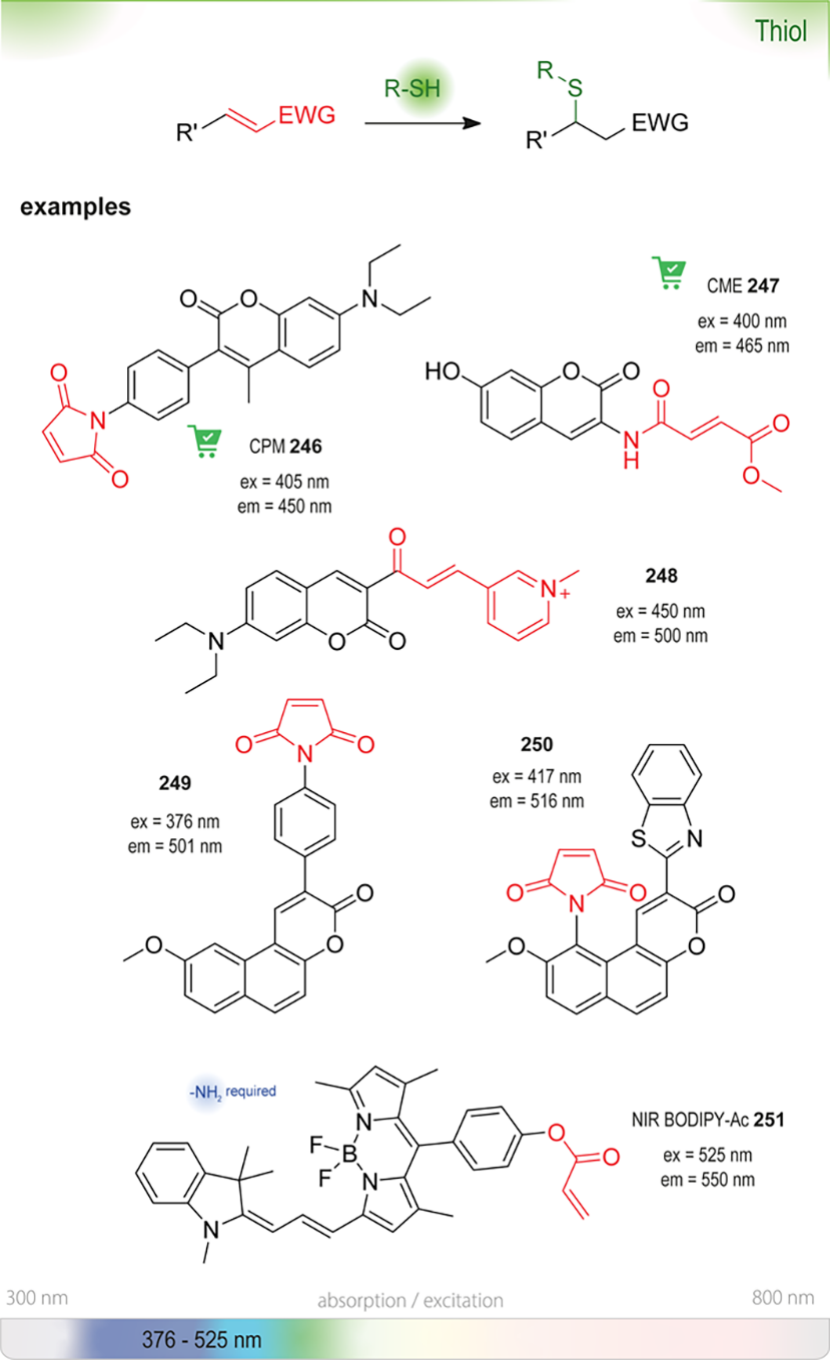
Summary of fluorogenic turn-on probes for the selective detection of thiols-based conjugate additions. Ex or em: wavelengths typically used for excitation and emission in fluorescence measurements. The wavelengths of probes in this section are shown in the strip at the bottom representing the visible spectrum.

Thiols readily undergo 1,4-additions to α,β-unsaturated carbonyl compounds due to their soft nucleophilicity. They are softer nucleophiles than amines and alcohols.^635−638^ Maleimides are frequently used as reactive centers for thiol-sensitive probes because they react selectively with thiols and quench fluorescence based on n−π* transitions or PET (Figure 77). Upon addition of the thiol, the quenching process is suppressed and fluorescence recovered.^635^

Commercially available *N*-(4-(7-diethylamino-4-methylcoumarin-3-yl)phenyl)maleimide (CPM, **246**) is often used to detect thiols.^639,640^ Aharoni *et al*. applied **246** in a HTS of serum paraoxonase (PON1) mutants to find variants with improved thiolactonase activity. The gene variants were transformed into *E. coli*, and the single cells were encapsulated in w/o/w (water-in-oil-in-water) droplets together with the substrate thiobutyrolactone and **246**. The droplets were sorted using FACS. With this method, they were able to discover variants with ∼100-fold higher catalytic efficiency than the wild-type PON1.^641^

Marcella and Barb presented a FabD (malonyl-coenzyme A transacylase) and a FabH (ketoacyl synthase III) assay that used **246** to detect the enzymatically released coenzyme A. Interfering thiol-containing holo-ACP in the FabD assay was removed by precipitation with trichloroacetic acid before the probe was added. Fluorescence was measured at 470 nm. They applied the assays for the rapid determination of FabH and FabD activity.^642^

**Figure 78:**
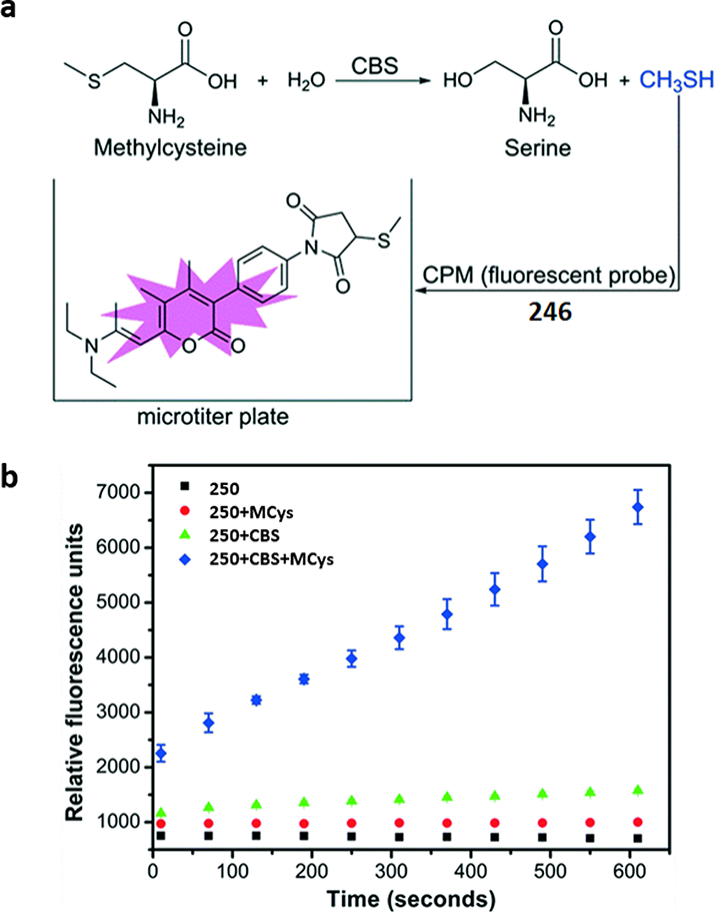
Illustration of the high-throughput assays for cystathionine β-synthase (CBS). (a) Methanethiol (CH_3_SH) generation catalyzed by CBS using methylcysteine (MCys) as a substrate was determined with the CPM **246**. (b) The CBS activity was monitored using 10 mM MCys as the substrate in the presence of 15 μM **246** and 2 μg of hCBS in a 200 μL reaction mixture containing 50 mM HEPES, pH 7.4. The fluorescence of the reaction mixture at 460 nm (λ_ex_ = 400 nm) was monitored for 600 s. Reproduced with permission from ref (640). Copyright 2017 Royal Society of Chemistry.

Niu *et al*. used **246** in an assay for HTS of potent and selective hCBS (human cystathionine β-synthase) inhibitors derived from natural compounds. The assay was based on the conversion of methylcysteine by cystathionine β-synthase (CBS), leading to the release of methanethiol, which was subsequently detected by **246** at physiological pH (Figure 78). With this approach, 6491 compounds were tested, and several compounds that inhibited hCBS activity were discovered, including sikokianin C, which exhibited cytotoxic activity against HT29 cancer cells with an IC_50_ value (half-maximal inhibitory concentration) of 1.5 uM. The assay also could be used for monitoring hCSE (human cystathionine γ-lyase) activity.^640^

Yi *et al*. designed the coumarin-based probe CME **247** with a maleimide moiety that showed a rapid response with high selectivity and sensitivity for thiols (<1 min for Cys) in Tris-HCl buffer (pH 7.4). It had a 470-fold increase in quantum yield upon conjugation with GSH. The assay’s sensitivity enabled the determination of GSH at concentrations as low as 0.5 nM. The water-soluble **247** could be efficiently synthesized in three steps and is stable for months in solution and in pure form. Yi *et al*. successfully used it to develop a high-throughput fluorescence assay for glutathione reductase.^643^

Gao *et al*. compared the performance of various fluorescent probes in assaying the activity of a histone acetyltransferase and concluded that probes **246** and **247** were well suited in a high-throughput format (fast reaction, fluorescence enhancement of >250-fold with coenzyme A).^558^

Zhou *et al*. developed the cationic probe **248**, consisting of a coumarin core linked to a methylpyridinium moiety by an unsaturated ketone. It enabled the detection of Cys at nM concentration under physiological conditions, based on a new assisted electrostatic attraction strategy, and showed a fast fluorescent and colorimetric response. They assumed that the pyridinium group confers high solubility in water, high reactivity with thiols (due to its electron-withdrawing properties), a fast response (due to electrostatic interactions), and good cell permeability. The nonfluorescent and water-soluble **248** showed a 148-fold increase in fluorescence emission upon the addition of Cys and reached the maximum fluorescence intensity within 1 min. Hcy and GSH reacted much slower with the probe than Cys, explained by the lower steric hindrance, p*K*_a_, and electrostatic attraction of Cys.^644^

Langmuir *et al*. developed several maleimide-containing probes (including **249**, **250**) that almost instantaneously reacted with thiols, such as ME, GSH, and *N*-acetyl-l-cysteine, under physiological pH. Upon addition of a thiol to the maleimide, the initially weak fluorescence of the probes was strongly enhanced.^639^

**Scheme 23:**
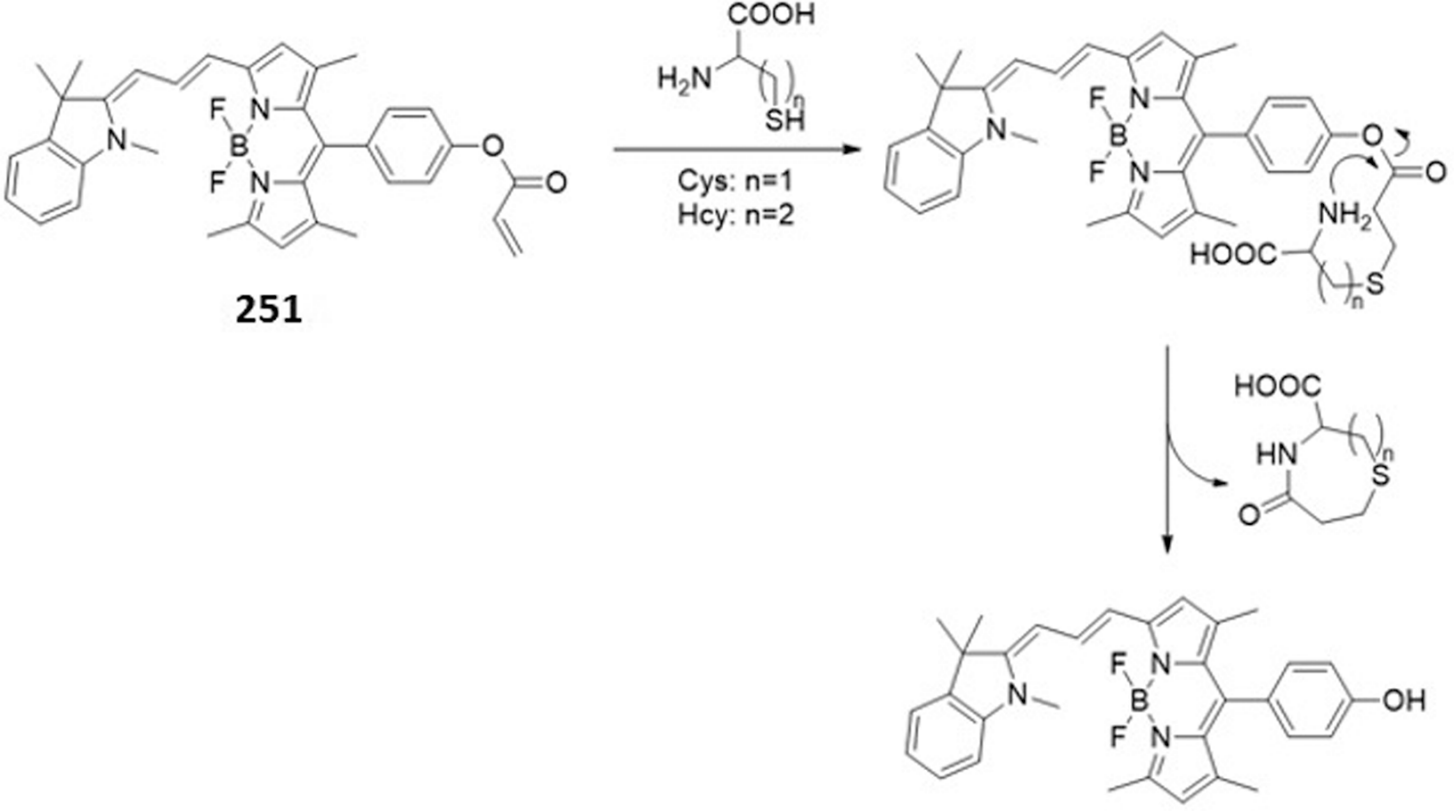
Proposed Response Mechanism for NIR-BODIPY-Ac **251** to Cys or Hcy

Li *et al*. reported on the development of the NIR probe NIR-BODIPY-Ac **251** for the selective detection of Cys in the presence of GSH and Hcy under neutral aqueous conditions. It consisted of an acrylate group as a reactive site and an indolium-BODIPY as NIR fluorophore. Upon conjugate addition of Cys/Hcy to the acrylate, an intramolecular cyclization led to the subsequent elimination of a cyclic amide (Scheme 23). This release prompted a strong increase in fluorescence emission. The probe is highly selective for Cys in the presence of Hcy, explained by the formation of a kinetically more favored, seven-membered ring with Cys. The probe was used to detect exogenous and endogenous Cys in *in vitro* and *in vivo*.^645^

### 15.8 Sulfur–Metal Interaction

**Figure 79:**
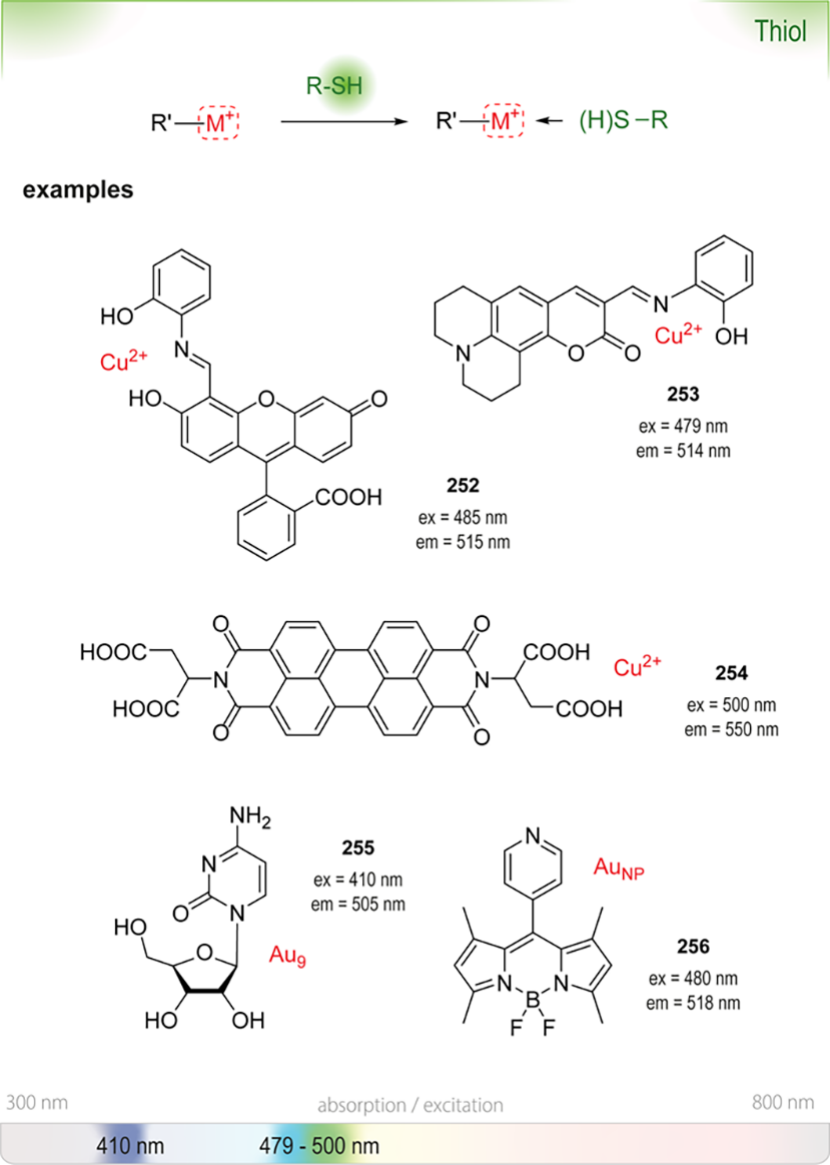
Summary of fluorogenic turn-on probes for the selective detection of thiols-based sulfur–metal interactions. Ex or em: wavelengths typically used for excitation and emission in fluorescence measurements. The wavelengths of probes in this section are shown in the strip at the bottom representing the visible spectrum.

**Figure 80:**
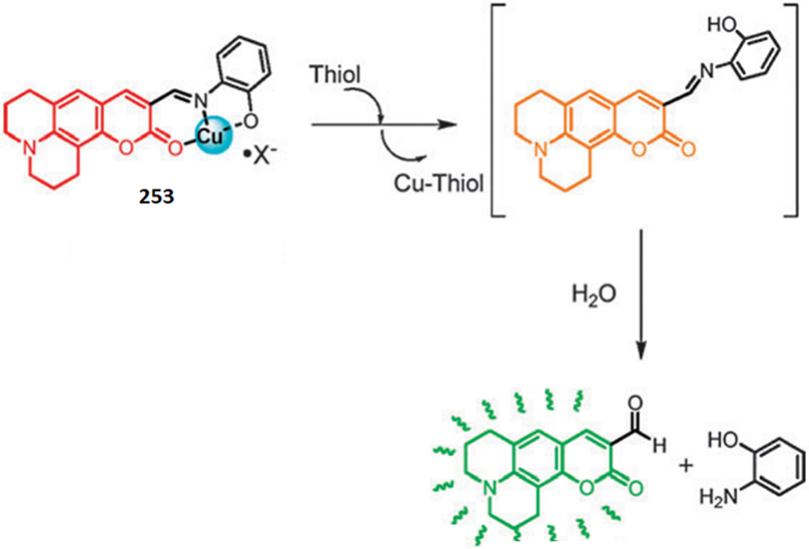
Schematic illustration of the thiol detecting chemodosimetric mechanism iminocoumarin-Cu^2+^ probe **253** in aqueous media. Adapted with permission from ref (654). Copyright 2011 The Royal Society of Chemistry.

Many assays rely on the high affinity of thiols toward metals and various probes that build upon these interactions with Ag, Cu, Hg, and Au, have been reported (Figure 79).^646−648^ Apart from concepts based on displacement, assays also use oxidation and reduction of metals.^649−652^ Jung *et al*. and Wang *et al*. developed probes **252** and **253** with Cu^2+^ ensembles for the detection of biogenic thiols (Figure 80). Both probes showed a fast response and a high selectivity toward thiols, in the presence of amino acids, in aqueous solution under neutral or alkaline conditions. It was inferred that in the presence of thiols, a decomplexation of Cu^2+^ from the iminofluorescein-Cu^2+^**252** probe or the iminocoumarin-Cu^2+^ probe **253** took place, followed by hydrolysis of the probe to yield the fluorescent fluoresceinaldehyde or coumarinaldehyde.^653,654^

Many assays based on thiol–metal interactions use metal nanoparticles or nanoclusters. Based on silver nanoparticles and fluorescent carbon dots, a colorimetric and fluorescent assay for the activity of AChE with acetylthiocholine as a substrate was developed by Zhao *et al*.^655^ The fluorescence of the carbon dots was quenched by the absorption of the dispersed nanoparticles (inner filter effect). The high affinity of the formed thiocholine for the silver nanoparticles and electrostatic interactions led to an aggregation, reducing inner filter effect and leading to a change of fluorescence (LOD: 0.016 mU mL^–1^) and color (LOD: 0.021 mU mL^–1^).

Polyethyleneimine-protected copper nanoclusters, or a water-soluble perylene derivative in combination with Cu^2+^, were used for the detection of thiols, measuring the activity of AChE, and for screening inhibitors.^656,657^ The mechanism for thiols was based on quenching the strong fluorescence of the polyethyleneimine-protected nanoclusters by binding of Cu^2+^ to the amino groups of polyethyleneimine. In the presence of thiols, however, the high affinity of Cu^2+^ to thiols led to the formation of thiol–Cu^2+^ complexes instead, which resulted in an increased fluorescent signal.^656^ Similarly, adding Cu^2+^ quenched the fluorescence of the perylene diimide derivative **254** by binding of Cu^2+^ to the carboxylic groups. Capture of the Cu^2+^ ions coordinated to the probe by thiols enhanced the fluorescence.^657^ Both probes enabled the determination of AChE activity with a detection limit of 1.38 mU mL^–1^ and 1.78 mU mL^–1^, respectively.^656,657^

Jiang *et al*. developed a sensitive, photoluminescent probe based on cytidine-stabilized Au nanoclusters **255** for rapid measurement of glutathione reductase activity and screening of its inhibitors. Although the photoluminescence of metal nanoclusters is often quenched by thiols, their probe showed enhanced photoluminescence. The authors speculated that the increase might result from the formation of smaller, fluorescent Au species (including Au_2_) etched by thiols. The probe enabled them to detect GSH at nM concentrations (LOD 2.0 nM).^169^

**Figure 81:**
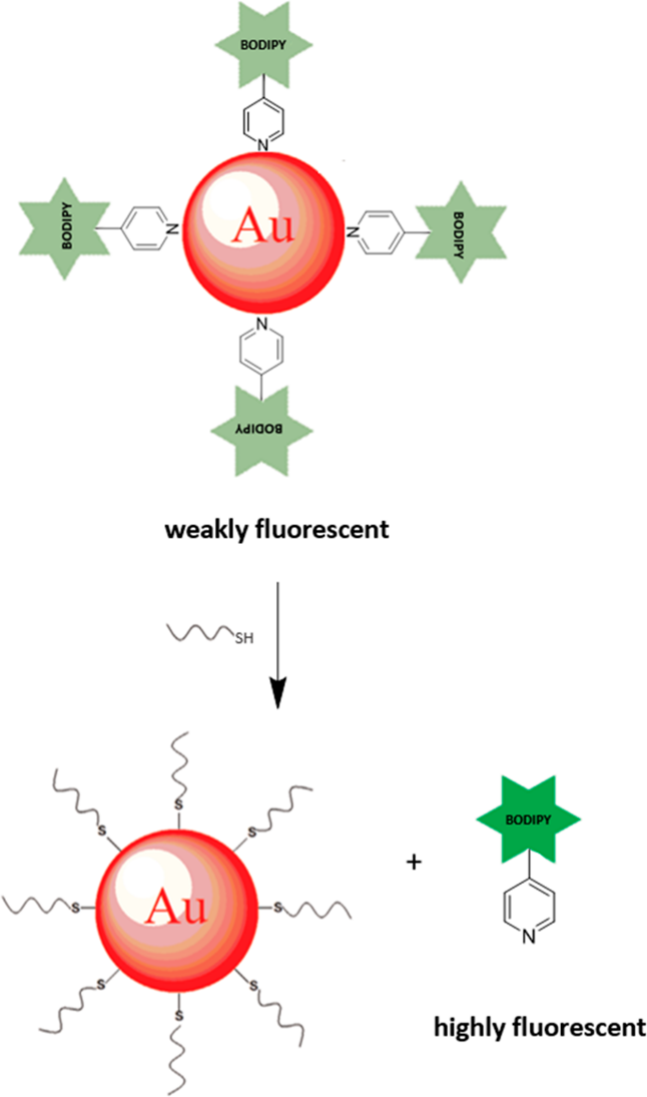
Schematic representation of the thiol sensor **256** based on modulation of the fluorescence quenching of the BODIPY chromophore by AuNPs. Reproduced with permission from ref (658) . Copyright 2016 Elsevier BV.

A displacement assay based on BODIPY-Au nanoparticles **256** was reported by Xu *et al*. The BODIPY chromophores on the Au nanoparticle (AuNP) surfaces could be displaced by thiols, thereby restoring the fluorescence of the BODIPY molecules (Figure 81). It showed a fast response for Cys and Hcy (∼2 min) in PBS buffer (pH 7.4), high selectivity and sensitivity (LOD: 30 nM for Cys) and was used for imaging thiols in living HeLa cells.^658^

### 15.9 Further Reaction Types

There are other types of thiol-sensing probes, based on reaction types such as 1,2 additions to *meso*-vinyl BODIPY derivatives,^659^ the addition of thiols to squaraine-like compounds,^573^ thiol-meditated reduction of quinone,^660^ thiol–halogen nucleophilic substitution reactions,^584,661−663^ or transesterification of thioesters.^664^ Chen *et al*. developed an NIR fluorescent probe for thiols (CHMC-thiol) that consisted of two reaction sites: the 2,4-dinitrobenzenesulfonate moiety was chosen as a high-sensitivity reaction site, and the less reactive chloro group was chosen as a low-sensitivity site. Low concentrations of cysteine (0–50 μM) led to the cleavage of the benzenesulfonate moiety and resulted in an increase in fluorescence at 680 nm. In the presence of high concentrations of cysteine (50–500 uM), the chlorine was substituted, followed by an intramolecular rearrangement to yield the amino product, and a ratiometric fluorescence response was obtained. This approach enabled the sensing of multiple concentration ranges *via* different fluorescence signals.^665^

## 16 Concluding Remarks

Enzymes have intrigued chemists to unleash their catalytic utility ever since their discovery. They provide an efficient and environmentally benign alternative to conventional organic–synthetic methodology due to their unparalleled selectivity. The surge in recent identifications and the potential for optimizing catalytically active proteins promoted their use in an industrial context. In particular, computationally designed proteins and directed evolution allowed chemists to explore uncharted territory in catalysis.

Directed evolution, however, requires sophisticated screening tools that can cope with the immense numbers of variants created in randomized approaches. Libraries containing millions or billions of variants need to be assessed in a time-effective way while maintaining accuracy in the screening process. The number of potential screening events thus imposes constraints in time and matter on the underlying analytical procedures. Every mechanical manipulation has an impact on throughput, making a direct detection in crude cell lysates the optimal choice. Molecular tools thus need to function in complex environments and reduce errors stemming from reactions with endogenous compounds to a minimum.

Fluorogenic substrates that mimic the natural or desired substrate offer a viable alternative to search for catalytic functionality but decouple the analytical signal from the synthetically relevant transformation. Functional group assays offer a more generalized approach for an optical read-out. By selective interaction with defined structural properties of analytes, these tools generate powerful handles to address the issues outlined above.

Finding and adapting these handles for compatibility to physiological conditions are the biggest challenges in the field of assay development, clearly shown in the distribution of probes presented here for the different substance classes. Some functional groups are more easily detected because of high reactivity (*e.g*., aldehydes, amines), while others have large numbers of reactive probes due to a strong scientific or applied interest (*e.g*., thiols). In general, assays that work under “real” screening conditions enable applications in HTS and are thus more likely to be used frequently.

Several limitations of functional group probes exist that limit the number of tools for detecting other (not highly reactive) substance classes *in situ*: low solubility in water, incompatibility with water, or high/low pH, slow reaction kinetics, especially at low product concentrations, insufficient selectivity, or undesirable optical properties. Nevertheless, there are designs that address all these disadvantages: Addition of PEG groups to **37** or incorporation of charged residues like quaternary ammonium or pyridinium groups to known fluorogenic dyes, such as **29** and **248**, have been used to increase their water solubility. Kinetical hurdles of ABAO reagents **126** were overcome by understanding the underlying reaction mechanism. Implementation of a methoxy group into the scaffold rendered the aldehyde selective tools also suitable for more challenging ketone detection. Selectivity issues for efficient discrimination between the GSH, Cys, and H_2_S were effectively overcome by designing the fluorogenic probe HMN **219**, which contained multiple reactive sites. The optical properties of pyrylium probes (*cf*. section 6.5) were also adjusted by modifications of their fluorophore, essentially making the assays applicable over the whole visible spectrum.

Understanding the mechanisms for the genesis of the optical signal and preserving structural features critical to the desired reactivity aids the development of good probes. Although some probes seem to be applicable for a broad set of analytes (*e.g*., nucleophiles targeting electrophilic reactive sites), the exact selectivity of most assays is generally not (extensively) characterized. It might thus prove useful for protein engineers to test a range of different assays.

We would like to encourage research groups working on new assays to characterize the selectivity toward various derivatives of the same substance or reactivity class (*e.g*., nucleophiles, electrophiles, oxidants) and with expected interference from cellular components. Only a small number of probes are commercially available or easily accessible by synthesis, particularly for some functional groups (*e.g*., alcohols, esters). We hope that this overview of available methods motivates the reader to tackle the remaining challenges, helping protein engineers to advance in their pursuit for new and better catalytic activity.

## Abbreviations

AA: amino acid
AAOx: aryl alcohol oxidase
AaeAPO: aromatic peroxygenase (*Agrocybe aegerita*)
AaeUPO: unspecific peroxygenase (*Agrocybe aegerita*)
ABAO: amino benzamidoxime
ABTS: 2,2′-azino-bis(3-ethylbenzothiazoline-6 sulfonic acid)
AChE: acetylcholinesterase
ACN: acetonitrile
ACP: acyl carrier protein
ADH: alcohol dehydrogenase
AHL: amidohydrolase
AIE: aggregation-induced emission
AKR: aldo-keto reductase
Alk: alkyl
AOC: antioxidant capability
AO/AOx: alcohol oxidase
Ar: aryl
ATA: amine transaminase
ATase: acyl transferase
AtHNL: hydroxynitrile lyase (*Arabidopsis thaliana*)
ATP: adenosine triphosphate
AuNCs: gold nanoclusters
AuNPs: gold nanoparticles
BChE: butyrylcholinesterase
BD: benzoxadiazole
BDHA: alcohol dehydrogenase (*Bacillus subtilis)*
BINOL: (1,1′-binaphthalene)-2,2′-diol
BmEH: epoxide hydrolase (*Bacillus megaterium*)
BnNH_2_: benzylamine
BphA: biphenyl dioxygenase
BSA: bovine serum albumin
(*n*)BuOH: butanol
BVMO: Baeyer–Villiger monooxygenase
CAR: carboxylic acid reductase
CBS: cystathionine β-synthase
CFE: cell-free extract
ChE: cholinesterase
CHEF: chelation-enhanced fluorescence
CoA/CoASH: coenzyme A
CQD: carbon quantum dot
CS: chitosan
CSA: salicylaldehyde azine derivative
CuPRAC: cupric ion reducing antioxidant capacity
Cys: cysteine
DCC: dicyclohexylcarbodiimide
*de*: diastereomeric excess
DERA: deoxyribose 5-phosphate aldolase
DMF: *N*,*N*-dimethylformamide
DMSO: dimethylsulfoxide
DNA: deoxyribonucleic acid
DNBS: 2,4-dinitrophenyl sulfonyl
DNPH: 2,4-dinitrophenylhydrazine
DO: dioxygenase
DPPH: 1,1-diphenyl-2-picrylhydrazyl
EDG: electron-donating group
*ee*: enantiomeric excess
EET: electronic energy transfer
EH: epoxide hydrolase
epPCR: error-prone PCR
ESIPT: excited-state intramolecular proton transfer
EtOH: ethanol
EtyGly: ethylene glycol
EWG: electron-withdrawing groups
ε: molar absorption coefficient
FA: formaldehyde
FabD: malonyl-coenzyme A transacylase
FabH: ketoacyl synthase III
FACS: fluorescence-activated cell sorting
FADS: fluorescence-activated droplet sorting
FC test: Folin–Ciocalteau test
FGA: functional group assays
FGI: functional group interconversion
FITC: fluorescein isothiocyanate
FRAP: ferric-reducing antioxidant power
FRET: Förster resonance energy transfer
GC: gas chromatography
GDH: glutamate dehydrogenase
GFP: green fluorescent protein
GSH: glutathione
hCBS: human cystathionine β-synthase
hCES: human cystathionine γ-lyase
Hcy: homocysteine
HHDH: halohydrin dehalogenase
HNL: hxdroxynitrile lyases
HPLC: high-performance liquid chromatography
HTA: high-throughput assay
HPTS: 8-hydroxypyrene-1,3,6-trisulfonic acid trisodium salt
HTS: high-throughput screening
IC: internal conversion
ICT: intramolecular charge transfer
*i*PrNH_2_: isopropylamine
IR: infrared
IRED: Imine reductase
ISC: intersystem crossing
ITC: isothiocyanate
KDC: keto acid decarboxylase
KIVD: 2-ketoisovalerate decarboxylase
K_M_: Michaelis–Menten constant
KpADH: diaromatic ketone reductase
KRED: ketoreductase
KS: ketone synthase
LDH: lactate dehydrogenase
LFER: linear free energy relationship
LG: leaving group
LOD: limit of detection
λ_abs_/abs: absorbance wavelength [nm]
λ_em_/em: emission wavelength [nm]
λ_ex_/ex: excitation wavelength [nm]
MAH: mandelamide hydrolase
MAO: monoamine oxidase
MCys: methylcysteine
MDA: malondialdehyde
MDH: mandelate dehydrogenase
ME: mercaptoethanol
MEK: methyl ethyl ketone
MeNH_2_: methylamine
MeNH_3_Cl: methylammonium chloride
MeOH: methanol
MO: monooxygenase
MS: mass spectrometry
μMS: microscale mass spectrometry
NAD^+^/NADH: nicotinamide adenine dinucleotide
NADP^+^/NADPH: nicotinamide adenine dinucleotide phosphate
NBD: nitrobenzoxadiazole
NCL: native chemical ligation
NHS: *N*-hydroxysuccinimide
NIR: near-infrared
NOS: reactive nitrogen species
Nuc: nucleophile
ORAC: oxygen radical absorbance capacity
PBS: phosphate-buffered saline
PcAOX: alcohol oxidase (Phanerochaete chrysosporium)
PCR: polymerase chain reaction
PEG: polyethylene glycol
PET: photoinduced electron transfer
PFP: pentafluorophenyl
PGM: phosphoglucomutase
PL: photoluminescence
PLP: pyridoxal phosphate
PMA: phosphomolybdic acid
PO: peroxygenase
(*n*)PrOH: propanol
PVC: polyvinyl chloride
Φ, Q_Y_: quantum yield
RA: reductive aminase
ROS: reactive oxygen species
RSS: reactive sulfur species
SHC: squalene hopene cyclase
S_NAr_: nucleophilic aromatic substitution
SQ: squaraine
TA: transaminase
*t*-BuOH: *tert*-butyl alcohol
TDO: toluene dioxygenase
TDP: thiamine diphosphate
TEAC: trolox equivalent antioxidant capacity
TEMPO: (2,2,6,6-tetramethylpiperidin-1-yl)oxyl
ThDP: thiamine diphosphate
TICT: twisted intramolecular charge transfer
TLC: thin-layer chromatography
*p*-TsOH: *p*-toluenesulfonic acid
TsrE: monooxygenase
TTN: total turnover number
TYR: tyrosinase
uHTS: ultra-high-throughput screening
UV: ultraviolet
VIS: visible
w/o/w: water-in-oil-in-water
YerE: ThDP-dependent flavoenzyme (*Yersinia pseudotuberculosis*)
